# Comparative analysis of atherosclerotic cardiovascular disease burden between ages 20–54 and over 55 years: insights from the Global Burden of Disease Study 2019

**DOI:** 10.1186/s12916-024-03527-4

**Published:** 2024-07-19

**Authors:** Ziyi Li, Yucheng Yang, Xuechen Wang, Na Yang, Liyun He, Jialu Wang, Fan Ping, Lingling Xu, Huabing Zhang, Wei Li, Yuxiu Li

**Affiliations:** 1 Department of Endocrinology, Key Laboratory of Endocrinology of National Health Commission, Translation Medicine Center, Peking Union Medical College Hospital, Chinese Academy of Medical Sciences & Peking Union Medical College, No.1 Shuaifuyuan, Beijing, Wangfujing, Dongcheng District China; 2 Diabetes Research Center of Chinese Academy of Medical Sciences, Beijing, China

**Keywords:** Premature atherosclerotic cardiovascular disease, Extremely premature atherosclerotic cardiovascular disease, Ischemic heart disease, Ischemic stroke, Peripheral artery disease, Global disease burden

## Abstract

**Background:**

To systematically analyze differences in atherosclerotic cardiovascular disease (ASCVD) burden between young and older adults.

**Methods:**

We estimated the prevalence, mortality, and disability-adjusted life years (DALYs) of ASCVD, including ischemic heart disease (IHD), ischemic stroke (IS), and peripheral artery disease (PAD), in individuals aged 20–54 and > 55 years from 1990–2019, utilizing data from the 2019 Global Burden of Disease Study. The annual percentage changes (EAPCs) for age-specific prevalence, mortality, or DALY rates were calculated to quantify the temporal trends of ASCVD burden. We also analyzed population attribution fractions (PAF) of premature ASCVD mortality and DALYs for different risk factors and compared the burden of extremely premature, premature, and non-premature ASCVD cases based on clinical classifications.

**Results:**

From 1990–2019, the global prevalence rates of IHD, IS, and PAD in the 20–54 years age group increased by 20.55% (from 694.74 to 837.49 per 100,000 population), 11.50% (from 439.48 to 490.03 per 100,000 population), and 7.38% (from 384.24 to 412.59 per 100,000 population), respectively. Conversely, the ASCVD prevalence in > 55years age group decreased. Adverse outcome burdens, including mortality and DALYs, varied among ASCVD subtypes. The decrease in the mortality/DALY burden of IHD and IS was lower in the 20–54 years group than in the > 55 years group. For PAD, DALYs among those aged 20–54 increased but decreased among those aged > 55 years. When grouped according to socio-demographic index (SDI) values, lower SDI regions exhibited a higher proportion of young ASCVD burden. The prevalence of young IHD, IS, and PAD in low SDI regions reached 20.70%, 40.05%, and 19.31% in 2019, respectively, compared with 12.14%, 16.32%, and 9.54%, respectively, in high SDI regions. Metabolic risks were the primary contributors to the ASCVD burden in both age groups. Increased susceptibility to ambient particulate matter pollution and inadequate control of high body-mass index and high fasting plasma glucose in young individuals may partially explain the differing temporal trends between young and older individuals.

**Conclusions:**

The ASCVD burden in young individuals may become a growing global health concern, especially in areas with lower socioeconomic development levels that require more effective primary prevention strategies.

**Supplementary Information:**

The online version contains supplementary material available at 10.1186/s12916-024-03527-4.

## Background

Atherosclerotic cardiovascular diseases (ASCVDs), including ischemic heart disease (IHD), ischemic stroke (IS), and peripheral arterial disease (PAD), are the most common cardiovascular diseases [1]. While previous studies have reported the global burden and trends of cardiovascular diseases (CVDs) from 1990–2019 [2, 3], the specific global disease burden of ASCVDs, distinguished by the unique pathogenesis of CVD and their status as the leading cause of global mortality and disability, remains incompletely documented.

Although the majority of the ASCVD burden occurs in older individuals, the risk of ASCVD in young individuals is increasing [4]. Studies indicate that atherosclerosis can begin early in life, and subclinical atherosclerosis in youth significantly increases the risk of subsequent all-cause mortality [5, 6]. Estimates suggest that the prevalence of ASCVD in young adults ranges from 7 to 30%, depending on different age groups, subtypes of ASCVD, and geographic regions [7–10]. Owing to the relatively young age at the time of diagnosis, ASCVD in younger age groups impose a great financial burden on the healthcare system. No study has precisely quantified the proportion of the total ASCVD burden attributable to young individuals.

Focusing on risk factors is crucial to reduce the burden of ASCVD. Previous research has suggested that > 90% of the population-attributable risk of myocardial infarction can be attributed to traditional cardiovascular risk factors [11]. Since the World Health Organization established a target for preventable risk factors to reduce ASCVD mortality by 25% from the 2010 levels by 2025 [12], incidence and mortality rates of ASCVD have declined [13]. However, a similar decline has not been observed among individuals with early-onset ASCVD [14]. Consequently, uncertainty persists regarding whether the disparity between age groups stems from varying changes in risk factors. Additionally, identifying age-specific ASCVD risk factors is vital for devising tailored intervention strategies.

To address this knowledge gap, our objective was to compare the burden of ASCVD between younger and older populations. This entails elucidating the global epidemiological characteristics of ASCVD burden in individuals aged 20–54 years and those > 55 years. Furthermore, we compared the burden associated with extremely premature, premature, and non-premature ASCVD, as defined by clinical classifications. This investigation included a comparison of disease burden trends across various ASCVD subcategories. To inform targeted public health strategies, we aimed to identify and describe the risk factors contributing to both demographic groups.

### Methods

#### Overview of the data sources

The 2019 Global Burden of Disease Study (GBD) estimated the disease burden of various disorders across 204 countries and territories [15]. Data were collected through systematic assessment of censuses, household surveys, civil registrations, vital statistics, disease registries, and other methods. Mortality data were analyzed using the Cause of Death Ensemble model (CODEm) to assess the death toll as accurately as possible.[16], whereas disability-adjusted life years (DALYs) were estimated using the DisMod-MR 2.1 a Bayesian meta-regression tool [17]. The world was divided into 21 super-regions based on epidemiological similarities and geographical proximity to analyze the disease burden across locations [18]. Meanwhile, socio-demographic index (SDI) was utilized to classify the countries and territories [19].

#### Estimation of ASCVD disease burden in young and older individuals

As IHD, IS, and PAD represent the primary components of ASCVD, the annual data for these three ASCVD subtypes were analyzed separately. According to the age ranges provided by the 2019 GBD database, we defined young group (age 20–54 years) and old group (age > 55 years). In addition, to further clarify the burden of ASCVD in different age groups, we further defined extremely premature ASCVD (age < 40 years), premature ASCVD (age < 55 years in males; age < 65 years in females), and non-premature ASCVD (age ≥ 55 years in males; age ≥ 65 years in females). New age group data were synthesized by multiplying the crude rates of each 5-year age group by the proportion of that group’s population under the standard population provided by the GBD database [18]. We obtained data on the prevalence, DALYs, and mortality rates of IHD, IS, and PAD according to sex and region from the 2019 GBD study. Relevant data were retrieved from the following website (https://vizhub.healthdat.org/gbd-results/).

#### Estimation of attributable risk factors for ASCVD

Metabolic, behavioral, and environmental factors contribute to the risk of ASCVD. A total of 27 subcategories of risk factors were related to ASCVD in the 2019 GBD study. Under the GBD framework, there is a theoretical minimum exposure level (TMREL) at which the risk to health outcomes are lowest. The risk factors and definitions are provided in the Appendix (Additional file 1:Table S1).

#### Statistical analysis

Descriptive analysis characterized the ASCVD burden, comparing the prevalence, mortality, and DALY rates in 1990 and 2019, both in terms of the number of cases and rates per 100,000 population. The estimated annual percentage change (EAPC), calculated using a generalized linear model with a Gaussian distribution to describe the age-specific rate (ASR) trend over time, was used to quantify the trends of this burden [19, 20]. We also explored the ASCVD burden across 204 countries and territories of the world. The relationships between the rates and SDI were evaluated using Pearson’s correlation. Population attribution fractions (PAFs) of ASCVD deaths and DALYs for different risk factors were analyzed. The 95% confidence interval (CI) based on 1000 draws from the posterior distribution of each step in the estimation process were calculated using the 2.5th and 97.5th percentiles of the ordered 1000 values.

Data analysis and illustrations were performed using R software version 4.0.2, and *p*-values < 0.05 were considered statistically significant.

## Results

### ASCVD burden among individuals aged 20–54 years

**Fig. 1:**
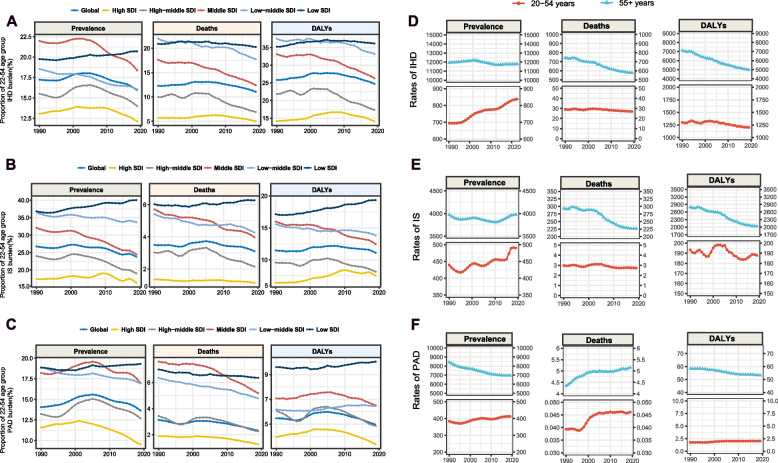
Global burden of ASCVD in the younger and the older. **A** Proportion of people aged 20–54 in total IHD burden. **B** Proportion of people aged 20–54 in total IS burden. **C** Proportion of people aged 20–54 in total PAD burden. **D** The rates of global IHD burden in people aged 20–54 and older than 55 during 1990 and 2019. **E** The rates of global IS burden in people aged 20–54 and older than 55 during 1990 and 2019. **F** The rates of global PAD burden in people aged 20–54 and older than 55 during 1990 and 2019.ASCVD: atherosclerotic cardiovascular disease; IHD: ischemic heart disease; IS: ischemic stroke; PAD: peripheral artery disease; SDI: socio-demographic index; DALYs: disability-adjusted life years

In 2019, there was an estimated total of 197.22, 77.19 and 113.44 million cases of IHD, IS, and PAD, respectively. Among them, 15.93%, 23.82%, and 13.65% of IHD, IS, and PAD cases, respectively, occurred in individuals aged 20–54 years. Regarding mortality rate, IHD accounted for the highest number of ASCVD-related mortality with 9.14 million deaths in 2019, followed by IS with 3.29 million deaths and PAD with 74,062 deaths. Among these, 11.08% of IHD mortality, 3.10% of IS mortality, and 2.34% of PAD mortality occurred in the 20–54 years age group. Meanwhile, individuals aged 20–54 years accounted for 24.74% for IHD DALYs, 11.10% for IS DALYs, and 4.96% for PAD DALYs. (Fig. 1A–C).

The proportion of younger patients with ASCVD was greater in regions with lower SDI values. In 2019, the prevalence of younger IHD, IS, and PAD in low SDI regions reached 20.70%, 40.05%, and 19.31%, respectively. The differences in mortality rates and DALYs across regions were also similar to the differences in prevalence (Fig. 1A–C).The incidence of early-onset ASCVD was higher in males than in females (Additional file 1: Figure S1–S2).

### Temporal trends in global ASCVD burden: 1990–2019 comparison between individuals aged 20–54 and > 55 years

It was estimated that the global prevalence rates of IHD, IS, and PAD in the 20–54 years age group increased by 20.55% (from 694.74 to 837.49 per 100,000 population), 11.50% (from 439.48 to 490.03 per 100,000 population), and 7.38% (from 384.24 to 412.59 per 100,000 population) across 1990–2019, respectively. Meanwhile, the global prevalence rates of IHD, IS, and PAD in individuals aged > 55 years decreased by 1.26%, 0.13%, and 17.26%, respectively (Additional file 1:Table S2–S7, Fig. 1D–F).

The burden of adverse outcomes, including mortality rates and DALYs, varied among ASCVD subtypes. The decline in adverse outcomes for IHD and IS was more significant in individuals aged > 55 years than in the younger group. For PAD, DALYs increased among those aged 20–54 years but decreased among those aged > 55 years. (Additional file 1:Table S2–S7, Fig. 1D–F).

### ASCVD burden for individuals aged 20–54 and > 55 years across locations in 2019

**Fig. 2:**
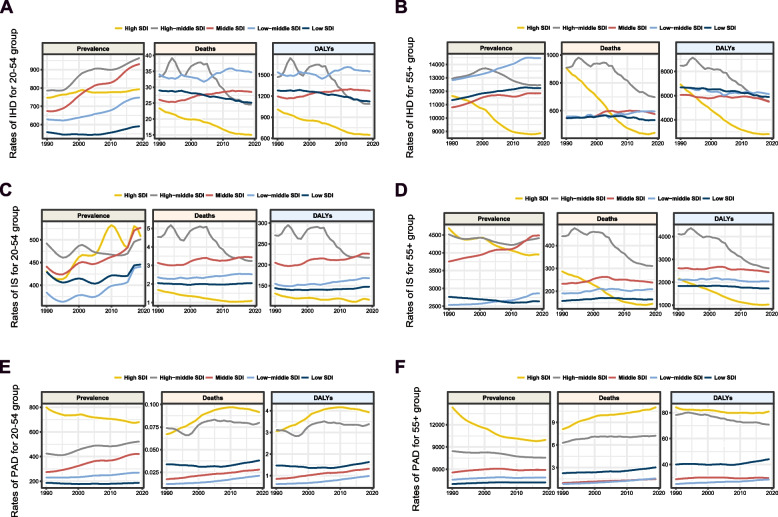
The rate of ASCVDs burden by SDI during 1990–2019. **A** The rate of IHD burden in people aged 20–54 during 1990–2019. **B** The rate of IHD burden in people older than 55 during 1990–2019. **C** The rate of IS burden in people aged 20–54 during 1990–2019. **D** The rate of IS burden in people older than 55 during 1990–2019. **E** The rate of PAD burden in people aged 20–54 during 1990–2019. **F** The rate of PAD burden in people older than 55 during 1990–2019. SDI: socio-demographic index; IHD: ischemic heart disease; IS: ischemic stroke; PAD: peripheral artery disease; DALYs: disability-adjusted life years

In 2019, the relationship between age-specific rates of prevalence, death, and DALYs of IHD and IS with SDI followed an inverse U-shaped curve in both the 20–54 and > 55 years age groups. Higher prevalence rates were observed in countries with a high-middle SDI, whereas higher mortality/DALY rates were observed in countries with a low-middle SDI. Additionally, the age-specific rates of death and DALYs of PAD in the > 55 years age group were positively correlated with SDI (*P* < 0.05), which was not observed in the 20–54 years age group (Fig. 2, Additional file 1: Figure S3–S5).

At the regional level, similar distribution characteristics were observed in the ASCVD burden groups aged 20–55 and > 55 years. Eastern Europe had the highest mortality rates and DALYs attributable to IHD, IS, and PAD in 2019. Meanwhile, the highest prevalence of IHD in younger patients was observed in North Africa and the Middle East (Additional file 1: Table S2–S7). Tables S8–S10 present the ASCVD burden in the 20–54 and > 55 age groups across countries and territories.

### Temporal trends in ASCVD burden across regions: 1990–2019 comparison between individuals aged 20–54 and > 55 years

The prevalence rate of IHD in individuals aged 20–54 increased across all five SDI categories from 1990 to 2019, with the highest EAPC observed in the middle SDI regions (1.20 [95% confidence interval [CI]: 1.14–1.27]). Conversely, during the same period, the increase of IHD cases among older individuals in high- and high-middle SDI regions was mitigated (EPAC < 0). Although there was a significant decline in IHD mortality and DALY rates among the 20–54 years age group in high-SDI regions over the past 30 years, it was comparatively smaller than that observed in the > 55 years age group (Additional file 1: Tables S2–S3, Fig. 2). The temporal trends of IS burden across SDI regions mirrored those of IHD (Additional file 1: Tables S3 and S6 and Fig. 2).

The prevalence rate (EAPC: -1.24 (95% CI: -1.39 to -1.10) and DALYs (EAPC: -0.15 (95% CI: -0.19 to -0.12)) of PAD in the > 55 years age group in high SDI regions decreased over the past 30 years, whereas the burden of PAD in the 20–54 years age group increased in almost all sub-SDI regions, especially in the middle and low-middle SDI regions (EAPC for PAD prevalence in middle SDI regions: 1.57 [95% CI: 1.48–1.67]; EAPC for PAD mortality in low-middle SDI regions: 2.12 [95% CI: 2.00–2.25]; EAPC for PAD DALYs in low-middle SDI regions: 1.76 [95% CI: 1.64–1.88]) (Additional file 1: Table S2 and S5 and Fig. 2).

**Fig. 3:**
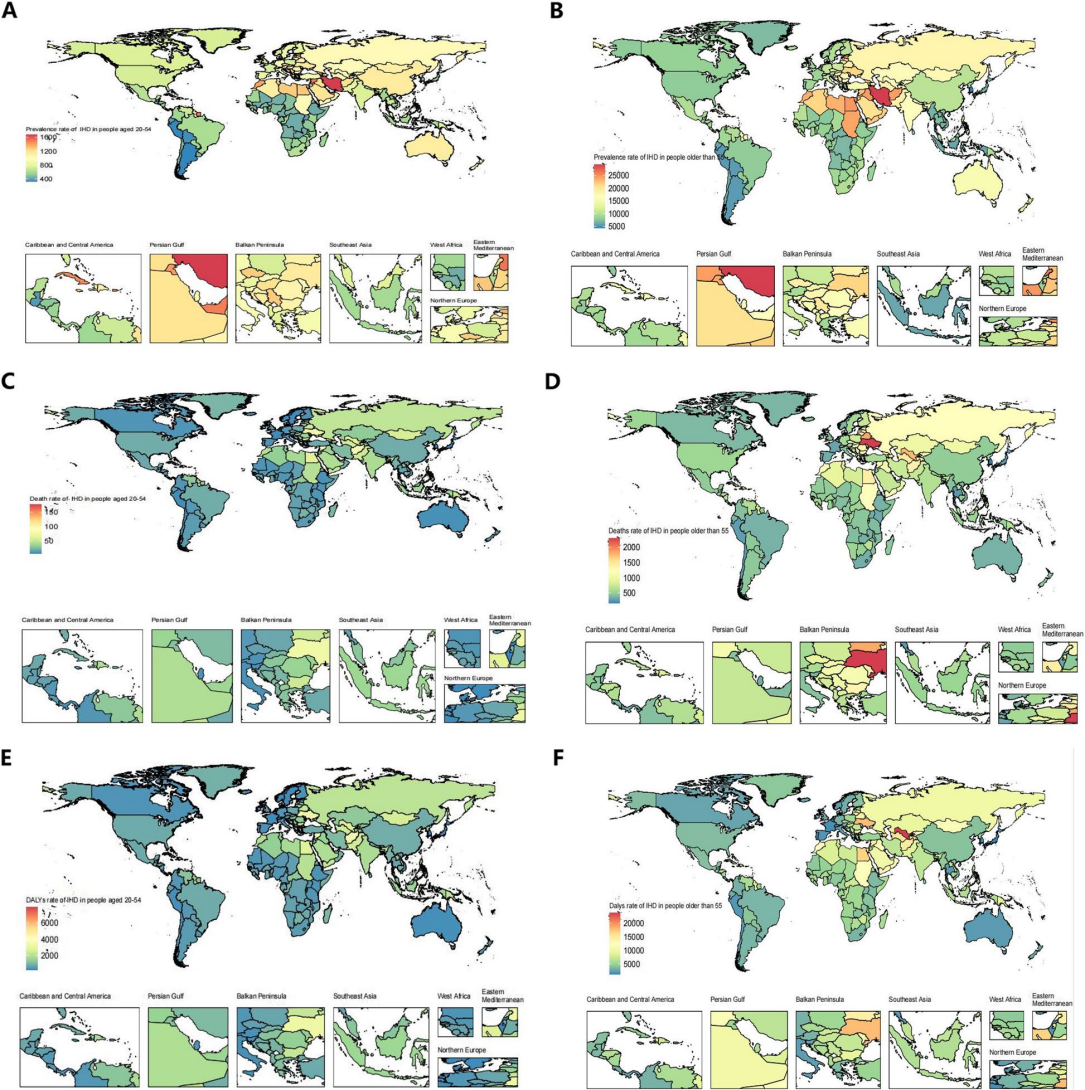
The change of IHD burden by countries and territories during 1990–2019. **A** The change of IHD prevalence cases in people aged 20–54 by countries and territories in 2019. **B** The change of IHD prevalence cases in people older than 55 by countries and territories in 2019. **C** The change of IHD death cases in people aged 20–54 by countries and territories in 2019. **D** The change of IHD death cases in people older than 55 by countries and territories in 2019. **E** The change of IHD DALYs cases in people aged 20–54 by countries and territories in 2019. **F** The change of IHD DALYs cases in people older than 55 by countries and territories in 2019. IHD: Ischemic heart disease. DALYs: disability-adjusted life years

**Fig. 4:**
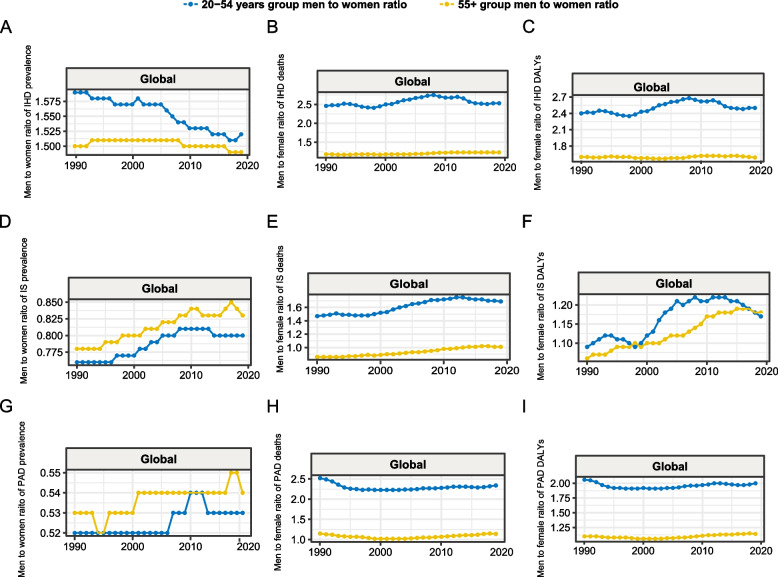
The man to women ratio of the rate of global ASCVDs burden during 1990–2019. **A** The man to women ratio of the rate of global IHD prevalence during 1990–2019. **B** The man to women ratio of the rate of global IHD death during 1990–2019. **C** The man to women ratio of the rate of global IHD DALYs during 1990–2019. **D** The man to women ratio of the rate of global IS prevalence during 1990–2019. **E** The man to women ratio of the rate of global IS death during 1990–2019. **F** The man to women ratio of the rate of IS DALYs during 1990–2019. **G** The man to women ratio of the rate of PAD prevalence during 1990–2019. **H** The man to women ratio of the rate of PAD death during 1990–2019. **I** The man to women ratio of the rate of PAD DALYs during 1990–2019. ASCVD: atherosclerotic cardiovascular disease; IHD: ischemic heart disease; IS: ischemic stroke; PAD: peripheral artery disease; SDI: socio-demographic index; DALYs: disability-adjusted life years

East Asia exhibited the most rapid increase in IHD prevalence burden during 1990–2019 both in the 20–54 and > 55 years age groups. Among the 20–54 years age group, high-income North America demonstrated the fastest increase in IS prevalence rate (EAPC: 1.52 [95% CI: 1.19–1.86]), whereas East Asia had the fastest increase among the > 55 years age group (EAPC: 1.40 [95% CI: 1.32–1.47]). Southeast Asia had the fastest increase in mortality and DALY rates for IHD among the 20–54 years age group, with East Asia having the fastest increase in among the > 55 years age group. Both the 20–54 and the > 55 years age group in Southeast Asia had the fastest increase in adverse outcomes for PAD (Tables S4 and S7 and Fig. 3). The temporal trends of ASCVD burden in both age groups according to country and territory are presented in Fig. 4 and Table S11–S12 and Figure S6–S7 in Additional file 1.

### ASCVD burden by sex and age

In 2019, global rates of prevalence, death, and DALYs for ASCVD were higher in males than in females in both the 20–54 and > 55 years age groups, except for the prevalence rates of IS and PAD. The male-to-female ratio of the ASCVD burden in the 20–54 years age group globally and in most SDI regions was significantly higher than that in the > 55 years age group. Notably, the male-to-female ratio of ASCVD burden rates varied over time, with the sex gap in the 20–54 years age group decreasing in high SDI regions and increasing in in low and low-middle SDI regions. Simultaneously, the male-to-female ratio of the ASCVD burden in the > 55 years age group remained stable in most regions (Fig. 4, Additional file 1: Figure S8). Overall, the age-specific prevalence, mortality, and DALY rates for all three types of ASCVDs increased with age, both globally and sub-SDI regionally (Additional file 1: Figures S9–S12).

### Attributable risk factors for death and DALYs of ASCVD in individuals aged 20–54 and > 55 years

**Fig. 5:**
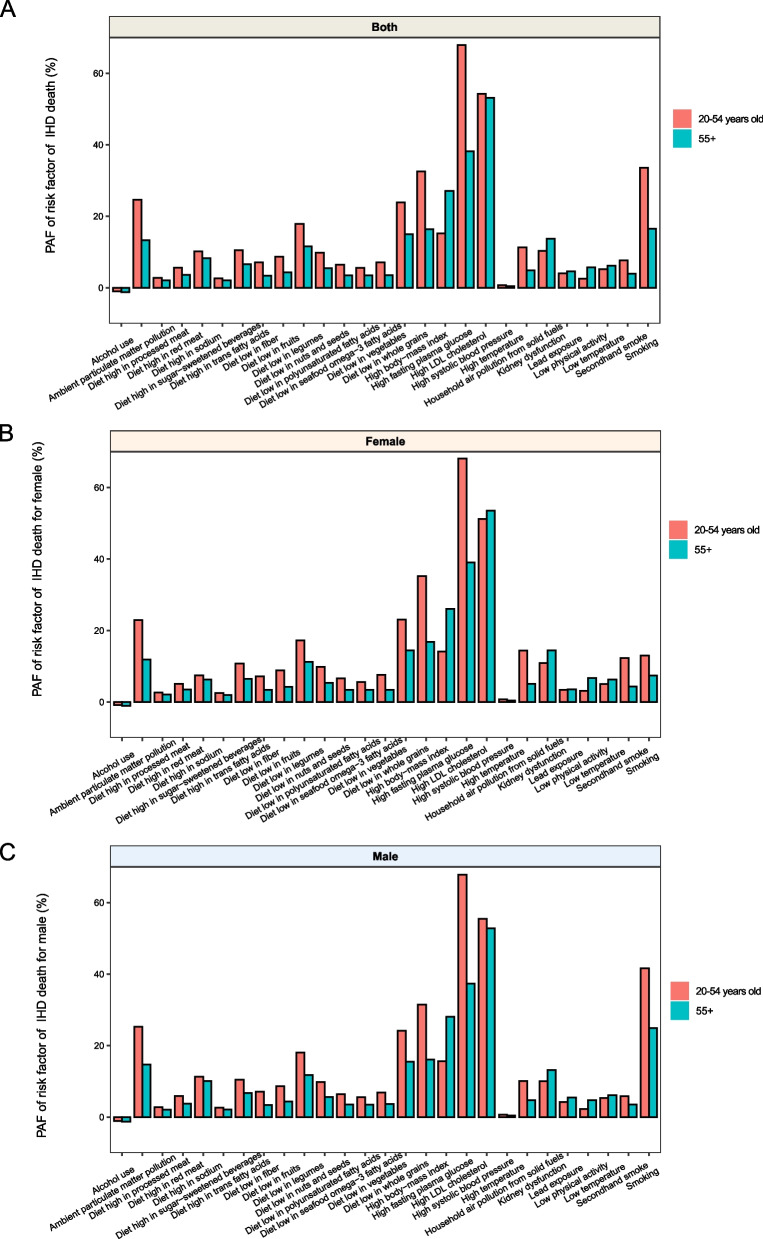
PAF of risk factor for IHD death for different gender. **A** PAF of risk factor for IHD death for both genders. **B** PAF for risk factor of IHD death for women. **C** PAF of risk factor for IHD death for male. PAF: population attribution fractions

Metabolic and behavioral risk factors drove changes in the overall ASCVD burden both in the 20–54 and > 55 years age groups during 1990–2019 (Additional file 1: Figure S13–S16). Most risk factors exerted a greater influence on the 20–54 years group than on the > 55 years age group, except for secondhand smoking, fasting plasma glucose level, high systolic blood pressure, low physical activity, and lead exposure. In addition, the contribution of risk factors varied between sexes in the 20–54 and > 55 years age groups. For instance, the contribution of high body-mass index was higher in females than in males, whereas the contribution of hypertension was higher in males. Males were also more likely to be affected by smoking, whereas females were more likely to be affected by exposure to secondhand smoking. (Fig. 5, Additional file 1: Figure S17–S21).

Regarding the burden of IHD and IS, mortality and DALY rates attributed to almost all risk factors in the > 55 years age group decreased significantly, with their annual change rates surpassing those observed in the 22–54 years age group. However, ambient particulate matter pollution, high body-mass index, and high fasting plasma glucose-induced IHD and IS burdens in the 20–54 years age group have increased over the past 30 years. In particular, high fasting plasma glucose- and high systolic blood pressure-induced PAD burden increased in the 20–54 years age group, whereas lead exposure-induced PAD burden increased in the > 55 years age group (Additional file 1: Figures S22–S27).

### Comparison of disease burden among extremely premature (age < 40 years), premature (age < 55 years in males; age < 65 years in females), and non-premature ASCVD (age ≥ 55 years in males; age ≥ 65 years in females)

The burden of premature ASCVD (age < 55 years in males; age < 65 years in females) and non-premature ASCVD (age ≥ 55 years in males; age ≥ 65 years in females) were attributed to IHD, IS, and PAD. However, the burden of extremely premature ASCVD (age < 40 years) was only attributed to IHD and IS. By 2019, premature IHD comprised 16.80% of all IHD prevalence cases in males, with extremely premature cases accounting for 2.72%. Meanwhile, premature and extremely premature IS represented 28.30% and 12.49% of total IS prevalence cases in males, respectively. Additionally, premature PAD accounted for 14.43% of the PAD prevalence in males. The proportions of premature and extremely premature ASCVD have decreased over the past 30 years (Figure S28). The proportion of premature ASCVD prevalence in females (IHD: 34.05%; IS: 45.31%; PAD: 33.86%) was higher than that in males (IHD: 16.08%; IS: 28.03%; PAD: 14.43%) (Additional file 1: Figure S28). Furthermore, the burden of premature and extremely premature ASCVD increased as the SDI decreased (Additional file 1: Figure S29–S31).

Different ASCVD subtypes exhibited varying trends in disease burden. In terms of IHD, non-premature cases saw a decline from 1990 to 2019 globally (female EAPC: -0.1 [95% CI: -0.12 to -0.09]; male EAPC: -0.13 [95% CI: -0.18 to -0.09]), whereas premature and extremely premature IHD cases increased, with premature IHD showing a steeper rise in both sexes. Over the past three decades, the prevalence of IS has increased across all groups, except for non-premature IS in females (EAPC: -0.08 [95% CI: -0.15 to -0.02]). Particularly, the global prevalence of premature IS has increased faster than extremely premature IS across both sexes. In contrast to the decline in non-premature PAD (female EAPC: -0.71 [95% CI: -0.78 to -0.64]; male EAPC: -0.55 [95% CI: -0.59 to -0.5]), the global prevalence of premature PAD increased. Regarding the burden of mortality and DALY rates, there was a significant decrease in non-premature and extremely premature ASCVD among females, whereas the burden of premature ASCVD remained unchanged or increased over the past 30 years. Meanwhile, the risk of premature ASCVD mortality and DALYs in males continued to increase (EAPC > 0), contrasting with the non-premature populations (Additional file 1: Table S13–S26, Additional file 1: Figure S32–33).

Different SDI regions demonstrated different trends in ASCVD burden. Although the prevalence of non-premature ASCVD in high SDI areas decreased, the prevalence of non-premature ASCVD in low-middle and middle SDI areas continued to increase for both sexes. Specifically, the prevalence of premature and extremely premature ASCVD increased in all SDI regions from 1990 to 2019, with the fastest-growing regions being the middle or low-middle SDI regions (Additional file 1: Tables S13–S26, Figures S34–S36).

## Discussion

Although the majority of the ASCVD burden occurs in older individuals, the ASCVD burden in young individuals is becoming increasingly noteworthy owing to changes in risk factors [21–24]. Recent findings indicate a concerning trend of CVD events occurring at younger ages [3, 25]. Approximately one-seventh of CVD events in females and a quarter of CVD events in males occur before the age of 55 years [26]. To the best of our knowledge, this study is the first to evaluate the global prevalence of premature ASCVD. In the present study, we found that, by 2019, 16.08%, 28.03%, and 14.43% of IHD, IS, and PAD cases, respectively, occurred in males aged < 55 years, whereas 34.05%, 45.31%, and 33.86% of IHD, IS, and PAD cases, respectively, occurred in females aged < 65 years. Additionally, regions with lower SDI demonstrated a higher proportion of premature ASCVD burden. Moving forward, prioritizing interventions for premature ASCVD in regions with lower SDI is imperative.

Over the past decade, public health strategies for ASCVD have impacted the global ASCVD burden [12]. However, small-scale population studies suggest that trends in the burden of early-onset ASCVD and ASCVD in older adults may differ [4, 6]. Previously, data on disease burden mostly analyzed the burden of a single specific age group, which may fail to provide a clear indication of whether public health strategies need to focus on specific groups of individuals [2, 3]. In this study, we compared the ASCVD burden between young and older individuals. We found that the mortality rates and DALYs of IHD and IS decreased in both age groups, but the decrease was smaller in the younger group. Notably, the prevalence of ASCVD in older adults has decreased owing to the global focus on chronic diseases. However, the number of young patients with ASCVD has been increasing globally, indicating an urgent need for measures to control the spread of ASCVD in young adults.

An important feature of this study was the division of the ASCVD burden into premature and non-premature groups according to clinical definitions. Previous studies have separately reported the CVD burden of young (age 15–39 years) and older (age > 70 years) groups [2, 3]; however, these findings may lack clinical relevance as the population was not divided according to clinical definitions. Hence, in this study, we conducted a comparative analysis of the burden of extremely premature, premature, and non-premature ASCVD in males and females according to specific guidelines, providing new evidence for clinical practice.

Interventions in risk factors might partially explain the trend differences in ASCVD burden between young and older individuals. Metabolic risk factors stand out as pivotal contributors to the global burden of IHD and IS. Over the past decades, the global disease burden attributable to a high body-mass index has more than doubled [27], and the mortality and DALY rates due to high systolic blood pressure increased by 54.1% [28]. Simultaneously, the prevalence rate of metabolic factors in young individuals has gradually been increasing [29]. Therefore, the control and management of metabolic risk factors are essential to reduce the ASCVD burden in young individuals. Our research suggests a deficiency in the past control of risk factors among this demographic. While the reduction in ASCVD burden caused by certain risk factors is significantly lower in young individuals than in older individuals, the burden of ASCVD caused by ambient particulate matter pollution, high body-mass index, and high fasting plasma glucose is still increasing among the young population. Drawing from the experiences of preventing and controlling risk factors in older individuals, it is evident that a significant portion of the ASCVD burden in young individuals can be prevented. Therefore, implementing targeted prevention strategies for risk factors in young individuals deserves greater attention.

The burden of ASCVD differs between young and older individuals in terms of sex differences. Males generally exhibit a higher rate of mortality and DALY burden of IHD, IS, and PAD compared with females, regardless of age, and this could be attributed to the cardioprotective effects of estrogen [30]. However, the differences between sexes in ASCVD are more pronounced among the young demographic. Behavioral factors and the accessibility of medical resources may contribute to these variations across age groups. Notably, the disparity in smoking and alcohol consumption between sexes is more prominent among young individuals [31, 32]. Additionally, young males may exhibit poorer medical compliance to drug therapy [33].

PAD exhibits distinct patterns in terms of burden changes compared with IHD and IS. The mortality rate due to PAD has increased in both young and older patients over the past 30 years. This contrasting trend may be due to the lower awareness of PAD compared with IHD or IS. Only 25.8% of individuals are aware of PAD, which is significantly lower than the awareness of stroke and coronary artery disease [34]. Moreover, inadequate treatment for early PAD is another significant factor contributing to the increasing trends in mortality and DALY rates. In developed countries, approximately one-third to half of PAD patients fail to receive long-term statin and antiplatelet therapy [35]. Given these circumstances, there is an urgent need to increase attention to PAD and implement standardized diagnosis and treatment protocols for both young and older individuals.

### Limitations

To the best of our knowledge, this is the first study to comprehensively assess the difference in ASCVD burden between young and older individuals. These findings will be beneficial for public health policymakers. However, this study has some limitations. First, our attempt to categorize ASCVD burden based on age cutoffs of 55 years for males and 65 years for females to delineate premature ASCVD and < 40 years for extremely premature ASCVD may underestimate the burden of these conditions. The effects of premature and extremely premature ASCVD can persist beyond these age thresholds, leading to potential underestimation of their burden while possibly overestimating the burden of non-premature ASCVD. Second, as the ASCVD data within the 2019 GBD study were based on secondary data from existing registers, bias and gaps could not be avoided in the modeling process related to ASCVD burden estimation. Third, the accuracy of fatal and nonfatal ASCVD burdens depends on detection methods, screening quality, and data registry, which are influenced by socioeconomic factors. Consequently, the ASCVD burden in countries with lower SDI may be underestimated.

## Conclusions

Although the ASCVD burden on older individuals is decreasing, it is increasing among young individuals, particularly in areas with lower levels of socioeconomic development. The increasing susceptibility of young individuals to the impact of ambient particulate matter pollution, as well as their insufficient control of high body-mass index and high fasting plasma glucose, may contribute to the disparity in the temporal trends of the ASCVD burden between young and older individuals. This evidence forms the foundation for the development of targeted public health policies.

### Supplementary Information

Additional file 1: Table S1 Definition of risk factors. Table S2 IHD burden in people aged 20–54 across regions. Table S3 IS burden in people aged 20–54 across regions. Table S4 PAD burden in people aged 20–54 across regions. Table S5 IHD burden in people older than 55 + across regions. Table S6 IS burden in people older than 55 + across regions. Table S7 PAD burden in people older than 55 + across regions. Table S8 IHD burden in 204 countries and territories by 2019. Table S9 IS burden in 204 countries and territories by 2019. Table S10 The PAD burden in 204 countries and territories by 2019. Table S11 The temporal trends of ASCVDs burden in people aged 20–54 in 204 countries and territories. Table S12 The temporal trends of ASCVDs burden in people older than 55 in 204 countries and territories. Table S13 IHD burden in people under 40 across SDI regions. Table S14 IS burden in people under 40 across SDI regions. Table S15 IHD burden under 55 for male across SDI regions. Table S16 IS burden under 55 for male across SDI regions. Table S17 PAD burden under 55 for male across SDI regions. Table S18 IHD burden under 65 for female across SDI regions. Table S19 IS burden under 65 for female across SDI regions. Table S20 IHD burden older than 65 for female across SDI regions. Table S21 IHD burden older than 65 for female across SDI regions. Table S22 IS burden older than 65 for female across SDI regions. Table S23 PAD burden older than 65 for female across SDI regions. Table S24 IHD burden older than 55 for male across SDI regions. Table S25 IS burden older than 55 for male across SDI regions. Table S26 PAD burden older than 55 for male across SDI regions. Figure S1 Proportion of people aged 20–54 in total men ASCVDs burden during 1990–2019. Figure S2 Proportion of people aged 20–54 in total women ASCVDs burden during 1990–2019. Figure S3 Association between SDI and age-specific prevalence, death and DALYs rates of IHD in 2019. Figure S4 Association between SDI and age-specific prevalence, death and DALYs rates of IS in 2019. Figure S5 Association between SDI and age-specific prevalence, death and DALYs rates of PAD in 2019. Figure S6 The change of IS burden by countries and territories during 1990–2019. Figure S7 The change of PAD burden by countries and territories during 1990–2019. Figure S8 The men to women ratio of the rate of ASCVDs burden by SDI region. Figure S9 The rates of global ASCVDs burden in different age group during 1990–2019. Figure S10 The rates of IHD burden by age group in different SDI region. Figure S11 The rates of IS burden by age group in different SDI region. Figure S12 The rates of PAD burden by age group in different SDI region. Figure S13 The rate of ASCVDs death in people aged 20–54. Figure S14 The rate of ASCVDs DALYs in people aged 20–54. Figure S15 The rate of ASCVDs death in people older than 55. Figure S16 The rate of ASCVDs DALYs in people older than 55. Figure S17 PAF of risk factor for IHD DALYs for different sex. Figure S18 PAF of risk factor for IS deaths for different sex. Figure S19 PAF of risk factor for IS DALYs. Figure S20 PAF of risk factor for PAD deaths. Figure S21 PAF of risk factor for PAD DALYs. Figure S22 Annual change of risk factor induced IHD death rate. Figure S23 Annual change of risk factor induced IHD DALYs rate. Figure S24 Annual change of risk factor induced IS death rate. Figure S25 Annual change of risk factor induced IS DALYs rate. Figure S26 Annual change of risk factor induced PAD death rate. Figure S27 Annual change of risk factor induced PAD DALYs rate. Figure S28 The proportion of global extremely premature, premature and non-premature ASCVD burden by gender. Figure S29 The proportion of extremely premature, premature and non-premature IHD burden. Figure S30 The proportion of extremely premature, premature and non-premature IS burden. Figure S31 The proportion of extremely premature, premature and non-premature PAD burden. Figure S32 The rates of global extremely premature, premature and non-premature ASCVD burden for male. Figure S33 The rates of global extremely premature, premature and non-premature ASCVD burden for female. Figure S34 The rates of extremely premature IHD, premature IHD and non-premature IHD burden. Figure S35 The rates of extremely premature IS, premature IS and non-premature IS burden. Figure S36 The rates of extremely premature PAD, premature PAD and non-premature PAD burden.

## Data Availability

Relevant data can be retrieved through the available website (https://vizhub.healthdata.org/gbd-results/).

## Abbreviations

ASCVDs: Atherosclerotic cardiovascular disease
IHD: Ischemic heart disease
IS: Ischemic stroke
PAD: Peripheral artery disease
PAF: Population attribution fractions
SDI: Socio-demographic index
DALY: Disability-adjusted life years
