# Supplementary material for: Comparative analysis of atherosclerotic cardiovascular disease burden between ages 20–54 and over 55 years: insights from the Global Burden of Disease Study 2019
Source: BMC Med. 2024 Jul 19;22:303. doi: 10.1186/s12916-024-03527-4 (PMC11264803; doi:10.1186/s12916-024-03527-4)
Supplement: Supplementary file 1 — Additional file 1: Table S1 Definition of risk factors. Table S2 IHD burden in people aged 20–54 across regions. Table S3 IS burden in people aged 20–54 across regions. Table S4 PAD burden in people aged 20–54 across regions. Table S5 IHD burden in people older than 55 + across regions. Table S6 IS burden in people older than 55 + across regions. Table S7 PAD burden in people older than 55 + across regions. Table S8 IHD burden in 204 countries and territories by 2019. Table S9 IS burden in 204 countries and territories by 2019. Table S10 The PAD burden in 204 countries and territories by 2019. Table S11 The temporal trends of ASCVDs burden in people aged 20–54 in 204 countries and territories. Table S12 The temporal trends of ASCVDs burden in people older than 55 in 204 countries and territories. Table S13 IHD burden in people under 40 across SDI regions. Table S14 IS burden in people under 40 across SDI regions. Table S15 IHD burden under 55 for male across SDI regions. Table S16 IS burden under 55 for male across SDI regions. Table S17 PAD burden under 55 for male across SDI regions. Table S18 IHD burden under 65 for female across SDI regions. Table S19 IS burden under 65 for female across SDI regions. Table S20 IHD burden older than 65 for female across SDI regions. Table S21 IHD burden older than 65 for female across SDI regions. Table S22 IS burden older than 65 for female across SDI regions. Table S23 PAD burden older than 65 for female across SDI regions. Table S24 IHD burden older than 55 for male across SDI regions. Table S25 IS burden older than 55 for male across SDI regions. Table S26 PAD burden older than 55 for male across SDI regions. Figure S1 Proportion of people aged 20–54 in total men ASCVDs burden during 1990–2019. Figure S2 Proportion of people aged 20–54 in total women ASCVDs burden during 1990–2019. Figure S3 Association between SDI and age-specific prevalence, death and DALYs rates of IHD in 2019. Figure S4 Association between SDI and age-spe [file 12916_2024_3527_MOESM1_ESM.docx]

**Additional file 1**

**Comparative Analysis of Atherosclerotic Cardiovascular Disease Burden Between Ages 20–54 and Over 55 years: Insights from the Global Burden of Disease Study 2019**

**Contents**

**Tables**

Table S1 Definition of risk factors of atherosclerotic cardiovascular disease.

Table S2 Ischemic heart disease burden in people aged 20-54 across regions during 1990-2019.

Table S3 Ischemic stroke burden in people aged 20-54 across regions during 1990-2019.

Table S4 Peripheral artery disease burden in people aged 20-54 across regions during 1990-2019.

Table S5 Ischemic heart disease burden in people older than 55+ across regions during 1990-2019.

Table S6 Ischemic stroke burden in people older than 55+ across regions during 1990-2019.

Table S7 Peripheral artery disease burden in people older than 55+ across regions during 1990-2019.

Table S8 The ischemic heart disease burden in 204 countries and territories by the year 2019.

Table S9 The ischemic stroke in 204 countries and territories by the year 2019.

Table S10 The peripheral artery disease burden in 204 countries and territories by the year 2019.

Table S11 The temporal trends of ASCVDs burden in people aged 20-54 in 204 countries and territories.

Table S12 The temporal trends of ASCVDs burden in people older than 55 in 204 countries and territories.

Table S13 Ischemic heart disease burden in people under 40 across SDI regions during 1990-2019.

Table S14 Ischemic stroke burden in people under 40 across SDI regions during 1990-2019.

Table S15 Ischemic heart disease burden under 55 for male across SDI regions during 1990-2019.

Table S16 Ischemic stroke burden under 55 for male across SDI regions during 1990-2019.

Table S17 Peripheral artery disease burden under 55 for male across SDI regions during 1990-2019.

Table S18 Ischemic heart disease burden under 65 for female across SDI regions during 1990-2019.

Table S19 Ischemic stroke burden under 65 for female across SDI regions during 1990-2019.

Table S20 Ischemic heart disease burden older than 65 for female across SDI regions during 1990-2019.

Table S21 Ischemic heart disease burden older than 65 for female across SDI regions during 1990-2019.

Table S22 Ischemic stroke burden older than 65 for female across SDI regions during 1990-2019.

Table S23 Peripheral artery disease burden older than 65 for female across SDI regions during 1990-2019.

Table S24 Ischemic heart disease burden older than 55 for male across SDI regions during 1990-2019.

Table S25 Ischemic stroke burden older than 55 for male across SDI regions during 1990-2019.

Table S26 Peripheral artery disease burden older than 55 for male across SDI regions during 1990-2019.

**Figures**

Figure S1 Proportion of people aged 20-54 in total men ASCVDs burden during 1990-2019.

Figure S2 Proportion of people aged 20-54 in total women ASCVDs burden during 1990-2019.

Figure S3 Association between SDI and age-specific prevalence, death and DALYs rates of IHD in 2019.

Figure S4 Association between SDI and age-specific prevalence, death and DALYs rates of IS in 2019.

Figure S5 Association between SDI and age-specific prevalence, death and DALYs rates of PAD in 2019.

Figure S6 The change of IS burden by countries and territories during 1990-2019.

Figure S7 The change of PAD burden by countries and territories during 1990-2019.

Figure S8 The men to women ratio of the rate of ASCVDs burden by SDI region during 1990-2019.

Figure S9 The rates of global ASCVDs burden in different age group during 1990-2019.

Figure S10 The rates of IHD burden by age group in different SDI region during 1990-2019.

Figure S11 The rates of IS burden by age group in different SDI region during 1990-2019.

Figure S12 The rates of PAD burden by age group in different SDI region during 1990-2019.

Figure S13 The rate of ASCVDs death in people aged 20-54 during 1990-2019.

Figure S14 The rate of ASCVDs DALYs in people aged 20-54 during 1990-2019.

Figure S15 The rate of ASCVDs death in people older than 55 during 1990-2019.

Figure S16 The rate of ASCVDs DALYs in people older than 55 during 1990-2019.

Figure S17 PAF of risk factor for IHD DALYs for different sex.

Figure S18 PAF of risk factor for IS deaths for different sex.

Figure S19 PAF of risk factor for IS DALYs for different sex.

Figure S20 PAF of risk factor for PAD deaths for different sex.

Figure S21 PAF of risk factor for PAD DALYs for different sex.

Figure S22 Annual change of risk factor induced IHD death rate for different sex during 1990-2019.

Figure S23 Annual change of risk factor induced IHD DALYs rate for different sex during 1990-2019.

Figure S24 Annual change of risk factor induced IS death rate for different sex during 1990-2019.

Figure S25 Annual change of risk factor induced IS DALYs rate for different sex during 1990-2019.

Figure S26 Annual change of risk factor induced PAD death rate for different sex during 1990-2019.

Figure S27 Annual change of risk factor induced PAD DALYs rate for different sex during 1990-2019.

Figure S28 The proportion of global extremely premature, premature and non-premature ASCVD burden by gender.

Figure S29 The proportion of extremely premature, premature and non-premature IHD burden by gender and SDI.

Figure S30 The proportion of extremely premature, premature and non-premature IS burden by gender and SDI.

Figure S31 The proportion of extremely premature, premature and non-premature PAD burden by gender and SDI.

Figure S32 The rates of global extremely premature, premature and non-premature ASCVD burden for male during 1990 and 2019.

Figure S33 The rates of global extremely premature, premature and non-premature ASCVD burden for female during 1990 and 2019.

Figure S34 The rates of extremely premature IHD, premature IHD and non-premature IHD burden by SDI and gender during 1990 and 2019.

Figure S35 The rates of extremely premature IS, premature IS and non-premature IS burden by SDI and gender during 1990 and 2019.

Figure S36 The rates of extremely premature PAD, premature PAD and non-premature PAD burden by SDI and gender during 1990 and 2019.

Table S1 Definition of risk factors of atherosclerotic cardiovascular disease.

| Risk factor | Definition |
| --- | --- |
| **Environment risks** |  |
| High temperature | High temperature (heat) exposure is defined as exposure to temperatures warmer than this TMREL. The TMREL was defined as the temperature associated with the lowest mortality for all included causes. A death-weighted average of the cause specific exposure-response curves was calculated with the minimum of this average curve being the TMREL. |
| low temperature | Low temperature (cold) is defined as temperatures colder than this TMREL. The TMREL was defined as the temperature associated with the lowest mortality for all included causes. A death-weighted average of the cause specific exposure-response curves was calculated with the minimum of this average curve being the TMREL. |
| Ambient particulate matter pollution | Exposure to ambient particulate matter pollution is defined as the population-weighted annual average mass concentration of particles with an aerodynamic diameter less than 2.5 micrometers (PM2.5) in a cubic meter of air. |
| Household air pollution from solid fuels | Exposure to household pollution is defined as the population-weighted annual average mass concentration of particles with an aerodynamic diameter less than 2.5 micrometers (PM2.5) in a cubic meter of air. Exposure to household air pollution from solid fuels (HAP) is estimated from both the proportion of individuals using solid cooking fuels and the level of PM2.5 air pollution exposure for these individuals. Solid fuels in our analysis include coal, wood, charcoal, dung, and agricultural residues. |
| Lead exposure | The TMREL was estimated at 2.0 µg/dL. |
| **Behavior risk** |  |
| Alcohol use | We defined exposure as the grams per day of pure alcohol consumed among current drinkers. We constructed this exposure using the indicators outlined below:1. Current drinkers, defined as the proportion of individuals who have consumed at least one alcoholic beverage (or some approximation) in a 12-month period. 2. Alcohol consumption (in grams per day), defined as grams of alcohol consumed by current drinkers, per day, over a 12-month period. 3. Alcohol liters per capita stock, defined in liters per capita of pure alcohol, over a 12-month period. |
| Smoking | The theoretical minimum-risk exposure level is 0. |
| Secondhand smoke | We define secondhand smoke exposure as current exposure to secondhand tobacco smoke at home, at work, or in other public places. The theoretical minimum-risk exposure level for secondhand smoke is zero exposure among nonsmokers, meaning that non-smokers would not live with any primary smokers. |
| Low physical activity | The theoretical minimum-risk exposure level for physical inactivity is 3000-4500 MET-min per week, which was calculated as the exposure at which minimal deaths across outcomes occurred. |
| Diet low in fruit | Average daily consumption (in grams per day) of less than 310-340 grams of fruit including fresh, frozen, cooked, canned, or dried fruit, excluding fruit juices and salted or pickled fruits. |
| Diet low in vegetables | Average daily consumption (in grams per day) of less than 280-320 grams of vegetables, including fresh, frozen, cooked, canned, or dried vegetables and excluding legumes and salted or pickled vegetables, juices, nuts and seeds, and starchy vegetables such as potatoes or corn. |
| Diet low in whole grains | Average daily consumption (in grams per day) of less than 140-160 grams of whole grains (bran, germ, and endosperm in their natural proportion) from breakfast cereals, bread, rice, pasta, biscuits, muffins, tortillas, pancakes, and other sources. |
| Diet low in nuts and seeds | Average daily consumption (in grams per day) of less than 10-19 grams of nuts and seeds, including tree nuts and seeds and peanuts. |
| Diet low in fiber | Average daily consumption (in grams per day) of less than 21-22 grams of fiber from all sources including fruits, vegetables, grains, legumes, and pulses. |
| Diet low in omega-3 fatty acids | Average daily consumption (in milligrams per day) of less than 430-470 milligrams of eicosatetraenoic acid (EPA) and docosahexaenoic acid (DHA). |
| Diet low in polyunsaturated fatty acids (PUFA) | Average daily consumption (in % daily energy) of less than 7-9% total energy intake from polyunsaturated fatty acids. |
| Diet low in calcium | Average daily consumption (in grams per day) of less than 1.06-1.1 grams of calcium from all sources, including milk, yogurt, and cheese. |
| Diet low in legumes | Average daily consumption (in grams per day) of less than of 90-100 grams of legumes and pulses, including fresh, frozen, cooked, canned, or dried legumes. |
| Diet high in red meat | Any intake (in grams per day) of red meat including beef, pork, lamb, and goat but excluding poultry, fish, eggs, and all processed meats. |
| Diet high in processed meat | Any intake (in grams per day) of meat preserved by smoking, curing, salting, or addition of chemical preservatives. |
| Diet high in sugar sweetened beverages (SSBs) | Any intake (in grams per day) of beverages with ≥50 kcal per 226.8 gram serving, including carbonated beverages, sodas, energy drinks, fruit drinks, but excluding 100% fruit and vegetable juices. |
| Diet high in trans fatty acids | Any intake (in percent daily energy) of trans fat from all sources, mainly partially hydrogenated vegetable oils and ruminant products. |
| Diet high in sodium | Average 24-hour urinary sodium excretion (in grams per day) greater than 1-5 grams. |
| **Metabolic risks** |  |
| High body-mass index | High body-mass index (BMI) for adults (ages 20+) is defined as BMI greater than 20 to 25 kg/m2. |
| High LDL cholesterol | The TMREL was estimated between 0.7 and 1.3 mmol/L. |
| High systolic blood pressure | The TMREL of SBP ranges from 110 to 115 mmHg. |
| Kidney dysfunction | The theoretical minimum-risk exposure level is ACR 30 mg/g or less and eGFR greater than 60ml/min/1.73m2. |
| High fasting plasma glucose | High fasting plasma glucose (FPG) is measured as the mean FPG in a population, where FPG is a continuous exposure in units of mmol/L. Since FPG is along a continuum, we define high FPG as any level above the TMREL, which is 4.8-5.4 mmol/L. |

TMREL: theoretical minimum exposure level.

Table S2 Ischemic heart disease burden in people aged 20-54 across regions during 1990-2019.

|  | Prevalence | | | | | Deaths | | | | | DALY | | | | |
| --- | --- | --- | --- | --- | --- | --- | --- | --- | --- | --- | --- | --- | --- | --- | --- |
|  | 1990 | | 2019 | | 1990-2019 | 1990 | | 2019 | | 1990-2019 | 1990 | | 2019 | | 1990-2019 |
| location | Number | Rate | Number | Rate | EAPC | Number | Rate | Number | Rate | EAPC | Number | Rate | Number | Rate | EAPC |
| Global | 16709672.86 (14525090.02-19384363.51) | 694.74 (603.91-805.94) | 31425971.75 (27282410.57-36562206.12) | 837.49 (727.07-974.37) | 0.69 (0.65-0.74) | 699173.66 (664576.3-736633.54) | 29.07 (27.63-30.63) | 1012293.98 (930430.05-1096671.72) | 26.98 (24.8-29.23) | -0.26 (-0.34--0.19) | 31264210.29 (29653010.16-32961395.72) | 1299.87 (1232.88-1370.43) | 45036980.45 (41431568.43-48767394.08) | 1200.22 (1104.14-1299.64) | -0.31 (-0.38--0.23) |
| SDI region |  |  |  |  |  |  |  |  |  |  |  |  |  |  |  |
| High SDI | 3078278.32 (2712076.22-3511658.3) | 746.74 (657.9-851.87) | 3816524.88 (3421162.44-4265199.28) | 793.23 (711.05-886.48) | 0.14 (0.1-0.18) | 96441.25 (95038.46-98398.48) | 23.39 (23.05-23.87) | 72258.78 (68017.59-77835.71) | 15.02 (14.14-16.18) | -1.57 (-1.65--1.48) | 4164145.5 (4091054.33-4248798.87) | 1010.15 (992.42-1030.69) | 3120327.17 (2942216.92-3364916.89) | 648.53 (611.51-699.36) | -1.57 (-1.65--1.49) |
| High-middle SDI | 4399717.3 (3796774.12-5151370.4) | 785.63 (677.97-919.85) | 7195813.08 (6122069.88-8501547.54) | 962.31 (818.72-1136.93) | 0.73 (0.64-0.82) | 186497.22 (175376.63-194528.27) | 33.3 (31.32-34.74) | 183745.33 (168535.49-199240.41) | 24.57 (22.54-26.64) | -1.47 (-1.83--1.11) | 8222432.77 (7719684.09-8599657.27) | 1468.23 (1378.46-1535.59) | 8135240.52 (7492725.6-8818896.54) | 1087.94 (1002.02-1179.37) | -1.49 (-1.83--1.15) |
| Middle SDI | 5243999.3 (4497285.76-6135854.45) | 674.33 (578.31-789.02) | 11447051.02 (9837966.08-13445182.49) | 929.69 (799-1091.97) | 1.20  (1.14-1.27) | 202853.19 (191093.54-215689.89) | 26.09 (24.57-27.74) | 351002.39 (318225.02-382577.01) | 28.51 (25.85-31.07) | 0.51 (0.45-0.58) | 9297026.97 (8743185.9-9905832.5) | 1195.52 (1124.3-1273.81) | 15681426.85 (14247669.71-17107228.01) | 1273.59 (1157.14-1389.38) | 0.39 (0.33-0.45) |
| Low-middle SDI | 2892723.31 (2516290.12-3351306.56) | 628.77 (546.95-728.45) | 6291741.61 (5466460.17-7311649.54) | 747.84 (649.75-869.07) | 0.67 (0.6-0.75) | 156697.37 (142709.98-171445.37) | 34.06 (31.02-37.27) | 291921.5 (260204.27-326101.84) | 34.7 (30.93-38.76) | 0.27 (0.16-0.38) | 7079577.41 (6442957.19-7743439.52) | 1538.84 (1400.46-1683.14) | 13033072.45 (11634975.11-14517640.38) | 1549.12 (1382.94-1725.57) | 0.19 (0.1-0.29) |
| Low SDI | 1084806.54 (949913.27-1249563.27) | 559.28 (489.74-644.22) | 2654035.85 (2322101.68-3051198.63) | 591.43 (517.47-679.94) | 0.21 (0.14-0.29) | 56275.94 (49756.04-64264.66) | 29.01 (25.65-33.13) | 112673.05 (98907.65-129003.88) | 25.11 (22.04-28.75) | -0.53 (-0.57--0.5) | 2482889.61 (2194709.54-2838641.35) | 1280.08 (1131.5-1463.49) | 5036351.68 (4424042.19-5768534.81) | 1122.32 (985.87-1285.48) | -0.49 (-0.54--0.43) |
| Sub-region |  |  |  |  |  |  |  |  |  |  |  |  |  |  |  |
| Andean Latin America | 44128.56 (36537.25-54299.29) | 281.41 (233-346.27) | 105046.44 (86878.75-130025.82) | 344.02 (284.52-425.83) | 0.73 (0.68-0.77) | 2600.67 (2272.11-2948.7) | 16.58 (14.49-18.8) | 3233.18 (2534.79-4111.81) | 10.59 (8.3-13.47) | -1.5 (-1.71--1.29) | 121439.99 (106236.2-136903.81) | 774.44 (677.48-873.05) | 150123.11 (119657.52-189865.5) | 491.64 (391.87-621.8) | -1.53 (-1.74--1.33) |
| Australasia | 104188.32 (93442.81-114948.78) | 1035.34 (928.56-1142.27) | 149776.08 (135012.19-164441.73) | 1099.35 (990.98-1206.99) | 0.19 (0.01-0.36) | 2182.42 (2126.62-2240.51) | 21.69 (21.13-22.26) | 1300.06 (1231.13-1368.83) | 9.54 (9.04-10.05) | -2.79 (-2.88--2.71) | 93516.59 (91042.9-96086.98) | 929.3 (904.71-954.84) | 56188.29 (53334.95-59173.71) | 412.42 (391.48-434.33) | -2.78 (-2.86--2.69) |
| Caribbean | 158476.24 (141345.07-177856.57) | 998.88 (890.91-1121.04) | 279924.44 (249131.26-314668.06) | 1227.79 (1092.73-1380.18) | 0.82 (0.77-0.86) | 4925.86 (4615.23-5271.42) | 31.05 (29.09-33.23) | 6634.5 (5364.32-8044.23) | 29.1 (23.53-35.28) | -0.09 (-0.33-0.15) | 217512.46 (203921.37-232166.81) | 1370.99 (1285.33-1463.36) | 289758.4 (234616.89-350267.46) | 1270.92 (1029.06-1536.32) | -0.16 (-0.4-0.09) |
| Central Asia | 268389.89 (238568.61-303492.87) | 902.12 (801.88-1020.11) | 465753.55 (414095.84-524709.69) | 1000.98 (889.96-1127.69) | 0.8 (0.6-0.99) | 14703.01 (14233.97-15140.56) | 49.42 (47.84-50.89) | 22188.7 (19666.14-25211.68) | 47.69 (42.27-54.18) | -0.47 (-0.94--0.01) | 639902.58 (619673.62-659253.69) | 2150.85 (2082.86-2215.89) | 967402.08 (860535.05-1095214.41) | 2079.11 (1849.44-2353.8) | -0.58 (-1.06--0.1) |
| Central Europe | 568127.12 (482874.14-681986.57) | 974.93 (828.63-1170.32) | 537101.54 (464372.81-640434.02) | 986.71 (853.1-1176.55) | -0.12 (-0.26-0.03) | 30054.27 (29557.98-30531.88) | 51.57 (50.72-52.39) | 13336.2 (11273.51-15473.02) | 24.5 (20.71-28.43) | -3.25 (-3.49--3.01) | 1301295.83 (1279537.72-1322378.56) | 2233.08 (2195.74-2269.26) | 568979.23 (484055.39-657465.97) | 1045.28 (889.26-1207.84) | -3.31 (-3.54--3.09) |
| Central Latin America | 339621.96 (295870.65-389961.92) | 497.9 (433.76-571.7) | 742981.88 (647401.13-860529.08) | 605.98 (528.02-701.85) | 0.72 (0.69-0.76) | 12475.03 (12220.33-12751.08) | 18.29 (17.92-18.69) | 21353.61 (17989.87-25374.49) | 17.42 (14.67-20.7) | -0.17 (-0.36-0.01) | 569167.27 (557255.77-582286.66) | 834.42 (816.96-853.65) | 954228.34 (812307.55-1125326.81) | 778.27 (662.52-917.82) | -0.24 (-0.45--0.04) |
| Central Sub-Saharan Africa | 87582.62 (77222.34-99201.43) | 433.45 (382.17-490.95) | 229935.01 (203579.98-260382.22) | 443.65 (392.8-502.4) | 0.14 (0.11-0.16) | 4068.95 (3204.64-5167.47) | 20.14 (15.86-25.57) | 8647.99 (6300.29-11814.41) | 16.69 (12.16-22.8) | -0.64 (-0.7--0.58) | 178021.77 (140483.3-224657.59) | 881.03 (695.25-1111.83) | 380297.19 (279969.18-516406.09) | 733.77 (540.19-996.39) | -0.64 (-0.7--0.58) |
| East Asia | 4472538.95 (3672169.96-5416982.26) | 733.52 (602.26-888.41) | 9041319.38 (7435816.86-11108547.11) | 1134.3 (932.88-1393.65) | 1.58 (1.43-1.72) | 102464.3 (86572.29-118736.35) | 16.8 (14.2-19.47) | 157012.78 (130180.61-187885.88) | 19.7 (16.33-23.57) | 0.82 (0.74-0.91) | 4840665.89 (4111914.51-5558833) | 793.9 (674.38-911.68) | 7155906.8 (5966004.27-8443760.85) | 897.76 (748.48-1059.33) | 0.65 (0.58-0.72) |
| Eastern Europe | 1072354.52 (936268.95-1230235.51) | 972.14 (848.77-1115.27) | 1071063.15 (934538.27-1232468.47) | 1064.16 (928.51-1224.52) | 0.46 (0.29-0.63) | 64810.31 (58661.44-67632.85) | 58.75 (53.18-61.31) | 54226.71 (47132.03-62288.47) | 53.88 (46.83-61.89) | -1.11 (-1.95--0.26) | 2754599.9 (2491253.35-2877944.99) | 2497.17 (2258.44-2608.99) | 2332727.97 (2035620.49-2678976.64) | 2317.69 (2022.5-2661.71) | -1.17 (-2--0.33) |
| Eastern Sub-Saharan Africa | 304648.81 (263449.31-356956.61) | 452.19 (391.04-529.83) | 797380.02 (688624.04-935444.52) | 490.78 (423.84-575.76) | 0.27 (0.24-0.29) | 10889.4 (9012.08-13212.78) | 16.16 (13.38-19.61) | 20410.74 (16229.9-25588.63) | 12.56 (9.99-15.75) | -1.11 (-1.18--1.04) | 488675.36 (404465.26-594545.27) | 725.34 (600.34-882.48) | 935025.23 (748069.18-1166512.31) | 575.5 (460.43-717.98) | -1.03 (-1.11--0.96) |
| High-income Asia Pacific | 435156.34 (382644.81-497871.66) | 493.71 (434.13-564.86) | 439444.2 (387970.08-496972.91) | 512.34 (452.32-579.41) | -0.02 (-0.14-0.1) | 12363.17 (11774.42-12765.49) | 14.03 (13.36-14.48) | 6220.76 (5924.32-6564) | 7.25 (6.91-7.65) | -1.99 (-2.17--1.8) | 552827.17 (522480.21-571631.51) | 627.21 (592.78-648.55) | 272180.14 (259798.43-287345.87) | 317.33 (302.89-335.01) | -2.05 (-2.24--1.86) |
| High-income North America | 1083945.62 (946537.99-1252124.71) | 766.85 (669.64-885.83) | 1213164.03 (1064857.3-1378919.39) | 729.08 (639.95-828.7) | -0.27 (-0.38--0.16) | 37406.01 (36795.09-38353.75) | 26.46 (26.03-27.13) | 31540.6 (30332.65-33514.01) | 18.96 (18.23-20.14) | -1.19 (-1.39--0.99) | 1606983.88 (1578127.84-1647913.87) | 1136.88 (1116.47-1165.84) | 1336835.9 (1286518.43-1420677.33) | 803.41 (773.17-853.79) | -1.24 (-1.43--1.05) |
| North Africa and Middle East | 1417440.29 (1272016.72-1585362.62) | 1041.73 (934.85-1165.14) | 3836622.13 (3436780.09-4295787.91) | 1243.37 (1113.79-1392.18) | 0.62 (0.56-0.69) | 76753.89 (70121.03-85063.27) | 56.41 (51.53-62.52) | 122726.49 (102327.76-148063.71) | 39.77 (33.16-47.98) | -1.45 (-1.58--1.32) | 3413893.87 (3118731.37-3781753.57) | 2508.99 (2292.07-2779.35) | 5413789.99 (4525492.87-6517668.81) | 1754.5 (1466.62-2112.24) | -1.48 (-1.61--1.36) |
| Oceania | 18875.22 (15750.71-22844.95) | 705.42 (588.65-853.78) | 49058.58 (40506.25-60035.47) | 813.22 (671.46-995.18) | 0.58 (0.54-0.63) | 1466.14 (1167.28-1899.68) | 54.79 (43.62-71) | 3747.81 (2884.52-4949.53) | 62.13 (47.82-82.05) | 0.61 (0.53-0.7) | 64990.11 (51681.58-84077.42) | 2428.87 (1931.49-3142.21) | 165528.76 (126360.91-218220.61) | 2743.9 (2094.63-3617.35) | 0.59 (0.5-0.68) |
| South Asia | 2941008.75 (2565402.78-3397244.21) | 644.55 (562.23-744.54) | 6603239.17 (5760232.46-7618158.14) | 753.91 (657.66-869.78) | 0.54 (0.42-0.66) | 187128.37 (168345.52-206742.87) | 41.01 (36.89-45.31) | 362783.11 (318275.64-413678.06) | 41.42 (36.34-47.23) | 0.22 (0.1-0.34) | 8447749.21 (7589583.65-9335899.22) | 1851.4 (1663.32-2046.04) | 16218471.63 (14248327.34-18448547.31) | 1851.7 (1626.77-2106.31) | 0.16 (0.04-0.28) |
| Southeast Asia | 1021670.15 (877990.44-1197590.51) | 502.88 (432.16-589.47) | 2286970.1 (1954319.22-2703066.03) | 665.45 (568.66-786.52) | 1.15 (1.09-1.22) | 50065.24 (45064.38-56086.91) | 24.64 (22.18-27.61) | 99938.16 (87238.11-114667.35) | 29.08 (25.38-33.37) | 0.86 (0.74-0.98) | 2288382.08 (2061844.96-2563374.84) | 1126.37 (1014.86-1261.72) | 4471381.74 (3914971.32-5116645.24) | 1301.06 (1139.15-1488.81) | 0.75 (0.64-0.87) |
| Southern Latin America | 82918.3 (73422.69-93722.33) | 372.65 (329.97-421.2) | 121187.69 (108372.26-135611.11) | 371.27 (332.01-415.46) | 0.02 (-0.01-0.05) | 5439.78 (5232.98-5643.13) | 24.45 (23.52-25.36) | 4073.26 (3838.27-4300.12) | 12.48 (11.76-13.17) | -2.41 (-2.54--2.29) | 234757.32 (226584.44-243179.99) | 1055.04 (1018.31-1092.89) | 175032.43 (164802.82-184911.38) | 536.23 (504.89-566.5) | -2.44 (-2.58--2.29) |
| Southern Sub-Saharan Africa | 118043.41 (102870.5-136614.63) | 541.88 (472.23-627.13) | 233757.17 (203428.52-270808.85) | 606.7 (527.98-702.86) | 0.21 (0.09-0.32) | 3912.47 (3533.47-4327.99) | 17.96 (16.22-19.87) | 5736.42 (4994.66-6609.02) | 14.89 (12.96-17.15) | -0.8 (-1.25--0.34) | 177134.45 (160455.04-195982.65) | 813.14 (736.57-899.66) | 254343.95 (222318.28-291739.5) | 660.13 (577.01-757.19) | -0.92 (-1.4--0.44) |
| Tropical Latin America | 336123.01 (278385.09-410611.11) | 493.35 (408.61-602.69) | 693796.23 (573766.92-855336.42) | 602.85 (498.55-743.21) | 0.68 (0.65-0.71) | 20922.07 (20337.61-21583.24) | 30.71 (29.85-31.68) | 22559.48 (21556.6-23650.57) | 19.6 (18.73-20.55) | -1.41 (-1.48--1.35) | 926024.79 (899875.42-955097.42) | 1359.2 (1320.82-1401.87) | 990666.84 (945739.03-1037801.8) | 860.8 (821.76-901.76) | -1.45 (-1.52--1.38) |
| Western Europe | 1505808.79 (1328438.48-1712181.54) | 796.76 (702.91-905.96) | 1647865.32 (1474640.08-1835212.07) | 826.14 (739.29-920.06) | 0.14 (0.06-0.21) | 43169.8 (42528.17-43954.54) | 22.84 (22.5-23.26) | 20870.53 (20187.63-21770.59) | 10.46 (10.12-10.91) | -2.79 (-2.88--2.71) | 1850416.3 (1819909.31-1886344.64) | 979.1 (962.96-998.12) | 891911.68 (861423.77-931655.71) | 447.15 (431.86-467.07) | -2.82 (-2.91--2.73) |
| Western Sub-Saharan Africa | 328625.99 (287253.63-379795.53) | 463.7 (405.32-535.9) | 880585.65 (768633.5-1022094.35) | 495.29 (432.32-574.88) | 0.29 (0.26-0.32) | 11372.49 (8947.43-14821.05) | 16.05 (12.63-20.91) | 23752.87 (18623.33-30255.2) | 13.36 (10.47-17.02) | -0.75 (-0.88--0.62) | 496253.47 (391179.79-644888.05) | 700.23 (551.97-909.96) | 1056200.75 (828560.57-1341602.38) | 594.07 (466.03-754.59) | -0.69 (-0.83--0.55) |

EAPCs: Estimated annual percentage changes; SDI: socio-demographic index; DALYs: disability-adjusted life years.

Table S3 Ischemic stroke burden in people aged 20-54 across regions during 1990-2019.

|  | Prevalence | | | | | Deaths | | | | | DALY | | | | |
| --- | --- | --- | --- | --- | --- | --- | --- | --- | --- | --- | --- | --- | --- | --- | --- |
|  | 1990 | | 2019 | | 1990-2019 | 1990 | | 2019 | | 1990-2019 | 1990 | | 2019 | | 1990-2019 |
| location | Number | Rate | Number | Rate | EAPC | Number | Rate | Number | Rate | EAPC | Number | Rate | Number | Rate | EAPC |
| Global | 10570295.96 (8873463.53-12464773.45) | 439.48 (368.93-518.25) | 18387960.54 (15440997.84-21640123.44) | 490.03 (411.5-576.7) | 0.45 (0.36-0.54) | 71685.61 (65514.74-82469.73) | 2.98 (2.72-3.43) | 102259.72 (91010.54-114078.59) | 2.73 (2.43-3.04) | -0.35 (-0.49--0.21) | 4647890.38 (4050766.46-5345120.73) | 193.24 (168.42-222.23) | 7048856.85 (6060573.82-8108032.88) | 187.85 (161.51-216.08) | -0.12 (-0.21--0.03) |
| SDI region |  |  |  |  |  |  |  |  |  |  |  |  |  |  |  |
| High SDI | 1775844.62 (1489221.24-2122424.53) | 430.79 (361.26-514.86) | 2443098.23 (2034481.72-2918834.05) | 507.77 (422.85-606.65) | 0.89 (0.73-1.04) | 6929.85 (6574.77-7402.37) | 1.68 (1.59-1.8) | 5219.19 (4594.34-5901.8) | 1.08 (0.95-1.23) | -1.72 (-1.87--1.57) | 541807.55 (463443.31-626888.8) | 131.43 (112.42-152.07) | 560096.01 (444883.11-676988.42) | 116.41 (92.46-140.71) | -0.34 (-0.43--0.24) |
| High-middle SDI | 2756793.35 (2301531.16-3294059.41) | 492.26 (410.97-588.2) | 3746097.58 (3099693.15-4491893.99) | 500.97 (414.53-600.71) | 0.05 (-0.05-0.14) | 25433.29 (23434.42-27604.71) | 4.54 (4.18-4.93) | 24222.35 (21549.88-26938.15) | 3.24 (2.88-3.6) | -1.52 (-1.91--1.13) | 1515901.8 (1355025.56-1704083.13) | 270.69 (241.96-304.29) | 1622401.74 (1397727-1863444.04) | 216.97 (186.92-249.2) | -1.06 (-1.32--0.8) |
| Middle SDI | 3430846.38 (2873884.07-4060643.16) | 441.18 (369.56-522.16) | 6480628.53 (5423074.97-7699922.42) | 526.33 (440.44-625.36) | 0.63 (0.52-0.74) | 24434.47 (21947.4-29286.93) | 3.14 (2.82-3.77) | 42330.76 (36789.07-48004.33) | 3.44 (2.99-3.9) | 0.48 (0.37-0.58) | 1595772.42 (1379424.44-1843305.71) | 205.2 (177.38-237.03) | 2787986.02 (2372546.18-3227575.42) | 226.43 (192.69-262.13) | 0.44 (0.36-0.52) |
| Low-middle SDI | 1767154.91 (1480657.97-2083953.29) | 384.12 (321.84-452.98) | 3706695.24 (3122085.09-4347851.44) | 440.58 (371.09-516.79) | 0.55 (0.44-0.67) | 10875.58 (8980.66-13787.44) | 2.36 (1.95-3) | 21226.72 (17886.95-25531.64) | 2.52 (2.13-3.03) | 0.41 (0.34-0.48) | 712229.1 (597258.65-863219.9) | 154.81 (129.82-187.63) | 1411897 (1193172.28-1655695.11) | 167.82 (141.82-196.8) | 0.42 (0.35-0.48) |
| Low SDI | 833457.44 (712424.04-962977.17) | 429.7 (367.3-496.47) | 1999438.99 (1706487.01-2324947.89) | 445.56 (380.28-518.1) | 0.18 (0.08-0.27) | 3978.31 (3099.1-5371.79) | 2.05 (1.6-2.77) | 9193.23 (7434.95-12294.64) | 2.05 (1.66-2.74) | 0.01 (-0.05-0.06) | 279921.39 (229617.86-350797.42) | 144.32 (118.38-180.86) | 662036.56 (551777.25-812792.04) | 147.53 (122.96-181.13) | 0.11 (0.05-0.16) |
| Sub-region |  |  |  |  |  |  |  |  |  |  |  |  |  |  |  |
| Andean Latin America | 66531.6 (58161.57-76213.25) | 424.28 (370.9-486.02) | 132053.46 (114471.38-152350.87) | 432.47 (374.89-498.94) | 0.06 (0.02-0.11) | 268.64 (214.48-324.9) | 1.71 (1.37-2.07) | 313.81 (243.91-401.76) | 1.03 (0.8-1.32) | -2.05 (-2.24--1.85) | 17935.03 (14997.12-21050.87) | 114.37 (95.64-134.24) | 24917.71 (20331.37-29947.66) | 81.6 (66.58-98.08) | -1.36 (-1.51--1.22) |
| Australasia | 35118.73 (29831.32-40994.33) | 348.98 (296.44-407.37) | 47506.11 (40295.92-56000.31) | 348.69 (295.77-411.04) | 0.16 (0.09-0.23) | 78.95 (72.94-85.87) | 0.78 (0.72-0.85) | 40.52 (34.41-46.65) | 0.3 (0.25-0.34) | -3.4 (-3.56--3.23) | 8122.73 (6621.71-9748.2) | 80.72 (65.8-96.87) | 8106.61 (6104.2-10253.54) | 59.5 (44.8-75.26) | -0.97 (-1.07--0.86) |
| Caribbean | 89941.02 (79247.5-101926.07) | 566.9 (499.5-642.45) | 143957.48 (126310.53-164921.56) | 631.42 (554.02-723.37) | 0.52 (0.46-0.57) | 461.28 (406.24-553.86) | 2.91 (2.56-3.49) | 681.67 (540.92-864.12) | 2.99 (2.37-3.79) | 0.38 (0.15-0.61) | 27603.73 (24428.44-31993) | 173.99 (153.97-201.65) | 41027.17 (33499.43-49904.93) | 179.95 (146.93-218.89) | 0.34 (0.17-0.51) |
| Central Asia | 148062.46 (130754.39-167106.1) | 497.67 (439.49-561.68) | 226681.45 (193537.52-267145.34) | 487.18 (415.95-574.14) | 0.11 (-0.01-0.22) | 1662.3 (1435.38-1967.91) | 5.59 (4.82-6.61) | 2366.66 (2054.04-2723.6) | 5.09 (4.41-5.85) | -0.53 (-1.02--0.03) | 95781.34 (83075.11-109693) | 321.94 (279.23-368.7) | 138362.67 (119762.93-159439.28) | 297.37 (257.39-342.66) | -0.47 (-0.86--0.07) |
| Central Europe | 336676.99 (286599.38-396040.23) | 577.75 (491.82-679.62) | 285115.21 (238686.23-340416.73) | 523.79 (438.49-625.38) | -0.39 (-0.46--0.32) | 3440.87 (3242.04-3714.94) | 5.9 (5.56-6.38) | 1699.34 (1429.95-2032.81) | 3.12 (2.63-3.73) | -2.6 (-2.89--2.32) | 202094.85 (182143.88-223224.86) | 346.8 (312.57-383.06) | 119379.52 (99897.53-140588.41) | 219.31 (183.52-258.28) | -1.9 (-2.07--1.73) |
| Central Latin America | 346868.92 (290092.99-408393.79) | 508.52 (425.29-598.72) | 611889.45 (510948.72-722174.22) | 499.06 (416.73-589.01) | -0.09 (-0.21-0.03) | 1122.08 (1057.88-1214.69) | 1.65 (1.55-1.78) | 1093.52 (897.99-1295.63) | 0.89 (0.73-1.06) | -2.21 (-2.57--1.86) | 79522.71 (70419.18-90067.63) | 116.58 (103.24-132.04) | 97868.67 (80814.93-117342.74) | 79.82 (65.91-95.71) | -1.37 (-1.63--1.1) |
| Central Sub-Saharan Africa | 88051.87 (76869.38-99795.25) | 435.77 (380.43-493.89) | 214955.68 (185657.92-245791.63) | 414.75 (358.22-474.25) | -0.14 (-0.21--0.08) | 398.17 (292.56-569.88) | 1.97 (1.45-2.82) | 835.57 (586.85-1216.66) | 1.61 (1.13-2.35) | -0.66 (-0.7--0.61) | 28152.37 (22553.05-36486.61) | 139.33 (111.62-180.57) | 63025.57 (49295.54-80199.06) | 121.61 (95.11-154.74) | -0.45 (-0.49--0.42) |
| East Asia | 2474760.44 (2037575.69-2985542.43) | 405.87 (334.17-489.64) | 4127134.86 (3379631.25-5009483.57) | 517.78 (424-628.48) | 0.74 (0.55-0.92) | 23049.84 (19448.99-27698.9) | 3.78 (3.19-4.54) | 34070.52 (27659.36-40642.57) | 4.27 (3.47-5.1) | 0.36 (0.14-0.58) | 1439746.36 (1226578.34-1693985.59) | 236.13 (201.17-277.82) | 2164874.27 (1795110.49-2554831.07) | 271.6 (225.21-320.52) | 0.38 (0.21-0.56) |
| Eastern Europe | 754477.87 (621756.8-923307.43) | 683.97 (563.65-837.02) | 658645.88 (537399.33-798833.27) | 654.4 (533.94-793.68) | 0.01 (-0.14-0.16) | 9418.77 (8386.89-10378.89) | 8.54 (7.6-9.41) | 6835.45 (5921.45-7782.76) | 6.79 (5.88-7.73) | -1.44 (-2.21--0.66) | 511245.13 (453715.22-573896.52) | 463.47 (411.31-520.26) | 398476.89 (343352.93-455060.9) | 395.91 (341.14-452.13) | -1.09 (-1.7--0.47) |
| Eastern Sub-Saharan Africa | 337892.14 (289404.92-391322.04) | 501.53 (429.56-580.84) | 816716.99 (696437.95-950363.85) | 502.68 (428.65-584.94) | 0.02 (-0.07-0.11) | 1051.71 (780.97-1540.54) | 1.56 (1.16-2.29) | 2193.75 (1668.54-3095.27) | 1.35 (1.03-1.91) | -0.64 (-0.73--0.55) | 88486.95 (71208.17-113080.17) | 131.34 (105.69-167.84) | 199131.03 (158796.19-247882.26) | 122.56 (97.74-152.57) | -0.31 (-0.38--0.23) |
| High-income Asia Pacific | 380679.85 (318975.37-452869.08) | 431.9 (361.89-513.8) | 422894.62 (348177.65-514163.41) | 493.04 (405.93-599.45) | 0.45 (0.4-0.5) | 1721.94 (1589.35-1877.59) | 1.95 (1.8-2.13) | 588.02 (536.37-698.8) | 0.69 (0.63-0.81) | -4.26 (-4.5--4.02) | 130647.48 (111275.46-151385.03) | 148.23 (126.25-171.75) | 88717.53 (67741.01-110871.18) | 103.43 (78.98-129.26) | -1.52 (-1.64--1.41) |
| High-income North America | 669218.62 (540398.89-819774.56) | 473.45 (382.31-579.96) | 1002353.79 (807438.49-1225966.56) | 602.39 (485.25-736.78) | 1.52 (1.19-1.86) | 1296.22 (1229.73-1375.34) | 0.92 (0.87-0.97) | 917.84 (858.99-1001.96) | 0.55 (0.52-0.6) | -1.79 (-1.92--1.67) | 144652.97 (116508.05-175883.17) | 102.34 (82.43-124.43) | 170467.99 (128890.4-218804.97) | 102.45 (77.46-131.5) | 0.48 (0.27-0.68) |
| North Africa and Middle East | 740051.84 (652081.4-838872.73) | 543.89 (479.24-616.52) | 2008734.31 (1767063.25-2281044.37) | 650.99 (572.67-739.24) | 0.71 (0.64-0.78) | 6033.23 (5329.67-7707.31) | 4.43 (3.92-5.66) | 16566.37 (13754.93-19840.93) | 5.37 (4.46-6.43) | 0.95 (0.79-1.12) | 370821.41 (326234.38-443810.99) | 272.53 (239.76-326.17) | 1002815.34 (848214.16-1166882.37) | 324.99 (274.89-378.16) | 0.85 (0.73-0.97) |
| Oceania | 11655.15 (10218.12-13202.59) | 435.59 (381.88-493.42) | 28227.23 (24852.67-31980.28) | 467.91 (411.97-530.12) | 0.32 (0.27-0.37) | 67.24 (47.5-98.93) | 2.51 (1.78-3.7) | 170.26 (116.92-257.22) | 2.82 (1.94-4.26) | 0.46 (0.39-0.52) | 5110.82 (4003.67-6579.73) | 191.01 (149.63-245.9) | 12641.12 (9665.36-16784.98) | 209.55 (160.22-278.24) | 0.38 (0.33-0.43) |
| South Asia | 1528018.82 (1250261.86-1834129.23) | 334.88 (274.01-401.97) | 3310541.43 (2715271.26-3964199.56) | 377.97 (310.01-452.6) | 0.51 (0.38-0.64) | 7194.97 (5232.62-11517.45) | 1.58 (1.15-2.52) | 14018.47 (10812.89-19848.2) | 1.6 (1.23-2.27) | 0.15 (0.01-0.3) | 513696.42 (398992.48-700127.39) | 112.58 (87.44-153.44) | 1043327.97 (842268.47-1309075.37) | 119.12 (96.16-149.46) | 0.27 (0.17-0.38) |
| Southeast Asia | 911781.67 (770095.72-1082765.16) | 448.79 (379.05-532.95) | 1866628.51 (1565159.26-2213195.79) | 543.14 (455.42-643.98) | 0.78 (0.7-0.86) | 5716.7 (4625.09-6934.62) | 2.81 (2.28-3.41) | 12751.85 (9518.91-15702.23) | 3.71 (2.77-4.57) | 1.44 (1.28-1.59) | 417087.68 (343783.52-494661.71) | 205.3 (169.21-243.48) | 878151.11 (701977.23-1051642.66) | 255.52 (204.26-306) | 1.07 (0.97-1.17) |
| Southern Latin America | 73773.57 (63269.54-85460.31) | 331.55 (284.34-384.07) | 99850.63 (85124.07-115653.13) | 305.9 (260.79-354.32) | -0.22 (-0.29--0.15) | 411.03 (353.11-473.79) | 1.85 (1.59-2.13) | 213.81 (186.33-244.44) | 0.66 (0.57-0.75) | -3.69 (-3.86--3.51) | 27592.83 (23639.83-31530.04) | 124.01 (106.24-141.7) | 22590.44 (18274.63-27279.98) | 69.21 (55.99-83.58) | -2.02 (-2.18--1.86) |
| Southern Sub-Saharan Africa | 130074.91 (107995.09-154773.27) | 597.11 (495.75-710.49) | 220717.79 (184319.92-260556.75) | 572.86 (478.39-676.26) | -0.21 (-0.31--0.12) | 466.58 (407.94-523.26) | 2.14 (1.87-2.4) | 618.77 (517.82-722.38) | 1.61 (1.34-1.87) | -0.92 (-1.39--0.45) | 37555.54 (31906.06-43215.73) | 172.4 (146.47-198.38) | 54588.26 (45582.21-64518.01) | 141.68 (118.31-167.45) | -0.69 (-1.01--0.37) |
| Tropical Latin America | 420012.87 (341507.82-511991.16) | 616.49 (501.26-751.49) | 587109.27 (477671.38-709810.01) | 510.15 (415.05-616.76) | -0.85 (-1.1--0.6) | 3121.85 (2896.56-3367.22) | 4.58 (4.25-4.94) | 1791.5 (1657.09-1951.78) | 1.56 (1.44-1.7) | -3.75 (-4--3.5) | 167281.85 (152506.71-182763.66) | 245.53 (223.85-268.26) | 121986.6 (106704.8-140234.86) | 106 (92.72-121.85) | -2.99 (-3.27--2.71) |
| Western Europe | 687488.08 (581022.54-809973.73) | 363.77 (307.43-428.58) | 711156.59 (598437.51-838190.57) | 356.53 (300.02-420.22) | 0.08 (-0.01-0.17) | 2996.23 (2794.02-3244.78) | 1.59 (1.48-1.72) | 1099.43 (1008.67-1191.4) | 0.55 (0.51-0.6) | -3.7 (-3.86--3.53) | 215055.64 (186713.51-245079.35) | 113.79 (98.8-129.68) | 133779.42 (106547.79-163434.92) | 67.07 (53.42-81.94) | -1.77 (-1.94--1.61) |
| Western Sub-Saharan Africa | 339158.54 (289940.34-392511.03) | 478.56 (409.12-553.85) | 865089.8 (737025.72-1006208.62) | 486.58 (414.55-565.95) | 0.1 (0.03-0.17) | 1707.02 (1340.39-2400.5) | 2.41 (1.89-3.39) | 3392.57 (2582.41-4831.74) | 1.91 (1.45-2.72) | -0.89 (-0.98--0.79) | 119696.52 (97904.79-151609.03) | 168.9 (138.15-213.93) | 264620.97 (215529.77-329647.68) | 148.84 (121.23-185.41) | -0.48 (-0.55--0.41) |

SDI: socio-demographic index; DALYs: disability-adjusted life years.

Table S4 Peripheral artery disease burden in people aged 20-54 across regions during 1990-2019.

|  | Prevalence | | | | | Deaths | | | | | DALY | | | | |
| --- | --- | --- | --- | --- | --- | --- | --- | --- | --- | --- | --- | --- | --- | --- | --- |
|  | 1990 | | 2019 | | 1990-2019 | 1990 | | 2019 | | 1990-2019 | 1990 | | 2019 | | 1990-2019 |
| location | Number | Rate | Number | Rate | EAPC | Number | Rate | Number | Rate | EAPC | Number | Rate | Number | Rate | EAPC |
| Global | 9241630.84 (7305519.13-11415506.6) | 384.24 (303.74-474.62) | 15481989.68 (12250770.48-19088829.69) | 412.59 (326.48-508.71) | 0.36 (0.31-0.42) | 946.7 (545.3-1611.23) | 0.04 (0.02-0.07) | 1730.7 (1144.73-2817.22) | 0.05 (0.03-0.08) | 0.71 (0.57-0.85) | 42431.93 (25800.73-69973.14) | 1.76 (1.07-2.91) | 76205.55 (52236.97-120234.9) | 2.03 (1.39-3.2) | 0.62 (0.5-0.74) |
| SDI region |  |  |  |  |  |  |  |  |  |  |  |  |  |  |  |
| High SDI | 3297557.99 (2625914.6-4035855.33) | 799.93 (637-979.03) | 3269879.29 (2695897.54-3897333.62) | 679.61 (560.32-810.02) | -0.46 (-0.51--0.42) | 276.41 (131.98-524.46) | 0.07 (0.03-0.13) | 440.37 (213.8-821.05) | 0.09 (0.04-0.17) | 1.23 (0.98-1.49) | 12589.11 (6615.9-22811.27) | 3.05 (1.6-5.53) | 18915.07 (9913.21-34054.52) | 3.93 (2.06-7.08) | 1.03 (0.8-1.26) |
| High-middle SDI | 2380319.63 (1884085.26-2926104.92) | 425.04 (336.43-522.5) | 3902971.91 (3071940.25-4836216.36) | 521.95 (410.82-646.76) | 0.85 (0.75-0.96) | 413.96 (191.08-792.84) | 0.07 (0.03-0.14) | 595.96 (312.13-1147.18) | 0.08 (0.04-0.15) | 0.52 (0.29-0.75) | 17472.6 (8683.38-32301.45) | 3.12 (1.55-5.77) | 25302.86 (14049-47283.65) | 3.38 (1.88-6.32) | 0.49 (0.29-0.7) |
| Middle SDI | 2134995.61 (1669316.4-2665996.15) | 274.54 (214.66-342.82) | 5188673.85 (4042211.2-6475003.49) | 421.4 (328.29-525.88) | 1.57 (1.48-1.67) | 135.05 (106.34-180.42) | 0.02 (0.01-0.02) | 344.37 (271.77-450.91) | 0.03 (0.02-0.04) | 1.68 (1.61-1.75) | 6644.98 (5092.59-8587.93) | 0.85 (0.65-1.1) | 16296.95 (12921.02-21284.03) | 1.32 (1.05-1.73) | 1.54 (1.47-1.61) |
| Low-middle SDI | 1061525.84 (826732.5-1320176.52) | 230.74 (179.7-286.96) | 2271754.79 (1769717.93-2827146.45) | 270.02 (210.35-336.04) | 0.61 (0.55-0.67) | 55.09 (41.61-76.91) | 0.01 (0.01-0.02) | 177.9 (142.53-223.32) | 0.02 (0.02-0.03) | 2.12 (2-2.25) | 2839.94 (2080.07-3803.61) | 0.62 (0.45-0.83) | 8304.66 (6595.56-10299.02) | 0.99 (0.78-1.22) | 1.76 (1.64-1.88) |
| Low SDI | 362906.12 (282375.8-453293.63) | 187.1 (145.58-233.7) | 840875.74 (656447.93-1053070.32) | 187.38 (146.29-234.67) | 0.01 (-0.06-0.09) | 65.51 (34.34-98.12) | 0.03 (0.02-0.05) | 170.56 (101.09-246.04) | 0.04 (0.02-0.05) | 0.33 (0.12-0.54) | 2856.06 (1609.7-4173.43) | 1.47 (0.83-2.15) | 7321.18 (4520.63-10452.9) | 1.63 (1.01-2.33) | 0.27 (0.07-0.47) |
| Sub region |  |  |  |  |  |  |  |  |  |  |  |  |  |  |  |
| Andean Latin America | 25689.29 (20185.92-32142.9) | 163.82 (128.73-204.98) | 61002.91 (48015.23-76206.2) | 199.78 (157.25-249.57) | 0.79 (0.75-0.82) | 0.81 (0.63-1.03) | 0.01 (0-0.01) | 2.55 (1.82-3.5) | 0.01 (0.01-0.01) | 1.84 (1.63-2.06) | 48.07 (36.08-63.94) | 0.31 (0.23-0.41) | 134.9 (96.83-181.19) | 0.44 (0.32-0.59) | 1.41 (1.24-1.57) |
| Australasia | 56765.06 (45393.92-69269.38) | 564.09 (451.09-688.35) | 61898.41 (48980.41-76256.33) | 454.33 (359.51-559.72) | -0.69 (-0.74--0.64) | 3.92 (1.76-7.32) | 0.04 (0.02-0.07) | 7.21 (3.47-14.64) | 0.05 (0.03-0.11) | 1.28 (1.05-1.5) | 179.1 (91.94-316.29) | 1.78 (0.91-3.14) | 309.18 (157.69-601.16) | 2.27 (1.16-4.41) | 1.05 (0.85-1.25) |
| Caribbean | 33864.74 (26519.02-42234.95) | 213.45 (167.15-266.21) | 60981.58 (47456.41-76050.47) | 267.47 (208.15-333.57) | 0.96 (0.91-1) | 14.25 (7.28-26.92) | 0.09 (0.05-0.17) | 32.28 (16.98-60.9) | 0.14 (0.07-0.27) | 1.83 (1.66-1.99) | 585.73 (304.77-1095.52) | 3.69 (1.92-6.91) | 1301.62 (699.84-2423.48) | 5.71 (3.07-10.63) | 1.77 (1.61-1.92) |
| Central Asia | 78087.45 (61000.43-97562.25) | 262.47 (205.04-327.93) | 144946.33 (114415.19-180710.76) | 311.51 (245.9-388.38) | 1.38 (1.07-1.69) | 2.89 (1.35-5.6) | 0.01 (0-0.02) | 7.48 (3.71-15.53) | 0.02 (0.01-0.03) | 2.45 (2.15-2.75) | 167.11 (92.52-285.24) | 0.56 (0.31-0.96) | 379.1 (208.91-699.68) | 0.81 (0.45-1.5) | 2.11 (1.76-2.46) |
| Central Europe | 239857.24 (188550.53-299801.12) | 411.61 (323.56-514.47) | 242692.76 (190436.37-301494.73) | 445.85 (349.85-553.88) | 0.19 (0-0.37) | 69.72 (28.78-144.4) | 0.12 (0.05-0.25) | 90.32 (39.12-189.76) | 0.17 (0.07-0.35) | 0.8 (0.56-1.04) | 2891.7 (1280.4-5824.17) | 4.96 (2.2-9.99) | 3685.54 (1654.24-7651.23) | 6.77 (3.04-14.06) | 0.73 (0.5-0.97) |
| Central Latin America | 142693.58 (111991.2-177950.12) | 209.19 (164.18-260.88) | 304420.21 (237368.14-378769.21) | 248.29 (193.6-308.93) | 0.65 (0.61-0.69) | 15.65 (7.24-28.32) | 0.02 (0.01-0.04) | 41.53 (19.97-85.5) | 0.03 (0.02-0.07) | 1.25 (1.14-1.36) | 700.79 (346.2-1211.05) | 1.03 (0.51-1.78) | 1792.78 (947.37-3468.32) | 1.46 (0.77-2.83) | 1.14 (1.04-1.24) |
| Central Sub-Saharan Africa | 39298.78 (30628.42-49014.57) | 194.49 (151.58-242.57) | 102517.69 (80134.44-127730.78) | 197.8 (154.62-246.45) | 0.2 (0.14-0.27) | 25.55 (8.43-48.75) | 0.13 (0.04-0.24) | 66.45 (31.56-119.16) | 0.13 (0.06-0.23) | 0.09 (-0.19-0.36) | 1040.93 (374.4-1966.18) | 5.15 (1.85-9.73) | 2708.58 (1300.47-4790.83) | 5.23 (2.51-9.24) | 0.07 (-0.19-0.33) |
| East Asia | 1977348.43 (1545295.46-2473505.19) | 324.3 (253.44-405.67) | 4958421.23 (3870568.71-6239543.72) | 622.07 (485.59-782.8) | 2.32 (2.14-2.49) | 55.92 (42.1-79.99) | 0.01 (0.01-0.01) | 118.27 (94.19-146.1) | 0.01 (0.01-0.02) | 1.65 (1.57-1.73) | 3315.06 (2386.02-4628.78) | 0.54 (0.39-0.76) | 6994.35 (5253.6-9490.79) | 0.88 (0.66-1.19) | 1.62 (1.49-1.75) |
| Eastern Europe | 509949.46 (397331.02-635417.43) | 462.29 (360.2-576.04) | 502587.74 (393129.63-626113.24) | 499.35 (390.6-622.08) | 0.77 (0.5-1.04) | 239.07 (92.18-492.52) | 0.22 (0.08-0.45) | 302.61 (125.5-686.7) | 0.3 (0.12-0.68) | 1.7 (1.35-2.06) | 9519.41 (3816.58-19395.69) | 8.63 (3.46-17.58) | 12084.77 (5054.26-27093.65) | 12.01 (5.02-26.92) | 1.66 (1.32-2) |
| Eastern Sub-Saharan Africa | 109234.32 (84370.67-136240.02) | 162.14 (125.23-202.22) | 268870.1 (209363.43-334679.77) | 165.49 (128.86-205.99) | 0.05 (-0.04-0.14) | 39.2 (22.46-58.59) | 0.06 (0.03-0.09) | 109.11 (68.69-147.56) | 0.07 (0.04-0.09) | 0.31 (0.12-0.5) | 1654.76 (981.84-2435.14) | 2.46 (1.46-3.61) | 4557.64 (2901.05-6158.66) | 2.81 (1.79-3.79) | 0.27 (0.09-0.46) |
| High-income Asia Pacific | 638227.72 (504664.1-781243.93) | 724.1 (572.57-886.36) | 465405.05 (368361.23-572628.1) | 542.6 (429.46-667.61) | -1.18 (-1.43--0.94) | 11.79 (7.62-20) | 0.01 (0.01-0.02) | 11.36 (7.1-19.48) | 0.01 (0.01-0.02) | -0.6 (-0.84--0.36) | 755.63 (490.38-1145.33) | 0.86 (0.56-1.3) | 652.49 (431.72-1008.02) | 0.76 (0.5-1.18) | -0.86 (-1.08--0.64) |
| High-income North America | 1195238.85 (942049.53-1480328.68) | 845.59 (666.46-1047.28) | 1362952.92 (1160809.1-1576702.28) | 819.1 (697.62-947.56) | 0.21 (0.05-0.38) | 154.01 (72.8-291.54) | 0.11 (0.05-0.21) | 283.37 (134.55-546.23) | 0.17 (0.08-0.33) | 1.77 (1.41-2.12) | 6728.3 (3459.18-12510.45) | 4.76 (2.45-8.85) | 11857.91 (5955.86-22513.27) | 7.13 (3.58-13.53) | 1.62 (1.28-1.96) |
| North Africa and Middle East | 310499.44 (242388.48-387086) | 228.2 (178.14-284.48) | 849886.35 (665708.03-1057579.3) | 275.43 (215.74-342.74) | 0.66 (0.57-0.76) | 28.63 (20.05-41.63) | 0.02 (0.01-0.03) | 73.44 (56.92-92.18) | 0.02 (0.02-0.03) | 0.31 (0.1-0.51) | 1350 (965.29-1912.31) | 0.99 (0.71-1.41) | 3385.38 (2643.68-4202.57) | 1.1 (0.86-1.36) | 0.22 (0.04-0.41) |
| Oceania | 8069.26 (6330.62-10058.77) | 301.57 (236.59-375.93) | 22884.11 (17927.11-28437.17) | 379.34 (297.17-471.39) | 0.91 (0.86-0.96) | 0.25 (0.17-0.41) | 0.01 (0.01-0.02) | 0.99 (0.63-1.58) | 0.02 (0.01-0.03) | 2.23 (1.77-2.7) | 15.59 (10.26-23.73) | 0.58 (0.38-0.89) | 55.19 (36.86-85.12) | 0.91 (0.61-1.41) | 1.84 (1.5-2.19) |
| South Asia | 901398.82 (700055.56-1124606.24) | 197.55 (153.42-246.47) | 1952577.76 (1521789.63-2443256.27) | 222.93 (173.75-278.95) | 0.4 (0.36-0.45) | 31.78 (25.21-49.59) | 0.01 (0.01-0.01) | 120.26 (91.88-158.97) | 0.01 (0.01-0.02) | 2.59 (2.45-2.73) | 1771.21 (1314.31-2572.55) | 0.39 (0.29-0.56) | 5758.28 (4347.78-7623.26) | 0.66 (0.5-0.87) | 2.02 (1.89-2.15) |
| Southeast Asia | 667946.21 (523011.78-830520.47) | 328.77 (257.43-408.79) | 1646437.44 (1290421.98-2049972.12) | 479.07 (375.48-596.49) | 1.54 (1.45-1.63) | 16.38 (13.11-19.88) | 0.01 (0.01-0.01) | 60.89 (46.17-77.68) | 0.02 (0.01-0.02) | 3.02 (2.84-3.21) | 1062.3 (778.63-1468.5) | 0.52 (0.38-0.72) | 3327.49 (2421.47-4429.25) | 0.97 (0.7-1.29) | 2.44 (2.31-2.57) |
| Southern Latin America | 135002.86 (107024.91-164186.2) | 606.72 (480.99-737.88) | 164715.58 (129913.07-202377.66) | 504.62 (398-620.01) | -0.55 (-0.61--0.49) | 3.75 (1.71-6.91) | 0.02 (0.01-0.03) | 8.24 (3.93-16.34) | 0.03 (0.01-0.05) | 1.67 (1.47-1.86) | 230.53 (131.13-376.39) | 1.04 (0.59-1.69) | 417.31 (231.76-744.66) | 1.28 (0.71-2.28) | 0.98 (0.84-1.12) |
| Southern Sub-Saharan Africa | 54015.4 (42180.31-67480.48) | 247.96 (193.63-309.77) | 100394.21 (78149.27-125519.97) | 260.57 (202.83-325.78) | 0.16 (0.06-0.25) | 42.96 (34.33-53.28) | 0.2 (0.16-0.24) | 90.73 (70.84-111.64) | 0.24 (0.18-0.29) | 0.59 (0.33-0.84) | 1793.36 (1445.47-2223.23) | 8.23 (6.64-10.21) | 3712.11 (2946.37-4548.21) | 9.63 (7.65-11.8) | 0.49 (0.24-0.74) |
| Tropical Latin America | 151050.05 (117759.36-187777.33) | 221.71 (172.84-275.62) | 279122.16 (217881.38-349736.61) | 242.53 (189.32-303.89) | 0.31 (0.25-0.36) | 53.05 (23.16-101.53) | 0.08 (0.03-0.15) | 126.32 (57.38-242.97) | 0.11 (0.05-0.21) | 1.17 (1.13-1.21) | 2213.76 (1000.11-4157.49) | 3.25 (1.47-6.1) | 5187.09 (2437.83-9852) | 4.51 (2.12-8.56) | 1.1 (1.05-1.14) |
| Western Europe | 1851777.97 (1499282.31-2254822.11) | 979.83 (793.31-1193.09) | 1626494.85 (1293536.16-1991703.8) | 815.42 (648.5-998.52) | -0.69 (-0.82--0.55) | 133.77 (57.92-269.58) | 0.07 (0.03-0.14) | 169.62 (76.02-344.45) | 0.09 (0.04-0.17) | 0.7 (0.66-0.75) | 6199.95 (3120.58-11663.99) | 3.28 (1.65-6.17) | 7417.98 (3584.99-14288.22) | 3.72 (1.8-7.16) | 0.49 (0.44-0.55) |
| Western Sub-Saharan Africa | 115615.91 (89632.45-144645.15) | 163.14 (126.47-204.1) | 302780.29 (235676.1-378549.67) | 170.3 (132.56-212.92) | 0.23 (0.15-0.31) | 3.35 (2.58-4.49) | 0 (0-0.01) | 7.66 (5.88-9.84) | 0 (0-0.01) | -0.45 (-0.67--0.22) | 208.64 (151.17-286) | 0.29 (0.21-0.4) | 485.86 (352.76-665.52) | 0.27 (0.2-0.37) | -0.33 (-0.51--0.14) |

SDI: socio-demographic index; DALYs: disability-adjusted life years.

Table S5 Ischemic heart disease burden in people older than 55+ across regions during 1990-2019.

|  | Prevalence | | | | | Deaths | | | | | DALY | | | | |
| --- | --- | --- | --- | --- | --- | --- | --- | --- | --- | --- | --- | --- | --- | --- | --- |
|  | 1990 | | 2019 | | 1990-2019 | 1990 | | 2019 | | 1990-2019 | 1990 | | 2019 | | 1990-2019 |
| location | Number | Rate | Number | Rate | EAPC | Number | Rate | Number | Rate | EAPC | Number | Rate | Number | Rate | EAPC |
| Global | 80109726.11 (70890303.07-90356143.17) | 11937.19 (10563.4-13464.01) | 165702961.67 (147337390.19-186382461.96) | 11787.04 (10480.63-13258.05) | -0.12 (-0.16--0.08) | 4986831.87 (4708601.07-5164764.07) | 743.09 (701.63-769.6) | 8116548.54 (7389086.62-8645624.86) | 577.36 (525.61-614.99) | -0.99 (-1.04--0.93) | 89097452.34 (85553910.35-92211022.86) | 7108.47 (6825.75-7356.88) | 136353278.53 (127375214.24-144906999.57) | 4974.08 (4646.57-5286.12) | -1.31 (-1.35--1.27) |
| SDI region |  |  |  |  |  |  |  |  |  |  |  |  |  |  |  |
| High SDI | 20450741.78 (18170355.29-22937959.42) | 11645 (10346.51-13061.26) | 27618088.03 (25031269.9-30465813.29) | 8868.5 (8037.84-9782.94) | -1.18 (-1.28--1.08) | 1591806.73 (1476268.45-1647093.45) | 906.4 (840.61-937.88) | 1374855.5 (1197724.57-1476558.95) | 441.48 (384.6-474.14) | -2.9 (-3.08--2.72) | 25169620.69 (23983310.28-25802347.73) | 6947.74 (6620.28-7122.4) | 18995359.95 (17427904.99-20045353.86) | 2829.37 (2595.9-2985.77) | -3.42 (-3.61--3.23) |
| High-middle SDI | 23948831.62 (21094965.78-27189084.96) | 12934.21 (11392.9-14684.19) | 44169156.62 (38878691.14-50114955.56) | 12453.47 (10961.83-14129.89) | -0.24 (-0.34--0.14) | 1682942.02 (1596054.94-1732939.83) | 908.92 (861.99-935.92) | 2473775.89 (2241651.86-2637797.03) | 697.48 (632.03-743.73) | -1.15 (-1.35--0.94) | 29323858.79 (28176738.85-30093635.93) | 8487.35 (8155.33-8710.15) | 38770255.31 (35863299.04-41209207.28) | 5538.58 (5123.3-5887) | -1.71 (-1.9--1.52) |
| Middle SDI | 18579854.06 (16276296.98-21185934.05) | 10791.28 (9453.36-12304.91) | 50782094.28 (44746355.91-57614912.07) | 11844.66 (10436.85-13438.37) | 0.31 (0.25-0.36) | 944064.8 (889358.74-1004240.15) | 548.32 (516.54-583.27) | 2470231.4 (2247752.33-2673044.17) | 576.17 (524.28-623.47) | 0.31 (0.22-0.4) | 18469749.37 (17400165.32-19571011.37) | 6058.98 (5708.11-6420.25) | 43730769.92 (40172102.74-47153405.12) | 5498.44 (5050.99-5928.78) | -0.18 (-0.24--0.11) |
| Low-middle SDI | 12687786.85 (11304396.53-14290875.66) | 12816.18 (11418.79-14435.49) | 32889292.95 (29399877.75-37156362.78) | 14469.53 (12934.37-16346.81) | 0.49 (0.46-0.51) | 553267.35 (506615.24-601344.43) | 558.87 (511.74-607.43) | 1351200.77 (1218332.42-1477886.1) | 594.46 (536-650.19) | 0.27 (0.22-0.33) | 11586805.64 (10642546.67-12567081.97) | 6703.03 (6156.77-7270.12) | 26008607.8 (23563616.37-28494501.01) | 6130.54 (5554.23-6716.5) | -0.26 (-0.34--0.19) |
| Low SDI | 4397767.48 (3925853.63-4951782.9) | 11332.81 (10116.71-12760.47) | 10150619.58 (9028167.37-11443854.21) | 12234.32 (10881.46-13793.03) | 0.28 (0.26-0.31) | 211978.97 (189420.9-238611.72) | 546.26 (488.13-614.89) | 442174.62 (391896.44-495434.13) | 532.94 (472.34-597.14) | -0.1 (-0.16--0.03) | 4498142.33 (4026096.3-5030559.5) | 6685.28 (5983.71-7476.57) | 8773290.88 (7831216.51-9855668.91) | 5890.19 (5257.7-6616.87) | -0.45 (-0.49--0.4) |
| Sub-region |  |  |  |  |  |  |  |  |  |  |  |  |  |  |  |
| Andean Latin America | 179584.8 (152424.18-213788.06) | 5344.08 (4535.84-6361.9) | 529541.13 (448105.07-625411.42) | 5645.4 (4777.21-6667.46) | 0.22 (0.19-0.24) | 16943.18 (14976.63-18859.84) | 504.19 (445.67-561.23) | 31133.74 (25570.11-36805.33) | 331.91 (272.6-392.38) | -1.38 (-1.64--1.12) | 287911.81 (256945.6-321518.59) | 4569.65 (4078.17-5103.05) | 486400.75 (404891.83-578526.58) | 2652.16 (2207.72-3154.49) | -1.84 (-2.12--1.56) |
| Australasia | 657919.49 (604737.48-711896.59) | 16700.56 (15350.59-18070.71) | 1237543.03 (1142772.61-1342057.74) | 15029.99 (13879-16299.32) | -0.48 (-0.52--0.45) | 38083.41 (35515.81-39427.97) | 966.71 (901.53-1000.84) | 31823.79 (27133.92-34443.5) | 386.5 (329.54-418.32) | -3.74 (-3.96--3.53) | 629068.78 (598501.1-646120.62) | 7802.34 (7423.21-8013.84) | 408997.47 (370468.69-435885.17) | 2335.36 (2115.37-2488.89) | -4.59 (-4.82--4.36) |
| Caribbean | 702009.91 (635898.44-777328.24) | 16249.53 (14719.24-17992.94) | 1402068.53 (1273720.9-1544080.62) | 15867.99 (14415.41-17475.22) | -0.07 (-0.08--0.06) | 39056.24 (36510.63-40808.91) | 904.04 (845.12-944.61) | 56858.43 (49278.47-64710.42) | 643.5 (557.71-732.36) | -1.29 (-1.48--1.11) | 679469.97 (644856.8-713339.45) | 7962.69 (7557.06-8359.61) | 952766.47 (832823.54-1089134.07) | 5426.18 (4743.08-6202.82) | -1.4 (-1.6--1.2) |
| Central Asia | 1512594.35 (1380428.87-1649965.96) | 18970.85 (17313.24-20693.75) | 2296185.29 (2106949.89-2516522.09) | 17637.62 (16184.05-19330.08) | -0.23 (-0.49-0.03) | 116866.23 (109984.56-120790.54) | 1465.73 (1379.42-1514.95) | 177844.81 (163679.82-193394.59) | 1366.07 (1257.27-1485.52) | -0.37 (-0.95-0.21) | 2019805.72 (1940663.51-2074672.71) | 14021.44 (13472.04-14402.33) | 3266575.6 (2986022.77-3578609.72) | 15050.66 (13758.02-16488.34) | -0.1 (-0.5-0.31) |
| Central Europe | 4330488.45 (3780672.15-4989413.91) | 16570.91 (14467-19092.33) | 5467257.65 (4772950.2-6241269.05) | 15022.13 (13114.41-17148.84) | -0.55 (-0.65--0.44) | 359791.66 (341997.93-368530.78) | 1376.77 (1308.68-1410.21) | 340770.02 (296448.21-380228.8) | 936.32 (814.54-1044.74) | -1.78 (-1.93--1.64) | 6119598.44 (5887869.98-6253774.26) | 12379.15 (11910.39-12650.57) | 4900877.23 (4325442.84-5486934.66) | 6401.59 (5649.95-7167.1) | -2.63 (-2.75--2.51) |
| Central Latin America | 1303845.38 (1153734.23-1482054.09) | 9573.29 (8471.12-10881.76) | 3793114.98 (3344157.46-4310763.69) | 9491.56 (8368.13-10786.88) | -0.08 (-0.11--0.06) | 79866.46 (74326.78-83100) | 586.41 (545.73-610.15) | 197431.19 (170180.55-225368.77) | 494.03 (425.84-563.94) | -0.69 (-0.83--0.55) | 1414769.18 (1348573.53-1463988.97) | 5577.58 (5316.61-5771.63) | 3169096.05 (2763569.63-3633392.33) | 4104.91 (3579.63-4706.31) | -1.19 (-1.34--1.04) |
| Central Sub-Saharan Africa | 282559.4 (253128.05-316110.03) | 7443.01 (6667.74-8326.78) | 617975.94 (553996.35-688442.63) | 7222.97 (6475.17-8046.59) | -0.19 (-0.27--0.1) | 18826.47 (15687.06-22999.08) | 495.92 (413.22-605.83) | 38952.21 (29708.39-50271.36) | 455.28 (347.23-587.58) | -0.4 (-0.57--0.23) | 410704.6 (341095.75-499904.3) | 6505.13 (5402.6-7917.96) | 788153.72 (601594.36-1024764.09) | 5469.43 (4174.79-7111.4) | -0.67 (-0.71--0.63) |
| East Asia | 13585332.58 (11487569.6-16073676.55) | 9060.83 (7661.71-10720.45) | 37595130.7 (32095524.65-44012193.2) | 10321.76 (8811.84-12083.57) | 0.43 (0.35-0.51) | 526333.93 (468857.64-585605.86) | 351.04 (312.71-390.57) | 1768630.79 (1526662.06-1998096.62) | 485.58 (419.15-548.58) | 1.7 (1.45-1.96) | 10393281 (9241567.92-11559983.65) | 3909.16 (3475.98-4347.99) | 28539607.94 (24848068.62-32405045.61) | 4121.35 (3588.26-4679.55) | 0.79 (0.53-1.05) |
| Eastern Europe | 8584454.53 (7616938.79-9687946.03) | 17562.18 (15582.82-19819.72) | 11773919.97 (10451750.7-13237288.77) | 19021.52 (16885.47-21385.69) | 0.26 (0.07-0.45) | 731230.25 (698263.17-748300.54) | 1495.96 (1428.52-1530.88) | 932256.97 (830672.13-1016192.03) | 1506.12 (1342-1641.72) | -0.25 (-0.69-0.19) | 12337954.38 (11956648.94-12583692.24) | 13425.06 (13010.16-13692.45) | 14744202.81 (13289332.77-16117016.21) | 12032.27 (10845-13152.58) | -0.76 (-1.18--0.34) |
| Eastern Sub-Saharan Africa | 995884.51 (868141.78-1146826.18) | 8188.44 (7138.1-9429.52) | 2240190.98 (1955948.34-2578092.79) | 8688.62 (7586.18-9999.17) | 0.19 (0.13-0.24) | 48121.18 (41375.53-53884.63) | 395.67 (340.2-443.05) | 99255.13 (79853.45-118252.49) | 384.96 (309.71-458.64) | -0.13 (-0.19--0.08) | 1002512.19 (868800.01-1124018.03) | 4691.38 (4065.66-5259.99) | 1931110.28 (1571125.52-2290525.24) | 4227.32 (3439.29-5014.1) | -0.41 (-0.44--0.37) |
| High-income Asia Pacific | 2054756.1 (1814732.22-2317489.87) | 5873.18 (5187.11-6624.16) | 4203391.18 (3757266.64-4663111.28) | 6072.33 (5427.85-6736.46) | -0.01 (-0.13-0.1) | 136541.54 (126340.64-142884.44) | 390.28 (361.12-408.41) | 166726.13 (133852.77-185376.65) | 240.86 (193.37-267.8) | -1.69 (-1.96--1.43) | 2204826.12 (2086951.58-2277112.32) | 3293.26 (3117.2-3401.24) | 2082309.2 (1780265.81-2255771.23) | 1295.95 (1107.97-1403.9) | -3.19 (-3.42--2.96) |
| High-income North America | 7954716.94 (7029449.9-8975409.41) | 13686.23 (12094.29-15442.35) | 8766011.02 (7865843.6-9729332.26) | 8099.86 (7268.1-8989.98) | -2.27 (-2.46--2.08) | 618011.42 (569463.83-642735.82) | 1063.3 (979.77-1105.84) | 574877.86 (508307.9-614102.19) | 531.19 (469.68-567.44) | -2.99 (-3.21--2.76) | 9694958.88 (9167652.21-9964535.02) | 7832.09 (7406.11-8049.87) | 8342672.62 (7733533.93-8786759.29) | 3739.47 (3466.43-3938.53) | -2.92 (-3.1--2.74) |
| North Africa and Middle East | 6623286.2 (6095644.34-7202577.48) | 23104.51 (21263.9-25125.3) | 16130604.43 (14851924.69-17597019.08) | 22607.12 (20815.04-24662.31) | -0.11 (-0.19--0.02) | 366108.39 (338770.28-391823.22) | 1277.12 (1181.76-1366.83) | 675122.43 (600691.88-760083.58) | 946.19 (841.87-1065.26) | -1.17 (-1.25--1.09) | 7317207.94 (6802835.61-7887214.6) | 14441.6 (13426.41-15566.59) | 12464094.73 (11018803.18-14106986.78) | 9688.86 (8565.37-10965.95) | -1.57 (-1.66--1.47) |
| Oceania | 47074.01 (40769.35-55188.16) | 9728.72 (8425.75-11405.66) | 115070.48 (99510.41-134354.46) | 10134.23 (8763.86-11832.57) | 0.05 (-0.04-0.15) | 3298.9 (2705.46-4139.98) | 681.78 (559.13-855.6) | 8533.96 (6869.37-10574.9) | 751.58 (604.98-931.33) | 0.34 (0.21-0.46) | 75416.74 (61554.19-94645.44) | 9267.26 (7563.82-11630.1) | 190258.29 (151936.46-236384.07) | 9998.74 (7984.79-12422.81) | 0.41 (0.34-0.48) |
| South Asia | 13752859.25 (12241660.14-15554948.21) | 14744.41 (13124.26-16676.42) | 39967773.83 (35548875.66-45152961.8) | 17021.64 (15139.7-19229.93) | 0.56 (0.52-0.6) | 564373.13 (503377.56-625589.43) | 605.06 (539.67-670.69) | 1491742.03 (1307258.16-1680498.98) | 635.31 (556.74-715.7) | 0.18 (0.1-0.26) | 12310950.11 (11060382.47-13571118.5) | 7782.02 (6991.51-8578.6) | 29561600.54 (26070137.24-33353080.72) | 6792.12 (5989.92-7663.26) | -0.47 (-0.57--0.37) |
| Southeast Asia | 3066869.68 (2653144.56-3555300.93) | 7243.65 (6266.47-8397.28) | 7699429.5 (6655657.96-8927784.81) | 7356.2 (6358.96-8529.8) | 0.03 (-0.06-0.12) | 197797.08 (179773.35-215409.5) | 467.18 (424.61-508.78) | 487681.41 (436551.72-532891.12) | 465.94 (417.09-509.14) | 0.03 (-0.14-0.19) | 3908121.94 (3555620.62-4257698.6) | 5236.37 (4764.07-5704.76) | 9264447.49 (8385537.28-10131813.74) | 4964.84 (4493.83-5429.66) | -0.13 (-0.2--0.06) |
| Southern Latin America | 539327.8 (488777.11-591356.43) | 6811.44 (6173.01-7468.54) | 842426.09 (770208.01-917674.66) | 5956.57 (5445.94-6488.64) | -0.55 (-0.58--0.52) | 60994.68 (57263.72-63166.1) | 770.33 (723.21-797.76) | 57350.24 (51718.83-61182.25) | 405.51 (365.69-432.6) | -2.22 (-2.36--2.09) | 1017887.12 (977511.47-1046226.4) | 6606.1 (6344.06-6790.02) | 882292.13 (824736.31-929972.85) | 3053.56 (2854.36-3218.58) | -2.63 (-2.8--2.46) |
| Southern Sub-Saharan Africa | 461212.09 (406923.13-525558.26) | 10246.96 (9040.8-11676.57) | 913019.42 (806157.27-1038369.73) | 9680.14 (8547.15-11009.15) | -0.39 (-0.5--0.28) | 17483.28 (15557.07-19119.12) | 388.43 (345.64-424.78) | 38085.27 (34458.18-41438.97) | 403.79 (365.34-439.35) | 0.11 (-0.31-0.53) | 329160.62 (295158.1-359804.78) | 3928.52 (3522.7-4294.25) | 707768.62 (644682.21-771923.85) | 4115.1 (3748.3-4488.11) | 0.26 (-0.16-0.68) |
| Tropical Latin America | 1179602.9 (983946.28-1408155.85) | 7802.96 (6508.71-9314.81) | 3405103.4 (2860061.04-4031817.18) | 8166.71 (6859.5-9669.8) | 0.17 (0.14-0.21) | 98408.1 (92708.69-101973.69) | 650.96 (613.26-674.55) | 153297.24 (138306.77-162193.68) | 367.66 (331.71-389) | -1.94 (-2--1.88) | 1903949.35 (1825324.12-1964667.44) | 6886.62 (6602.24-7106.24) | 2818286.13 (2632581.72-2956621.42) | 3516.16 (3284.47-3688.75) | -2.25 (-2.34--2.16) |
| Western Europe | 11139801.73 (9792522.34-12544396.61) | 11461.1 (10074.96-12906.2) | 14104600.83 (12696293.25-15587990.58) | 9754.94 (8780.93-10780.87) | -0.72 (-0.79--0.65) | 872942.2 (816980.36-902247.69) | 898.12 (840.54-928.27) | 645857.4 (566236.05-696222.38) | 446.68 (391.62-481.52) | -2.81 (-3--2.62) | 13615687.86 (12999502.27-13965267.58) | 6692.82 (6389.93-6864.66) | 8231160.84 (7478827.83-8725070.07) | 2558.53 (2324.68-2712.05) | -3.7 (-3.91--3.49) |
| Western Sub-Saharan Africa | 1155546.03 (1024634.75-1310845.34) | 8041.77 (7130.72-9122.54) | 2602603.28 (2300362.18-2965087.28) | 8604.28 (7605.06-9802.66) | 0.27 (0.18-0.37) | 75754.11 (63339.66-92932.32) | 527.19 (440.8-646.74) | 142317.51 (120011.8-164622.94) | 470.51 (396.76-544.25) | -0.36 (-0.45--0.27) | 1424209.57 (1188851.46-1766715.47) | 5493.78 (4585.9-6814.97) | 2620599.63 (2195637.87-3065749.75) | 4928.82 (4129.55-5766.05) | -0.36 (-0.43--0.3) |

EAPCs: Estimated annual percentage changes; SDI: socio-demographic index; DALYs: disability-adjusted life years.

Table S6 Ischemic stroke in people older than 55 burden across regions during 1990-2019.

|  | Prevalence | | | | | Deaths | | | | | DALY | | | | |
| --- | --- | --- | --- | --- | --- | --- | --- | --- | --- | --- | --- | --- | --- | --- | --- |
|  | 1990 | | 2019 | | 1990-2019 | 1990 | | 2019 | | 1990-2019 | 1990 | | 2019 | | 1990-2019 |
| location | Number | Rate | Number | Rate | EAPC | Number | Rate | Number | Rate | EAPC | Number | Rate | Number | Rate | EAPC |
| Global | 26653910.94 (23038784.03-31116305.44) | 3971.71 (3433.02-4636.66) | 55905363.93 (48376275.79-65108297.06) | 3976.75 (3441.18-4631.38) | 0 (-0.05-0.05) | 1971343.22 (1822394.07-2146918.98) | 293.75 (271.56-319.91) | 3187919.32 (2873086.82-3423836.25) | 226.77 (204.37-243.55) | -1.13 (-1.26--1) | 34959151.74 (32561457.44-37747632.53) | 2789.15 (2597.85-3011.62) | 55761201.5 (50998881.63-60254758.44) | 2034.13 (1860.41-2198.06) | -1.27 (-1.36--1.18) |
| SDI region |  |  |  |  |  |  |  |  |  |  |  |  |  |  |  |
| High SDI | 8235118.34 (7150640.92-9487934.97) | 4689.22 (4071.7-5402.59) | 12299557.16 (10797906.9-14044567.26) | 3949.53 (3467.34-4509.88) | -0.57 (-0.63--0.52) | 504280.22 (460353.72-526998.04) | 287.15 (262.13-300.08) | 451747.92 (377825.34-492109.55) | 145.06 (121.32-158.02) | -2.85 (-3.05--2.65) | 7874435.25 (7293446.98-8325442.02) | 2173.63 (2013.26-2298.13) | 6860505.43 (6042468.49-7555587.5) | 1021.88 (900.03-1125.41) | -2.94 (-3.1--2.78) |
| High-middle SDI | 8358878.97 (7158421.65-9770776.66) | 4514.44 (3866.1-5276.97) | 15651016.11 (13432698.7-18232462.24) | 4412.8 (3787.34-5140.63) | -0.12 (-0.18--0.05) | 815627.76 (767314.78-853937.5) | 440.5 (414.41-461.19) | 1101609.89 (988797.3-1191562.5) | 310.6 (278.79-335.96) | -1.6 (-1.86--1.34) | 14203813.64 (13445846.91-14901863.77) | 4111.08 (3891.7-4313.12) | 18274752.5 (16609359.77-19768949.65) | 2610.67 (2372.75-2824.12) | -1.92 (-2.13--1.72) |
| Middle SDI | 6464216.33 (5478832.93-7637016.16) | 3754.45 (3182.14-4435.62) | 19235626.56 (16499752.17-22372985.16) | 4486.61 (3848.48-5218.38) | 0.6 (0.55-0.65) | 400415.58 (362867.56-468909.13) | 232.56 (210.76-272.34) | 1021591.77 (908059.92-1127882.43) | 238.28 (211.8-263.07) | 0.12 (-0.03-0.27) | 7970290.8 (7254972.95-9152955.63) | 2614.65 (2379.99-3002.62) | 19386707.47 (17450736.25-21459064.62) | 2437.57 (2194.15-2698.13) | -0.18 (-0.25--0.11) |
| Low-middle SDI | 2510724.17 (2147554.89-2963431.71) | 2536.13 (2169.29-2993.42) | 6505558.33 (5604756.7-7643031.66) | 2862.1 (2465.79-3362.52) | 0.42 (0.37-0.47) | 188964.49 (158450.56-227942.78) | 190.88 (160.05-230.25) | 474906.34 (425561.22-526708.59) | 208.93 (187.22-231.72) | 0.29 (0.19-0.39) | 3660960.94 (3145057.02-4380023.1) | 2117.88 (1819.43-2533.87) | 8639414.29 (7753759.05-9569861.35) | 2036.41 (1827.65-2255.73) | -0.18 (-0.25--0.11) |
| Low SDI | 1072294.68 (937830.15-1248482.7) | 2763.24 (2416.74-3217.27) | 2187993.02 (1917540.25-2532931.18) | 2637.14 (2311.17-3052.89) | -0.21 (-0.24--0.17) | 61175.86 (46748.6-79190.96) | 157.65 (120.47-204.07) | 136518.28 (112448.85-168279.05) | 164.54 (135.53-202.82) | 0.12 (0.03-0.2) | 1234988.31 (990547.03-1556175.5) | 1835.48 (1472.18-2312.84) | 2574191.1 (2168729.32-3132660.48) | 1728.25 (1456.03-2103.2) | -0.24 (-0.29--0.2) |
| Sub-region |  |  |  |  |  |  |  |  |  |  |  |  |  |  |  |
| Andean Latin America | 91330.19 (82676.22-100776.71) | 2717.8 (2460.28-2998.91) | 223700.38 (202901.82-247713.06) | 2384.85 (2163.12-2640.85) | -0.51 (-0.56--0.46) | 4898.53 (4205.98-5566.69) | 145.77 (125.16-165.65) | 9478.51 (7726.26-11444.29) | 101.05 (82.37-122.01) | -1.26 (-1.47--1.05) | 79934.33 (69597.29-89883.7) | 1268.69 (1104.63-1426.61) | 144464.43 (122008.47-171651.42) | 787.71 (665.27-935.95) | -1.71 (-1.95--1.47) |
| Australasia | 138262.55 (123258.44-154819.45) | 3509.64 (3128.78-3929.92) | 187410.2 (166253.19-210521.18) | 2276.1 (2019.15-2556.78) | -1.51 (-1.64--1.38) | 9497.4 (8533.63-10016.39) | 241.08 (216.62-254.26) | 10466.9 (8537.25-11688) | 127.12 (103.69-141.95) | -2.78 (-2.97--2.58) | 141933.59 (131030.19-150444.15) | 1760.4 (1625.17-1865.96) | 130416.87 (113638.64-144190.68) | 744.68 (648.87-823.32) | -3.36 (-3.53--3.19) |
| Caribbean | 151954.64 (135445.85-170292.41) | 3517.32 (3135.19-3941.78) | 291723.04 (261252.89-324226.79) | 3301.59 (2956.74-3669.46) | -0.09 (-0.17--0.01) | 10481.13 (9401.45-11596.71) | 242.61 (217.62-268.43) | 19237.93 (16483.57-22166.57) | 217.73 (186.55-250.87) | -0.35 (-0.42--0.28) | 171810.81 (154128.05-192124.49) | 2013.45 (1806.22-2251.5) | 294265.18 (256025.67-335985.93) | 1675.89 (1458.11-1913.5) | -0.58 (-0.67--0.49) |
| Central Asia | 283022.86 (249087.34-318526.43) | 3549.65 (3124.03-3994.93) | 393779.52 (346321.03-450650.88) | 3024.73 (2660.18-3461.57) | -0.55 (-0.72--0.39) | 29444.67 (26838.45-33019.83) | 369.29 (336.61-414.13) | 38951.6 (35458.32-42574.05) | 299.2 (272.36-327.02) | -0.82 (-1.32--0.32) | 534583.95 (489066.23-601553.97) | 3711.07 (3395.09-4175.97) | 740378.33 (677492.13-813251.26) | 3411.27 (3121.53-3747.03) | -0.56 (-0.88--0.23) |
| Central Europe | 1448882.71 (1267241.83-1658036.58) | 5544.25 (4849.19-6344.59) | 1625765.58 (1413212.81-1874653.52) | 4467.04 (3883.02-5150.9) | -0.85 (-0.92--0.79) | 138032.83 (129668.77-143236.43) | 528.19 (496.19-548.1) | 151076.99 (131683.57-168820.53) | 415.11 (361.82-463.86) | -1.2 (-1.39--1) | 2392023.78 (2266941.43-2503567.33) | 4838.75 (4585.73-5064.39) | 2299712.35 (2014798.42-2558739.4) | 3003.91 (2631.75-3342.26) | -1.92 (-2.09--1.76) |
| Central Latin America | 487244.77 (426812.42-556391.51) | 3577.52 (3133.81-4085.22) | 1054758.75 (927840.77-1208437.64) | 2639.34 (2321.75-3023.89) | -1.22 (-1.37--1.08) | 21938.77 (19950.61-23224.77) | 161.08 (146.48-170.52) | 42380.35 (35754.33-48777.64) | 106.05 (89.47-122.06) | -1.66 (-1.79--1.53) | 370756.49 (345064.61-393143.87) | 1461.67 (1360.38-1549.93) | 661544.26 (579834.14-750258.42) | 856.89 (751.06-971.8) | -2.06 (-2.22--1.9) |
| Central Sub-Saharan Africa | 128306.5 (114407.01-144709.44) | 3379.77 (3013.64-3811.85) | 262833.07 (235346.49-293837.68) | 3072.02 (2750.75-3434.41) | -0.45 (-0.5--0.39) | 6201.81 (4631.89-7887.58) | 163.36 (122.01-207.77) | 14723.19 (11195.25-19127.44) | 172.09 (130.85-223.56) | 0.11 (-0.11-0.33) | 133494.63 (105406.49-166427.05) | 2114.42 (1669.53-2636.03) | 283975.47 (223185.08-361930.9) | 1970.66 (1548.8-2511.64) | -0.31 (-0.38--0.24) |
| East Asia | 5607925.86 (4641604.52-6704250.4) | 3740.24 (3095.75-4471.45) | 20524929.99 (17269717.34-24073722.59) | 5635.13 (4741.41-6609.45) | 1.4 (1.32-1.47) | 369527.11 (324618.4-443713.19) | 246.46 (216.51-295.94) | 1020941.24 (875648.33-1162138.68) | 280.3 (240.41-319.07) | 0.47 (0.26-0.69) | 7696837.39 (6822711.3-8995753.63) | 2894.97 (2566.19-3383.52) | 19748621.16 (17340899.76-22422461.8) | 2851.86 (2504.17-3237.99) | 0.05 (-0.08-0.17) |
| Eastern Europe | 2769969.91 (2355911.23-3277911.8) | 5666.84 (4819.75-6705.99) | 2641971.87 (2247663.64-3110094.91) | 4268.27 (3631.24-5024.56) | -0.97 (-1.05--0.89) | 368199.4 (350348.81-378482.26) | 753.27 (716.75-774.3) | 345116.56 (303934.26-378270.97) | 557.56 (491.03-611.12) | -1.65 (-2.12--1.18) | 6295839.05 (6039860.67-6520941.9) | 6850.57 (6572.04-7095.51) | 5407988.67 (4838050.39-5959981.63) | 4413.28 (3948.18-4863.75) | -2.15 (-2.57--1.73) |
| Eastern Sub-Saharan Africa | 423884.91 (373741.22-488338.7) | 3485.3 (3073-4015.26) | 868285.93 (767696.33-993930.63) | 3367.66 (2977.52-3854.98) | -0.17 (-0.2--0.14) | 18432.29 (14495.09-23537.03) | 151.56 (119.18-193.53) | 45023.74 (36955.31-53374.87) | 174.63 (143.33-207.02) | 0.54 (0.47-0.6) | 379713.24 (311828.92-476066.35) | 1776.92 (1459.24-2227.81) | 851670.55 (723241.71-1003485.42) | 1864.36 (1583.22-2196.69) | 0.19 (0.16-0.22) |
| High-income Asia Pacific | 1724228.75 (1511674.38-1964386.88) | 4928.42 (4320.87-5614.87) | 3052009.8 (2635590.58-3533451.21) | 4409.02 (3807.45-5104.52) | -0.55 (-0.66--0.45) | 100045.08 (90780.01-105163.67) | 285.96 (259.48-300.59) | 114110.9 (89116.97-129073.4) | 164.85 (128.74-186.46) | -2.38 (-2.64--2.12) | 1646956.25 (1510811.78-1752368.43) | 2460 (2256.64-2617.45) | 1703766.54 (1435326.84-1925967.8) | 1060.36 (893.29-1198.65) | -3.31 (-3.5--3.12) |
| High-income North America | 3302895.87 (2774366.68-3879881.97) | 5682.69 (4773.35-6675.4) | 5276278.63 (4573671.23-6088031.66) | 4875.32 (4226.11-5625.39) | -0.48 (-0.58--0.38) | 112009.93 (99681.05-118433.13) | 192.72 (171.5-203.77) | 120004.06 (101608.76-132710.95) | 110.88 (93.89-122.63) | -2.6 (-2.89--2.3) | 1899110.47 (1717742.98-2066162.12) | 1534.2 (1387.68-1669.15) | 2068819.76 (1805652.42-2316676.08) | 927.32 (809.36-1038.41) | -2.08 (-2.24--1.92) |
| North Africa and Middle East | 1408352.92 (1250845.05-1608302.47) | 4912.86 (4363.42-5610.36) | 3737352.41 (3326992.65-4223393.06) | 5237.92 (4662.8-5919.11) | 0.28 (0.23-0.34) | 78060.37 (68394.09-88090.71) | 272.3 (238.58-307.29) | 192391.96 (172328.5-213290.2) | 269.64 (241.52-298.93) | 0.19 (0-0.37) | 1527120.03 (1361710.98-1717971.52) | 3014 (2687.54-3390.67) | 3622099.02 (3266392.89-3980995.44) | 2815.61 (2539.1-3094.59) | -0.08 (-0.18-0.01) |
| Oceania | 16632.59 (15012.43-18615.39) | 3437.44 (3102.6-3847.22) | 38995.22 (35169.29-43282.12) | 3434.3 (3097.35-3811.85) | -0.12 (-0.18--0.05) | 562.57 (418.45-760.41) | 116.27 (86.48-157.15) | 1361.36 (1072.84-1801.72) | 119.89 (94.48-158.68) | -0.12 (-0.29-0.06) | 13583.11 (10770.89-17565.08) | 1669.1 (1323.53-2158.41) | 31518.04 (25790.88-39975.64) | 1656.38 (1355.4-2100.86) | -0.11 (-0.17--0.05) |
| South Asia | 1590178.94 (1329629.02-1950066.74) | 1704.83 (1425.49-2090.66) | 4273607.02 (3585202.3-5215551.16) | 1820.06 (1526.88-2221.22) | 0.19 (0.11-0.27) | 146803.51 (114688.02-188129.46) | 157.39 (122.96-201.69) | 363309.85 (309516.68-425803.25) | 154.73 (131.82-181.34) | -0.26 (-0.4--0.12) | 2840042.04 (2268260.64-3619034.71) | 1795.25 (1433.82-2287.67) | 6562722.74 (5605945.04-7698755.02) | 1507.86 (1288.03-1768.88) | -0.79 (-0.9--0.67) |
| Southeast Asia | 1769151.23 (1525508.49-2064412.13) | 4178.56 (3603.1-4875.94) | 4603948.53 (3962191.39-5418083.4) | 4398.71 (3785.56-5176.55) | 0.15 (0.09-0.21) | 105128.96 (90785.99-120469.23) | 248.3 (214.43-284.54) | 288125.48 (247365.27-321576.33) | 275.28 (236.34-307.24) | 0.61 (0.39-0.83) | 2051286.9 (1788720.04-2345489.31) | 2748.46 (2396.65-3142.65) | 5386286.16 (4644689.35-6031840.04) | 2886.52 (2489.1-3232.48) | 0.35 (0.24-0.46) |
| Southern Latin America | 247263.71 (221374-277358.26) | 3122.82 (2795.84-3502.9) | 337235.05 (302150.29-376464.47) | 2384.5 (2136.43-2661.88) | -1.09 (-1.16--1.01) | 19352.58 (17576.65-21010.71) | 244.41 (221.98-265.35) | 21400.94 (18774.22-23345.73) | 151.32 (132.75-165.07) | -1.75 (-1.86--1.64) | 321286.04 (293090.38-349084.27) | 2085.15 (1902.16-2265.56) | 319958.02 (289732.08-347430.58) | 1107.35 (1002.74-1202.43) | -2.27 (-2.41--2.13) |
| Southern Sub-Saharan Africa | 196278.59 (168686.2-231796.56) | 4360.81 (3747.78-5149.93) | 411259.39 (347563.73-488545.5) | 4360.31 (3684.99-5179.72) | -0.11 (-0.23-0.02) | 9063.86 (7785.4-10170.46) | 201.38 (172.97-225.96) | 21040.07 (18996.64-22598.03) | 223.07 (201.41-239.59) | 0.44 (-0.07-0.95) | 164617.71 (142817.57-184109.24) | 1964.7 (1704.52-2197.33) | 373428.47 (341534.61-404051.96) | 2171.18 (1985.75-2349.23) | 0.49 (0.04-0.94) |
| Tropical Latin America | 804765.62 (671298.78-952829.58) | 5323.44 (4440.57-6302.87) | 1427040.36 (1208470.95-1697770.6) | 3422.58 (2898.37-4071.89) | -1.55 (-1.65--1.45) | 50310.73 (46570.36-52781.33) | 332.8 (308.06-349.14) | 73885.46 (64616.08-79597.89) | 177.2 (154.97-190.91) | -2.19 (-2.26--2.13) | 894684.69 (842237.97-939904.36) | 3236.09 (3046.39-3399.65) | 1167724.01 (1063372.69-1245519.73) | 1456.88 (1326.69-1553.94) | -2.78 (-2.89--2.66) |
| Western Europe | 3595107.48 (3173829.49-4120272.62) | 3698.8 (3265.37-4239.11) | 3730398.81 (3317524.84-4211706.76) | 2580 (2294.45-2912.87) | -1.12 (-1.18--1.06) | 348168.79 (318404.64-363875.38) | 358.21 (327.59-374.37) | 248419.97 (209741.46-271059.28) | 171.81 (145.06-187.47) | -2.98 (-3.21--2.74) | 4917118.87 (4574120.23-5144596.64) | 2417.02 (2248.42-2528.84) | 3091784.27 (2731064.03-3341374.01) | 961.03 (848.91-1038.61) | -3.51 (-3.74--3.29) |
| Western Sub-Saharan Africa | 468270.35 (406207.16-549392.79) | 3258.83 (2826.91-3823.38) | 942080.39 (822914.75-1092490.26) | 3114.54 (2720.58-3611.8) | -0.19 (-0.21--0.17) | 25182.89 (20600.36-32768.68) | 175.25 (143.36-228.05) | 46472.25 (39947.89-53303.38) | 153.64 (132.07-176.22) | -0.5 (-0.61--0.4) | 486418.36 (404174.61-620657.72) | 1876.32 (1559.07-2394.14) | 870057.19 (753871.71-995293.89) | 1636.4 (1417.88-1871.95) | -0.54 (-0.61--0.47) |

EAPCs: Estimated annual percentage changes; SDI: socio-demographic index; DALYs: disability-adjusted life years.

Table S7 Peripheral artery disease burden in people older than 55 across regions during 1990-2019.

|  | Prevalence | | | | | Deaths | | | | | DALY | | | | |
| --- | --- | --- | --- | --- | --- | --- | --- | --- | --- | --- | --- | --- | --- | --- | --- |
|  | 1990 | | 2019 | | 1990-2019 | 1990 | | 2019 | | 1990-2019 | 1990 | | 2019 | | 1990-2019 |
| location | Number | Rate | Number | Rate | EAPC | Number | Rate | Number | Rate | EAPC | Number | Rate | Number | Rate | EAPC |
| Global | 56522868.27 (48667435.89-65053648.96) | 8422.50 (7251.96-9693.68) | 97961027.03 (84720164.82-112255993.29) | 6968.32 (6026.45-7985.17) | -0.65 (-0.7--0.61) | 29221.76 (15600.75-50857.42) | 4.35 (2.32-7.58) | 72332.30 (40082.24-125656.08) | 5.15 (2.85-8.94) | 0.45 (0.36-0.53) | 733083.12 (462817.75-1113370.26) | 58.49 (36.93-88.83) | 1460175.16 (952762.44-2246216.19) | 53.27 (34.76-81.94) | -0.38 (-0.41--0.36) |
| SDI region |  |  |  |  |  |  |  |  |  |  |  |  |  |  |  |
| High SDI | 25174130.34 (21442653.67-29036871.59) | 14334.58 (12209.81-16534.09) | 31021919.18 (27092237.38-35174252.01) | 9961.51 (8699.64-11294.87) | -1.24 (-1.39--1.1) | 14233.65 (7044.04-26412.23) | 8.10 (4.01-15.04) | 34272.82 (16608.56-62803.25) | 11.01 (5.33-20.17) | 0.89 (0.78-1) | 304943.03 (185118.27-481596.75) | 84.18 (51.1-132.94) | 543376.89 (312516.51-897998.64) | 80.94 (46.55-133.76) | -0.15 (-0.19--0.12) |
| High-middle SDI | 15625326.14 (13435754.38-17967886.64) | 8438.88 (7256.34-9704.04) | 26852498.2 (23140664.48-30812464.49) | 7571.05 (6524.5-8687.56) | -0.42 (-0.46--0.37) | 11639.21 (5707.37-20274.6) | 6.29 (3.08-10.95) | 25647.28 (13203.39-45882.79) | 7.23 (3.72-12.94) | 0.34 (0.25-0.43) | 270341.97 (157648.36-432131.96) | 78.25 (45.63-125.07) | 495371.48 (304599.57-808871.56) | 70.77 (43.51-115.55) | -0.45 (-0.49--0.41) |
| Middle SDI | 9588342.38 (8180151.88-11112586.45) | 5568.96 (4751.08-6454.25) | 25388089.15 (21677074.58-29379175.4) | 5921.64 (5056.06-6852.54) | 0.12 (0.04-0.2) | 1643.44 (1208.48-2293.85) | 0.95 (0.7-1.33) | 6298.99 (4747.02-8856.27) | 1.47 (1.11-2.07) | 1.46 (1.41-1.52) | 87348.39 (53644.2-135640.72) | 28.65 (17.6-44.5) | 234452.87 (156412.23-353358.9) | 29.48 (19.67-44.43) | 0.03 (-0.02-0.08) |
| Low-middle SDI | 4547140.63 (3870074.56-5310340.4) | 4593.15 (3909.24-5364.08) | 11138359.26 (9529276.19-12895935) | 4900.28 (4192.37-5673.52) | 0.17 (0.12-0.22) | 813.4 (589.76-1158.38) | 0.82 (0.6-1.17) | 3543.07 (2630.81-4545.58) | 1.56 (1.16-2) | 2.29 (2.22-2.35) | 43099.9 (26999.09-66485.31) | 24.93 (15.62-38.46) | 120373.9 (82919.12-175028.46) | 28.37 (19.55-41.26) | 0.41 (0.39-0.44) |
| Low SDI | 1561128.53 (1324792.38-1826232.31) | 4022.94 (3413.92-4706.1) | 3513729.66 (2999914.22-4089072.24) | 4235.02 (3615.73-4928.47) | 0.15 (0.11-0.19) | 871.22 (462.62-1273.13) | 2.25 (1.19-3.28) | 2513.34 (1389.79-3401.89) | 3.03 (1.68-4.1) | 0.93 (0.82-1.03) | 26901.56 (16396.26-37949.33) | 39.98 (24.37-56.4) | 65621.6 (40891.86-89451.35) | 44.06 (27.45-60.06) | 0.24 (0.15-0.33) |
| Sub region |  |  |  |  |  |  |  |  |  |  |  |  |  |  |  |
| Andean Latin America | 138763.99 (118314.68-160635.48) | 4129.34 (3520.81-4780.19) | 393203.06 (333065.26-453982.02) | 4191.91 (3550.78-4839.87) | 0.10 (0.08-0.12) | 17.16 (12.08-20.96) | 0.51 (0.36-0.62) | 68.28 (50.27-85.74) | 0.73 (0.54-0.91) | 1.31 (1.05-1.56) | 1112.53 (642.31-1788.9) | 17.66 (10.19-28.39) | 3003.44 (1882.85-4776.43) | 16.38 (10.27-26.04) | -0.21 (-0.31--0.12) |
| Australasia | 406432.57 (347375.06-467756.44) | 10316.84 (8817.73-11873.48) | 563187.90 (481893.65-648567.17) | 6839.93 (5852.61-7876.86) | -1.54 (-1.71--1.36) | 450.19 (204.43-780.54) | 11.43 (5.19-19.81) | 1447.43 (692.55-2730.62) | 17.58 (8.41-33.16) | 1.33 (1.15-1.51) | 7506.78 (4089.14-12047.14) | 93.11 (50.72-149.42) | 17865.62 (9737.25-32229.51) | 102.01 (55.6-184.03) | 0.31 (0.24-0.37) |
| Caribbean | 230131.32 (195284.03-268489.2) | 5326.89 (4520.27-6214.76) | 450235.94 (384010.18-522192.14) | 5095.57 (4346.06-5909.94) | -0.16 (-0.17--0.15) | 346.33 (182.04-638.03) | 8.02 (4.21-14.77) | 991.41 (519.72-1749.36) | 11.22 (5.88-19.8) | 1.2 (1.13-1.27) | 6534.18 (3865.68-11038.16) | 76.57 (45.3-129.36) | 15732.85 (9175.89-25907.04) | 89.6 (52.26-147.55) | 0.6 (0.5-0.69) |
| Central Asia | 448604.11 (384987.83-518714.86) | 5626.36 (4828.49-6505.68) | 664215.39 (569453.99-771773.19) | 5102.02 (4374.13-5928.2) | -0.17 (-0.32--0.01) | 42.18 (21.05-72.91) | 0.53 (0.26-0.91) | 93.40 (48.09-168.14) | 0.72 (0.37-1.29) | 1.13 (0.92-1.33) | 3214.28 (1778.02-5216.6) | 22.31 (12.34-36.21) | 4938.87 (2908.54-8038.53) | 22.76 (13.4-37.04) | 0.11 (0.07-0.15) |
| Central Europe | 1865629.44 (1596632-2154938.19) | 7138.96 (6109.62-8246.02) | 2607180.8 (2216509.66-3024148.93) | 7163.63 (6090.2-8309.31) | -0.02 (-0.09-0.05) | 1781.65 (866.68-3363.37) | 6.82 (3.32-12.87) | 3913.52 (1896.02-7215.95) | 10.75 (5.21-19.83) | 1.47 (1.39-1.54) | 39466.96 (22235.3-66529.09) | 79.84 (44.98-134.58) | 69324.29 (38720.95-119750.95) | 90.55 (50.58-156.42) | 0.43 (0.38-0.47) |
| Central Latin America | 801087.64 (681186.83-927385.36) | 5881.86 (5001.51-6809.19) | 2087915.85 (1780143.4-2420050.08) | 5224.62 (4454.47-6055.72) | -0.45 (-0.47--0.42) | 328.76 (157.26-595.05) | 2.41 (1.15-4.37) | 1256.07 (610.56-2474.9) | 3.14 (1.53-6.19) | 0.69 (0.58-0.8) | 9364.42 (5665.35-14853.93) | 36.92 (22.34-58.56) | 27166.39 (16542.6-44443.19) | 35.19 (21.43-57.57) | -0.28 (-0.36--0.2) |
| Central Sub-Saharan Africa | 168769.88 (142532.9-199139.95) | 4445.63 (3754.51-5245.62) | 387348.04 (328202.23-451501.22) | 4527.36 (3836.06-5277.19) | 0.01 (-0.09-0.11) | 321.83 (111.16-598.77) | 8.48 (2.93-15.77) | 733.83 (364.86-1173.35) | 8.58 (4.26-13.71) | -0.16 (-0.3--0.01) | 7566.66 (3144.61-13162.2) | 119.85 (49.81-208.48) | 16361.26 (9090.16-25115.33) | 113.54 (63.08-174.29) | -0.34 (-0.55--0.12) |
| East Asia | 8865191.43 (7519108.7-10281286.86) | 5912.7 (5014.92-6857.17) | 24630739.22 (20987977.06-28446017.41) | 6762.38 (5762.26-7809.87) | 0.32 (0.21-0.43) | 572.39 (452.84-768.26) | 0.38 (0.3-0.51) | 2160.52 (1780.46-2684.06) | 0.59 (0.49-0.74) | 1.49 (1.44-1.55) | 66739.67 (35959.92-110320.69) | 25.1 (13.53-41.49) | 165074.13 (93645.71-276141) | 23.84 (13.52-39.88) | -0.33 (-0.41--0.25) |
| Eastern Europe | 4152065.5 (3550767.9-4794328.07) | 8494.35 (7264.2-9808.29) | 5406067.89 (4644634.23-6225092.18) | 8733.85 (7503.7-10057.04) | 0.24 (0.17-0.32) | 6245.59 (2920.09-11322.55) | 12.78 (5.97-23.16) | 12027.92 (5800.71-23013.6) | 19.43 (9.37-37.18) | 1.46 (1.39-1.53) | 126345.66 (66386.67-216302.18) | 137.48 (72.24-235.36) | 213373.45 (112410.46-392579.87) | 174.13 (91.73-320.37) | 0.78 (0.71-0.86) |
| Eastern Sub-Saharan Africa | 486990.39 (413208-569748.03) | 4004.17 (3397.51-4684.63) | 1080143.82 (919532.48-1257923.73) | 4189.35 (3566.42-4878.88) | 0.17 (0.14-0.19) | 472.28 (247.46-721.64) | 3.88 (2.03-5.93) | 1494.55 (778.84-2000.52) | 5.8 (3.02-7.76) | 1.32 (1.24-1.39) | 11987.99 (7193.46-16843.95) | 56.1 (33.66-78.82) | 32461.44 (19644.68-42593.19) | 71.06 (43-93.24) | 0.76 (0.68-0.84) |
| High-income Asia Pacific | 3754846.09 (3229036.96-4317526.03) | 10732.6 (9229.66-12340.93) | 5570597.32 (4756943.51-6457231.55) | 8047.44 (6872.01-9328.29) | -1.06 (-1.24--0.88) | 364.74 (205.53-641.94) | 1.04 (0.59-1.83) | 1270.23 (643.51-2090.62) | 1.84 (0.93-3.02) | 1.82 (1.71-1.93) | 22419.95 (12800.29-36725.02) | 33.49 (19.12-54.85) | 38166.52 (22992.27-62311.51) | 23.75 (14.31-38.78) | -1.3 (-1.47--1.12) |
| High-income North America | 9917495.91 (8386059.3-11507837.1) | 17063.22 (14428.36-19799.43) | 12598487.28 (11248421.1-14056793.79) | 11641.1 (10393.63-12988.58) | -1.12 (-1.29--0.95) | 6110.37 (2971.56-11268.75) | 10.51 (5.11-19.39) | 15674.31 (7622.72-29261.6) | 14.48 (7.04-27.04) | 0.81 (0.57-1.05) | 124510.32 (73752.71-196143.14) | 100.59 (59.58-158.45) | 248273 (140707.99-430323.22) | 111.28 (63.07-192.89) | 0.42 (0.35-0.5) |
| North Africa and Middle East | 1434253.06 (1224119.25-1663465.66) | 5003.21 (4270.19-5802.79) | 3633822.02 (3108689.27-4202281.07) | 5092.82 (4356.84-5889.52) | 0.07 (0.01-0.13) | 366.09 (278.89-506.2) | 1.28 (0.97-1.77) | 1265.53 (1031.43-1762.57) | 1.77 (1.45-2.47) | 1.33 (1.17-1.5) | 14801.36 (9674.41-21702.65) | 29.21 (19.09-42.83) | 37750.23 (27129.3-54173.65) | 29.34 (21.09-42.11) | 0.03 (-0.02-0.08) |
| Oceania | 26958.52 (22931.16-31453.56) | 5571.48 (4739.15-6500.47) | 69298.89 (58657.92-81095.93) | 6103.14 (5165.99-7142.1) | 0.24 (0.11-0.36) | 2.11 (1.55-3.62) | 0.44 (0.32-0.75) | 7.89 (6.06-11.51) | 0.69 (0.53-1.01) | 1.69 (1.23-2.15) | 219.93 (122.29-356.57) | 27.02 (15.03-43.82) | 584.11 (347.69-929.97) | 30.7 (18.27-48.87) | 0.5 (0.34-0.65) |
| South Asia | 3613496.44 (3060999.97-4212013.29) | 3874.02 (3281.69-4515.69) | 10051885.76 (8586184.17-11671483.49) | 4280.94 (3656.72-4970.7) | 0.29 (0.24-0.34) | 518.14 (388.74-848.57) | 0.56 (0.42-0.91) | 2844.01 (2060.61-3633.58) | 1.21 (0.88-1.55) | 2.76 (2.67-2.85) | 31293.84 (19201.84-48067.23) | 19.78 (12.14-30.38) | 102004.92 (69590.15-147878.4) | 23.44 (15.99-33.98) | 0.56 (0.53-0.58) |
| Southeast Asia | 2739983.15 (2340607.44-3177480.78) | 6471.58 (5528.29-7504.9) | 7159254.02 (6117749.48-8278710.02) | 6840.11 (5845.03-7909.66) | 0.15 (0.04-0.27) | 209.81 (169.2-259.93) | 0.5 (0.4-0.61) | 851.66 (665.82-1057.76) | 0.81 (0.64-1.01) | 1.68 (1.53-1.83) | 21532.96 (11855.33-35423.47) | 28.85 (15.88-47.46) | 56121.1 (33918.91-89026.58) | 30.08 (18.18-47.71) | 0.11 (0.03-0.18) |
| Southern Latin America | 814194.95 (698194.83-947392.51) | 10282.88 (8817.85-11965.1) | 1151361.58 (976997.82-1329575.19) | 8140.98 (6908.09-9401.08) | -0.76 (-0.81--0.72) | 159.64 (73.04-298.23) | 2.02 (0.92-3.77) | 544.6 (258.46-1020.73) | 3.85 (1.83-7.22) | 2.41 (2.15-2.68) | 6692.33 (3883.66-10823.37) | 43.43 (25.2-70.24) | 12754.33 (7681.18-20758.2) | 44.14 (26.58-71.84) | 0.24 (0.16-0.33) |
| Southern Sub-Saharan Africa | 325279.8 (276998.5-376415.27) | 7226.89 (6154.2-8362.99) | 575655.59 (492564.89-666096.07) | 6103.29 (5222.34-7062.17) | -0.66 (-0.75--0.58) | 259.84 (197.42-301.05) | 5.77 (4.39-6.69) | 822.26 (648.92-928.95) | 8.72 (6.88-9.85) | 1.54 (1.32-1.76) | 6626.42 (4983.15-8524.41) | 79.09 (59.47-101.74) | 18324.09 (14646.67-22027.83) | 106.54 (85.16-128.07) | 1.23 (1.05-1.42) |
| Tropical Latin America | 835614.95 (710388.46-967721.33) | 5527.51 (4699.15-6401.38) | 1954684.67 (1669915.8-2261625.79) | 4688.06 (4005.08-5424.22) | -0.73 (-0.83--0.64) | 677.54 (307.88-1236.48) | 4.48 (2.04-8.18) | 2668.01 (1224.32-4934.11) | 6.4 (2.94-11.83) | 1.23 (1.19-1.27) | 16449.89 (9157.14-26805.75) | 59.5 (33.12-96.96) | 49549.49 (26953.24-84372.86) | 61.82 (33.63-105.27) | 0.09 (0.05-0.14) |
| Western Europe | 14934178.07 (12762689.2-17190697.38) | 15364.91 (13130.79-17686.52) | 15711769.28 (13359856.96-18098316.33) | 10866.48 (9239.86-12517.04) | -1.35 (-1.49--1.21) | 9819.03 (4726.62-18480.22) | 10.1 (4.86-19.01) | 21752.35 (10188.61-40040.36) | 15.04 (7.05-27.69) | 1.24 (1.16-1.33) | 201997.33 (120777.07-319964.45) | 99.29 (59.37-157.28) | 315743.64 (178408.07-533467.31) | 98.14 (55.46-165.82) | -0.15 (-0.22--0.08) |
| Western Sub-Saharan Africa | 562901.06 (477622.37-657424.34) | 3917.39 (3323.91-4575.2) | 1213972.71 (1036181.03-1412423.03) | 4013.43 (3425.64-4669.51) | 0.12 (0.09-0.15) | 156.09 (110.18-216.98) | 1.09 (0.77-1.51) | 444.53 (241.03-595) | 1.47 (0.8-1.97) | 1.06 (0.99-1.12) | 6699.65 (4481.09-9740.68) | 25.84 (17.29-37.57) | 15602.01 (9627.73-22244.55) | 29.34 (18.11-41.84) | 0.43 (0.37-0.49) |

EAPCs: Estimated annual percentage changes; SDI: socio-demographic index; DALYs: disability-adjusted life years.

Table S8 The IHD burden in 204 countries and territories by the year 2019.

|  | 20-54 age group | | | | | | 55+ age group | | | | | |
| --- | --- | --- | --- | --- | --- | --- | --- | --- | --- | --- | --- | --- |
|  | Prevalence | | Death | | DALYs | | Prevalence | | Death | | DALYs | |
| location | Number in 2019 | Rate in 2019 | Number in 2019 | Rate in 2019 | Number in 2019 | Rate in 2019 | Number in 2019 | Rate in 2019 | Number in 2019 | Rate in 2019 | Number in 2019 | Rate in 2019 |
| Afghanistan | 180351.65 (160282.42-202437.55) | 1199.45 (1065.98-1346.34) | 528161.19 (377504.18-741328.77) | 3512.61 (2510.64-4930.31) | 528161.19 (377504.18-741328.77) | 3512.61 (2510.64-4930.31) | 399208.87 (366070.01-436638.22) | 24370.5 (22347.47-26655.44) | 22359.27 (17816.48-26863.39) | 1364.97 (1087.64-1639.93) | 447307.96 (353658.14-542854.69) | 14695.09 (11618.48-17834.01) |
| Albania | 10047.74 (8675.82-11834.58) | 781.93 (675.16-920.98) | 12882.46 (9406.6-17432.15) | 1002.53 (732.03-1356.59) | 12882.46 (9406.6-17432.15) | 1002.53 (732.03-1356.59) | 99490.37 (87644.83-113032.25) | 12928.07 (11388.83-14687.74) | 5723.6 (4523.86-7193.51) | 743.74 (587.84-934.75) | 87965.1 (68664.96-112124.11) | 5786.86 (4517.18-7376.18) |
| Algeria | 240976.11 (213861.86-268693.75) | 1123.42 (997.01-1252.63) | 310486.04 (233057.19-421000.31) | 1447.47 (1086.5-1962.68) | 310486.04 (233057.19-421000.31) | 1447.47 (1086.5-1962.68) | 1205576.29 (1108174.24-1310286.28) | 21459.21 (19725.46-23323.05) | 51676.56 (42526.11-62370.44) | 919.84 (756.96-1110.19) | 852223.51 (687483.81-1054513.56) | 8266.23 (6668.32-10228.36) |
| American Samoa | 261.52 (218.25-318.66) | 1065.09 (888.84-1297.79) | 561.4 (430.44-710.18) | 2286.41 (1753.04-2892.3) | 561.4 (430.44-710.18) | 2286.41 (1753.04-2892.3) | 933.92 (814.22-1088.28) | 11273.03 (9828.22-13136.26) | 51.43 (45.13-58) | 620.76 (544.76-700.12) | 1024.51 (891.35-1166.39) | 6909.71 (6011.63-7866.61) |
| Andorra | 359.24 (321.46-403.35) | 812.99 (727.49-912.82) | 170.02 (122.96-230.98) | 384.76 (278.27-522.72) | 170.02 (122.96-230.98) | 384.76 (278.27-522.72) | 1995.04 (1794.7-2212.84) | 8318.09 (7482.78-9226.16) | 71.81 (56.28-89.27) | 299.39 (234.64-372.2) | 951.1 (742.17-1193.76) | 1985.48 (1549.34-2492.06) |
| Angola | 50498.43 (44630.02-57225.55) | 447.68 (395.65-507.31) | 78305.12 (54785.96-106208.94) | 694.19 (485.69-941.56) | 78305.12 (54785.96-106208.94) | 694.19 (485.69-941.56) | 132089.67 (118460.46-147121.76) | 7327.98 (6571.87-8161.92) | 7493.5 (5784.13-9422.97) | 415.72 (320.89-522.76) | 155113.17 (120194.95-197620.32) | 5233.79 (4055.59-6668.05) |
| Antigua and Barbuda | 621.22 (550.81-697.28) | 1318.98 (1169.49-1480.47) | 245.4 (201.94-294.19) | 521.04 (428.75-624.64) | 245.4 (201.94-294.19) | 521.04 (428.75-624.64) | 2664.1 (2407.46-2939.93) | 15089.53 (13635.92-16651.8) | 72.53 (63.24-81.3) | 410.81 (358.21-460.49) | 1217.34 (1051.33-1378.27) | 3708.98 (3203.18-4199.3) |
| Argentina | 70743.3 (63018.81-79688.24) | 323.02 (287.74-363.86) | 129948.24 (120318.36-139032.88) | 593.35 (549.38-634.83) | 129948.24 (120318.36-139032.88) | 593.35 (549.38-634.83) | 503109.63 (461607.9-549697.86) | 5551.87 (5093.89-6065.98) | 42327.01 (38198.51-45382.47) | 467.08 (421.52-500.8) | 647376.21 (602546.68-685569.79) | 3461.64 (3221.93-3665.87) |
| Armenia | 16458.06 (14512.81-18612.78) | 1111.26 (979.91-1256.75) | 22590.98 (18693.89-26906.18) | 1525.36 (1262.22-1816.72) | 22590.98 (18693.89-26906.18) | 1525.36 (1262.22-1816.72) | 153598.3 (139586.15-169147.1) | 20106.51 (18272.28-22141.9) | 8726.57 (7409.94-10042.43) | 1142.34 (969.98-1314.59) | 135793.13 (115938.92-156816.07) | 9414.12 (8037.69-10871.57) |
| Australia | 131728.88 (119078.64-144235.97) | 1132.51 (1023.76-1240.04) | 45565.66 (42559.78-48360.84) | 391.74 (365.9-415.77) | 45565.66 (42559.78-48360.84) | 391.74 (365.9-415.77) | 1073652.51 (995504.34-1159280.9) | 15526.65 (14396.51-16764.96) | 25583.81 (21729.24-27810.68) | 369.98 (314.24-402.18) | 326859.13 (294543.05-349801.2) | 2216.85 (1997.67-2372.45) |
| Austria | 35396.81 (32094.04-39027.54) | 824.41 (747.49-908.97) | 18424 (17215.6-19663.53) | 429.1 (400.96-457.97) | 18424 (17215.6-19663.53) | 429.1 (400.96-457.97) | 271486.88 (247676.45-296883.66) | 9429.17 (8602.2-10311.24) | 17651.89 (15324.89-19473.02) | 613.08 (532.26-676.33) | 214926.49 (194052.95-231808.05) | 3431.91 (3098.61-3701.47) |
| Azerbaijan | 62039.02 (55080.14-70219.49) | 1116.45 (991.22-1263.67) | 112930.64 (87660.88-140610.52) | 2032.3 (1577.54-2530.42) | 112930.64 (87660.88-140610.52) | 2032.3 (1577.54-2530.42) | 300118.45 (274776.15-329177.82) | 17400.62 (15931.3-19085.46) | 24560.09 (21613.63-27835.79) | 1423.97 (1253.14-1613.9) | 449348.84 (389507.44-515568.78) | 16363.07 (14183.94-18774.48) |
| Bahamas | 2597.19 (2304.14-2933.77) | 1306.12 (1158.75-1475.39) | 2226.36 (1761.42-2857.18) | 1119.63 (885.82-1436.87) | 2226.36 (1761.42-2857.18) | 1119.63 (885.82-1436.87) | 10267.47 (9263.88-11262.97) | 15194.61 (13709.43-16667.83) | 274.76 (228.61-331.71) | 406.61 (338.31-490.89) | 5161.41 (4301.95-6259.78) | 4229.58 (3525.28-5129.65) |
| Bahrain | 15014.22 (13294.14-16885.51) | 1599.99 (1416.69-1799.4) | 8582.33 (6788.7-10664.7) | 914.57 (723.44-1136.48) | 8582.33 (6788.7-10664.7) | 914.57 (723.44-1136.48) | 34497.62 (31261.39-37862.46) | 18799.73 (17036.13-20633.43) | 673.26 (544.58-827.4) | 366.9 (296.77-450.9) | 14506.49 (11670.88-17834.24) | 5510.17 (4433.09-6774.19) |
| Bangladesh | 580490.77 (503795.53-672406.57) | 743.8 (645.53-861.58) | 939337.51 (718734.59-1192243.89) | 1203.6 (920.94-1527.66) | 939337.51 (718734.59-1192243.89) | 1203.6 (920.94-1527.66) | 3705393.92 (3372006.55-4088449.27) | 16959.53 (15433.62-18712.77) | 109168.15 (85348.35-133719.43) | 499.66 (390.64-612.03) | 2122880.38 (1661587.57-2629023.51) | 5105.1 (3995.78-6322.27) |
| Barbados | 2188.04 (1940.46-2462.95) | 1544.18 (1369.46-1738.19) | 772.49 (611.56-964.32) | 545.17 (431.6-680.55) | 772.49 (611.56-964.32) | 545.17 (431.6-680.55) | 15038.47 (13581.43-16613.64) | 17333.82 (15654.39-19149.41) | 324.65 (277.06-372.62) | 374.2 (319.35-429.49) | 5274.85 (4475.94-6130.61) | 3046.53 (2585.11-3540.78) |
| Belarus | 49364.4 (43505.71-56242.37) | 1085.25 (956.45-1236.45) | 103818.43 (78847.8-136864.9) | 2282.38 (1733.42-3008.89) | 103818.43 (78847.8-136864.9) | 2282.38 (1733.42-3008.89) | 530923.47 (483185.8-584354.63) | 18531.61 (16865.35-20396.59) | 52156.92 (42963.33-63452.52) | 1820.51 (1499.61-2214.78) | 806052.86 (658624.39-994709.76) | 14351.88 (11726.9-17710.95) |
| Belgium | 43956.85 (39954.61-48591.55) | 847.74 (770.55-937.12) | 19704.43 (18372.66-21005.58) | 380.01 (354.33-405.11) | 19704.43 (18372.66-21005.58) | 380.01 (354.33-405.11) | 363471.38 (332105.59-399831.03) | 9889.04 (9035.67-10878.29) | 14686.22 (12877.18-15906.55) | 399.57 (350.35-432.77) | 193549.15 (176124.71-206561.64) | 2400.72 (2184.59-2562.12) |
| Belize | 2322.8 (2071.31-2620.28) | 1178.05 (1050.5-1328.91) | 1305.37 (1106.33-1544.15) | 662.04 (561.09-783.14) | 1305.37 (1106.33-1544.15) | 662.04 (561.09-783.14) | 7654.5 (6926.61-8504.66) | 16737.65 (15146-18596.64) | 180.71 (157.82-203.55) | 395.15 (345.1-445.08) | 3279.16 (2888.89-3744.1) | 3929.35 (3461.7-4486.48) |
| Benin | 22963.78 (20046.51-26729.21) | 478.78 (417.96-557.29) | 28604.24 (19615.73-40342.62) | 596.39 (408.98-841.13) | 28604.24 (19615.73-40342.62) | 596.39 (408.98-841.13) | 66999.02 (59367.88-75950.41) | 8735.12 (7740.2-9902.17) | 3778.89 (3104.55-4652.69) | 492.68 (404.76-606.6) | 70193.96 (56091.61-88182.99) | 5052.68 (4037.57-6347.56) |
| Bermuda | 436.93 (386.59-490.35) | 1468.59 (1299.39-1648.15) | 194.78 (156.85-237.12) | 654.67 (527.2-797) | 194.78 (156.85-237.12) | 654.67 (527.2-797) | 3530.21 (3198.73-3907.16) | 15774.69 (14293.45-17459.06) | 105.07 (87.51-124.24) | 469.51 (391.05-555.18) | 1507.81 (1289.32-1775.81) | 3264.49 (2791.46-3844.72) |
| Bhutan | 2617.89 (2289.61-2995.68) | 660.23 (577.44-755.51) | 4306.21 (2947.34-5937.97) | 1086.02 (743.32-1497.55) | 4306.21 (2947.34-5937.97) | 1086.02 (743.32-1497.55) | 15617.15 (14203.4-17154.35) | 16823.2 (15300.28-18479.12) | 566.69 (443.65-688.27) | 610.46 (477.91-741.42) | 10285.17 (8090.93-12583.18) | 5870.63 (4618.19-7182.3) |
| Bolivia (Plurinational State of) | 19061.9 (15747.88-23677.15) | 345.05 (285.06-428.59) | 29667.61 (18793.97-42968.81) | 537.02 (340.2-777.79) | 29667.61 (18793.97-42968.81) | 537.02 (340.2-777.79) | 83721.79 (70819.05-99453.35) | 5610.83 (4746.12-6665.12) | 7037.21 (5026.09-9183.61) | 471.62 (336.84-615.46) | 120883.45 (85461.95-160294.53) | 4323.04 (3056.29-5732.46) |
| Bosnia and Herzegovina | 17734.81 (15282.9-21087.4) | 1127.16 (971.33-1340.24) | 16223.98 (12404.01-21101.06) | 1031.14 (788.35-1341.11) | 16223.98 (12404.01-21101.06) | 1031.14 (788.35-1341.11) | 163916.3 (144402.98-185833.19) | 15423.54 (13587.46-17485.79) | 8436.91 (6937.94-10180.02) | 793.86 (652.82-957.88) | 130936.93 (107369.63-159386.24) | 6237.85 (5115.1-7593.18) |
| Botswana | 6673.43 (5791.93-7742.15) | 558.02 (484.31-647.38) | 11667.6 (7154.47-18214.48) | 975.62 (598.24-1523.05) | 11667.6 (7154.47-18214.48) | 975.62 (598.24-1523.05) | 19399.47 (17339.53-21837.78) | 8655.72 (7736.61-9743.65) | 1140.68 (861.17-1463.89) | 508.95 (384.24-653.17) | 23232.82 (17395.97-29962.51) | 6041.52 (4523.69-7791.53) |
| Brazil | 674060.3 (557430.84-831014.12) | 603.39 (498.99-743.89) | 966339.71 (921786.34-1013367.6) | 865.03 (825.14-907.12) | 966339.71 (921786.34-1013367.6) | 865.03 (825.14-907.12) | 3327982.89 (2796180.5-3942110.67) | 8166.01 (6861.1-9672.92) | 149095.87 (134713.92-157884.9) | 365.84 (330.55-387.41) | 2744821.75 (2567261.62-2879977.29) | 3502.4 (3275.84-3674.86) |
| Brunei Darussalam | 1275.53 (1145.27-1425.64) | 502.85 (451.5-562.03) | 2719.45 (2294.44-3182.1) | 1072.1 (904.54-1254.49) | 2719.45 (2294.44-3182.1) | 1072.1 (904.54-1254.49) | 2661.35 (2397.43-2955.49) | 5108.93 (4602.3-5673.58) | 200.38 (177.82-224.34) | 384.66 (341.37-430.67) | 3961.15 (3508.47-4477.5) | 4772.91 (4227.46-5395.08) |
| Bulgaria | 35044.87 (30301.2-41337.72) | 1097.56 (948.99-1294.64) | 72284.42 (56330.56-92494.69) | 2263.85 (1764.2-2896.81) | 72284.42 (56330.56-92494.69) | 2263.85 (1764.2-2896.81) | 409687.69 (361510.32-464264.85) | 16818.55 (14840.77-19059.06) | 32742.82 (27271.75-38394.28) | 1344.16 (1119.56-1576.17) | 487178.45 (403346.04-580987.77) | 9219.36 (7632.91-10994.6) |
| Burkina Faso | 40012.96 (34430.18-46955.68) | 473.26 (407.23-555.38) | 62835.27 (45788.62-83692.34) | 743.2 (541.58-989.89) | 62835.27 (45788.62-83692.34) | 743.2 (541.58-989.89) | 121027.36 (106431.33-138824.54) | 8252.24 (7257.01-9465.74) | 8028.16 (6753.55-9376.81) | 547.4 (460.49-639.36) | 152488.47 (125754.42-181073.68) | 5871.04 (4841.74-6971.61) |
| Burundi | 24158.36 (20816.31-28551.7) | 533.59 (459.78-630.63) | 35296.48 (24704.72-49298.74) | 779.6 (545.66-1088.88) | 35296.48 (24704.72-49298.74) | 779.6 (545.66-1088.88) | 65684.72 (57432.24-75732.6) | 8797.19 (7691.93-10142.9) | 3227.53 (2425.36-4218.46) | 432.26 (324.83-564.98) | 67082.77 (50542.71-88685.75) | 5370.32 (4046.2-7099.75) |
| Cabo Verde | 1753.91 (1528.25-2030.62) | 619.97 (540.21-717.78) | 1724.64 (1307.47-2253.04) | 609.63 (462.16-796.4) | 1724.64 (1307.47-2253.04) | 609.63 (462.16-796.4) | 7057.29 (6304.11-7913.58) | 9853.5 (8801.9-11049.07) | 528.99 (448.42-590.87) | 738.59 (626.09-824.98) | 8036.92 (6934.59-9062.19) | 5992 (5170.15-6756.39) |
| Cambodia | 43293.55 (37359.9-50807.44) | 537.19 (463.56-630.42) | 74417.23 (56458.02-98945.06) | 923.37 (700.53-1227.71) | 74417.23 (56458.02-98945.06) | 923.37 (700.53-1227.71) | 139272.78 (121663.63-160600.66) | 6867.68 (5999.36-7919.38) | 9762.06 (7958.16-11579.15) | 481.38 (392.43-570.98) | 186634.77 (150973.7-224600.43) | 5199.36 (4205.9-6257.03) |
| Cameroon | 51613.91 (44816.93-60066.41) | 432.18 (375.27-502.96) | 74138.26 (49402.07-110449.18) | 620.79 (413.66-924.84) | 74138.26 (49402.07-110449.18) | 620.79 (413.66-924.84) | 150666.82 (133403.69-169819.34) | 7898.55 (6993.55-8902.6) | 8928.82 (7120.78-11328.15) | 468.08 (373.3-593.87) | 171921.22 (134399.46-221553.01) | 5151.36 (4027.08-6638.51) |
| Canada | 123470.04 (110883.82-135923.59) | 743.19 (667.43-818.15) | 77818.69 (72623.94-82662.35) | 468.41 (437.14-497.56) | 77818.69 (72623.94-82662.35) | 468.41 (437.14-497.56) | 991674.15 (907514.76-1074325.31) | 8421.57 (7706.87-9123.47) | 46956.49 (41039.45-50852.36) | 398.77 (348.52-431.85) | 657089.36 (603110.06-698215.29) | 2684.76 (2464.21-2852.8) |
| Central African Republic | 9809.44 (8683.16-11178.75) | 457.94 (405.36-521.87) | 28882.89 (19591.6-41299.6) | 1348.37 (914.61-1928.03) | 28882.89 (19591.6-41299.6) | 1348.37 (914.61-1928.03) | 23467.3 (20870.46-26335.26) | 6920.42 (6154.62-7766.17) | 1846.29 (1375.8-2430.82) | 544.46 (405.72-716.84) | 41538.97 (30791.4-54293.97) | 7634.82 (5659.43-9979.18) |
| Chad | 27082.23 (23315.67-31842.58) | 499.76 (430.25-587.6) | 35146.27 (25003.66-47889.03) | 648.57 (461.4-883.71) | 35146.27 (25003.66-47889.03) | 648.57 (461.4-883.71) | 83184.87 (72624.04-94978.09) | 9351.76 (8164.5-10677.57) | 4557.48 (3777.82-5535.48) | 512.36 (424.71-622.31) | 87207.15 (71457.75-106994.12) | 5461.61 (4475.25-6700.82) |
| Chile | 43049.55 (38254.96-48376.31) | 470.82 (418.38-529.08) | 37229.45 (34676.3-39823.91) | 407.17 (379.24-435.54) | 37229.45 (34676.3-39823.91) | 407.17 (379.24-435.54) | 274231.11 (249473.68-300413.8) | 6543.72 (5952.95-7168.49) | 11115.63 (9929.01-11962.96) | 265.24 (236.93-285.46) | 177428.76 (165049.33-188066.13) | 2145.78 (1996.07-2274.42) |
| China | 8782149.46 (7214383.61-10803108.47) | 1139.51 (936.09-1401.74) | 6884645.38 (5727411.2-8190232.04) | 893.31 (743.15-1062.71) | 6884645.38 (5727411.2-8190232.04) | 893.31 (743.15-1062.71) | 36403013.97 (31023325.92-42633033.79) | 10349.29 (8819.86-12120.47) | 1722250.01 (1481980.07-1951681.31) | 489.63 (421.32-554.86) | 27745134.17 (24051159.6-31601247.04) | 4151.48 (3598.75-4728.46) |
| Colombia | 135541.3 (118732.73-157150.62) | 572.51 (501.51-663.79) | 122366.57 (92252.87-160344.22) | 516.86 (389.67-677.28) | 122366.57 (92252.87-160344.22) | 516.86 (389.67-677.28) | 822180.7 (730762.68-928651.39) | 9043.75 (8038.18-10214.89) | 38936.67 (30303.56-48266.16) | 428.29 (333.33-530.91) | 593240.93 (473165.84-740023.31) | 3301.42 (2633.2-4118.28) |
| Comoros | 2269.32 (1947.65-2687.88) | 686.03 (588.79-812.57) | 2459.08 (1522.29-3497.86) | 743.4 (460.2-1057.43) | 2459.08 (1522.29-3497.86) | 743.4 (460.2-1057.43) | 7946.32 (6932.24-9067.86) | 10089.41 (8801.84-11513.44) | 387.61 (295.48-501.21) | 492.15 (375.17-636.39) | 6945.82 (5329.5-9033.01) | 4641.74 (3561.58-6036.56) |
| Congo | 12848.32 (11320.07-14658.08) | 545.31 (480.44-622.12) | 22747.35 (14549.43-33963.7) | 965.44 (617.5-1441.48) | 22747.35 (14549.43-33963.7) | 965.44 (617.5-1441.48) | 34016.76 (30443.96-37970.2) | 8274.24 (7405.19-9235.88) | 2269.22 (1716.42-2974.09) | 551.97 (417.5-723.42) | 45318.54 (34196.43-59839.56) | 6433.83 (4854.84-8495.37) |
| Cook Islands | 88.64 (73.84-108.14) | 1113.13 (927.34-1358.08) | 139.46 (99.19-181.17) | 1751.32 (1245.66-2275.14) | 139.46 (99.19-181.17) | 1751.32 (1245.66-2275.14) | 532.59 (464.99-616.64) | 11909.7 (10398.14-13789.3) | 21.1 (17.96-24.75) | 471.89 (401.61-553.52) | 388.89 (332.4-457.69) | 4526.84 (3869.26-5327.72) |
| Costa Rica | 14884.7 (13003.29-17211.63) | 618.8 (540.58-715.54) | 13124.81 (10047-16978.73) | 545.64 (417.68-705.85) | 13124.81 (10047-16978.73) | 545.64 (417.68-705.85) | 84623.97 (75085.72-95177.36) | 9458.88 (8392.74-10638.49) | 3407.8 (2685.02-4209.37) | 380.91 (300.12-470.5) | 55142.57 (44342.41-68683.56) | 3196.11 (2570.13-3980.96) |
| Cte d'Ivoire | 60487.42 (52900.01-70156.13) | 541.75 (473.8-628.35) | 80624.32 (55105.56-113717.06) | 722.11 (493.55-1018.51) | 80624.32 (55105.56-113717.06) | 722.11 (493.55-1018.51) | 151625.98 (134233.51-172181.21) | 9018.83 (7984.31-10241.47) | 7817.66 (6223.85-9516.78) | 465 (370.2-566.07) | 152760.6 (118110.77-189445.21) | 5343.77 (4131.67-6627.05) |
| Croatia | 21222.51 (18064.38-25746.21) | 1085.28 (923.78-1316.61) | 16055.62 (12435.44-20642.75) | 821.05 (635.93-1055.63) | 16055.62 (12435.44-20642.75) | 821.05 (635.93-1055.63) | 234121.12 (204045.29-266767.16) | 15919.59 (13874.52-18139.43) | 12821.44 (10372.34-15383.29) | 871.82 (705.29-1046.02) | 173628.15 (140947.75-209907.45) | 5519.6 (4480.69-6672.91) |
| Cuba | 81882.19 (72878.14-92136.96) | 1451.37 (1291.77-1633.13) | 51030.98 (40832-63117.28) | 904.53 (723.75-1118.76) | 51030.98 (40832-63117.28) | 904.53 (723.75-1118.76) | 477861.74 (432895.78-526872.76) | 14759.26 (13370.44-16273.02) | 21057.04 (17634.57-24918.39) | 650.37 (544.66-769.63) | 319411.28 (267636.86-381682.22) | 4826.4 (4044.08-5767.34) |
| Cyprus | 4202.48 (3646.16-4865.47) | 596.38 (517.43-690.47) | 4849.05 (4237.83-5546.99) | 688.14 (601.4-787.19) | 4849.05 (4237.83-5546.99) | 688.14 (601.4-787.19) | 25849.39 (22534.3-29784.11) | 7827.38 (6823.55-9018.84) | 1484.35 (1292.94-1779.88) | 449.47 (391.51-538.96) | 23072.11 (20386.41-27009.02) | 3417.5 (3019.69-4000.65) |
| Czechia | 64194.13 (56287.88-74625.88) | 1279.88 (1122.25-1487.86) | 34155.71 (27124.55-42118.1) | 680.98 (540.8-839.73) | 34155.71 (27124.55-42118.1) | 680.98 (540.8-839.73) | 650985.73 (577052.48-733565.26) | 18868.98 (16726.01-21262.57) | 32168.96 (26629.04-37697.68) | 932.43 (771.85-1092.68) | 442353.78 (369009.24-520721.83) | 5886.86 (4910.79-6929.79) |
| Democratic People's Republic of Korea | 132561.27 (108419.26-163081.3) | 937.72 (766.94-1153.62) | 213733.55 (148419.51-307168.98) | 1511.92 (1049.9-2172.87) | 213733.55 (148419.51-307168.98) | 1511.92 (1049.9-2172.87) | 487313.04 (420122.43-568875.38) | 9037.37 (7791.3-10549.97) | 30945.18 (26149.56-36287.58) | 573.89 (484.95-672.96) | 549900.92 (465457.4-649003.21) | 5300.7 (4486.72-6255.98) |
| Democratic Republic of the Congo | 150602.88 (133133.32-170636.93) | 434.82 (384.39-492.67) | 242341.03 (168814.38-342585.12) | 699.69 (487.4-989.12) | 242341.03 (168814.38-342585.12) | 699.69 (487.4-989.12) | 408354.38 (365454.56-455805.37) | 7096.9 (6351.33-7921.56) | 26192.37 (19061.84-35340.46) | 455.2 (331.28-614.19) | 524687.27 (381310.49-706554.49) | 5375.18 (3906.35-7238.33) |
| Denmark | 21397.32 (19230.27-23895.91) | 810.46 (728.38-905.1) | 7891.88 (7157.87-8741.79) | 298.92 (271.12-331.11) | 7891.88 (7157.87-8741.79) | 298.92 (271.12-331.11) | 173601.43 (155754.49-191898.37) | 9336.69 (8376.84-10320.74) | 6981.73 (6097.28-7565.19) | 375.49 (327.93-406.87) | 91456.48 (83294.93-97947.08) | 2235.48 (2035.99-2394.13) |
| Djibouti | 3885.2 (3322.9-4610.67) | 666.23 (569.81-790.63) | 4516.06 (2629.77-7131.75) | 774.41 (450.95-1222.95) | 4516.06 (2629.77-7131.75) | 774.41 (450.95-1222.95) | 9042.42 (7853.49-10474.99) | 9520.97 (8269.11-11029.35) | 394.26 (285.11-532.29) | 415.13 (300.19-560.46) | 8211.59 (5856.56-11292.56) | 5209.06 (3715.14-7163.49) |
| Dominica | 442.65 (394.72-500.36) | 1348.75 (1202.72-1524.62) | 194.35 (148.85-248.92) | 592.2 (453.56-758.47) | 194.35 (148.85-248.92) | 592.2 (453.56-758.47) | 2588.26 (2336.82-2863.15) | 16566.75 (14957.32-18326.24) | 76.68 (64.84-90.76) | 490.82 (415.04-580.96) | 1204.79 (1018.07-1434.44) | 3871.47 (3271.48-4609.42) |
| Dominican Republic | 58691.93 (52219.61-66063) | 1103.19 (981.53-1241.74) | 101476.88 (71737.75-136906.31) | 1907.39 (1348.4-2573.33) | 101476.88 (71737.75-136906.31) | 1907.39 (1348.4-2573.33) | 242897.07 (219510.98-266821.02) | 15696.96 (14185.66-17243.02) | 13004.85 (10409.86-16103.32) | 840.42 (672.73-1040.66) | 225043.35 (176895.32-283193.11) | 7528.89 (5918.09-9474.31) |
| Ecuador | 31443.67 (25719.43-39120.37) | 377.02 (308.38-469.07) | 56804.8 (43731.11-75042.37) | 681.11 (524.35-899.78) | 56804.8 (43731.11-75042.37) | 681.11 (524.35-899.78) | 154299.95 (129663.11-183137.42) | 6024.7 (5062.74-7150.67) | 9430.38 (7671.94-11697.87) | 368.21 (299.55-456.75) | 152048.07 (122756.06-190763.88) | 3072.26 (2480.39-3854.55) |
| Egypt | 597701.2 (534608.18-666679.22) | 1290.09 (1153.91-1438.97) | 1256770.83 (893027.3-1703962.18) | 2712.64 (1927.53-3677.86) | 1256770.83 (893027.3-1703962.18) | 2712.64 (1927.53-3677.86) | 2663731.57 (2465645.5-2878170.02) | 24604.07 (22774.41-26584.77) | 152821.66 (117974.86-193300.46) | 1411.57 (1089.7-1785.46) | 3119878.16 (2383712.59-3988378.6) | 16840.5 (12866.82-21528.5) |
| El Salvador | 14684.36 (12778.8-17053.26) | 492.1 (428.24-571.49) | 23956.29 (17691.64-32513.71) | 802.82 (592.88-1089.59) | 23956.29 (17691.64-32513.71) | 802.82 (592.88-1089.59) | 90114.79 (79793.87-101490.74) | 9191.94 (8139.18-10352.32) | 5942.06 (4639.53-7429.02) | 606.11 (473.24-757.78) | 86890.77 (67593.46-109011.54) | 4302.64 (3347.08-5398.01) |
| Equatorial Guinea | 2107.84 (1870.38-2382.3) | 352.33 (312.64-398.2) | 2159.56 (1277.2-3559.53) | 360.97 (213.48-594.98) | 2159.56 (1277.2-3559.53) | 360.97 (213.48-594.98) | 5844.74 (5265.28-6482.76) | 7800.65 (7027.28-8652.17) | 300.47 (217.86-405.92) | 401.02 (290.77-541.76) | 5465.6 (3909.83-7661.17) | 4114.84 (2943.56-5767.8) |
| Eritrea | 13525.13 (11682.32-15947.01) | 467.9 (404.14-551.68) | 25490.07 (17177.18-37336.32) | 881.82 (594.24-1291.63) | 25490.07 (17177.18-37336.32) | 881.82 (594.24-1291.63) | 29925.36 (26194.55-34348.29) | 7146.6 (6255.63-8202.86) | 1694.2 (1338.72-2125.31) | 404.6 (319.7-507.55) | 36287.93 (28375.51-45710.46) | 5293.03 (4138.91-6667.42) |
| Estonia | 8142.98 (7155.7-9221.23) | 1352.48 (1188.5-1531.57) | 4051.79 (2974.75-5438.68) | 672.97 (494.08-903.32) | 4051.79 (2974.75-5438.68) | 672.97 (494.08-903.32) | 108955.24 (98903.35-119641.57) | 25145.32 (22825.49-27611.58) | 4339.57 (3424.51-5775.14) | 1001.51 (790.33-1332.82) | 56782.1 (45173.66-75660.28) | 5955.71 (4738.13-7935.78) |
| Eswatini | 2725.56 (2353.72-3165.08) | 526.39 (454.57-611.27) | 4779.82 (3015.81-7130.83) | 923.13 (582.44-1377.18) | 4779.82 (3015.81-7130.83) | 923.13 (582.44-1377.18) | 8548.31 (7606.13-9750.89) | 9173.35 (8162.28-10463.86) | 439.1 (336.63-577.29) | 471.2 (361.25-619.5) | 8896.51 (6734.07-11796.72) | 5332.86 (4036.62-7071.34) |
| Ethiopia | 180657.03 (154929.07-213273.92) | 425.15 (364.61-501.91) | 190072.22 (136829.42-250418.66) | 447.31 (322.01-589.33) | 190072.22 (136829.42-250418.66) | 447.31 (322.01-589.33) | 536698.43 (460731.9-626751.71) | 8284.14 (7111.57-9674.14) | 24145.7 (18001.45-30265.98) | 372.7 (277.86-467.17) | 450471.26 (339115.53-562190.93) | 3783.72 (2848.39-4722.1) |
| Fiji | 4514 (3760.9-5498.32) | 1029.72 (857.92-1254.26) | 15773.48 (12094.78-20180.37) | 3598.19 (2759.02-4603.48) | 15773.48 (12094.78-20180.37) | 3598.19 (2759.02-4603.48) | 13949.37 (12020.31-16408.65) | 10651.26 (9178.3-12529.08) | 1208.55 (973.1-1480.98) | 922.8 (743.03-1130.82) | 26342.59 (20893.11-32502.13) | 11893.17 (9432.84-14674.09) |
| Finland | 18775.08 (16880.78-21092.62) | 784.24 (705.11-881.04) | 10495.25 (9416.35-12203.06) | 438.39 (393.32-509.72) | 10495.25 (9416.35-12203.06) | 438.39 (393.32-509.72) | 203085.16 (183915.51-224032.81) | 10313.83 (9340.29-11377.67) | 14562.68 (12607.23-15785.99) | 739.58 (640.27-801.7) | 182142.1 (165332.56-195467.56) | 4126.02 (3745.23-4427.88) |
| France | 208310.91 (186678.33-233831.7) | 725.67 (650.31-814.57) | 95363.63 (88226.45-102893.4) | 332.21 (307.35-358.44) | 95363.63 (88226.45-102893.4) | 332.21 (307.35-358.44) | 1892820.77 (1697797.14-2097892.04) | 8776 (7871.78-9726.8) | 66122.62 (55699.98-73512.8) | 306.58 (258.25-340.84) | 801403.03 (707673.71-870277.98) | 1656.79 (1463.02-1799.18) |
| Gabon | 4068.1 (3586.47-4618.13) | 498.63 (439.6-566.05) | 5861.24 (4063.08-8536.03) | 718.42 (498.02-1046.27) | 5861.24 (4063.08-8536.03) | 718.42 (498.02-1046.27) | 14203.1 (12818.62-15789.7) | 8160.77 (7365.28-9072.39) | 850.36 (666.02-1042.31) | 488.6 (382.68-598.89) | 16030.17 (12483.38-19687.6) | 5274.96 (4107.84-6478.49) |
| Gambia | 5231.03 (4568.42-6040.03) | 573.76 (501.08-662.49) | 6837.03 (4669.07-9748.99) | 749.91 (512.12-1069.31) | 6837.03 (4669.07-9748.99) | 749.91 (512.12-1069.31) | 16493.59 (14517.04-18662.63) | 10981.57 (9665.57-12425.73) | 1048.7 (861.64-1247.23) | 698.23 (573.69-830.41) | 18968.45 (15205.32-23007.66) | 6709.86 (5378.69-8138.68) |
| Georgia | 23040.51 (20451.61-26119.37) | 1366.61 (1213.05-1549.22) | 35088.81 (29168.56-41423.3) | 2081.23 (1730.08-2456.95) | 35088.81 (29168.56-41423.3) | 2081.23 (1730.08-2456.95) | 232317.31 (210681.06-256543.26) | 21978.82 (19931.88-24270.76) | 13098.7 (11094.12-15145.04) | 1239.23 (1049.58-1432.83) | 197876.41 (169458.85-228314.33) | 9181.89 (7863.26-10594.28) |
| Germany | 359953.5 (324043.61-397861.25) | 932.88 (839.81-1031.13) | 193526.01 (180119.21-205862.86) | 501.56 (466.81-533.53) | 193526.01 (180119.21-205862.86) | 501.56 (466.81-533.53) | 3286103.43 (2996028.61-3591239.52) | 10730.89 (9783.64-11727.33) | 179703.98 (159886.29-192863.54) | 586.83 (522.11-629.8) | 2283591.97 (2098939.08-2419671.46) | 3372.69 (3099.97-3573.67) |
| Ghana | 75614.08 (66150.06-87028.94) | 527 (461.04-606.55) | 103388.95 (76346.49-137439.59) | 720.57 (532.1-957.89) | 103388.95 (76346.49-137439.59) | 720.57 (532.1-957.89) | 232239.34 (206811.14-262629.25) | 8954.41 (7973.98-10126.15) | 13518.49 (11439.81-16038.71) | 521.23 (441.08-618.4) | 252109.06 (209880.6-302450.88) | 5477.78 (4560.25-6571.6) |
| Greece | 36179.05 (32441.3-40454.51) | 765.86 (686.73-856.36) | 56334.7 (53469.98-59652.31) | 1192.52 (1131.88-1262.75) | 56334.7 (53469.98-59652.31) | 1192.52 (1131.88-1262.75) | 331676.24 (297195.18-369649.76) | 9109.78 (8162.73-10152.76) | 24815.7 (21699.72-26730.43) | 681.59 (596-734.18) | 319850.66 (290694.06-339079.07) | 3801.67 (3455.12-4030.22) |
| Greenland | 205.22 (182.38-227.37) | 731.52 (650.08-810.45) | 259.03 (194.07-332.84) | 923.3 (691.76-1186.39) | 259.03 (194.07-332.84) | 923.3 (691.76-1186.39) | 932.69 (851.33-1024.57) | 7237.68 (6606.31-7950.6) | 49.64 (41.56-57.87) | 385.22 (322.48-449.11) | 959.87 (793.55-1129.43) | 4348.77 (3595.25-5116.99) |
| Grenada | 753.86 (670.03-854.22) | 1428.66 (1269.78-1618.85) | 404.19 (343.8-471.77) | 765.99 (651.53-894.06) | 404.19 (343.8-471.77) | 765.99 (651.53-894.06) | 3163.15 (2861.55-3493.76) | 16208.36 (14662.96-17902.48) | 95.73 (87.71-103.81) | 490.55 (449.44-531.93) | 1801.22 (1652.63-1949.93) | 5000.98 (4588.44-5413.87) |
| Guam | 828.52 (694.45-1006.46) | 1054.49 (883.85-1280.95) | 2086.79 (1736.32-2499.94) | 2655.93 (2209.88-3181.77) | 2086.79 (1736.32-2499.94) | 2655.93 (2209.88-3181.77) | 3909.3 (3420.49-4485.08) | 11729.41 (10262.8-13456.97) | 272.04 (231.03-317.53) | 816.22 (693.19-952.72) | 4989.74 (4248.3-5863.23) | 7870.89 (6701.34-9248.75) |
| Guatemala | 31056.79 (26957.96-36009.09) | 376.82 (327.09-436.91) | 57661.35 (44703.47-72854.85) | 699.62 (542.4-883.97) | 57661.35 (44703.47-72854.85) | 699.62 (542.4-883.97) | 145328.5 (128003.43-164206.83) | 8039.16 (7080.78-9083.45) | 8159.26 (6679.57-9798.46) | 451.35 (369.49-542.02) | 126469.63 (103318.07-153718.76) | 3565.58 (2912.87-4333.82) |
| Guinea | 21161.03 (18416.47-24587.19) | 448.89 (390.67-521.57) | 33835.2 (24756.44-45291.53) | 717.75 (525.16-960.77) | 33835.2 (24756.44-45291.53) | 717.75 (525.16-960.77) | 77419.51 (68647.5-88137.65) | 8696.75 (7711.37-9900.75) | 5048.96 (4149.31-6175.79) | 567.16 (466.1-693.74) | 92462.55 (74459.1-115203.07) | 5615.06 (4521.75-6996.04) |
| Guinea-Bissau | 3874.77 (3365.42-4495.97) | 492.27 (427.56-571.18) | 8695.61 (6238.13-11626.97) | 1104.72 (792.52-1477.13) | 8695.61 (6238.13-11626.97) | 1104.72 (792.52-1477.13) | 10092.55 (8891.33-11468.65) | 8944.34 (7879.78-10163.89) | 737.67 (590.94-913.24) | 653.75 (523.71-809.35) | 15164.58 (11938.26-18939.16) | 7772.12 (6118.57-9706.66) |
| Guyana | 4513.08 (3997.23-5125.25) | 1185.67 (1050.14-1346.5) | 6995.38 (5225.15-9153.99) | 1837.81 (1372.74-2404.92) | 6995.38 (5225.15-9153.99) | 1837.81 (1372.74-2404.92) | 16343.55 (14775.12-18241.33) | 15076.79 (13629.92-16827.48) | 882.54 (710.24-1073.08) | 814.13 (655.19-989.91) | 17795.96 (14137.33-22079.39) | 9336.16 (7416.76-11583.34) |
| Haiti | 54047.85 (48020.64-60684.16) | 938.29 (833.65-1053.5) | 83599.55 (53761.49-123459.93) | 1451.32 (933.32-2143.3) | 83599.55 (53761.49-123459.93) | 1451.32 (933.32-2143.3) | 155216.42 (140198.63-173079.72) | 13610.43 (12293.56-15176.8) | 9229.06 (6722.77-12603.06) | 809.27 (589.5-1105.12) | 184230.83 (134338.27-255154.09) | 9155.63 (6676.14-12680.27) |
| Honduras | 22885.64 (19913.45-26625.13) | 514.04 (447.28-598.03) | 30247.57 (19815.81-42894.28) | 679.4 (445.09-963.46) | 30247.57 (19815.81-42894.28) | 679.4 (445.09-963.46) | 93965.26 (83241.28-106429.95) | 9402.53 (8329.45-10649.8) | 6906.13 (5676.91-8415.38) | 691.05 (568.05-842.08) | 120665.87 (99545.5-149165.18) | 6428.78 (5303.54-7947.15) |
| Hungary | 55412.7 (47810.61-64964.75) | 1203.26 (1038.19-1410.68) | 56681.46 (45416.56-69212.19) | 1230.81 (986.2-1502.91) | 56681.46 (45416.56-69212.19) | 1230.81 (986.2-1502.91) | 580534.95 (513486.59-651989.96) | 18147.32 (16051.42-20380.98) | 34585.5 (28927.18-40265.74) | 1081.13 (904.25-1258.69) | 492788.71 (416364.19-577430.07) | 7145.73 (6037.53-8373.08) |
| Iceland | 1233.32 (1113.57-1359.98) | 755.75 (682.37-833.37) | 661.99 (594.77-735.45) | 405.65 (364.46-450.67) | 661.99 (594.77-735.45) | 405.65 (364.46-450.67) | 9127.55 (8291-9973.62) | 9796.52 (8898.66-10704.6) | 420.98 (354.46-465.93) | 451.84 (380.44-500.08) | 5361.45 (4712.76-5890.15) | 2757.36 (2423.75-3029.27) |
| India | 5157282.85 (4491195.83-5950167.18) | 748.64 (651.95-863.73) | 12767815.33 (10831801.53-14880011.28) | 1853.39 (1572.36-2160) | 12767815.33 (10831801.53-14880011.28) | 1853.39 (1572.36-2160) | 32380074.33 (28644825.98-36707136.29) | 16925.43 (14972.97-19187.23) | 1230633.17 (1062928.17-1414859.61) | 643.27 (555.6-739.56) | 24292011.3 (20987764.63-27960482.57) | 6833.91 (5904.35-7865.94) |
| Indonesia | 787130.88 (642174.24-974574.3) | 584.37 (476.75-723.53) | 2185873.4 (1776230-2704554.25) | 1622.8 (1318.68-2007.87) | 2185873.4 (1776230-2704554.25) | 1622.8 (1318.68-2007.87) | 2275652.46 (1890620.83-2757957.54) | 6190.2 (5142.85-7502.16) | 195664.23 (165827.59-216599.42) | 532.24 (451.08-589.19) | 3920763.16 (3298069.18-4377612.5) | 6184.29 (5202.11-6904.89) |
| Iran (Islamic Republic of) | 777437.9 (682197.5-889104.81) | 1678.97 (1473.28-1920.12) | 516917.14 (487464.21-593430.56) | 1116.34 (1052.74-1281.58) | 516917.14 (487464.21-593430.56) | 1116.34 (1052.74-1281.58) | 3556529.34 (3205256.3-3942075) | 29311.02 (26416.01-32488.48) | 91167.93 (83154.99-98294.39) | 751.36 (685.32-810.09) | 1497843.31 (1393801.24-1623356.32) | 6700.72 (6235.28-7262.21) |
| Iraq | 244824.73 (217409.82-275550.1) | 1208.94 (1073.57-1360.66) | 307043.21 (226006.77-414775.96) | 1516.17 (1116.02-2048.16) | 307043.21 (226006.77-414775.96) | 1516.17 (1116.02-2048.16) | 895885.21 (822907.66-978434.02) | 24358.7 (22374.47-26603.16) | 39670.72 (32892.03-45670.3) | 1078.63 (894.32-1241.75) | 759160.33 (609965.71-891394.09) | 11626.99 (9341.99-13652.23) |
| Ireland | 18342.17 (16450.24-20592.1) | 788.85 (707.48-885.61) | 9966.66 (8976.58-10862.03) | 428.64 (386.06-467.15) | 9966.66 (8976.58-10862.03) | 428.64 (386.06-467.15) | 116823.01 (104994.95-129553.84) | 9375.96 (8426.66-10397.71) | 5574.86 (4864.99-6036.03) | 447.43 (390.45-484.44) | 77646.88 (69620.23-83112.68) | 2988.39 (2679.47-3198.76) |
| Israel | 25268.07 (22510.03-28448.45) | 617.46 (550.06-695.17) | 9989.3 (9265.39-10729.28) | 244.1 (226.41-262.18) | 9989.3 (9265.39-10729.28) | 244.1 (226.41-262.18) | 162282.51 (144704.91-181127.32) | 8525.64 (7602.18-9515.66) | 5938.98 (5156.11-6432.13) | 312.01 (270.88-337.92) | 78399.73 (71151.85-83881.36) | 1938.11 (1758.94-2073.63) |
| Italy | 256702.62 (224090.59-292171.54) | 936.27 (817.33-1065.64) | 111487.4 (106772.9-116657.14) | 406.63 (389.43-425.48) | 111487.4 (106772.9-116657.14) | 406.63 (389.43-425.48) | 2499808.54 (2215728.77-2825436.54) | 11332.62 (10044.77-12808.82) | 98574.8 (82848.14-108951.75) | 446.88 (375.58-493.92) | 1184234.04 (1034883.82-1279308.38) | 2346.69 (2050.74-2535.09) |
| Jamaica | 18753.21 (16677.11-21059.39) | 1302.42 (1158.23-1462.59) | 7332.71 (5644.01-9518.45) | 509.26 (391.98-661.06) | 7332.71 (5644.01-9518.45) | 509.26 (391.98-661.06) | 90173.25 (81626.66-99513.33) | 17779.6 (16094.45-19621.2) | 1944.01 (1594-2306.34) | 383.3 (314.29-454.74) | 29959.83 (24658.16-35969.02) | 2959.81 (2436.04-3553.47) |
| Japan | 292330.64 (255512.2-335176.7) | 536.93 (469.31-615.63) | 188292.27 (181796.13-195205.59) | 345.84 (333.91-358.54) | 188292.27 (181796.13-195205.59) | 345.84 (333.91-358.54) | 3402905.88 (3021316.2-3801944.09) | 6576.1 (5838.68-7347.23) | 136274.67 (107715.2-152221.4) | 263.35 (208.16-294.17) | 1647233.91 (1389155.72-1793201.07) | 1295.2 (1092.27-1409.97) |
| Jordan | 70713.4 (62948.78-79347.04) | 1249.85 (1112.61-1402.44) | 49411.4 (39832.06-60651.76) | 873.34 (704.02-1072.01) | 49411.4 (39832.06-60651.76) | 873.34 (704.02-1072.01) | 239364.39 (219334.09-262496.52) | 22420.81 (20544.61-24587.55) | 4988.15 (4229.54-5923.34) | 467.23 (396.17-554.83) | 97508.6 (82699.54-115892.68) | 5260.21 (4461.32-6251.96) |
| Kazakhstan | 93250.08 (82514.47-105671.29) | 1026.15 (908.01-1162.84) | 122862.02 (98376.52-159536.13) | 1352.01 (1082.57-1755.58) | 122862.02 (98376.52-159536.13) | 1352.01 (1082.57-1755.58) | 524062.83 (476643.38-577734.04) | 17226.4 (15667.68-18990.61) | 30193.63 (26355.21-33901.52) | 992.49 (866.32-1114.37) | 508308.1 (442053.54-574987.42) | 9465.01 (8231.31-10706.62) |
| Kenya | 124840.9 (106660.22-149644.75) | 576.18 (492.27-690.66) | 103768.22 (77916.9-136087.2) | 478.93 (359.61-628.09) | 103768.22 (77916.9-136087.2) | 478.93 (359.61-628.09) | 343404.74 (293085.46-403475.9) | 9707.28 (8284.87-11405.36) | 11412.86 (8876.71-14299.12) | 322.62 (250.92-404.2) | 226545.19 (179304.85-281185.04) | 3688.4 (2919.27-4577.99) |
| Kiribati | 441.55 (365.14-535.01) | 817.26 (675.83-990.24) | 2589.87 (1990.84-3364.16) | 4793.6 (3684.84-6226.71) | 2589.87 (1990.84-3364.16) | 4793.6 (3684.84-6226.71) | 1098.51 (941.08-1311.07) | 9447.39 (8093.47-11275.47) | 99.88 (79.08-122.9) | 858.96 (680.08-1057.01) | 2431.87 (1917.91-3045.06) | 13202.48 (10412.18-16531.45) |
| Kuwait | 41533.77 (37107.97-46580.39) | 1415.91 (1265.03-1587.95) | 36667.12 (30193.15-44357.75) | 1250 (1029.3-1512.18) | 36667.12 (30193.15-44357.75) | 1250 (1029.3-1512.18) | 94659.05 (87063.03-102988.91) | 23853.72 (21939.55-25952.81) | 1787.77 (1494.54-2128.34) | 450.51 (376.62-536.33) | 34790.79 (28989.79-41550.73) | 5201.88 (4334.52-6212.62) |
| Kyrgyzstan | 26417.84 (23288.16-30083.7) | 843.4 (743.48-960.44) | 38425.1 (32666.07-45006.72) | 1226.74 (1042.88-1436.86) | 38425.1 (32666.07-45006.72) | 1226.74 (1042.88-1436.86) | 127606.51 (116198.4-140503.84) | 16164.35 (14719.25-17798.1) | 10596.46 (9417.73-11743.78) | 1342.29 (1192.98-1487.62) | 171592.48 (152500.21-191666.76) | 12673.68 (11263.54-14156.35) |
| Lao People's Democratic Republic | 17079.36 (14856.38-19819.96) | 490.97 (427.06-569.75) | 47099.48 (33311.39-62434.81) | 1353.93 (957.58-1794.76) | 47099.48 (33311.39-62434.81) | 1353.93 (957.58-1794.76) | 49093.14 (43421.58-55860.27) | 6793.5 (6008.67-7729.93) | 4615.95 (3776.9-5477.58) | 638.75 (522.65-757.99) | 88598.15 (71118.08-107287.22) | 7049.42 (5658.6-8536.44) |
| Latvia | 10255 (8961.44-11985.01) | 1192.37 (1041.97-1393.52) | 11443.27 (8734.52-14849.23) | 1330.53 (1015.58-1726.55) | 11443.27 (8734.52-14849.23) | 1330.53 (1015.58-1726.55) | 137986.07 (123355.2-153883.8) | 20807.18 (18600.96-23204.42) | 8746.42 (7449.25-10229.39) | 1318.89 (1123.29-1542.51) | 122934.04 (105901.37-143690.65) | 8501.25 (7323.39-9936.63) |
| Lebanon | 31639.88 (28226.5-35441.12) | 1224.8 (1092.67-1371.95) | 51024.27 (36415.91-64674.49) | 1975.19 (1409.69-2503.6) | 51024.27 (36415.91-64674.49) | 1975.19 (1409.69-2503.6) | 225658.07 (207689.51-244092.71) | 26052.9 (23978.38-28181.24) | 11098.62 (8070.67-12723.03) | 1281.37 (931.78-1468.91) | 181399.47 (134498-208913.78) | 10294.34 (7632.7-11855.76) |
| Lesotho | 4701.72 (4070-5482.11) | 470.11 (406.94-548.13) | 10453.99 (6863.77-14815.51) | 1045.25 (686.28-1481.34) | 10453.99 (6863.77-14815.51) | 1045.25 (686.28-1481.34) | 16445.74 (14697.38-18586.62) | 7800.89 (6971.57-8816.39) | 1028.33 (768.48-1327.23) | 487.78 (364.52-629.56) | 21224.18 (15649.3-27581.97) | 5686.03 (4192.5-7389.31) |
| Liberia | 12889.23 (11158.73-14985.77) | 619.96 (536.73-720.8) | 14263.13 (9482.45-20355.12) | 686.05 (456.1-979.07) | 14263.13 (9482.45-20355.12) | 686.05 (456.1-979.07) | 30955.78 (27396.29-35199.48) | 9894.25 (8756.55-11250.64) | 1563.78 (1242.56-1981.93) | 499.82 (397.16-633.47) | 28706.78 (22270.39-37294.32) | 5132.23 (3981.52-6667.52) |
| Libya | 49741.85 (44320.88-56041.33) | 1279.31 (1139.89-1441.33) | 68961.45 (52110.55-98990.92) | 1773.62 (1340.23-2545.95) | 68961.45 (52110.55-98990.92) | 1773.62 (1340.23-2545.95) | 184397.78 (169602.77-200970.9) | 23309.68 (21439.44-25404.68) | 6284.2 (4986.12-8253.01) | 794.38 (630.29-1043.26) | 112505.96 (89359.64-147264.97) | 7712.59 (6125.85-10095.41) |
| Lithuania | 13855.48 (12124.52-15997.74) | 1085.71 (950.07-1253.57) | 18481.54 (14742.03-22795.25) | 1448.2 (1155.18-1786.22) | 18481.54 (14742.03-22795.25) | 1448.2 (1155.18-1786.22) | 185302.55 (166830.11-205834.03) | 19221.77 (17305.59-21351.53) | 14196.24 (11821.48-16663.41) | 1472.6 (1226.26-1728.53) | 188231.3 (158736.35-223429.22) | 9041.84 (7625.03-10732.6) |
| Luxembourg | 2090.76 (1853.31-2381.82) | 652.4 (578.3-743.22) | 1033.96 (901.24-1179.24) | 322.64 (281.22-367.97) | 1033.96 (901.24-1179.24) | 322.64 (281.22-367.97) | 12797.55 (11332.68-14449.35) | 7720.7 (6836.95-8717.22) | 592.88 (495.09-675.78) | 357.68 (298.68-407.69) | 7882.04 (6801.46-8919.13) | 2291.03 (1976.94-2592.47) |
| Madagascar | 61963.22 (52962.25-73081.98) | 559.95 (478.61-660.42) | 114506 (76831.3-160267.65) | 1034.76 (694.3-1448.3) | 114506 (76831.3-160267.65) | 1034.76 (694.3-1448.3) | 149168.26 (130298.75-172482.3) | 8510.99 (7434.37-9841.21) | 8152.28 (5938.31-10785.83) | 465.14 (338.82-615.4) | 168438.77 (121292.07-225558.28) | 5861.84 (4221.09-7849.66) |
| Malawi | 39525.45 (33667.41-47294.61) | 554.7 (472.49-663.74) | 45577.09 (32428.01-61283.33) | 639.63 (455.1-860.05) | 45577.09 (32428.01-61283.33) | 639.63 (455.1-860.05) | 115198.17 (99645.16-133904.22) | 10081.87 (8720.71-11718.98) | 4443.42 (3516.75-5434.72) | 388.88 (307.78-475.63) | 88350.23 (69779.82-108700.81) | 4239.72 (3348.57-5216.29) |
| Malaysia | 123747.18 (106599.06-145035.89) | 761.47 (655.95-892.47) | 207390.49 (160713.24-264289.87) | 1276.17 (988.94-1626.29) | 207390.49 (160713.24-264289.87) | 1276.17 (988.94-1626.29) | 449785.74 (395469.28-516016.16) | 9716.53 (8543.15-11147.28) | 29322.32 (23419.48-35619.11) | 633.44 (505.92-769.46) | 576696.65 (460715.27-707548.62) | 6865 (5484.36-8422.66) |
| Maldives | 1847.85 (1588.1-2156.29) | 596.35 (512.52-695.89) | 2268.78 (1847.21-2726.39) | 732.19 (596.14-879.87) | 2268.78 (1847.21-2726.39) | 732.19 (596.14-879.87) | 3870.08 (3414.18-4416.15) | 8000.41 (7057.96-9129.27) | 258.52 (216.68-301.64) | 534.43 (447.93-623.57) | 4324.1 (3649.07-5072.76) | 5050.41 (4261.99-5924.82) |
| Mali | 33658.1 (29406.22-38697.06) | 434.51 (379.62-499.56) | 39013.52 (27079.22-55009.98) | 503.65 (349.58-710.16) | 39013.52 (27079.22-55009.98) | 503.65 (349.58-710.16) | 111972.57 (100177.95-126341.93) | 8023.58 (7178.42-9053.24) | 6707.23 (5418.72-8173.24) | 480.62 (388.29-585.67) | 119718.61 (96087.35-149726.1) | 4804.12 (3855.84-6008.28) |
| Malta | 1307.71 (1169.32-1471.9) | 647.58 (579.05-728.89) | 1069.13 (949.47-1199.94) | 529.44 (470.18-594.21) | 1069.13 (949.47-1199.94) | 529.44 (470.18-594.21) | 13251.83 (11872.12-14704.62) | 8617.61 (7720.39-9562.35) | 895.41 (771.47-992.04) | 582.28 (501.68-645.12) | 12519.55 (11144.72-13809.71) | 3729.52 (3319.97-4113.85) |
| Marshall Islands | 214.91 (177.77-262.63) | 790.16 (653.6-965.62) | 1245.11 (863.72-1738.24) | 4577.94 (3175.67-6391.05) | 1245.11 (863.72-1738.24) | 4577.94 (3175.67-6391.05) | 512.74 (439.5-606.45) | 8982.2 (7699.19-10623.92) | 52.35 (40.35-68.55) | 917.07 (706.78-1200.86) | 1212.34 (927.26-1597.79) | 13219.41 (10110.9-17422.38) |
| Mauritania | 9227.82 (8054.83-10656) | 573.56 (500.65-662.33) | 7626.9 (4884.58-11154.04) | 474.06 (303.61-693.29) | 7626.9 (4884.58-11154.04) | 474.06 (303.61-693.29) | 34909.16 (31054.56-39493.1) | 10173.87 (9050.49-11509.8) | 1651.69 (1353.29-2001.42) | 481.36 (394.4-583.29) | 28591.82 (22633.66-35746.66) | 4500.34 (3562.53-5626.51) |
| Mauritius | 5353.21 (4612.65-6367.8) | 824.15 (710.14-980.35) | 8278.64 (6557.12-10241.48) | 1274.53 (1009.49-1576.71) | 8278.64 (6557.12-10241.48) | 1274.53 (1009.49-1576.71) | 26562.13 (23185.79-30446.32) | 8327.54 (7269.02-9545.28) | 1472.94 (1225.29-1743.68) | 461.78 (384.14-546.67) | 26104.75 (21649.51-31267.9) | 4437.4 (3680.08-5315.06) |
| Mexico | 399272.04 (344470.43-464728.94) | 643.91 (555.53-749.48) | 503830.16 (423915.51-596821.92) | 812.54 (683.66-962.5) | 503830.16 (423915.51-596821.92) | 812.54 (683.66-962.5) | 1928617.52 (1675553.23-2212529.55) | 9782.4 (8498.79-11222.46) | 95006.16 (81815.41-107292.34) | 481.89 (414.99-544.21) | 1509723.12 (1315935.62-1712142.25) | 3994.55 (3481.81-4530.12) |
| Micronesia (Federated States of) | 367.75 (305.96-447.61) | 778.96 (648.07-948.11) | 2264.67 (1144.22-3338.28) | 4796.92 (2423.63-7071.01) | 2264.67 (1144.22-3338.28) | 4796.92 (2423.63-7071.01) | 1039.28 (894.21-1224.49) | 8411.51 (7237.33-9910.52) | 121.22 (91.71-155.29) | 981.13 (742.22-1256.82) | 2777.97 (2074.29-3606.98) | 14275.09 (10659.12-18535.12) |
| Monaco | 132.74 (118.02-148.93) | 849.18 (755.01-952.76) | 90 (63.18-120.96) | 575.76 (404.19-773.85) | 90 (63.18-120.96) | 575.76 (404.19-773.85) | 1430.7 (1285.75-1585.25) | 9445.1 (8488.19-10465.39) | 77.55 (62.47-89.5) | 511.97 (412.41-590.88) | 954.04 (771.1-1105.42) | 2801.92 (2264.65-3246.49) |
| Mongolia | 19653.39 (17414.09-22204.23) | 1109.72 (983.28-1253.75) | 44709.43 (33773.39-59116.99) | 2524.5 (1907-3338.02) | 44709.43 (33773.39-59116.99) | 2524.5 (1907-3338.02) | 68220.29 (61999.97-75186.93) | 17701.24 (16087.25-19508.89) | 3883.84 (3171.87-4792.11) | 1007.75 (823.01-1243.42) | 74476.6 (59545.97-93984.11) | 11851.89 (9475.89-14956.23) |
| Montenegro | 3284.62 (2839.6-3905.3) | 1108.65 (958.44-1318.14) | 3211.12 (2521.28-4034.88) | 1083.84 (851-1361.88) | 3211.12 (2521.28-4034.88) | 1083.84 (851-1361.88) | 28786.4 (25237.74-32910.2) | 16505.25 (14470.55-18869.71) | 1412.73 (1194.75-1643.23) | 810.02 (685.04-942.18) | 23838.44 (20147.57-27915.93) | 6932.45 (5859.11-8118.22) |
| Morocco | 248567.58 (219388.22-281633.81) | 1384 (1221.53-1568.11) | 351387.01 (250746.84-517937.87) | 1956.49 (1396.13-2883.83) | 351387.01 (250746.84-517937.87) | 1956.49 (1396.13-2883.83) | 1281667.06 (1177992.07-1402515.44) | 23522.72 (21619.95-25740.67) | 63778.16 (51349.15-73299.94) | 1170.53 (942.42-1345.29) | 1193894.6 (935389.91-1391398.71) | 12473.83 (9772.97-14537.36) |
| Mozambique | 60304.3 (51470.77-71321.63) | 559.41 (477.47-661.61) | 70621.09 (47657.88-98497.35) | 655.12 (442.1-913.71) | 70621.09 (47657.88-98497.35) | 655.12 (442.1-913.71) | 168023.21 (146336.67-192610.81) | 9684.02 (8434.12-11101.13) | 7323.2 (5803.31-9275.92) | 422.07 (334.47-534.62) | 149801.03 (118184.35-190525.22) | 4897.51 (3863.85-6228.92) |
| Myanmar | 155037.91 (134402.72-180282.56) | 578.02 (501.09-672.14) | 260455.65 (192150.08-334879.81) | 971.04 (716.38-1248.51) | 260455.65 (192150.08-334879.81) | 971.04 (716.38-1248.51) | 538307.25 (476098.8-609567.13) | 6801.46 (6015.46-7701.82) | 34677.11 (30515.09-39998.64) | 438.14 (385.56-505.38) | 648258.32 (563709.53-752657.19) | 4579.19 (3981.95-5316.64) |
| Namibia | 5799.55 (5033.42-6710.69) | 527.88 (458.15-610.81) | 7336.22 (4780.05-10624.81) | 667.75 (435.08-967.08) | 7336.22 (4780.05-10624.81) | 667.75 (435.08-967.08) | 20576.24 (18302.61-23186.83) | 9080.18 (8076.85-10232.23) | 1203.02 (973.35-1438.06) | 530.89 (429.53-634.61) | 21760.89 (17338.73-26556.72) | 5132.23 (4089.28-6263.31) |
| Nauru | 36.55 (30.25-44.86) | 752.15 (622.41-923.09) | 280.58 (214.27-360.18) | 5774.04 (4409.32-7412.02) | 280.58 (214.27-360.18) | 5774.04 (4409.32-7412.02) | 52.34 (44.93-61.54) | 8115.9 (6966.95-9543) | 6.74 (5.45-8.19) | 1044.88 (844.46-1270.21) | 169.08 (135.62-208.03) | 18647.75 (14957.23-22943.24) |
| Nepal | 85328.39 (74883.67-98271.06) | 604.15 (530.2-695.79) | 157351.55 (112441.74-207210.21) | 1114.1 (796.12-1467.11) | 157351.55 (112441.74-207210.21) | 1114.1 (796.12-1467.11) | 546755.43 (496100.86-604913.19) | 14489.03 (13146.68-16030.21) | 20130.79 (15941.73-24214.05) | 533.47 (422.46-641.67) | 394087.17 (310516.1-479008.9) | 5666.05 (4464.5-6887.03) |
| Netherlands | 83906.22 (75103.52-93399.91) | 1083.94 (970.22-1206.58) | 25784.73 (23841.36-27670.29) | 333.1 (307.99-357.46) | 25784.73 (23841.36-27670.29) | 333.1 (307.99-357.46) | 655987.02 (591443.72-723002.53) | 11618.49 (10475.34-12805.44) | 17043.48 (14949.67-18467.7) | 301.87 (264.78-327.09) | 236702.17 (216097.23-252716.43) | 1954.8 (1784.63-2087.05) |
| New Zealand | 18047.19 (15684.15-20867.41) | 905.75 (787.15-1047.29) | 10622.63 (9896.62-11530.08) | 533.13 (496.69-578.67) | 10622.63 (9896.62-11530.08) | 533.13 (496.69-578.67) | 163890.52 (146091.67-183332.73) | 12426.07 (11076.58-13900.17) | 6239.98 (5415.61-6745.51) | 473.11 (410.61-511.44) | 82138.34 (74768.72-87079.41) | 2966.45 (2700.29-3144.9) |
| Nicaragua | 17393.17 (15111.97-20198.26) | 553.43 (480.84-642.68) | 17073.73 (13374.3-21452.54) | 543.26 (425.55-682.59) | 17073.73 (13374.3-21452.54) | 543.26 (425.55-682.59) | 66214.51 (58397.28-75142.8) | 8963.69 (7905.44-10172.34) | 4703.04 (4037.47-5330.51) | 636.67 (546.57-721.61) | 76112.02 (64525.67-88685.81) | 5604.22 (4751.1-6530.04) |
| Niger | 32878.3 (28644.76-38142.78) | 441.8 (384.91-512.54) | 41367.7 (27494.54-59917.28) | 555.88 (369.46-805.14) | 41367.7 (27494.54-59917.28) | 555.88 (369.46-805.14) | 102009.84 (89871.35-116003.43) | 7873.57 (6936.67-8953.66) | 5802.53 (4579.39-7266.85) | 447.87 (353.46-560.89) | 114374.62 (88878-147093.96) | 5190.1 (4033.12-6674.85) |
| Nigeria | 406710.82 (350177.28-471814.96) | 489.69 (421.62-568.08) | 425111.18 (292567.11-608588.37) | 511.85 (352.26-732.76) | 425111.18 (292567.11-608588.37) | 511.85 (352.26-732.76) | 1169978.58 (1017392-1349446.92) | 8379.58 (7286.73-9664.96) | 59839.46 (43856.81-75487.76) | 428.58 (314.11-540.66) | 1070113.08 (778970.35-1383842.08) | 4399.73 (3202.71-5689.62) |
| Niue | 8.38 (6.94-10.21) | 1123.12 (930.6-1368.5) | 23.84 (15.92-33.61) | 3195.05 (2134.23-4504.65) | 23.84 (15.92-33.61) | 3195.05 (2134.23-4504.65) | 44.45 (38.72-51.51) | 11495.2 (10012.36-13321.82) | 3.86 (3.13-4.46) | 998.22 (809.91-1154.47) | 70.93 (57.67-81.82) | 9572.01 (7781.76-11040.33) |
| North Macedonia | 10467.5 (8994.95-12343.97) | 940.27 (807.99-1108.83) | 14844.63 (11163.37-19265.16) | 1333.45 (1002.78-1730.54) | 14844.63 (11163.37-19265.16) | 1333.45 (1002.78-1730.54) | 79317.24 (69498.9-90351.56) | 13867.75 (12151.12-15796.98) | 4808.81 (3956.2-5773.97) | 840.77 (691.7-1009.52) | 81675.06 (66572.01-99910.66) | 7501.71 (6114.52-9176.61) |
| Northern Mariana Islands | 270.99 (226.34-329.05) | 1304.03 (1089.17-1583.42) | 537.65 (409.17-690.63) | 2587.2 (1968.98-3323.39) | 537.65 (409.17-690.63) | 2587.2 (1968.98-3323.39) | 919.39 (794.4-1075.76) | 9140.98 (7898.29-10695.77) | 51.52 (44.7-58.27) | 512.22 (444.41-579.37) | 1185.37 (1022.68-1340.81) | 7644.58 (6595.37-8647.03) |
| Norway | 18505.52 (16232.08-21119.43) | 729.24 (639.65-832.25) | 7883.09 (7360.28-8503.28) | 310.65 (290.05-335.09) | 7883.09 (7360.28-8503.28) | 310.65 (290.05-335.09) | 138661.24 (122099.95-156865.83) | 8913.04 (7848.49-10083.22) | 6089.03 (5274.59-6635.33) | 391.4 (339.05-426.51) | 78497.96 (70764.65-84181.52) | 2332.32 (2102.55-2501.19) |
| Oman | 32317.15 (28885.05-36448.4) | 1067.98 (954.56-1204.5) | 27546.29 (21816.59-34440.51) | 910.32 (720.97-1138.15) | 27546.29 (21816.59-34440.51) | 910.32 (720.97-1138.15) | 59894.01 (54693.35-65611.31) | 21761.32 (19871.76-23838.59) | 2812.18 (2536.11-3120.36) | 1021.75 (921.44-1133.72) | 55213.44 (49524.02-61695.65) | 12719.89 (11409.18-14213.24) |
| Pakistan | 777519.28 (673289.28-899836.68) | 823.51 (713.12-953.06) | 2349661.03 (1881817.55-2934604.09) | 2488.65 (1993.13-3108.19) | 2349661.03 (1881817.55-2934604.09) | 2488.65 (1993.13-3108.19) | 3319933.01 (2942482.23-3759517.84) | 18671.71 (16548.88-21143.99) | 131243.22 (109040.92-157543.98) | 738.13 (613.26-886.05) | 2742336.52 (2277760.05-3307711.32) | 8830.03 (7334.14-10650.47) |
| Palau | 118.04 (97.5-144.24) | 1227.5 (1013.95-1500.02) | 478.52 (353.07-632.07) | 4976.33 (3671.78-6573.18) | 478.52 (353.07-632.07) | 4976.33 (3671.78-6573.18) | 372.95 (323.71-437.44) | 9691.66 (8412.19-11367.64) | 29.67 (24.19-36.4) | 771.13 (628.56-945.85) | 662.85 (537.52-823.01) | 10503.21 (8517.33-13041.13) |
| Palestine | 23824.33 (21193.86-26669.7) | 1086.86 (966.86-1216.67) | 23547.13 (19996.23-27540.6) | 1074.22 (912.23-1256.4) | 23547.13 (19996.23-27540.6) | 1074.22 (912.23-1256.4) | 84785.03 (77870.34-92494.05) | 21755.91 (19981.6-23734.06) | 3270.95 (2862.8-3739.74) | 839.33 (734.6-959.62) | 62815.37 (54544.01-72018.86) | 9343 (8112.74-10711.91) |
| Panama | 11836.24 (10337.1-13667.25) | 600.72 (524.64-693.65) | 8348.91 (6104.57-11046) | 423.73 (309.83-560.62) | 8348.91 (6104.57-11046) | 423.73 (309.83-560.62) | 67408.39 (59852.39-76223.67) | 9681.94 (8596.66-10948.09) | 2321.76 (1799.36-2888.28) | 333.48 (258.44-414.85) | 35626.66 (28298.7-44371.68) | 2562.42 (2035.36-3191.4) |
| Papua New Guinea | 35066.27 (28930.24-42981.94) | 781.7 (644.92-958.16) | 100455.51 (70749.48-145165.6) | 2239.38 (1577.16-3236.06) | 100455.51 (70749.48-145165.6) | 2239.38 (1577.16-3236.06) | 73886.58 (63797.61-86168.53) | 9915.37 (8561.46-11563.57) | 4802.81 (3492.25-6480.48) | 644.52 (468.65-869.66) | 109085.52 (78772.37-147818.65) | 8858.26 (6396.69-12003.58) |
| Paraguay | 19735.94 (16161.75-24408.96) | 584.93 (479-723.43) | 24327.14 (18098.98-32125) | 721 (536.41-952.11) | 24327.14 (18098.98-32125) | 721 (536.41-952.11) | 77120.51 (65286.7-91946.66) | 8197.2 (6939.38-9773.09) | 4201.37 (3307.86-5272.72) | 446.57 (351.6-560.44) | 73464.37 (57674.12-93096.87) | 4120.91 (3235.17-5222.18) |
| Peru | 54540.88 (45124.12-67274.58) | 327.17 (270.68-403.56) | 63650.7 (44447.55-87719.32) | 381.82 (266.63-526.2) | 63650.7 (44447.55-87719.32) | 381.82 (266.63-526.2) | 291519.39 (247688.69-344646.76) | 5472.71 (4649.88-6470.07) | 14666.15 (10916.22-18653.29) | 275.33 (204.93-350.18) | 213469.24 (160000.8-272910.74) | 2014.91 (1510.23-2575.97) |
| Philippines | 459675.96 (400037.43-531501.13) | 872.07 (758.93-1008.33) | 960573.18 (735481.2-1184472.59) | 1822.34 (1395.31-2247.11) | 960573.18 (735481.2-1184472.59) | 1822.34 (1395.31-2247.11) | 1339015.92 (1167655.41-1529519.84) | 10203.24 (8897.48-11654.88) | 76229.46 (63282.87-88704.26) | 580.87 (482.21-675.92) | 1474007.2 (1220725.5-1750679.02) | 6369.44 (5274.97-7564.99) |
| Poland | 135452.09 (112746.08-166294.86) | 725.7 (604.05-890.95) | 140989.71 (111928.34-172733.86) | 755.37 (599.67-925.44) | 140989.71 (111928.34-172733.86) | 755.37 (599.67-925.44) | 1355808.87 (1134863.02-1602650.34) | 11240.84 (9409.01-13287.37) | 93893.91 (79119.12-108566.55) | 778.46 (655.97-900.11) | 1325903.07 (1130826.75-1531488.27) | 5311.23 (4529.8-6134.75) |
| Portugal | 30853.64 (26577.02-35838.24) | 630.66 (543.25-732.55) | 21262.69 (19760.88-22935.41) | 434.62 (403.92-468.81) | 21262.69 (19760.88-22935.41) | 434.62 (403.92-468.81) | 273080.82 (237099.64-314304.3) | 7207.64 (6257.96-8295.69) | 12493.2 (10770.34-13709.9) | 329.74 (284.27-361.86) | 162665.74 (147082.14-175434.33) | 1906 (1723.4-2055.61) |
| Puerto Rico | 24377.36 (21674.41-27360.24) | 1532.49 (1362.56-1720) | 10009.32 (7481.37-13135.54) | 629.24 (470.32-825.77) | 10009.32 (7481.37-13135.54) | 629.24 (470.32-825.77) | 227979.18 (207370.09-250909.82) | 19383.99 (17631.7-21333.67) | 4620.51 (3603.49-5656.79) | 392.86 (306.39-480.97) | 69352.24 (55528.23-85110.87) | 2685.77 (2150.41-3296.04) |
| Qatar | 19565.93 (17344.79-21968.86) | 900 (797.83-1010.53) | 10380.83 (7642.64-13892.81) | 477.5 (351.55-639.04) | 10380.83 (7642.64-13892.81) | 477.5 (351.55-639.04) | 26609.58 (24028.43-29323.85) | 15397.33 (13903.77-16967.91) | 605.88 (466.42-769.36) | 350.58 (269.89-445.18) | 13789.69 (10451.63-17533.81) | 6066 (4597.6-7713.01) |
| Republic of Korea | 127127.23 (112785.04-142246.77) | 456.75 (405.22-511.07) | 67180.38 (58189.19-80370.4) | 241.37 (209.06-288.76) | 67180.38 (58189.19-80370.4) | 241.37 (209.06-288.76) | 711811.07 (645458.92-787899.44) | 4439.74 (4025.88-4914.32) | 26745.32 (22546.76-30855.44) | 166.82 (140.63-192.45) | 373044.77 (327090.32-419716.23) | 1211.34 (1062.12-1362.89) |
| Republic of Moldova | 19187.06 (16880.61-21942.49) | 1023.41 (900.38-1170.38) | 27779.2 (23508.31-32179.96) | 1481.7 (1253.89-1716.42) | 27779.2 (23508.31-32179.96) | 1481.7 (1253.89-1716.42) | 182092.91 (164317.7-201675.65) | 17500.59 (15792.25-19382.64) | 14719.12 (12904.31-16502.47) | 1414.63 (1240.21-1586.02) | 225169.58 (198502.12-252491.07) | 11183.52 (9859.03-12540.5) |
| Romania | 97435.83 (83695.2-116759.39) | 1078.02 (925.99-1291.81) | 131581.6 (105388.06-161684.1) | 1455.8 (1166-1788.85) | 131581.6 (105388.06-161684.1) | 1455.8 (1166-1788.85) | 982475.5 (865092.13-1117995.67) | 15998.4 (14086.96-18205.18) | 66696.97 (56595.54-77635.79) | 1086.08 (921.59-1264.2) | 952413.33 (805646.2-1115043.02) | 7201.78 (6091.98-8431.52) |
| Russian Federation | 723202.71 (628849.99-833734.43) | 1035 (899.97-1193.19) | 1469642.47 (1201520.6-1810357.45) | 2103.26 (1719.54-2590.87) | 1469642.47 (1201520.6-1810357.45) | 2103.26 (1719.54-2590.87) | 7732834.24 (6848381.63-8724969.85) | 18211.56 (16128.59-20548.14) | 528356.02 (457401.73-591890.48) | 1244.33 (1077.22-1393.96) | 8462801.28 (7394836.26-9539798.1) | 10167.29 (8884.22-11461.2) |
| Rwanda | 22330.17 (19216.9-26087.53) | 410.95 (353.66-480.1) | 22643 (14377.53-34053.15) | 416.71 (264.6-626.7) | 22643 (14377.53-34053.15) | 416.71 (264.6-626.7) | 69186.15 (60357.83-79576.08) | 6890.17 (6010.96-7924.89) | 3260.83 (2385.85-4307.64) | 324.74 (237.6-428.99) | 62506.25 (45902.48-83181.93) | 3634.96 (2669.4-4837.33) |
| Saint Kitts and Nevis | 435.97 (387.32-491.51) | 1399.04 (1242.91-1577.27) | 233.22 (131.83-322.15) | 748.4 (423.05-1033.78) | 233.22 (131.83-322.15) | 748.4 (423.05-1033.78) | 1731.32 (1562.52-1922.95) | 14316.92 (12921.04-15901.56) | 49.64 (44.46-55.82) | 410.48 (367.66-461.57) | 952.52 (846.08-1082.34) | 4664.51 (4143.27-5300.22) |
| Saint Lucia | 1366.14 (1213.53-1543.14) | 1475.06 (1310.28-1666.17) | 513.08 (422.71-623.41) | 553.99 (456.41-673.11) | 513.08 (422.71-623.41) | 553.99 (456.41-673.11) | 6259.03 (5668.38-6936.01) | 17037.92 (15430.09-18880.74) | 121.98 (105.25-139.97) | 332.05 (286.51-381.03) | 2022.8 (1741.51-2339.73) | 2838.75 (2443.99-3283.53) |
| Saint Vincent and the Grenadines | 747.32 (663.14-844.54) | 1354.15 (1201.61-1530.31) | 562.61 (472.27-666.69) | 1019.46 (855.75-1208.05) | 562.61 (472.27-666.69) | 1019.46 (855.75-1208.05) | 3871.22 (3496.34-4287.37) | 16271.67 (14695.95-18020.85) | 146.35 (130.73-163.51) | 615.14 (549.5-687.27) | 2376.9 (2109.03-2677.42) | 5253.32 (4661.29-5917.52) |
| Samoa | 828.86 (688.52-1015.1) | 927.65 (770.59-1136.09) | 2454.07 (1686.37-3362.04) | 2746.58 (1887.37-3762.77) | 2454.07 (1686.37-3362.04) | 2746.58 (1887.37-3762.77) | 2738.98 (2377.53-3192.03) | 11146.97 (9675.97-12990.8) | 229.84 (195.72-273.56) | 935.39 (796.51-1113.33) | 4425.17 (3780.41-5258.57) | 9976.33 (8522.75-11855.17) |
| San Marino | 117.34 (104.89-131.53) | 752.52 (672.66-843.47) | 51.02 (30.12-80.35) | 327.21 (193.15-515.3) | 51.02 (30.12-80.35) | 327.21 (193.15-515.3) | 930.08 (839.27-1030.05) | 9203.36 (8304.86-10192.58) | 35.86 (25.11-47.75) | 354.89 (248.48-472.48) | 443.45 (313.41-606.63) | 1977.57 (1397.68-2705.3) |
| Sao Tome and Principe | 558.31 (485.53-643.89) | 612.94 (533.04-706.9) | 689.91 (476.99-926.42) | 757.42 (523.66-1017.07) | 689.91 (476.99-926.42) | 757.42 (523.66-1017.07) | 1609.9 (1426.67-1825.33) | 9472.42 (8394.34-10739.99) | 108.77 (89.32-125.64) | 639.98 (525.53-739.25) | 1895.31 (1525.49-2215.64) | 6291.79 (5064.13-7355.18) |
| Saudi Arabia | 277362.71 (247254.53-311922.37) | 1188.27 (1059.28-1336.33) | 440559.23 (331100.17-581456.5) | 1887.43 (1418.49-2491.06) | 440559.23 (331100.17-581456.5) | 1887.43 (1418.49-2491.06) | 557228.33 (510677.03-606601.56) | 20498 (18785.58-22314.22) | 19682.08 (16492.66-22974.24) | 724.02 (606.69-845.12) | 440861.72 (363352.44-516949.04) | 10396.22 (8568.43-12190.48) |
| Senegal | 35440.75 (30571.53-41040.96) | 579.49 (499.88-671.06) | 36461.57 (24893.49-49806.09) | 596.18 (407.03-814.38) | 36461.57 (24893.49-49806.09) | 596.18 (407.03-814.38) | 124183.23 (109769.22-141095.54) | 10015.81 (8853.27-11379.84) | 6298.19 (5090.22-7646.92) | 507.97 (410.54-616.75) | 113885.67 (90234.58-141534.1) | 5073.67 (4020-6305.42) |
| Serbia | 51588.34 (44680.33-59963.28) | 1283.67 (1111.78-1492.07) | 40110.04 (30427.42-52015.02) | 998.06 (757.12-1294.29) | 40110.04 (30427.42-52015.02) | 998.06 (757.12-1294.29) | 539287.66 (478073.85-606737.22) | 19703.57 (17467.05-22167.93) | 27421.48 (22852.02-32423.89) | 1001.88 (834.93-1184.65) | 412551.69 (341665.04-493735.88) | 7233.74 (5990.8-8657.24) |
| Seychelles | 423.72 (362.58-496.52) | 781.8 (668.98-916.12) | 694.92 (585.18-813.95) | 1282.18 (1079.7-1501.81) | 694.92 (585.18-813.95) | 1282.18 (1079.7-1501.81) | 1529.6 (1340.15-1747.64) | 7976.45 (6988.49-9113.43) | 91.73 (80.88-102.42) | 478.35 (421.75-534.08) | 1669.76 (1478.87-1875.18) | 4858.54 (4303.12-5456.26) |
| Sierra Leone | 19704.26 (17164.56-22827.18) | 571.38 (497.74-661.94) | 26014.25 (17516.36-37748.55) | 754.36 (507.94-1094.63) | 26014.25 (17516.36-37748.55) | 754.36 (507.94-1094.63) | 59223.31 (52130.89-67574.13) | 10449.27 (9197.89-11922.68) | 3327.39 (2547.28-4246.22) | 587.08 (449.44-749.2) | 61815.08 (46575.22-79727.3) | 5949.33 (4482.58-7673.27) |
| Singapore | 18710.81 (17328.45-20271.61) | 577.32 (534.67-625.48) | 13988.04 (12749.08-15732.24) | 431.6 (393.37-485.42) | 13988.04 (12749.08-15732.24) | 431.6 (393.37-485.42) | 86012.87 (81385.72-91088.54) | 6185.44 (5852.68-6550.44) | 3505.76 (3059.28-3792.35) | 252.11 (220-272.72) | 58069.37 (52807.57-62013.74) | 2217.09 (2016.19-2367.68) |
| Slovakia | 24922.6 (21381.77-29333.52) | 917.53 (787.17-1079.91) | 26011.08 (19367.57-34423.88) | 957.6 (713.02-1267.32) | 26011.08 (19367.57-34423.88) | 957.6 (713.02-1267.32) | 229197.41 (201935.8-258898.53) | 14262.44 (12566.01-16110.67) | 17166.88 (13856.66-20688.36) | 1068.26 (862.27-1287.39) | 249453.68 (199857.5-302709.91) | 7706.63 (6174.41-9351.93) |
| Slovenia | 10293.79 (8836.21-12150.28) | 1075 (922.78-1268.88) | 3947.41 (3006.67-5152.95) | 412.24 (313.99-538.13) | 3947.41 (3006.67-5152.95) | 412.24 (313.99-538.13) | 113648.42 (100318.01-129220.25) | 15920.68 (14053.27-18102.1) | 2890 (2244.81-3729.45) | 404.85 (314.47-522.45) | 40190.83 (31931.39-51287.45) | 2616.43 (2078.74-3338.82) |
| Solomon Islands | 2110.07 (1743.53-2567.26) | 746.19 (616.57-907.87) | 22489.97 (17090.97-28679) | 7953.17 (6043.91-10141.81) | 22489.97 (17090.97-28679) | 7953.17 (6043.91-10141.81) | 4438.25 (3833.37-5224.95) | 9433.18 (8147.56-11105.24) | 736.62 (601.71-869.3) | 1565.63 (1278.89-1847.64) | 17399.19 (14085.4-20808.9) | 22326.05 (18073.91-26701.28) |
| Somalia | 36960.7 (31661.18-43814.41) | 491.14 (420.72-582.22) | 71178.47 (48928.35-101521.77) | 945.84 (650.17-1349.05) | 71178.47 (48928.35-101521.77) | 945.84 (650.17-1349.05) | 81439.27 (70856.16-93526.95) | 8000.09 (6960.47-9187.51) | 4785.03 (3542.46-6381.78) | 470.05 (347.99-626.91) | 104640.73 (76693.92-139974.75) | 6064.3 (4444.68-8112.03) |
| South Africa | 179814.29 (156336.26-208766.24) | 632.24 (549.69-734.03) | 167388.43 (146398.57-188211.26) | 588.55 (514.74-661.76) | 167388.43 (146398.57-188211.26) | 588.55 (514.74-661.76) | 746118.77 (655524.46-850790.43) | 9865.27 (8667.42-11249.25) | 26738.4 (24310.6-28752.34) | 353.54 (321.44-380.17) | 481011.41 (436429.38-517528.48) | 3468.15 (3146.71-3731.44) |
| South Sudan | 21321.23 (18347.77-25203.98) | 631.29 (543.25-746.25) | 17466.47 (10717.75-26311.94) | 517.16 (317.34-779.06) | 17466.47 (10717.75-26311.94) | 517.16 (317.34-779.06) | 58877.48 (51514.29-67722.74) | 9660.51 (8452.37-11111.82) | 2093.46 (1494.49-2792.62) | 343.49 (245.21-458.21) | 40400.82 (28764.03-54644.09) | 3824.46 (2722.89-5172.77) |
| Spain | 185247.42 (165836.02-206799.2) | 842.43 (754.15-940.44) | 97166.05 (91134.54-104443.45) | 441.87 (414.44-474.96) | 97166.05 (91134.54-104443.45) | 441.87 (414.44-474.96) | 1343087.24 (1209268.78-1490012) | 8958.23 (8065.67-9938.2) | 51368.57 (44099.13-57394.01) | 342.62 (294.14-382.81) | 645114.85 (582327.41-704089.69) | 1926.86 (1739.32-2103.01) |
| Sri Lanka | 79027.4 (68001.03-93265.43) | 754.66 (649.37-890.63) | 102291.08 (73813.33-138771.25) | 976.82 (704.87-1325.18) | 102291.08 (73813.33-138771.25) | 976.82 (704.87-1325.18) | 379710.07 (332390.02-435112.1) | 8333.32 (7294.81-9549.2) | 21625.79 (16241.09-27428.76) | 474.61 (356.44-601.97) | 385683.73 (285464.66-494030.69) | 4520.34 (3345.74-5790.21) |
| Sudan | 182563.77 (162792.39-204121.71) | 1034.68 (922.63-1156.86) | 357721.55 (228552.58-517272.06) | 2027.39 (1295.33-2931.65) | 357721.55 (228552.58-517272.06) | 2027.39 (1295.33-2931.65) | 696796.02 (640530.2-756034.32) | 23936.94 (22004.05-25971.95) | 35075.76 (27945.24-43521.01) | 1204.95 (960-1495.07) | 650193.25 (516442.65-818874.88) | 12212.58 (9700.35-15380.93) |
| Suriname | 4198.11 (3721.69-4750.17) | 1509.66 (1338.34-1708.18) | 3655.33 (2929.35-4571.92) | 1314.47 (1053.4-1644.08) | 3655.33 (2929.35-4571.92) | 1314.47 (1053.4-1644.08) | 17927.05 (16213.97-19841.03) | 17219.61 (15574.14-19058.06) | 521.61 (439.31-608.06) | 501.02 (421.98-584.07) | 9815.78 (8282.01-11532.52) | 5058.19 (4267.81-5942.84) |
| Sweden | 39655.52 (34625.27-45736.55) | 864.12 (754.51-996.63) | 14903.62 (13621.65-16225.62) | 324.76 (296.82-353.57) | 14903.62 (13621.65-16225.62) | 324.76 (296.82-353.57) | 324398.1 (280694.5-374609.69) | 9940.17 (8601.01-11478.75) | 18631.45 (16204.84-20739.62) | 570.9 (496.55-635.5) | 235369.63 (213708.67-255294.02) | 3168.38 (2876.8-3436.59) |
| Switzerland | 30181.82 (27084.39-33715.57) | 712.62 (639.49-796.05) | 11942 (11037.18-12858.02) | 281.96 (260.6-303.59) | 11942 (11037.18-12858.02) | 281.96 (260.6-303.59) | 240053.01 (216877.33-264139.79) | 8579.8 (7751.47-9440.69) | 12092.69 (10095.97-13793.78) | 432.21 (360.84-493.01) | 139506.06 (122669.1-154846.82) | 2288.16 (2012.01-2539.78) |
| Syrian Arab Republic | 97949.77 (87541.92-109521.29) | 1518.57 (1357.21-1697.97) | 212334.45 (156938.79-292259.69) | 3291.94 (2433.11-4531.06) | 212334.45 (156938.79-292259.69) | 3291.94 (2433.11-4531.06) | 487973.62 (449511.59-531588.23) | 22633.87 (20849.87-24656.87) | 28489.01 (22463.25-36176.66) | 1321.42 (1041.92-1678) | 542047.71 (420361.53-701570.95) | 14470.21 (11221.74-18728.76) |
| Taiwan (Province of China) | 126608.66 (109908.79-146321.74) | 1033.19 (896.92-1194.06) | 57527.87 (45043.41-74480.31) | 469.46 (367.58-607.8) | 57527.87 (45043.41-74480.31) | 469.46 (367.58-607.8) | 704803.69 (636682.64-786174.37) | 9932.9 (8972.86-11079.67) | 15435.6 (12529.43-19071.76) | 217.54 (176.58-268.78) | 244572.85 (198475.57-300956.68) | 1773.87 (1439.53-2182.82) |
| Tajikistan | 39962.3 (35394.44-45439.16) | 888.33 (786.79-1010.08) | 71565.91 (56632.71-90926.57) | 1590.86 (1258.9-2021.23) | 71565.91 (56632.71-90926.57) | 1590.86 (1258.9-2021.23) | 141872.76 (129056.38-156385.29) | 16197.66 (14734.41-17854.56) | 11869.34 (10024.62-14198.96) | 1355.13 (1144.51-1621.1) | 229118 (189428.33-278784.3) | 17101.96 (14139.42-20809.18) |
| Thailand | 261227.45 (225338.85-305540.93) | 705.51 (608.58-825.19) | 263948.38 (190116.61-362339.04) | 712.86 (513.46-978.58) | 263948.38 (190116.61-362339.04) | 712.86 (513.46-978.58) | 1319663.75 (1161701.29-1513940.42) | 7280.1 (6408.68-8351.85) | 45751.73 (34395.89-58067.71) | 252.4 (189.75-320.34) | 759642 (580661.27-972936.36) | 2203.97 (1684.69-2822.81) |
| Timor-Leste | 2832.86 (2436.12-3318.12) | 530.93 (456.57-621.87) | 5409.54 (2267.62-8121.45) | 1013.84 (424.99-1522.1) | 5409.54 (2267.62-8121.45) | 1013.84 (424.99-1522.1) | 10741.61 (9359.2-12346.94) | 7885.3 (6870.48-9063.75) | 897.29 (689.7-1098.6) | 658.69 (506.3-806.47) | 16951.27 (12841.75-21015.67) | 6645.58 (5034.49-8238.99) |
| Togo | 19710.62 (17160.37-22915.29) | 582.97 (507.54-677.76) | 29808.04 (19794.82-41639.39) | 881.62 (585.46-1231.55) | 29808.04 (19794.82-41639.39) | 881.62 (585.46-1231.55) | 50918.21 (45010.82-57825.41) | 8718.42 (7706.93-9901.1) | 3022.66 (2466.83-3745) | 517.55 (422.38-641.23) | 60149.08 (47646.24-76272.87) | 6006.06 (4757.62-7616.07) |
| Tokelau | 5.76 (4.77-7.09) | 975.5 (807.48-1200.45) | 15.11 (9.84-20.86) | 2558.48 (1666.44-3531.6) | 15.11 (9.84-20.86) | 2558.48 (1666.44-3531.6) | 26.8 (23.14-31.21) | 11488.98 (9920.93-13379.85) | 1.96 (1.63-2.38) | 842.05 (698.18-1020.54) | 36.28 (30.04-44.75) | 8169.27 (6762.9-10075.71) |
| Tonga | 422.39 (350.46-515.23) | 985.18 (817.43-1201.73) | 684.08 (498.77-909.07) | 1595.57 (1163.35-2120.32) | 684.08 (498.77-909.07) | 1595.57 (1163.35-2120.32) | 1621.47 (1411.24-1878.13) | 12417.52 (10807.55-14383.06) | 84.77 (69.89-101.04) | 649.15 (535.21-773.81) | 1529.76 (1254.53-1828.14) | 5961.72 (4889.12-7124.56) |
| Trinidad and Tobago | 11413.76 (10177.51-12869.52) | 1650.19 (1471.46-1860.67) | 8500.17 (6095.97-11534.5) | 1228.95 (881.35-1667.65) | 8500.17 (6095.97-11534.5) | 1228.95 (881.35-1667.65) | 64064.16 (58020.75-70943.33) | 19164.2 (17356.37-21222.04) | 1945.59 (1514.87-2456.59) | 582 (453.16-734.87) | 35151.33 (27045.86-45050.27) | 5518.56 (4246.05-7072.64) |
| Tunisia | 72505.54 (64796.59-80911.19) | 1225.28 (1095-1367.33) | 78221.2 (53233.48-107441.19) | 1321.87 (899.6-1815.66) | 78221.2 (53233.48-107441.19) | 1321.87 (899.6-1815.66) | 474266.28 (436692.95-514942.51) | 21535.13 (19829.03-23382.13) | 19669.38 (14920.06-24990.06) | 893.13 (677.48-1134.73) | 329649.69 (248152.93-426230.67) | 8020.47 (6037.63-10370.32) |
| Turkey | 397783.71 (348887.67-455763.4) | 905.88 (794.52-1037.91) | 364809.53 (288342.76-457486.75) | 830.78 (656.65-1041.84) | 364809.53 (288342.76-457486.75) | 830.78 (656.65-1041.84) | 2386082.64 (2152419.56-2651123.55) | 15746 (14204.04-17495.04) | 90570.69 (73028.06-110081.79) | 597.69 (481.92-726.44) | 1478623.56 (1196109.11-1790363.08) | 5106.66 (4130.95-6183.3) |
| Turkmenistan | 26149.74 (23120.84-29825.01) | 1049.44 (927.89-1196.94) | 61256.69 (48149.88-78137.79) | 2458.36 (1932.35-3135.83) | 61256.69 (48149.88-78137.79) | 2458.36 (1932.35-3135.83) | 125262.33 (114795.67-137718.13) | 18542.36 (16993-20386.17) | 9886.66 (8233.25-11924.54) | 1463.5 (1218.75-1765.17) | 179518.23 (146921.74-219448.99) | 15698.93 (12848.36-19190.89) |
| Tuvalu | 47.79 (39.69-58.46) | 879.05 (729.97-1075.26) | 213.01 (152.11-293.93) | 3918.01 (2797.83-5406.29) | 213.01 (152.11-293.93) | 3918.01 (2797.83-5406.29) | 189.27 (163.47-221.74) | 10618.27 (9170.76-12439.6) | 17.84 (14.53-22.7) | 1000.9 (814.9-1273.72) | 360.98 (291.96-463.2) | 11113.94 (8988.95-14261.4) |
| Uganda | 58660.28 (50426.82-69054.99) | 391.69 (336.71-461.09) | 71558.04 (47250.67-101285.88) | 477.81 (315.5-676.31) | 71558.04 (47250.67-101285.88) | 477.81 (315.5-676.31) | 165850.23 (145365.99-190616.62) | 7458.7 (6537.47-8572.51) | 7973.81 (5451.84-10016.02) | 358.6 (245.18-450.45) | 150250.39 (105885.25-189673.87) | 3809.82 (2684.88-4809.47) |
| Ukraine | 247055.5 (213817.11-286320.78) | 1143.11 (989.32-1324.79) | 697511.27 (559933.23-852284.74) | 3227.35 (2590.78-3943.47) | 697511.27 (559933.23-852284.74) | 3227.35 (2590.78-3943.47) | 2895825.49 (2572474.26-3267211.54) | 21497.04 (19096.66-24254.01) | 309742.68 (270541.61-351686.41) | 2299.36 (2008.35-2610.73) | 4882231.66 (4266399.77-5613056.29) | 17954.5 (15689.77-20642.12) |
| United Arab Emirates | 107217.16 (95700.67-120488.8) | 1492.51 (1332.2-1677.26) | 115310.47 (78925.36-167097.8) | 1605.18 (1098.68-2326.08) | 115310.47 (78925.36-167097.8) | 1605.18 (1098.68-2326.08) | 90301.35 (81902.67-99680.28) | 16648.24 (15099.84-18377.37) | 2272.75 (1663.72-3032.4) | 419.01 (306.73-559.06) | 58750.75 (42735.88-78585.05) | 8381.43 (6096.73-11211) |
| United Kingdom | 224352.66 (197480.42-255763.76) | 712.72 (627.35-812.5) | 171083.54 (164474.39-182641.39) | 543.49 (522.5-580.21) | 171083.54 (164474.39-182641.39) | 543.49 (522.5-580.21) | 1750495.92 (1543029.18-1971100.73) | 8664.34 (7637.45-9756.26) | 89383.64 (80392.9-94165.72) | 442.42 (397.92-466.09) | 1247744.48 (1158440.79-1303633.7) | 2814.32 (2612.89-2940.38) |
| United Republic of Tanzania | 114438.23 (99046.56-134022.81) | 516.92 (447.4-605.38) | 109730.08 (69907.21-159916.01) | 495.65 (315.77-722.34) | 109730.08 (69907.21-159916.01) | 495.65 (315.77-722.34) | 355530 (314415.32-402875.07) | 9117.31 (8062.95-10331.44) | 16162.09 (11966.92-20280.31) | 414.46 (306.88-520.07) | 295011.15 (218624.77-372695.81) | 4163.78 (3085.67-5260.22) |
| United States of America | 1089469.5 (950846.43-1244195.42) | 727.52 (634.95-830.84) | 1258736.94 (1209149.29-1340855.61) | 840.55 (807.43-895.38) | 1258736.94 (1209149.29-1340855.61) | 840.55 (807.43-895.38) | 7773264.92 (6952839.73-8679691.41) | 8060.69 (7209.93-9000.63) | 527862.59 (468127.13-563912.4) | 547.38 (485.44-584.76) | 7684490.86 (7131357.17-8081330.08) | 3869.38 (3590.86-4069.2) |
| United States Virgin Islands | 652.4 (579.96-733.15) | 1466.77 (1303.89-1648.32) | 690.67 (535.33-888.54) | 1552.81 (1203.56-1997.67) | 690.67 (535.33-888.54) | 1552.81 (1203.56-1997.67) | 5343.41 (4837.1-5906.64) | 16182.57 (14649.19-17888.3) | 279.04 (249.46-307.43) | 845.09 (755.48-931.05) | 4932.19 (4360.5-5464.46) | 7378.19 (6522.98-8174.43) |
| Uruguay | 7388.75 (6567.83-8364.41) | 463.23 (411.76-524.39) | 7845.95 (7247.94-8399.41) | 491.89 (454.4-526.59) | 7845.95 (7247.94-8399.41) | 491.89 (454.4-526.59) | 65043.01 (59244.21-71620.29) | 7313.58 (6661.55-8053.14) | 3904.72 (3484.86-4182.9) | 439.05 (391.85-470.33) | 57442.82 (53175.56-60850.05) | 2988.21 (2766.22-3165.45) |
| Uzbekistan | 158782.59 (141311.37-179474.43) | 943.74 (839.9-1066.73) | 457972.5 (377514.56-544957.08) | 2722.02 (2243.81-3239.02) | 457972.5 (377514.56-544957.08) | 2722.02 (2243.81-3239.02) | 623126.52 (571688.46-680392.58) | 16820.67 (15432.15-18366.51) | 65029.53 (56921.98-73679.17) | 1755.41 (1536.55-1988.89) | 1320543.81 (1141546.54-1514726.11) | 23904.24 (20664.06-27419.29) |
| Vanuatu | 1109.51 (920.45-1367.89) | 874.47 (725.46-1078.11) | 5417.52 (3736.12-7639.21) | 4269.86 (2944.65-6020.89) | 5417.52 (3736.12-7639.21) | 4269.86 (2944.65-6020.89) | 3369.41 (2911.57-3952.03) | 11911 (10292.53-13970.59) | 338.7 (272.87-434.61) | 1197.32 (964.59-1536.35) | 7179.16 (5780.06-9416.51) | 14067.61 (11326.08-18451.72) |
| Venezuela (Bolivarian Republic of) | 95427.64 (83256.54-110637.8) | 695.02 (606.37-805.8) | 177618.95 (129431.53-240230.74) | 1293.63 (942.67-1749.64) | 177618.95 (129431.53-240230.74) | 1293.63 (942.67-1749.64) | 494661.35 (441079.72-556615.48) | 9815.41 (8752.2-11044.74) | 32048.32 (25164.44-40428.98) | 635.92 (499.33-802.22) | 565224.48 (440105.21-719960.31) | 5936.25 (4622.19-7561.36) |
| Viet Nam | 347296.62 (299631.98-407609.99) | 665.86 (574.48-781.5) | 346822.99 (255420.91-469072.21) | 664.95 (489.71-899.34) | 346822.99 (255420.91-469072.21) | 664.95 (489.71-899.34) | 1156137.94 (1015077.4-1313194.38) | 7162.04 (6288.2-8134.97) | 66673.36 (55115.28-77795.13) | 413.03 (341.43-481.93) | 1162976.25 (939272.74-1398035.53) | 4087.8 (3301.49-4914.02) |
| Yemen | 123131.8 (110449.46-137829.02) | 941.51 (844.53-1053.89) | 292446.99 (198661.65-418203) | 2236.15 (1519.03-3197.72) | 292446.99 (198661.65-418203) | 2236.15 (1519.03-3197.72) | 469103.87 (431499.21-510900.64) | 22231.5 (20449.36-24212.3) | 25681.54 (21289.57-32658.19) | 1217.08 (1008.94-1547.72) | 508467.05 (414492.77-655027.22) | 13514.07 (11016.41-17409.35) |
| Zambia | 31903.05 (27533.04-37081.99) | 429.51 (370.68-499.24) | 49395.36 (35201.43-68612.41) | 665.01 (473.92-923.73) | 49395.36 (35201.43-68612.41) | 665.01 (473.92-923.73) | 82425.34 (72667.12-93521.16) | 8064.31 (7109.59-9149.91) | 3719.48 (2979.73-4590.08) | 363.91 (291.53-449.08) | 74622.53 (59196.93-92938.27) | 4124.88 (3272.21-5137.31) |
| Zimbabwe | 34042.6 (29254.78-39912.89) | 542.44 (466.15-635.98) | 52717.89 (37461.99-72276.86) | 840.02 (596.93-1151.68) | 52717.89 (37461.99-72276.86) | 840.02 (596.93-1151.68) | 101930.88 (90705.71-115147.91) | 9149.45 (8141.86-10335.83) | 7535.74 (6083.33-9141.72) | 676.42 (546.05-820.57) | 151642.82 (121510.17-185298.76) | 7653.85 (6132.97-9352.56) |

Table S9 The IS burden in 204 countries and territories by the year 2019.

|  | 20-54 age group | | | | | | 55+ age group | | | | | |
| --- | --- | --- | --- | --- | --- | --- | --- | --- | --- | --- | --- | --- |
|  | Prevalence | | Death | | DALYs | | Prevalence | | Death | | DALYs | |
| location | Number in 2019 | Rate in 2019 | Number in 2019 | Rate in 2019 | Number in 2019 | Rate in 2019 | Number in 2019 | Rate in 2019 | Number in 2019 | Rate in 2019 | Number in 2019 | Rate in 2019 |
| Afghanistan | 98611.16 (87653.32-110614.98) | 655.83 (582.95-735.66) | 1654.93 (1128.7-2491.05) | 11.01 (7.51-16.57) | 85389.68 (62161.47-121034.42) | 567.9 (413.41-804.96) | 86213.59 (76022.41-97027.83) | 5263.08 (4640.94-5923.26) | 6976.72 (4863.07-9192.72) | 425.91 (296.88-561.19) | 132865.85 (96253.01-174846.24) | 4364.95 (3162.13-5744.1) |
| Albania | 4710.76 (4073.08-5450.97) | 366.6 (316.97-424.2) | 21.2 (14.4-30.32) | 1.65 (1.12-2.36) | 1789.76 (1345.56-2244.13) | 139.28 (104.71-174.64) | 21200.75 (18691.43-23799.97) | 2754.89 (2428.82-3092.64) | 1265.13 (934.24-1658.55) | 164.39 (121.4-215.52) | 21432.99 (16601.86-27212.11) | 1409.99 (1092.17-1790.17) |
| Algeria | 139215.22 (123651.5-155857.33) | 649.01 (576.46-726.6) | 1034.88 (741.46-1388.44) | 4.82 (3.46-6.47) | 64425.76 (50553.66-80533.17) | 300.35 (235.68-375.44) | 316091.43 (280474.64-356328.2) | 5626.42 (4992.44-6342.63) | 17582.34 (14042.59-21523.26) | 312.96 (249.96-383.11) | 303692.1 (247421.42-368467.46) | 2945.69 (2399.89-3573.99) |
| American Samoa | 146.72 (129.51-166.03) | 597.53 (527.44-676.18) | 0.73 (0.53-0.96) | 2.97 (2.17-3.9) | 57.79 (45.38-71.74) | 235.34 (184.81-292.18) | 360.46 (324.2-402.95) | 4351.05 (3913.29-4863.85) | 11.86 (9.93-13.78) | 143.13 (119.84-166.33) | 252.71 (216.4-287.72) | 1704.37 (1459.46-1940.52) |
| Andorra | 145.93 (122.76-172.56) | 330.26 (277.81-390.52) | 0.15 (0.09-0.23) | 0.34 (0.21-0.53) | 24.34 (18.06-31.21) | 55.08 (40.87-70.63) | 508.27 (450.01-571.42) | 2119.18 (1876.27-2382.45) | 24.23 (18.36-30.48) | 101.03 (76.54-127.07) | 326.3 (264.13-399.71) | 681.17 (551.38-834.42) |
| Angola | 53040.23 (45963.03-60603) | 470.21 (407.47-537.26) | 206.48 (138.81-295.15) | 1.83 (1.23-2.62) | 15623.72 (12147.77-19962.61) | 138.51 (107.69-176.97) | 66147.28 (59427.83-74185.78) | 3669.68 (3296.9-4115.63) | 3088.08 (2518.95-3829.19) | 171.32 (139.74-212.43) | 63042.62 (52712.88-76535.48) | 2127.17 (1778.62-2582.44) |
| Antigua and Barbuda | 282.84 (244.89-326.56) | 600.54 (519.95-693.36) | 0.66 (0.51-0.85) | 1.41 (1.08-1.81) | 50.31 (40.86-60.9) | 106.81 (86.75-129.31) | 502.58 (453.32-562.48) | 2846.62 (2567.63-3185.87) | 30.66 (26.3-35.17) | 173.68 (148.98-199.19) | 466.49 (404.59-531.27) | 1421.29 (1232.72-1618.68) |
| Argentina | 63404.87 (53967.36-73547.25) | 289.51 (246.42-335.82) | 126.08 (104.16-150.14) | 0.58 (0.48-0.69) | 13950 (11107.85-17105) | 63.7 (50.72-78.1) | 209256.93 (187459.15-233038.09) | 2309.17 (2068.63-2571.6) | 12676.63 (11028.36-14061.06) | 139.89 (121.7-155.17) | 194648.81 (174158.9-215475.69) | 1040.82 (931.26-1152.19) |
| Armenia | 6703.69 (5710.86-7878.81) | 452.64 (385.6-531.98) | 38.45 (29.42-49.31) | 2.6 (1.99-3.33) | 2775.17 (2261.56-3376.31) | 187.38 (152.7-227.97) | 18801.83 (16494.12-21325.92) | 2461.22 (2159.13-2791.63) | 1737.32 (1459.28-2020.03) | 227.42 (191.02-264.43) | 28447.78 (24504.36-32927.68) | 1972.2 (1698.81-2282.77) |
| Australia | 40463.39 (34489.93-47452.87) | 347.88 (296.52-407.97) | 31.83 (25.82-38.15) | 0.27 (0.22-0.33) | 6792.74 (5054.54-8724.13) | 58.4 (43.46-75) | 159743.61 (141685.87-177680.81) | 2310.14 (2048.99-2569.53) | 8552.99 (6968.37-9609.41) | 123.69 (100.77-138.97) | 106817.57 (92373.71-118186.47) | 724.47 (626.5-801.57) |
| Austria | 15758.05 (13520.37-18471.98) | 367.01 (314.9-430.22) | 14.23 (11.57-17.14) | 0.33 (0.27-0.4) | 2546.9 (1904.43-3271.64) | 59.32 (44.36-76.2) | 83209.4 (74137.29-91762.04) | 2890 (2574.91-3187.04) | 3621.2 (3014.02-4095.03) | 125.77 (104.68-142.23) | 50392.89 (44190.37-55782.8) | 804.67 (705.62-890.73) |
| Azerbaijan | 24828.01 (21507.43-28776.48) | 446.8 (387.05-517.86) | 149.12 (104.95-200.04) | 2.68 (1.89-3.6) | 10445.74 (8190.92-12823.95) | 187.98 (147.4-230.78) | 45613.17 (39981.88-51682.65) | 2644.61 (2318.12-2996.52) | 3467.89 (2721.09-4114.58) | 201.07 (157.77-238.56) | 66123.37 (53857.87-77740.5) | 2407.89 (1961.24-2830.93) |
| Bahamas | 1210.26 (1058.81-1381.05) | 608.64 (532.47-694.53) | 4.42 (3.29-5.92) | 2.22 (1.65-2.98) | 286.74 (229.21-354.21) | 144.2 (115.27-178.13) | 1934.58 (1745.98-2139.45) | 2862.94 (2583.83-3166.12) | 92.2 (76.58-108.96) | 136.45 (113.33-161.25) | 1498.14 (1270.85-1769.97) | 1227.67 (1041.41-1450.43) |
| Bahrain | 5499.88 (4780.95-6356.85) | 586.09 (509.48-677.42) | 11.72 (8.57-15.65) | 1.25 (0.91-1.67) | 1243.13 (984.46-1521.82) | 132.47 (104.91-162.17) | 5315.3 (4721.25-6000.85) | 2896.61 (2572.88-3270.21) | 124.3 (101.29-164.03) | 67.74 (55.2-89.39) | 2843.36 (2383.75-3493.05) | 1080.03 (905.45-1326.81) |
| Bangladesh | 277885.69 (236561.65-324491.46) | 356.06 (303.11-415.78) | 1611.32 (958.35-2673.57) | 2.06 (1.23-3.43) | 106845.39 (76037.64-155378.51) | 136.9 (97.43-199.09) | 435124.84 (377168.91-506655.98) | 1991.56 (1726.3-2318.96) | 59822.54 (44122.14-76753.23) | 273.81 (201.95-351.3) | 938327.87 (701348.53-1225355.44) | 2256.49 (1686.6-2946.73) |
| Barbados | 974.83 (845.16-1119.62) | 687.97 (596.46-790.16) | 2.84 (2.08-3.75) | 2.01 (1.47-2.65) | 197.05 (157.49-243.2) | 139.06 (111.15-171.64) | 2790.26 (2487.23-3127.85) | 3216.15 (2866.86-3605.26) | 199.44 (165.57-233.37) | 229.88 (190.84-268.99) | 2838.66 (2392.33-3289.18) | 1639.49 (1381.71-1899.69) |
| Belarus | 28516.54 (24952.67-32691.87) | 626.92 (548.57-718.71) | 230.5 (140.86-349.26) | 5.07 (3.1-7.68) | 14291.88 (10395.03-19138.96) | 314.2 (228.53-420.76) | 118131.64 (102563.42-133832.79) | 4123.32 (3579.92-4671.36) | 12300.1 (9637.24-15423.42) | 429.33 (336.38-538.35) | 199826.85 (157739.68-249736.07) | 3557.94 (2808.58-4446.59) |
| Belgium | 17045.94 (14462.11-19993.96) | 328.74 (278.91-385.6) | 25.98 (21.52-31.44) | 0.5 (0.42-0.61) | 3202.95 (2513.85-3969.12) | 61.77 (48.48-76.55) | 89682.64 (80053.43-99768.51) | 2440.01 (2178.03-2714.42) | 5806.75 (4898.21-6527.68) | 157.99 (133.27-177.6) | 73406.75 (65026.24-80848.35) | 910.51 (806.56-1002.82) |
| Belize | 1032.15 (899.27-1176.01) | 523.47 (456.08-596.43) | 2.59 (2.06-3.17) | 1.31 (1.05-1.61) | 196.51 (162.98-233.81) | 99.66 (82.66-118.58) | 1151.07 (1036.86-1264.81) | 2516.97 (2267.24-2765.69) | 56.38 (47.33-64.71) | 123.28 (103.49-141.49) | 890.83 (767.92-1016.34) | 1067.46 (920.18-1217.87) |
| Benin | 22601.74 (19556.63-25922.93) | 471.24 (407.75-540.48) | 80.77 (51.94-120.18) | 1.68 (1.08-2.51) | 6557.28 (5024.24-8498.9) | 136.72 (104.75-177.2) | 24849.08 (22067.27-28013.77) | 3239.75 (2877.06-3652.35) | 1334.02 (1092.3-1623.8) | 173.93 (142.41-211.71) | 24495.28 (20362.03-29506.26) | 1763.21 (1465.69-2123.91) |
| Bermuda | 189.08 (163.92-217.44) | 635.52 (550.97-730.84) | 0.3 (0.22-0.4) | 1.02 (0.74-1.36) | 27.46 (21.68-34.23) | 92.29 (72.87-115.07) | 621.91 (557.58-691.76) | 2778.98 (2491.54-3091.12) | 29.75 (24.12-35.66) | 132.92 (107.78-159.33) | 414.86 (350.34-487.6) | 898.21 (758.51-1055.68) |
| Bhutan | 1233.23 (1048.5-1436.01) | 311.02 (264.43-362.16) | 3.29 (2-5.33) | 0.83 (0.5-1.34) | 307.3 (231.84-411.17) | 77.5 (58.47-103.7) | 1641.07 (1430.61-1860.58) | 1767.8 (1541.09-2004.26) | 147.65 (115.47-184.9) | 159.05 (124.39-199.18) | 2477.85 (1997.86-3059.31) | 1414.32 (1140.35-1746.21) |
| Bolivia (Plurinational State of) | 23374.4 (20243.59-26836.27) | 423.11 (366.44-485.77) | 71.07 (42.36-108.93) | 1.29 (0.77-1.97) | 5018.21 (3551.85-6831.68) | 90.84 (64.29-123.66) | 35523.24 (31783.94-39713.85) | 2380.68 (2130.08-2661.53) | 2109.75 (1531.43-2802.4) | 141.39 (102.63-187.81) | 34364.05 (25606.58-45152.28) | 1228.93 (915.74-1614.74) |
| Bosnia and Herzegovina | 12129.16 (10519.04-13828.19) | 770.89 (668.55-878.87) | 103.06 (75.32-139.95) | 6.55 (4.79-8.89) | 6426.45 (5065.17-8245) | 408.44 (321.92-524.02) | 62325.3 (53922.49-71006.23) | 5864.44 (5073.78-6681.26) | 5997.74 (4902.35-7340.65) | 564.35 (461.28-690.71) | 96763.54 (79898.92-118781.09) | 4609.83 (3806.4-5658.75) |
| Botswana | 6580.55 (5743.79-7532.98) | 550.25 (480.28-629.89) | 26.17 (14.35-42.67) | 2.19 (1.2-3.57) | 1947.3 (1358.59-2698) | 162.83 (113.6-225.6) | 9914.35 (8809.31-11162.96) | 4423.62 (3930.57-4980.72) | 602.16 (446.77-788.77) | 268.67 (199.34-351.94) | 11368 (8759.23-14599.03) | 2956.16 (2277.77-3796.37) |
| Brazil | 571549.57 (464774.03-692229.71) | 511.63 (416.05-619.65) | 1753.44 (1620.87-1916.15) | 1.57 (1.45-1.72) | 119134.73 (104203.22-136702.13) | 106.64 (93.28-122.37) | 1395510.46 (1180513.54-1663271.48) | 3424.22 (2896.67-4081.24) | 72142.88 (63141.34-77695.54) | 177.02 (154.93-190.64) | 1141067.9 (1038644.16-1217705.29) | 1456.01 (1325.31-1553.8) |
| Brunei Darussalam | 1175.42 (995.86-1392.91) | 463.39 (392.6-549.13) | 2.16 (1.69-2.7) | 0.85 (0.67-1.07) | 270.6 (210.3-337.25) | 106.68 (82.91-132.95) | 2240.47 (1976.08-2602.45) | 4300.97 (3793.43-4995.86) | 55.93 (49.13-63.86) | 107.37 (94.31-122.59) | 1289.12 (1134.34-1471.89) | 1553.3 (1366.8-1773.52) |
| Bulgaria | 23324.26 (19274.05-28295.73) | 730.49 (603.64-886.19) | 288.09 (209.47-387.59) | 9.02 (6.56-12.14) | 15821.41 (12319.44-20165.15) | 495.51 (385.83-631.55) | 153897.7 (129007.64-181175.54) | 6317.83 (5296.04-7437.64) | 19936.29 (16574.82-23612.72) | 818.43 (680.43-969.35) | 302544.58 (251666.13-358295.47) | 5725.35 (4762.53-6780.38) |
| Burkina Faso | 33235.47 (28610-38436.47) | 393.1 (338.39-454.62) | 119.41 (78.62-181.14) | 1.41 (0.93-2.14) | 9661.53 (7406.51-12812.65) | 114.27 (87.6-151.54) | 32719.28 (29156.73-36816.44) | 2230.96 (1988.05-2510.33) | 1488.73 (1185.79-1877.3) | 101.51 (80.85-128) | 28737.19 (23051.98-35958.09) | 1106.43 (887.54-1384.44) |
| Burundi | 22910.31 (19755.71-26275.63) | 506.03 (436.35-580.36) | 61.93 (41.44-96.91) | 1.37 (0.92-2.14) | 5582.4 (4298.86-7234.95) | 123.3 (94.95-159.8) | 22336.27 (19959.63-24909.69) | 2991.51 (2673.2-3336.17) | 1119.35 (872.32-1475.86) | 149.92 (116.83-197.66) | 21910.34 (17211.51-28695.72) | 1754.03 (1377.87-2297.24) |
| Cabo Verde | 1444.18 (1255.65-1657.9) | 510.49 (443.85-586.03) | 4.46 (3.12-6.39) | 1.58 (1.1-2.26) | 382.31 (294.79-483.07) | 135.14 (104.2-170.75) | 2154.7 (1933.73-2388.41) | 3008.43 (2699.9-3334.73) | 130.41 (104.1-165.23) | 182.08 (145.35-230.69) | 1971.57 (1647.07-2427.73) | 1469.92 (1227.98-1810.02) |
| Cambodia | 28658.43 (24579.85-33389.93) | 355.59 (304.99-414.3) | 154.77 (103.99-219.89) | 1.92 (1.29-2.73) | 11724.87 (8973.76-15034.25) | 145.48 (111.35-186.54) | 71670.54 (64126.8-81786.08) | 3534.15 (3162.16-4032.95) | 4959.1 (3973.48-5938.06) | 244.54 (195.94-292.81) | 89535.21 (73189.5-105830.74) | 2494.32 (2038.95-2948.28) |
| Cameroon | 53278.27 (46269.15-60610.88) | 446.12 (387.43-507.52) | 232.1 (137.92-365.03) | 1.94 (1.15-3.06) | 17522.78 (12670.39-24339.63) | 146.73 (106.09-203.81) | 57655.2 (51515.73-64735.08) | 3022.51 (2700.66-3393.67) | 2584.59 (1949.97-3335.37) | 135.49 (102.23-174.85) | 50073.08 (38453.89-63725.29) | 1500.37 (1152.21-1909.43) |
| Canada | 78827.27 (64224.3-97044.78) | 474.48 (386.58-584.13) | 46.81 (37.68-55.78) | 0.28 (0.23-0.34) | 12386.67 (8872.75-16185.94) | 74.56 (53.41-97.43) | 437278.1 (371998.16-508187.35) | 3713.49 (3159.11-4315.67) | 11938.35 (9726.63-13558.95) | 101.38 (82.6-115.15) | 187980.57 (161090.84-213597.32) | 768.06 (658.19-872.72) |
| Central African Republic | 9812.4 (8583.49-11130.55) | 458.08 (400.71-519.62) | 59.89 (37.71-97.61) | 2.8 (1.76-4.56) | 3796.35 (2796.05-5471.96) | 177.23 (130.53-255.45) | 10554.82 (9401.34-11914.77) | 3112.58 (2772.42-3513.62) | 620.52 (397.52-914.68) | 182.99 (117.23-269.74) | 13077.23 (9115.99-18805.45) | 2403.58 (1675.51-3456.42) |
| Chad | 25174.89 (21932.03-28583.22) | 464.56 (404.72-527.46) | 115.47 (71.86-183.7) | 2.13 (1.33-3.39) | 8528.49 (6330.89-11632.63) | 157.38 (116.83-214.66) | 27236.02 (24023.62-30896.77) | 3061.91 (2700.77-3473.46) | 1292.21 (975.68-1740.07) | 145.27 (109.69-195.62) | 25052.38 (19325.82-33337.72) | 1568.98 (1210.34-2087.87) |
| Chile | 30262.67 (25791.58-35281.36) | 330.97 (282.07-385.86) | 66.33 (56.07-78.6) | 0.73 (0.61-0.86) | 6901.87 (5567.05-8464.7) | 75.48 (60.88-92.58) | 98879.65 (87568.78-112241.93) | 2359.47 (2089.57-2678.32) | 6366.78 (5436.57-7052.57) | 151.92 (129.73-168.29) | 93259.89 (83457.49-101867.85) | 1127.86 (1009.31-1231.96) |
| China | 3989576.86 (3257518.29-4852870.02) | 517.66 (422.67-629.68) | 32878.04 (26548.85-39416.98) | 4.27 (3.44-5.11) | 2090348.88 (1728583.13-2479429.57) | 271.23 (224.29-321.71) | 19931929.81 (16741333.25-23424389.38) | 5666.6 (4759.52-6659.5) | 996260.57 (852973.63-1136995.7) | 283.23 (242.5-323.25) | 19239118.9 (16847254.71-21912099.92) | 2878.73 (2520.84-3278.68) |
| Colombia | 104311.58 (90246.32-120763.59) | 440.6 (381.19-510.09) | 135.76 (92.65-189.83) | 0.57 (0.39-0.8) | 14544.42 (11318.5-18093.31) | 61.43 (47.81-76.42) | 214579.3 (191247.69-237480.75) | 2360.31 (2103.67-2612.22) | 8152.92 (6159.8-10305.08) | 89.68 (67.76-113.35) | 120727.93 (96563.35-147547.53) | 671.86 (537.38-821.11) |
| Comoros | 1989.54 (1744.88-2276.21) | 601.45 (527.49-688.11) | 5.72 (3.31-9.42) | 1.73 (1-2.85) | 500.67 (376.05-676.05) | 151.36 (113.68-204.38) | 3436.83 (3084.63-3842.37) | 4363.73 (3916.55-4878.64) | 175.06 (134.57-218.89) | 222.27 (170.86-277.92) | 3159.42 (2523.94-3884.3) | 2111.37 (1686.69-2595.79) |
| Congo | 12659.06 (11111.15-14286.07) | 537.27 (471.58-606.33) | 57.58 (36.37-87.23) | 2.44 (1.54-3.7) | 4070.2 (2999.33-5454.29) | 172.75 (127.3-231.49) | 17057.56 (15181.53-19172.74) | 4149.08 (3692.76-4663.58) | 890.69 (693.02-1140.51) | 216.65 (168.57-277.42) | 17353.14 (13807.7-21783.99) | 2463.61 (1960.27-3092.65) |
| Cook Islands | 52.18 (45.64-59.68) | 655.32 (573.14-749.46) | 0.2 (0.13-0.28) | 2.57 (1.66-3.53) | 18.31 (14.3-22.91) | 229.89 (179.62-287.65) | 207.88 (187.25-231.67) | 4648.71 (4187.32-5180.67) | 5.67 (4.65-6.78) | 126.89 (104.08-151.54) | 121.6 (102.95-141.03) | 1415.48 (1198.43-1641.66) |
| Costa Rica | 10946.25 (9473.14-12672.68) | 455.07 (393.82-526.84) | 12.72 (9.1-17.18) | 0.53 (0.38-0.71) | 1472.55 (1146.74-1837.42) | 61.22 (47.67-76.39) | 21275.06 (19205.71-23696.85) | 2378.03 (2146.73-2648.73) | 830.24 (622.33-1038.32) | 92.8 (69.56-116.06) | 11657.92 (9248.52-14211.11) | 675.7 (536.05-823.69) |
| Cte d'Ivoire | 58595.03 (51145.49-66700.78) | 524.81 (458.08-597.4) | 236.8 (147.83-359.17) | 2.12 (1.32-3.22) | 18458.79 (13730.51-24700.2) | 165.33 (122.98-221.23) | 57382.49 (50727.9-64897.53) | 3413.16 (3017.34-3860.16) | 2199.11 (1789.05-2738.38) | 130.8 (106.41-162.88) | 44507.96 (36212.93-54339.09) | 1556.95 (1266.78-1900.85) |
| Croatia | 8356.67 (7091.15-9990.29) | 427.34 (362.63-510.89) | 20.3 (14.4-28.08) | 1.04 (0.74-1.44) | 2268.26 (1710.6-2875.37) | 115.99 (87.48-147.04) | 58643.78 (50878.66-66065.07) | 3987.62 (3459.61-4492.24) | 5562.37 (4543.12-6704.16) | 378.23 (308.92-455.86) | 79743.34 (66264.72-94600.7) | 2535.02 (2106.54-3007.33) |
| Cuba | 37378.6 (32202.86-42825.19) | 662.54 (570.8-759.08) | 135.53 (99.69-176.51) | 2.4 (1.77-3.13) | 8436.75 (6673.67-10404.33) | 149.54 (118.29-184.42) | 102606.9 (90730.09-115045.66) | 3169.12 (2802.29-3553.31) | 6921.4 (5639.81-8299.76) | 213.77 (174.19-256.35) | 100718.27 (84083.48-119740.03) | 1521.88 (1270.53-1809.31) |
| Cyprus | 2013.54 (1621.98-2462.73) | 285.75 (230.18-349.49) | 1.99 (1.6-2.45) | 0.28 (0.23-0.35) | 336.72 (251.64-436.44) | 47.79 (35.71-61.94) | 6590.28 (5549.23-7985.15) | 1995.58 (1680.35-2417.96) | 471.94 (400.61-598.9) | 142.91 (121.31-181.35) | 6335.43 (5470.37-7686.78) | 938.42 (810.29-1138.59) |
| Czechia | 24561.39 (21208.44-28783.09) | 489.7 (422.85-573.87) | 59.83 (45.04-78.37) | 1.19 (0.9-1.56) | 6695.65 (5235.32-8373.24) | 133.5 (104.38-166.94) | 134111.9 (118859.05-149428.18) | 3887.27 (3445.16-4331.21) | 8890.56 (7288.87-10523.98) | 257.7 (211.27-305.04) | 132676.96 (112631.11-154373.32) | 1765.67 (1498.9-2054.41) |
| Democratic People's Republic of Korea | 79472.45 (70509.17-89155.39) | 562.18 (498.77-630.67) | 1086.35 (698.51-1628.44) | 7.68 (4.94-11.52) | 59182.11 (42403.42-81942.45) | 418.65 (299.96-579.65) | 323450.84 (284996.81-362763.37) | 5998.5 (5285.36-6727.56) | 17999.59 (15025.92-21529.47) | 333.81 (278.66-399.27) | 370319.04 (314032.26-434485.59) | 3569.64 (3027.07-4188.17) |
| Democratic Republic of the Congo | 132892.58 (113647.28-152842.54) | 383.69 (328.12-441.29) | 491.18 (317.37-757.13) | 1.42 (0.92-2.19) | 37837.34 (28741.66-49772.39) | 109.24 (82.98-143.7) | 159162.72 (141383.8-178348.87) | 2766.13 (2457.15-3099.57) | 9627.49 (6846.17-13001.1) | 167.32 (118.98-225.95) | 181290.23 (134363.94-241256.01) | 1857.24 (1376.5-2471.56) |
| Denmark | 9258.09 (7859.68-10815.91) | 350.67 (297.7-409.67) | 15.46 (12.3-19.43) | 0.59 (0.47-0.74) | 1774.73 (1404.88-2190.76) | 67.22 (53.21-82.98) | 47296.28 (41989.37-53517.47) | 2543.7 (2258.29-2878.29) | 2979.98 (2530.22-3336.66) | 160.27 (136.08-179.45) | 40023.64 (35561.33-43873.59) | 978.3 (869.23-1072.41) |
| Djibouti | 3517.64 (3050.14-4025.53) | 603.2 (523.04-690.29) | 11.94 (6.29-20.45) | 2.05 (1.08-3.51) | 958.88 (691.67-1357.96) | 164.43 (118.61-232.86) | 3810.31 (3408.22-4248.95) | 4011.96 (3588.59-4473.81) | 165.07 (128.1-214.81) | 173.81 (134.88-226.18) | 3432.57 (2692.46-4456.26) | 2177.46 (1707.97-2826.85) |
| Dominica | 201.71 (174.72-232.7) | 614.62 (532.38-709.03) | 0.62 (0.45-0.83) | 1.88 (1.37-2.52) | 42.01 (33.25-52.26) | 127.99 (101.3-159.24) | 497.75 (443.96-562.43) | 3185.94 (2841.64-3599.97) | 39.53 (32.85-46.53) | 252.99 (210.24-297.81) | 562.67 (476.44-657.9) | 1808.08 (1531-2114.09) |
| Dominican Republic | 32664.43 (28903.12-36922.06) | 613.97 (543.27-694) | 186.9 (119.99-279.21) | 3.51 (2.26-5.25) | 10789.83 (7683.39-14966.94) | 202.81 (144.42-281.32) | 56554.09 (50663.44-62657.7) | 3654.75 (3274.07-4049.19) | 3500.74 (2794.74-4417.97) | 226.23 (180.61-285.51) | 56015.93 (44820.42-70542.63) | 1874.03 (1499.48-2360.03) |
| Ecuador | 40112.82 (34686.9-46198.65) | 480.97 (415.91-553.94) | 116.62 (86.91-158.21) | 1.4 (1.04-1.9) | 8569.9 (6784.39-10637.53) | 102.76 (81.35-127.55) | 71298.34 (64639.41-79643.97) | 2783.87 (2523.87-3109.73) | 2883.58 (2338.5-3586.38) | 112.59 (91.31-140.03) | 45081.35 (36956.72-54834.96) | 910.91 (746.74-1107.99) |
| Egypt | 331972.65 (294335.23-371583.23) | 716.54 (635.3-802.03) | 3504.95 (2456.76-5042.62) | 7.57 (5.3-10.88) | 205923.91 (155297.96-273153.07) | 444.47 (335.2-589.58) | 711749.83 (628581.59-809600.58) | 6574.21 (5806.01-7478.03) | 25896.59 (18712.33-36125.08) | 239.2 (172.84-333.68) | 596365.65 (448396.11-799419.73) | 3219.07 (2420.36-4315.11) |
| El Salvador | 12080.69 (10485.66-14014.48) | 404.85 (351.39-469.65) | 22.15 (15.15-32.6) | 0.74 (0.51-1.09) | 1984.33 (1527.95-2551.64) | 66.5 (51.2-85.51) | 23190.58 (20931.28-25713.04) | 2365.5 (2135.04-2622.8) | 1062.82 (806.85-1335.79) | 108.41 (82.3-136.25) | 15298.2 (11984.02-19084.04) | 757.53 (593.42-945) |
| Equatorial Guinea | 2504.54 (2135.01-2888.48) | 418.64 (356.87-482.81) | 5.8 (2.89-10.51) | 0.97 (0.48-1.76) | 568.66 (408.44-796.43) | 95.05 (68.27-133.12) | 2955.5 (2656.52-3325.58) | 3944.55 (3545.5-4438.46) | 138.43 (101.07-181.97) | 184.75 (134.9-242.86) | 2571.88 (1962.7-3383.62) | 1936.27 (1477.65-2547.4) |
| Eritrea | 13498.2 (11688.14-15523.98) | 466.96 (404.35-537.05) | 48.18 (29.02-80.27) | 1.67 (1-2.78) | 3795.65 (2810.86-5203.96) | 131.31 (97.24-180.03) | 13003.46 (11564.73-14532.59) | 3105.41 (2761.82-3470.59) | 635.34 (482.62-846.79) | 151.73 (115.26-202.22) | 12943.44 (10131.44-16737.26) | 1887.96 (1477.79-2441.33) |
| Estonia | 2893.2 (2497.07-3393.53) | 480.54 (414.74-563.64) | 7.5 (5.17-10.36) | 1.25 (0.86-1.72) | 803.68 (621.05-1001.89) | 133.48 (103.15-166.41) | 11242.77 (9772.06-12651.22) | 2594.67 (2255.25-2919.72) | 921.41 (709.95-1228.41) | 212.65 (163.85-283.5) | 14115.97 (11400.1-17917.25) | 1480.58 (1195.72-1879.29) |
| Eswatini | 2547.64 (2199.78-2900.08) | 492.03 (424.84-560.09) | 8.68 (4.93-13.85) | 1.68 (0.95-2.67) | 689.82 (504.88-932.18) | 133.23 (97.51-180.03) | 3885.99 (3452.22-4387.92) | 4170.12 (3704.64-4708.76) | 242.49 (185.35-315.75) | 260.22 (198.91-338.84) | 4584.63 (3575.56-5883.66) | 2748.18 (2143.31-3526.86) |
| Ethiopia | 178020.66 (143931.28-215766.52) | 418.95 (338.72-507.78) | 236.99 (172.43-355.85) | 0.56 (0.41-0.84) | 32828.33 (24974.31-41445.76) | 77.26 (58.77-97.54) | 150099.18 (125553.19-182355.9) | 2316.84 (1937.96-2814.73) | 8041.64 (6476.39-10095.46) | 124.13 (99.97-155.83) | 140444.47 (115618.63-175955.06) | 1179.66 (971.13-1477.93) |
| Fiji | 2621.19 (2296.45-3006.37) | 597.94 (523.86-685.8) | 17.88 (13.23-24.12) | 4.08 (3.02-5.5) | 1248.36 (979.73-1573.47) | 284.77 (223.49-358.93) | 5680.84 (5090.2-6346.3) | 4337.7 (3886.71-4845.81) | 204.98 (166.18-250.27) | 156.51 (126.89-191.1) | 4720.34 (3896.37-5636.38) | 2131.14 (1759.14-2544.72) |
| Finland | 9590.54 (8290.49-10994.04) | 400.6 (346.3-459.22) | 15.99 (12.87-21.75) | 0.67 (0.54-0.91) | 1868.39 (1455.42-2311.44) | 78.04 (60.79-96.55) | 61176.07 (54960.76-68939.18) | 3106.87 (2791.22-3501.13) | 4002.88 (3367.23-4457.35) | 203.29 (171.01-226.37) | 51669.63 (45615.46-56925.74) | 1170.46 (1033.32-1289.52) |
| France | 93627.75 (80837.83-109048.78) | 326.16 (281.61-379.88) | 148.49 (121.73-180.3) | 0.52 (0.42-0.63) | 17840.51 (13993.67-21892.89) | 62.15 (48.75-76.27) | 499070.3 (442926.01-552234.15) | 2313.92 (2053.61-2560.41) | 28976.93 (23336.29-33068.04) | 134.35 (108.2-153.32) | 357228.64 (307314.16-397403.68) | 738.52 (635.33-821.58) |
| Gabon | 4046.87 (3465.39-4639.2) | 496.03 (424.76-568.63) | 14.63 (9.39-21.15) | 1.79 (1.15-2.59) | 1129.29 (861.64-1459.21) | 138.42 (105.61-178.86) | 6955.18 (6225.63-7787.32) | 3996.29 (3577.1-4474.41) | 357.98 (289.43-429.65) | 205.69 (166.3-246.87) | 6640.38 (5442.64-7875.83) | 2185.11 (1790.98-2591.66) |
| Gambia | 4920.14 (4287.82-5632.26) | 539.66 (470.3-617.77) | 20.7 (13.24-31.24) | 2.27 (1.45-3.43) | 1586.03 (1188.03-2078.18) | 173.96 (130.31-227.94) | 5780.53 (5138.74-6469.74) | 3848.73 (3421.42-4307.61) | 294.14 (237.93-354.44) | 195.84 (158.41-235.99) | 5353.23 (4417.76-6358.68) | 1893.64 (1562.73-2249.3) |
| Georgia | 8395.54 (6811.21-10306.52) | 497.97 (403.99-611.31) | 65.5 (49.84-84.17) | 3.88 (2.96-4.99) | 4175.03 (3407.54-5065.63) | 247.63 (202.11-300.46) | 31076.37 (26342.66-36719.07) | 2940.04 (2492.2-3473.88) | 4800.19 (3979.29-5623.81) | 454.13 (376.47-532.05) | 70299.18 (59749.75-81477.32) | 3262.03 (2772.52-3780.72) |
| Germany | 148255.68 (126780.7-171384.47) | 384.23 (328.57-444.17) | 366.35 (302.24-443.16) | 0.95 (0.78-1.15) | 33411.59 (27156.13-40290.41) | 86.59 (70.38-104.42) | 932206.07 (837102.59-1033657.42) | 3044.15 (2733.59-3375.45) | 51306.13 (44051.08-56813.68) | 167.54 (143.85-185.53) | 708074.35 (628030.28-779984.36) | 1045.77 (927.55-1151.98) |
| Ghana | 89049.64 (78706.41-100644.98) | 620.64 (548.55-701.45) | 591.09 (400.15-812.51) | 4.12 (2.79-5.66) | 38132.21 (29193.83-48401.09) | 265.76 (203.47-337.33) | 120390.19 (107074.5-135868.31) | 4641.86 (4128.45-5238.65) | 6631.35 (5580.83-7899.35) | 255.68 (215.18-304.57) | 125927.07 (104940.94-149920.28) | 2736.12 (2280.14-3257.44) |
| Greece | 16943.27 (13855.4-20564.25) | 358.66 (293.3-435.31) | 20.75 (17.3-25.08) | 0.44 (0.37-0.53) | 2976.23 (2264.5-3776.94) | 63 (47.94-79.95) | 106778.73 (90841.71-125929.92) | 2932.77 (2495.05-3458.78) | 13262.57 (11106.3-14571.2) | 364.27 (305.04-400.21) | 148155.33 (130049.54-161315.16) | 1760.94 (1545.74-1917.36) |
| Greenland | 191.65 (151.95-240.78) | 683.12 (541.63-858.25) | 0.34 (0.24-0.46) | 1.21 (0.86-1.63) | 38.47 (29.51-49.84) | 137.13 (105.2-177.66) | 748.83 (619.35-921.32) | 5810.88 (4806.14-7149.41) | 18.17 (15.15-21.16) | 141 (117.6-164.17) | 358.74 (304.96-418.44) | 1625.29 (1381.65-1895.79) |
| Grenada | 371.27 (320.66-432.03) | 703.6 (607.7-818.76) | 1.57 (1.23-1.97) | 2.97 (2.33-3.72) | 94.92 (78.85-114.85) | 179.89 (149.42-217.66) | 727.49 (635.88-830.98) | 3727.77 (3258.31-4258.07) | 48.85 (42.54-53.88) | 250.32 (217.97-276.08) | 804.32 (719.19-885.56) | 2233.16 (1996.8-2458.7) |
| Guam | 535.98 (474.91-604.87) | 682.16 (604.43-769.83) | 2.61 (1.99-3.4) | 3.33 (2.54-4.32) | 209.54 (169.86-255.02) | 266.69 (216.18-324.57) | 1650.13 (1498.45-1830.4) | 4951.04 (4495.94-5491.92) | 36.19 (29.97-43.04) | 108.58 (89.91-129.15) | 865.04 (727.28-1004.22) | 1364.53 (1147.23-1584.07) |
| Guatemala | 30727.33 (26163.89-35990.96) | 372.82 (317.45-436.69) | 52.77 (38.92-70.38) | 0.64 (0.47-0.85) | 5046.63 (3983.69-6278.63) | 61.23 (48.34-76.18) | 41686.85 (37055.49-46786.31) | 2306 (2049.8-2588.09) | 1896.74 (1509.43-2321.14) | 104.92 (83.5-128.4) | 28485.41 (23300.63-34347.07) | 803.09 (656.92-968.35) |
| Guinea | 20850.34 (18235.21-23699.07) | 442.3 (386.82-502.73) | 105.95 (71.92-154.27) | 2.25 (1.53-3.27) | 7569.32 (5723.69-9840.06) | 160.57 (121.42-208.74) | 26984.23 (24080.17-30158.26) | 3031.22 (2704.99-3387.76) | 1530.15 (1219.18-1907.81) | 171.89 (136.95-214.31) | 27382.82 (22261.07-33420.58) | 1662.9 (1351.87-2029.56) |
| Guinea-Bissau | 4295.89 (3771.34-4825.99) | 545.77 (479.13-613.11) | 25.46 (17.82-35.42) | 3.23 (2.26-4.5) | 1741.03 (1345.59-2236.08) | 221.19 (170.95-284.08) | 4070.22 (3621.03-4546.22) | 3607.16 (3209.08-4029.01) | 191.68 (150.07-239.62) | 169.88 (133-212.36) | 3947.84 (3206.61-4895) | 2023.34 (1643.45-2508.77) |
| Guyana | 2738.75 (2367.78-3232.59) | 719.52 (622.06-849.26) | 19 (13.2-25.95) | 4.99 (3.47-6.82) | 1041.1 (781.67-1360.57) | 273.51 (205.36-357.45) | 4459.9 (3880.65-5209.61) | 4114.22 (3579.87-4805.82) | 354.81 (283.95-436.46) | 327.31 (261.94-402.63) | 6243.45 (4996.39-7710.81) | 3275.45 (2621.22-4045.27) |
| Haiti | 34628.4 (29991.53-40911.93) | 601.16 (520.66-710.24) | 245.54 (152.61-420.81) | 4.26 (2.65-7.31) | 13745.93 (9388.07-21686.16) | 238.63 (162.98-376.48) | 40295.49 (35148.26-46582.04) | 3533.38 (3082.04-4084.63) | 3438.81 (2341.49-5261.71) | 301.54 (205.32-461.38) | 59788.06 (41043.61-90100.64) | 2971.26 (2039.72-4477.69) |
| Honduras | 18291.11 (15814.3-21051.17) | 410.84 (355.21-472.83) | 98.36 (62.39-142.67) | 2.21 (1.4-3.2) | 5846.84 (4095.76-7893.05) | 131.33 (92-177.29) | 23975.53 (21440.65-26733.82) | 2399.09 (2145.44-2675.09) | 2463.78 (2003.13-3220.12) | 246.54 (200.44-322.22) | 39829.64 (32886.94-51464.92) | 2122.03 (1752.14-2741.92) |
| Hungary | 27277.43 (23468.71-31813.02) | 592.32 (509.61-690.81) | 156.98 (118.15-202.63) | 3.41 (2.57-4.4) | 11108.51 (8915.81-13533.54) | 241.22 (193.6-293.88) | 156938.76 (137837.84-174231.2) | 4905.85 (4308.76-5446.41) | 9762.97 (8080.88-11473.66) | 305.19 (252.61-358.66) | 161100.23 (136803.71-187281.53) | 2336.05 (1983.74-2715.69) |
| Iceland | 477.18 (402.34-560.3) | 292.4 (246.55-343.34) | 0.38 (0.3-0.49) | 0.24 (0.18-0.3) | 75.75 (56.62-96.98) | 46.42 (34.7-59.43) | 2139.28 (1928.29-2375.6) | 2296.07 (2069.61-2549.71) | 100.13 (79.91-114.61) | 107.47 (85.77-123.01) | 1318.75 (1136.29-1489.48) | 678.23 (584.39-766.03) |
| India | 2576995.29 (2108784.91-3099134.67) | 374.08 (306.11-449.87) | 9922.46 (7506.24-13988.4) | 1.44 (1.09-2.03) | 765481.58 (613656.49-950224.34) | 111.12 (89.08-137.94) | 3346947.51 (2791882.74-4094274.24) | 1749.49 (1459.35-2140.12) | 261094.28 (219831.86-307273.62) | 136.48 (114.91-160.62) | 4831476.83 (4106612.42-5698425.29) | 1359.21 (1155.29-1603.1) |
| Indonesia | 918508.41 (748107.69-1123876.51) | 681.9 (555.4-834.37) | 6059.3 (3789.97-8478.23) | 4.5 (2.81-6.29) | 421443.49 (309753.31-532386.47) | 312.88 (229.96-395.25) | 2088775.4 (1717235.75-2571356.52) | 5681.86 (4671.21-6994.57) | 130014.24 (101821.4-151991.64) | 353.66 (276.97-413.45) | 2510215.44 (1977499.14-2934926.42) | 3959.41 (3119.15-4629.31) |
| Iran (Islamic Republic of) | 271884.43 (221878.84-331412.96) | 587.17 (479.17-715.72) | 1925.09 (1779.32-2078.29) | 4.16 (3.84-4.49) | 121374.91 (107901.27-135101.32) | 262.12 (233.03-291.77) | 551033.04 (463020.89-656022.29) | 4541.32 (3815.97-5406.59) | 31165.02 (27551.16-33591.86) | 256.85 (227.06-276.85) | 539321.12 (488542.94-578004.27) | 2412.69 (2185.53-2585.75) |
| Iraq | 140330.89 (125455.1-157820.83) | 692.95 (619.49-779.32) | 1238.01 (861.79-1730.67) | 6.11 (4.26-8.55) | 71669.04 (54289.54-93829.58) | 353.9 (268.08-463.33) | 250658.26 (223045.71-280570.05) | 6815.28 (6064.51-7628.57) | 15408.39 (12757.53-17802.06) | 418.95 (346.87-484.03) | 295862.39 (244676.58-342678.03) | 4531.31 (3747.37-5248.32) |
| Ireland | 7143.62 (5803.56-8746.34) | 307.23 (249.6-376.16) | 6.07 (4.86-7.65) | 0.26 (0.21-0.33) | 1140.49 (843.22-1466.51) | 49.05 (36.26-63.07) | 24770.77 (21072.22-29441.7) | 1988.05 (1691.21-2362.93) | 1617.47 (1344.68-1823.51) | 129.81 (107.92-146.35) | 20635.26 (18003.98-22826.07) | 794.19 (692.92-878.51) |
| Israel | 12537.35 (10644.12-14789) | 306.37 (260.1-361.39) | 12.56 (10.26-15.29) | 0.31 (0.25-0.37) | 2097.38 (1590.82-2676.01) | 51.25 (38.87-65.39) | 42766.7 (38337.83-47592.6) | 2246.78 (2014.11-2500.31) | 1938.06 (1613.5-2173.96) | 101.82 (84.77-114.21) | 27123.93 (23708.62-30024.35) | 670.53 (586.1-742.23) |
| Italy | 90309.12 (73496.67-110493.07) | 329.38 (268.06-403) | 103.57 (91.71-112.2) | 0.38 (0.33-0.41) | 15518.2 (11859.69-19578.33) | 56.6 (43.26-71.41) | 521824.74 (438270.86-619573.6) | 2365.64 (1986.85-2808.77) | 45284.46 (37227.25-50791.13) | 205.29 (168.77-230.26) | 511088.63 (440092.56-558385.74) | 1012.78 (872.09-1106.5) |
| Jamaica | 8435.8 (7305.21-9721.26) | 585.87 (507.35-675.15) | 19.21 (13.72-26.28) | 1.33 (0.95-1.83) | 1522 (1185.91-1906.31) | 105.7 (82.36-132.39) | 16415.81 (14544.37-18466.49) | 3236.73 (2867.74-3641.07) | 1583.87 (1271.61-1879.17) | 312.3 (250.73-370.52) | 20315.45 (16746.44-24110.07) | 2007.02 (1654.42-2381.9) |
| Japan | 271642.13 (216318.95-338747.2) | 498.93 (397.32-622.19) | 345.12 (324.58-368.9) | 0.63 (0.6-0.68) | 55665.52 (41643.39-71070.25) | 102.24 (76.49-130.54) | 2391221.85 (2040165.6-2782072.37) | 4621.02 (3942.61-5376.34) | 90828.29 (69624.18-103374.45) | 175.53 (134.55-199.77) | 1302331.56 (1084297.53-1472364.56) | 1024 (852.57-1157.7) |
| Jordan | 40077.17 (34798.75-46147.94) | 708.36 (615.06-815.66) | 128.71 (99.74-162.52) | 2.27 (1.76-2.87) | 10837.7 (8736.08-13141.35) | 191.55 (154.41-232.27) | 66787.51 (58039.49-74772.75) | 6255.86 (5436.45-7003.82) | 2339.89 (1898.64-2765.78) | 219.17 (177.84-259.07) | 47892.06 (40259.89-55740.32) | 2583.59 (2171.87-3006.97) |
| Kazakhstan | 53369.56 (45159.26-63924.03) | 587.3 (496.95-703.44) | 537.63 (426.43-664.4) | 5.92 (4.69-7.31) | 31766.65 (26345.84-38078.4) | 349.57 (289.92-419.03) | 115718.96 (99688.76-133887) | 3803.78 (3276.86-4400.98) | 13014.68 (11373.31-14772.36) | 427.8 (373.85-485.58) | 227897.54 (201406.3-256579.55) | 4243.59 (3750.31-4777.67) |
| Kenya | 120061.22 (98420.32-144436.26) | 554.12 (454.24-666.62) | 230.58 (173.82-337.16) | 1.06 (0.8-1.56) | 25150.14 (19589.48-32119.92) | 116.08 (90.41-148.24) | 119868.88 (100535.35-145159.07) | 3388.42 (2841.91-4103.32) | 5208.76 (4050.67-6379.26) | 147.24 (114.5-180.33) | 99708.52 (80608.79-119400.46) | 1623.36 (1312.4-1943.97) |
| Kiribati | 338.53 (301.5-378.63) | 626.58 (558.04-700.81) | 4.75 (3.45-6.22) | 8.8 (6.39-11.52) | 277.01 (216.63-350.15) | 512.72 (400.97-648.09) | 485.57 (435.26-547.96) | 4176.03 (3743.36-4712.6) | 25.91 (20.39-31.54) | 222.84 (175.37-271.27) | 606.76 (490.29-731.74) | 3294.08 (2661.76-3972.56) |
| Kuwait | 17271.45 (15056.81-19813.41) | 588.79 (513.3-675.45) | 56.79 (43.49-73.38) | 1.94 (1.48-2.5) | 4828.77 (3914.55-5920.02) | 164.62 (133.45-201.82) | 14487.72 (12988.54-16008.43) | 3650.85 (3273.06-4034.06) | 558.91 (452.78-672.54) | 140.84 (114.1-169.48) | 10122.82 (8486.98-11888.44) | 1513.55 (1268.96-1777.55) |
| Kyrgyzstan | 13224.8 (11257.66-15370.81) | 422.21 (359.41-490.72) | 155.95 (126.85-187.56) | 4.98 (4.05-5.99) | 8768.59 (7418.02-10237.82) | 279.94 (236.82-326.85) | 20872.69 (18237.62-23705.85) | 2644.01 (2310.22-3002.9) | 2364.59 (2072.3-2668.61) | 299.53 (262.5-338.04) | 44818.87 (39672.54-50745.77) | 3310.29 (2930.18-3748.04) |
| Lao People's Democratic Republic | 14520.59 (12666.74-16530.32) | 417.41 (364.12-475.18) | 108.52 (70.57-167.93) | 3.12 (2.03-4.83) | 7315.05 (5439.59-9748.3) | 210.28 (156.37-280.23) | 30650.41 (26993.99-34763.94) | 4241.4 (3735.42-4810.63) | 2024.47 (1619.61-2528.87) | 280.15 (224.12-349.94) | 38690.63 (30919.96-48109.69) | 3078.47 (2460.18-3827.91) |
| Latvia | 5270.58 (4418.98-6285.97) | 612.82 (513.8-730.88) | 32.98 (24.52-44.14) | 3.84 (2.85-5.13) | 2226.98 (1779.98-2766) | 258.94 (206.96-321.61) | 28992.71 (24489.45-33235.57) | 4371.86 (3692.81-5011.65) | 4046.38 (3410.49-4779.98) | 610.16 (514.27-720.78) | 57004.42 (49034.43-66944.19) | 3942.02 (3390.87-4629.39) |
| Lebanon | 16293.48 (14344.69-18385.07) | 630.73 (555.29-711.7) | 50.88 (28.51-74.86) | 1.97 (1.1-2.9) | 4493.47 (3323.47-5821.28) | 173.95 (128.65-225.35) | 47508.23 (42516.15-53000.1) | 5484.97 (4908.62-6119.02) | 1417.97 (950.53-1841.03) | 163.71 (109.74-212.55) | 26216.55 (20826.73-32361.64) | 1487.78 (1181.91-1836.51) |
| Lesotho | 4647.3 (4042.88-5294.77) | 464.66 (404.23-529.4) | 18.7 (10.48-31.5) | 1.87 (1.05-3.15) | 1373.3 (938.77-1959.48) | 137.31 (93.86-195.92) | 7440.77 (6637.45-8445.67) | 3529.46 (3148.41-4006.12) | 577.2 (429.32-761.29) | 273.79 (203.64-361.11) | 10540.59 (7906.27-13887.03) | 2823.86 (2118.12-3720.38) |
| Liberia | 10091.8 (8736.03-11551.76) | 485.41 (420.2-555.63) | 39.39 (24.99-62.29) | 1.89 (1.2-3) | 3078.68 (2290.64-4129.45) | 148.08 (110.18-198.62) | 9037.09 (8105.4-10145.23) | 2888.48 (2590.69-3242.67) | 438.18 (347.76-563.91) | 140.05 (111.15-180.24) | 7956.07 (6377.77-10114.01) | 1422.4 (1140.22-1808.19) |
| Libya | 29798.47 (26478.87-33144.01) | 766.39 (681.01-852.43) | 254.17 (170.2-356.49) | 6.54 (4.38-9.17) | 15483.19 (11554.23-20259.54) | 398.21 (297.16-521.06) | 43965.76 (39340.22-49064.85) | 5557.7 (4972.99-6202.28) | 2032.16 (1532.01-2734.71) | 256.88 (193.66-345.69) | 38304.36 (29845.42-48872.97) | 2625.87 (2045.98-3350.38) |
| Lithuania | 8106.54 (6715.59-9652.66) | 635.22 (526.23-756.38) | 40.52 (29.52-53.62) | 3.17 (2.31-4.2) | 3047.56 (2425.34-3792.78) | 238.8 (190.05-297.2) | 38890.35 (32090.19-46831.69) | 4034.17 (3328.77-4857.94) | 4013.18 (3326.62-4720.52) | 416.29 (345.08-489.67) | 59204.59 (50794.61-69413.65) | 2843.94 (2439.96-3334.34) |
| Luxembourg | 984.35 (818.88-1178.03) | 307.15 (255.52-367.59) | 1.26 (0.99-1.58) | 0.39 (0.31-0.49) | 175.92 (135.46-225.21) | 54.89 (42.27-70.28) | 3435.5 (3038.64-3989.05) | 2072.62 (1833.2-2406.57) | 222.31 (180.73-254.83) | 134.12 (109.03-153.74) | 2820.58 (2426.05-3196.46) | 819.84 (705.17-929.1) |
| Madagascar | 71629.39 (62796.56-80571.95) | 647.3 (567.48-728.11) | 333.16 (223.75-522.87) | 3.01 (2.02-4.73) | 24026.44 (18285.09-33670.79) | 217.12 (165.24-304.27) | 81586.37 (72660.37-91261.89) | 4655.02 (4145.73-5207.07) | 3563.81 (2676.49-4625.24) | 203.34 (152.71-263.9) | 73981.09 (57808.15-94428.16) | 2574.62 (2011.78-3286.2) |
| Malawi | 36590.45 (31495.9-42125.47) | 513.51 (442.02-591.19) | 105.91 (66.9-161.89) | 1.49 (0.94-2.27) | 9288.4 (6939.07-12190.53) | 130.35 (97.38-171.08) | 41348.07 (36726.12-46587.53) | 3618.69 (3214.18-4077.23) | 2351.37 (1832.51-2928.32) | 205.79 (160.38-256.28) | 43618.72 (35273.03-53773.63) | 2093.16 (1692.67-2580.47) |
| Malaysia | 80968.36 (70148.18-92853.31) | 498.23 (431.65-571.37) | 367.57 (262.93-504.16) | 2.26 (1.62-3.1) | 30438.03 (23750.48-37359.94) | 187.3 (146.15-229.89) | 204982.97 (182576.74-228931.19) | 4428.16 (3944.13-4945.5) | 7827.32 (6154.02-9964.64) | 169.09 (132.94-215.26) | 165522.01 (135565.83-202585.38) | 1970.37 (1613.78-2411.58) |
| Maldives | 1098.58 (939.52-1278.09) | 354.54 (303.21-412.47) | 2.87 (2.08-3.71) | 0.93 (0.67-1.2) | 324.72 (251.62-406.18) | 104.79 (81.2-131.09) | 1408.11 (1258.58-1577.82) | 2910.92 (2601.79-3261.74) | 64.75 (52.83-78.04) | 133.85 (109.22-161.33) | 1150.12 (971.28-1341.38) | 1343.3 (1134.43-1566.69) |
| Mali | 33425.07 (28914.38-38112.76) | 431.5 (373.27-492.02) | 133.89 (90.12-198.88) | 1.73 (1.16-2.57) | 10574.87 (8097.81-13816.98) | 136.52 (104.54-178.37) | 37558.33 (33420.04-42289.25) | 2691.3 (2394.77-3030.31) | 1926.66 (1503.44-2381.2) | 138.06 (107.73-170.63) | 34842.1 (28358.23-42626.75) | 1398.16 (1137.97-1710.55) |
| Malta | 582.32 (478.74-707.76) | 288.37 (237.07-350.48) | 0.87 (0.69-1.08) | 0.43 (0.34-0.54) | 110.5 (85.31-140.93) | 54.72 (42.25-69.79) | 3377.39 (2907.5-4037.81) | 2196.3 (1890.73-2625.77) | 227.73 (187.73-262.15) | 148.09 (122.08-170.48) | 3053.58 (2632.5-3436.84) | 909.65 (784.21-1023.82) |
| Marshall Islands | 160.38 (143.64-180.28) | 589.68 (528.13-662.85) | 1.41 (0.9-2.18) | 5.2 (3.31-8.01) | 92.73 (68.23-126.76) | 340.95 (250.85-466.06) | 237.63 (212.45-267.26) | 4162.73 (3721.65-4681.8) | 8.43 (6.41-11.26) | 147.67 (112.23-197.34) | 205.23 (161.31-263.99) | 2237.87 (1758.91-2878.59) |
| Mauritania | 8658.7 (7571.75-9848.09) | 538.19 (470.63-612.12) | 28.57 (17.47-44.68) | 1.78 (1.09-2.78) | 2397.71 (1789.77-3192.47) | 149.03 (111.24-198.43) | 12266.26 (11013.29-13658.37) | 3574.86 (3209.69-3980.57) | 506.03 (411.11-613.05) | 147.48 (119.81-178.67) | 9330.73 (7659.57-11225.84) | 1468.65 (1205.61-1766.94) |
| Mauritius | 3303.64 (2878.98-3800.8) | 508.61 (443.23-585.15) | 12.59 (9.31-16.59) | 1.94 (1.43-2.55) | 1126.87 (899.19-1399.23) | 173.49 (138.43-215.42) | 11462.7 (10311.97-12741.39) | 3593.69 (3232.92-3994.58) | 549.28 (450.05-649.57) | 172.21 (141.1-203.65) | 9989.7 (8447.45-11658.05) | 1698.09 (1435.94-1981.69) |
| Mexico | 345019.87 (282233.91-417098.53) | 556.42 (455.16-672.66) | 641.75 (517.81-768.13) | 1.03 (0.84-1.24) | 55998.62 (45741.46-67472.49) | 90.31 (73.77-108.81) | 562915.26 (481885.09-663917.69) | 2855.24 (2444.23-3367.54) | 19499.12 (16433.94-22294.98) | 98.9 (83.36-113.09) | 317980.76 (277543.85-360089.58) | 841.34 (734.35-952.75) |
| Micronesia (Federated States of) | 254.07 (225.86-285.58) | 538.16 (478.41-604.89) | 2.54 (1.3-4.05) | 5.38 (2.76-8.59) | 158.14 (97.76-231.36) | 334.96 (207.07-490.07) | 445.65 (399.93-498.36) | 3606.9 (3236.85-4033.52) | 20.31 (15.26-25.7) | 164.38 (123.49-208.01) | 457.11 (348.27-571.67) | 2348.92 (1789.67-2937.65) |
| Monaco | 61.5 (52.16-72.08) | 393.44 (333.66-461.1) | 0.15 (0.1-0.22) | 0.99 (0.67-1.41) | 14.08 (10.94-17.72) | 90.05 (69.96-113.37) | 477.23 (425.6-545.76) | 3150.55 (2809.68-3602.95) | 33.5 (25.75-40.65) | 221.18 (169.99-268.35) | 418.76 (343.75-500.95) | 1229.85 (1009.57-1471.23) |
| Mongolia | 7121.64 (6137.37-8221.98) | 402.12 (346.54-464.25) | 70.55 (49.52-98.06) | 3.98 (2.8-5.54) | 4235.52 (3277.57-5353.23) | 239.16 (185.07-302.27) | 7175.83 (6254.03-8084.27) | 1861.92 (1622.74-2097.64) | 360.65 (278.43-472.95) | 93.58 (72.25-122.72) | 8242.31 (6582.56-10541.82) | 1311.65 (1047.52-1677.58) |
| Montenegro | 1226.02 (1042.69-1428.96) | 413.81 (351.93-482.31) | 3.51 (2.62-4.69) | 1.19 (0.88-1.58) | 357.57 (278.86-445.7) | 120.69 (94.12-150.44) | 5941.4 (5241.13-6724.46) | 3406.62 (3005.1-3855.6) | 397.39 (327.95-464.23) | 227.85 (188.04-266.17) | 6572.1 (5595.72-7584.61) | 1911.23 (1627.29-2205.68) |
| Morocco | 141180.47 (126634.95-157755.54) | 786.08 (705.09-878.37) | 1234.92 (825.06-1795.07) | 6.88 (4.59-9.99) | 71842.73 (53052.65-98083.33) | 400.01 (295.39-546.12) | 321000.51 (286238.14-359982.86) | 5891.39 (5253.39-6606.84) | 19950.99 (16066.55-24105.35) | 366.16 (294.87-442.41) | 375213.2 (305015.66-454234.21) | 3920.23 (3186.81-4745.85) |
| Mozambique | 67646.1 (59159.02-76227.71) | 627.52 (548.79-707.12) | 322.52 (199.42-493.99) | 2.99 (1.85-4.58) | 22251.04 (16480.07-30044.82) | 206.41 (152.88-278.71) | 83804.02 (74852.77-93639.6) | 4830.05 (4314.14-5396.92) | 5148.36 (4028.58-6564.57) | 296.73 (232.19-378.35) | 103352 (81669.25-131327.44) | 3378.93 (2670.05-4293.54) |
| Myanmar | 105531.03 (90888.21-121537.78) | 393.45 (338.85-453.12) | 1247.38 (843.52-1723.71) | 4.65 (3.14-6.43) | 72960.74 (53412.02-94929.44) | 272.02 (199.13-353.92) | 247715.01 (217773.37-281014.85) | 3129.86 (2751.55-3550.6) | 30766.67 (25970.15-35866.95) | 388.73 (328.13-453.18) | 514942.74 (436294.89-602645.62) | 3637.47 (3081.91-4256.99) |
| Namibia | 5484.87 (4737.75-6315.11) | 499.24 (431.23-574.81) | 14.96 (9.34-22.74) | 1.36 (0.85-2.07) | 1318.01 (996.3-1716.06) | 119.97 (90.68-156.2) | 9221.84 (8188.88-10386.6) | 4069.55 (3613.71-4583.55) | 725.06 (593.55-875.79) | 319.97 (261.93-386.48) | 12057.19 (9985.62-14566.66) | 2843.65 (2355.07-3435.5) |
| Nauru | 31.25 (27.69-34.93) | 643.05 (569.85-718.87) | 0.46 (0.31-0.65) | 9.39 (6.38-13.38) | 26.61 (19.24-35.67) | 547.7 (395.99-733.94) | 28.31 (25.33-31.79) | 4389.41 (3928.13-4930.34) | 1.14 (0.89-1.46) | 176.85 (137.25-225.78) | 29.54 (23.41-37.26) | 3257.48 (2581.6-4109.57) |
| Nepal | 36853.97 (31322.65-42767.45) | 260.94 (221.77-302.81) | 150.56 (86.62-261.44) | 1.07 (0.61-1.85) | 11263.9 (8004.26-15921.9) | 79.75 (56.67-112.73) | 48645.96 (42429.89-55481.06) | 1289.12 (1124.39-1470.25) | 5803.91 (4179.74-8065.83) | 153.8 (110.76-213.74) | 101506.08 (72775.88-142790.14) | 1459.42 (1046.35-2052.99) |
| Netherlands | 28400.85 (24285.19-33200.91) | 366.89 (313.73-428.9) | 53.02 (43.27-63.68) | 0.68 (0.56-0.82) | 5713.11 (4520.93-7020.73) | 73.8 (58.4-90.7) | 144020.96 (129635.99-159966.46) | 2550.82 (2296.04-2833.24) | 8430.63 (7108.11-9494.83) | 149.32 (125.9-168.17) | 112678.74 (99409.5-124716.51) | 930.55 (820.97-1029.97) |
| New Zealand | 7042.72 (5761.57-8563.37) | 353.46 (289.16-429.78) | 8.69 (7.42-10.19) | 0.44 (0.37-0.51) | 1313.87 (1007.61-1683.08) | 65.94 (50.57-84.47) | 27666.59 (23544.7-33177.34) | 2097.66 (1785.14-2515.48) | 1913.91 (1571.22-2157.58) | 145.11 (119.13-163.59) | 23599.3 (20417.69-26233.93) | 852.29 (737.39-947.45) |
| Nicaragua | 13998.17 (12000.57-16258.14) | 445.4 (381.84-517.31) | 22.18 (16.54-29.25) | 0.71 (0.53-0.93) | 2098.16 (1649.53-2601.48) | 66.76 (52.49-82.78) | 17804.96 (16011.9-19704.09) | 2410.32 (2167.59-2667.41) | 978.98 (831.58-1119.82) | 132.53 (112.57-151.59) | 15512.96 (13226.01-17815.65) | 1142.24 (973.85-1311.79) |
| Niger | 31377.61 (27287.29-35622.73) | 421.64 (366.67-478.68) | 133.35 (81.93-214.24) | 1.79 (1.1-2.88) | 10163.7 (7457-14026.76) | 136.57 (100.2-188.48) | 34239.73 (30563.74-38583.63) | 2642.77 (2359.05-2978.06) | 1595.47 (1145.97-2112.67) | 123.15 (88.45-163.07) | 31830.57 (23769.84-42682.67) | 1444.41 (1078.63-1936.86) |
| Nigeria | 397820.41 (327723.13-476678.71) | 478.99 (394.59-573.93) | 1221.39 (822.02-2094.35) | 1.47 (0.99-2.52) | 105351.78 (80038.17-144696.15) | 126.85 (96.37-174.22) | 406179.04 (339286-493361.44) | 2909.12 (2430.02-3533.54) | 20591.66 (16452.5-24945.07) | 147.48 (117.84-178.66) | 377093.17 (305277.5-459625.35) | 1550.41 (1255.14-1889.73) |
| Niue | 4.75 (4.2-5.35) | 636.58 (563.29-717.51) | 0.03 (0.02-0.05) | 4.56 (2.83-7.09) | 2.3 (1.63-3.15) | 308.41 (218.7-422) | 18.07 (16.19-20.33) | 4673.25 (4187.38-5257.72) | 0.86 (0.65-1.08) | 222.44 (168.84-279.96) | 16.22 (12.88-19.87) | 2188.22 (1738.17-2680.64) |
| North Macedonia | 6563.47 (5298-8056.07) | 589.58 (475.91-723.66) | 46.82 (33.47-64.07) | 4.21 (3.01-5.76) | 3078.54 (2392.24-3859.08) | 276.54 (214.89-346.65) | 33630.5 (27971.08-40527.54) | 5879.92 (4890.44-7085.8) | 4898.06 (4098.41-5799.98) | 856.37 (716.56-1014.06) | 77333.79 (64526.33-91688.2) | 7102.97 (5926.63-8421.4) |
| Northern Mariana Islands | 136.91 (120.38-155.49) | 658.82 (579.26-748.22) | 0.7 (0.49-0.96) | 3.37 (2.38-4.62) | 53.5 (41.67-66.46) | 257.43 (200.53-319.8) | 353.01 (317.86-394.51) | 3509.8 (3160.31-3922.36) | 8.99 (7.61-10.41) | 89.43 (75.64-103.5) | 227.17 (194.06-261.54) | 1465.03 (1251.52-1686.68) |
| Norway | 11624.41 (9530.28-14052.53) | 458.08 (375.56-553.76) | 7.57 (6.76-8.36) | 0.3 (0.27-0.33) | 1741.56 (1291.39-2245.04) | 68.63 (50.89-88.47) | 62211.57 (52714.76-73539.58) | 3998.91 (3388.47-4727.07) | 2260.32 (1892.46-2514.8) | 145.29 (121.65-161.65) | 32084.22 (27971.22-35476.67) | 953.28 (831.08-1054.08) |
| Oman | 15284.8 (13224.16-17795.74) | 505.11 (437.02-588.09) | 56.65 (41.63-80.99) | 1.87 (1.38-2.68) | 4640.36 (3637.16-5946.54) | 153.35 (120.2-196.51) | 13123.35 (11622.46-14866.52) | 4768.11 (4222.79-5401.46) | 588.32 (499.71-683.72) | 213.75 (181.56-248.42) | 12060.74 (10404.73-13839.29) | 2778.51 (2397.01-3188.25) |
| Pakistan | 417573.26 (341540.13-504397.92) | 442.27 (361.74-534.23) | 2330.84 (1728.29-3420.11) | 2.47 (1.83-3.62) | 159429.8 (124884.78-207706.97) | 168.86 (132.27-219.99) | 441247.65 (364181.59-540113.93) | 2481.63 (2048.2-3037.67) | 36441.49 (30221.39-48468.42) | 204.95 (169.97-272.59) | 688934.13 (576369.18-905194.01) | 2218.29 (1855.85-2914.63) |
| Palau | 77.74 (69.05-87.74) | 808.48 (718.12-912.49) | 0.86 (0.59-1.2) | 8.96 (6.14-12.5) | 50.66 (37.99-66.03) | 526.84 (395.03-686.7) | 184.84 (165.21-205.48) | 4803.35 (4293.12-5339.74) | 6.73 (5.38-8.33) | 174.93 (139.9-216.49) | 163.82 (134.38-199.86) | 2595.78 (2129.31-3166.87) |
| Palestine | 11813.34 (10230.28-13737.08) | 538.92 (466.7-626.68) | 70.78 (55.95-87.11) | 3.23 (2.55-3.97) | 4605.09 (3817.84-5432.6) | 210.08 (174.17-247.83) | 20825.49 (18415.5-23728.57) | 5343.84 (4725.43-6088.77) | 1437.73 (1223.3-1636.32) | 368.92 (313.9-419.88) | 26044.77 (22619.04-29552.33) | 3873.83 (3364.3-4395.54) |
| Panama | 9096.91 (7837.16-10522.78) | 461.7 (397.76-534.06) | 11.44 (7.8-16.19) | 0.58 (0.4-0.82) | 1236.06 (946.89-1548.43) | 62.73 (48.06-78.59) | 19408.82 (17424.88-21481.96) | 2787.71 (2502.75-3085.48) | 979.24 (731.98-1218.28) | 140.65 (105.13-174.98) | 13093.07 (10349.39-16056.7) | 941.71 (744.37-1154.87) |
| Papua New Guinea | 19679.53 (17265.99-22387.53) | 438.7 (384.9-499.07) | 97.19 (52.67-172.17) | 2.17 (1.17-3.84) | 7881.54 (5552.91-11199.42) | 175.7 (123.79-249.66) | 22719.07 (20378.23-25401.77) | 3048.84 (2734.7-3408.85) | 710.43 (463.09-1094.11) | 95.34 (62.15-146.83) | 16993.61 (12494.55-24162.06) | 1379.96 (1014.62-1962.07) |
| Paraguay | 15559.7 (13436.29-17962.51) | 461.15 (398.22-532.37) | 38.05 (25.09-56.6) | 1.13 (0.74-1.68) | 2851.87 (2168.83-3687.64) | 84.52 (64.28-109.29) | 31529.9 (28099.24-35193) | 3351.34 (2986.69-3740.69) | 1742.57 (1316.86-2261.61) | 185.22 (139.97-240.39) | 26656.11 (20943.85-34276.09) | 1495.25 (1174.82-1922.68) |
| Peru | 68566.25 (59074.62-79269.52) | 411.31 (354.37-475.51) | 126.11 (82.02-182.54) | 0.76 (0.49-1.09) | 11329.6 (8564.74-14416.87) | 67.96 (51.38-86.48) | 116878.8 (105364.92-129544.32) | 2194.17 (1978.02-2431.94) | 4485.19 (3281.42-5941.21) | 84.2 (61.6-111.53) | 65019.03 (50244.34-83958.3) | 613.71 (474.25-792.47) |
| Philippines | 243396.76 (198307.95-296136.4) | 461.76 (376.22-561.81) | 1482.13 (1076.49-1842.52) | 2.81 (2.04-3.5) | 108310.03 (85447-129290.14) | 205.48 (162.1-245.28) | 497064.34 (415329.38-590266.54) | 3787.61 (3164.79-4497.81) | 22813.16 (19279.43-26551.84) | 173.84 (146.91-202.32) | 468228.52 (394133.96-541556.94) | 2023.3 (1703.12-2340.16) |
| Poland | 83624.1 (67877.99-102130.48) | 448.03 (363.66-547.18) | 413.96 (330.76-510.99) | 2.22 (1.77-2.74) | 31710.68 (25809.7-38141.8) | 169.89 (138.28-204.35) | 427936.22 (362337.19-506128.56) | 3547.97 (3004.09-4196.25) | 32554.45 (26959.16-37631.07) | 269.9 (223.51-311.99) | 497348.44 (429679.51-568430.12) | 1992.25 (1721.19-2276.99) |
| Portugal | 19150.7 (15526.46-23509.62) | 391.45 (317.37-480.55) | 28.93 (23.63-35.18) | 0.59 (0.48-0.72) | 3561.53 (2769.66-4495.12) | 72.8 (56.61-91.88) | 112542.13 (95745.62-133880.3) | 2970.42 (2527.09-3533.61) | 11592.15 (9853.38-12801.49) | 305.96 (260.07-337.88) | 137537.84 (122073.05-150038.37) | 1611.57 (1430.36-1758.04) |
| Puerto Rico | 10102.66 (8792.36-11665.63) | 635.11 (552.73-733.36) | 6.21 (4.3-8.6) | 0.39 (0.27-0.54) | 1074.09 (800.45-1370.07) | 67.52 (50.32-86.13) | 33565.9 (30083.24-37360.87) | 2853.95 (2557.84-3176.62) | 1116.47 (837.56-1381.68) | 94.93 (71.21-117.48) | 15267.49 (12408.3-18333.11) | 591.26 (480.53-709.98) |
| Qatar | 10470.14 (9032.95-12177.74) | 481.61 (415.5-560.15) | 13.66 (9.27-19.49) | 0.63 (0.43-0.9) | 1990.64 (1505.33-2491.58) | 91.57 (69.24-114.61) | 4847.03 (4288.81-5429.04) | 2804.68 (2481.67-3141.45) | 55.96 (42.45-77.72) | 32.38 (24.56-44.97) | 1702.66 (1358.39-2171.38) | 748.99 (597.55-955.17) |
| Republic of Korea | 137273.15 (116236.27-161335.14) | 493.2 (417.62-579.65) | 231.76 (185.72-346.48) | 0.83 (0.67-1.24) | 30435.65 (23435.17-38391.45) | 109.35 (84.2-137.93) | 615479.68 (543236.76-717241.17) | 3838.89 (3388.3-4473.6) | 22345.28 (18658.49-27036.84) | 139.37 (116.38-168.64) | 381994.8 (333333.94-443199.36) | 1240.4 (1082.39-1439.14) |
| Republic of Moldova | 9399.6 (7985.44-10985.3) | 501.36 (425.93-585.94) | 67.11 (54.75-81.61) | 3.58 (2.92-4.35) | 4402.87 (3716.31-5210.07) | 234.84 (198.22-277.9) | 33360.86 (29051.45-38089.35) | 3206.25 (2792.08-3660.69) | 3538.4 (3067.35-4001.91) | 340.07 (294.8-384.62) | 60979.73 (54046.38-69129) | 3028.69 (2684.33-3433.44) |
| Romania | 52109.93 (43498.01-63386.33) | 576.54 (481.26-701.3) | 338.14 (255.95-436.33) | 3.74 (2.83-4.83) | 22837.67 (18384.67-28288.91) | 252.67 (203.41-312.98) | 319287.28 (263795.05-372317.81) | 5199.2 (4295.58-6062.74) | 36139.78 (30491.93-42088.79) | 588.49 (496.52-685.36) | 528050.71 (454751.54-611376.15) | 3992.91 (3438.65-4622.99) |
| Russian Federation | 446949.31 (364186.47-547601.26) | 639.65 (521.2-783.69) | 4576.34 (3808.52-5419.59) | 6.55 (5.45-7.76) | 267765.53 (225501.53-314265.78) | 383.21 (322.72-449.76) | 1878088.65 (1593311.05-2226923.41) | 4423.08 (3752.4-5244.62) | 250076.33 (215972.16-279818.58) | 588.95 (508.64-659) | 3869558.93 (3438984.52-4342284.82) | 4648.92 (4131.63-5216.86) |
| Rwanda | 23340.02 (19844.33-27003.45) | 429.54 (365.21-496.96) | 44.33 (27.08-78.3) | 0.82 (0.5-1.44) | 4871.31 (3612.3-6514.53) | 89.65 (66.48-119.89) | 28487.3 (25406.15-31880.56) | 2837.02 (2530.17-3174.95) | 1401.65 (1007.62-1846.95) | 139.59 (100.35-183.94) | 26176.99 (19913.11-33987.45) | 1522.29 (1158.02-1976.49) |
| Saint Kitts and Nevis | 238.27 (203.4-282.87) | 764.61 (652.71-907.75) | 1.1 (0.62-1.57) | 3.54 (2-5.02) | 64.01 (42.92-84.47) | 205.42 (137.74-271.07) | 464.47 (403.76-531.28) | 3840.85 (3338.84-4393.33) | 32.87 (28.7-37.52) | 271.77 (237.35-310.26) | 539.97 (474.23-612.27) | 2644.23 (2322.31-2998.29) |
| Saint Lucia | 661.24 (565.4-776.85) | 713.96 (610.48-838.79) | 2.31 (1.76-3) | 2.5 (1.9-3.23) | 149.77 (121.16-183.51) | 161.72 (130.82-198.14) | 1334.38 (1177.3-1532.94) | 3632.37 (3204.78-4172.87) | 95.47 (80.91-110.03) | 259.89 (220.24-299.52) | 1367.05 (1173.41-1573.64) | 1918.49 (1646.74-2208.41) |
| Saint Vincent and the Grenadines | 377.97 (327.11-437.92) | 684.89 (592.72-793.51) | 1.61 (1.25-2.02) | 2.92 (2.27-3.65) | 98.45 (80.67-117.81) | 178.38 (146.18-213.47) | 849.11 (752.33-956.89) | 3569.01 (3162.22-4022.06) | 56.78 (49.6-64.4) | 238.67 (208.46-270.67) | 866.85 (758.03-978.82) | 1915.88 (1675.36-2163.35) |
| Samoa | 500.75 (441.24-566.35) | 560.43 (493.83-633.86) | 3.4 (2.17-4.96) | 3.81 (2.43-5.55) | 240.15 (175.14-313.46) | 268.77 (196.02-350.82) | 1086.39 (979.46-1211.49) | 4421.33 (3986.16-4930.48) | 48.07 (38.93-58.59) | 195.65 (158.42-238.46) | 941.26 (780.78-1119.52) | 2122.02 (1760.23-2523.9) |
| San Marino | 52.91 (45.04-61.51) | 339.31 (288.82-394.46) | 0.09 (0.05-0.15) | 0.58 (0.33-0.96) | 10.41 (7.62-13.67) | 66.76 (48.85-87.69) | 262.67 (232.55-296.26) | 2599.22 (2301.1-2931.56) | 21.09 (15.12-27.88) | 208.69 (149.61-275.88) | 256.75 (190.4-333.13) | 1144.97 (849.12-1485.61) |
| Sao Tome and Principe | 672.93 (596.42-754.17) | 738.78 (654.77-827.97) | 2.85 (1.98-4.11) | 3.13 (2.17-4.52) | 217.8 (169.1-288.76) | 239.11 (185.64-317.02) | 840.3 (750.78-941.98) | 4944.23 (4417.49-5542.5) | 35.36 (29.05-45.82) | 208.07 (170.95-269.62) | 664.47 (545.64-836.98) | 2205.82 (1811.33-2778.51) |
| Saudi Arabia | 161507 (141660.44-187162.67) | 691.92 (606.9-801.84) | 1368.73 (919.42-1858.35) | 5.86 (3.94-7.96) | 81981.99 (61485.39-105738.96) | 351.23 (263.41-453) | 146728.31 (130759.62-165981.98) | 5397.49 (4810.08-6105.75) | 5655.41 (4267.74-6697.08) | 208.04 (156.99-246.36) | 123471.62 (96859.66-145469.19) | 2911.66 (2284.11-3430.4) |
| Senegal | 31367.31 (27349.25-35702.87) | 512.89 (447.19-583.78) | 118.74 (76.86-173.98) | 1.94 (1.26-2.84) | 9447.71 (7177.5-12271.26) | 154.48 (117.36-200.65) | 40667.34 (36313.43-45410.13) | 3279.96 (2928.8-3662.48) | 1867.15 (1542.24-2279.49) | 150.59 (124.39-183.85) | 34581.06 (28537.33-41355.9) | 1540.61 (1271.35-1842.43) |
| Serbia | 23934.41 (19672.97-29211.87) | 595.56 (489.52-726.88) | 169.01 (122.67-231.15) | 4.21 (3.05-5.75) | 11024.81 (8718.15-13851.69) | 274.33 (216.93-344.67) | 161757.04 (136111.14-192624.28) | 5910 (4973-7037.78) | 19940.3 (16824.03-23465.61) | 728.54 (614.69-857.35) | 302326.55 (256018.96-356550.53) | 5301.04 (4489.07-6251.81) |
| Seychelles | 300.72 (265.49-341.14) | 554.86 (489.85-629.44) | 1.68 (1.17-2.27) | 3.1 (2.15-4.2) | 125.74 (99.11-156.87) | 232 (182.86-289.44) | 837.21 (750.99-934.11) | 4365.8 (3916.21-4871.12) | 32.92 (27.38-39.09) | 171.66 (142.76-203.86) | 642.93 (546.53-755.95) | 1870.76 (1590.25-2199.62) |
| Sierra Leone | 19499.06 (17145.69-21899.62) | 565.43 (497.19-635.05) | 91.59 (57.65-140.07) | 2.66 (1.67-4.06) | 6750.84 (4934.02-9139.45) | 195.76 (143.08-265.03) | 21757.39 (19358.97-24416.5) | 3838.84 (3415.67-4308.01) | 1004.74 (772.78-1304.18) | 177.27 (136.35-230.11) | 19107.38 (14895.68-24169.46) | 1838.97 (1433.62-2326.16) |
| Singapore | 12803.92 (10865.61-15009.65) | 395.07 (335.26-463.12) | 8.99 (7.46-10.77) | 0.28 (0.23-0.33) | 2345.76 (1694.96-3034.27) | 72.38 (52.3-93.62) | 43067.8 (38350.59-48106.2) | 3097.13 (2757.9-3459.46) | 881.4 (722.07-995.12) | 63.38 (51.93-71.56) | 18151.06 (15522.82-20555.2) | 693.01 (592.66-784.8) |
| Slovakia | 13891.8 (12088.49-15893.62) | 511.43 (445.04-585.12) | 72.38 (51.91-96.85) | 2.66 (1.91-3.57) | 5422.09 (4289.2-6607.57) | 199.61 (157.91-243.26) | 70673.17 (63381.2-78217.84) | 4397.83 (3944.07-4867.32) | 4245.63 (3428.32-5152.04) | 264.2 (213.34-320.6) | 73459.39 (60814.97-87605.19) | 2269.46 (1878.82-2706.48) |
| Slovenia | 3405.81 (2869.1-4021.63) | 355.68 (299.63-419.99) | 6.05 (4.29-8.68) | 0.63 (0.45-0.91) | 838.13 (631.22-1069.92) | 87.53 (65.92-111.73) | 19421.78 (17317.1-21734.68) | 2720.74 (2425.9-3044.75) | 1486.29 (1130.08-1934.18) | 208.21 (158.31-270.95) | 20359.71 (16445.58-25228.19) | 1325.42 (1070.61-1642.36) |
| Solomon Islands | 1318.64 (1160.68-1484.46) | 466.31 (410.45-524.95) | 20.48 (13.53-31) | 7.24 (4.78-10.96) | 1136.47 (822.5-1589.63) | 401.89 (290.86-562.14) | 1647.94 (1469-1859.16) | 3502.57 (3122.26-3951.51) | 114.66 (88.85-147.81) | 243.7 (188.85-314.17) | 2486.74 (1992.1-3164.01) | 3190.9 (2556.2-4059.95) |
| Somalia | 36261.22 (31519.26-41386.07) | 481.85 (418.84-549.95) | 131.69 (77.06-212.02) | 1.75 (1.02-2.82) | 10420.71 (7626.88-14280.75) | 138.47 (101.35-189.77) | 32707.44 (29079.04-36772.45) | 3212.98 (2856.55-3612.3) | 1702.25 (1166.02-2495.09) | 167.22 (114.54-245.1) | 35802.56 (25660.13-51385.4) | 2074.88 (1487.09-2977.96) |
| South Africa | 169414.27 (139140.82-203111.28) | 595.67 (489.23-714.15) | 395.6 (340.76-450.21) | 1.39 (1.2-1.58) | 38401.21 (31629.18-45769.42) | 135.02 (111.21-160.93) | 336145.56 (280772.6-405083.45) | 4444.56 (3712.41-5356.06) | 16298.16 (14716.82-17468.84) | 215.5 (194.59-230.98) | 285278.97 (259239.67-308055.17) | 2056.9 (1869.15-2221.12) |
| South Sudan | 19451.55 (16884.71-22216.68) | 575.93 (499.93-657.8) | 39.33 (20.42-78.55) | 1.16 (0.6-2.33) | 4122.96 (2997.45-5893.71) | 122.07 (88.75-174.5) | 24129.78 (21606.02-27045.25) | 3959.17 (3545.08-4437.53) | 888.74 (569.58-1293.08) | 145.82 (93.46-212.17) | 17311.49 (12121.77-25166.39) | 1638.76 (1147.48-2382.33) |
| Spain | 76455.28 (65032.79-89327.13) | 347.69 (295.74-406.22) | 87.47 (71.14-105.31) | 0.4 (0.32-0.48) | 13129.7 (9999.88-16542.49) | 59.71 (45.48-75.23) | 354217.85 (316052.42-395005.71) | 2362.59 (2108.03-2634.64) | 23167.38 (19037.47-26737.33) | 154.52 (126.98-178.33) | 276002.69 (239637.81-306863.28) | 824.38 (715.76-916.55) |
| Sri Lanka | 47297.54 (41151.28-54324.39) | 451.66 (392.97-518.76) | 222.51 (140.64-330.64) | 2.12 (1.34-3.16) | 18141.27 (13353.94-23838.98) | 173.24 (127.52-227.65) | 171086.79 (152397.86-193555.02) | 3754.76 (3344.6-4247.86) | 8763.22 (6616.81-11274.97) | 192.32 (145.22-247.45) | 158189.47 (125287.31-198030.75) | 1854.03 (1468.41-2320.99) |
| Sudan | 122733.84 (109679.37-136253.19) | 695.6 (621.61-772.22) | 1250.79 (740.78-1976.08) | 7.09 (4.2-11.2) | 72927.08 (48166.48-106905.5) | 413.32 (272.98-605.89) | 180793.08 (159488.05-205715.9) | 6210.76 (5478.87-7066.93) | 11720.45 (8732.9-17014.92) | 402.63 (300-584.51) | 217170.25 (166672.54-309589.74) | 4079.11 (3130.61-5815.03) |
| Suriname | 1977.96 (1735-2252.71) | 711.28 (623.91-810.08) | 8.94 (6.66-11.87) | 3.22 (2.4-4.27) | 536.3 (431.83-666.46) | 192.86 (155.29-239.66) | 3868.26 (3455.32-4333.84) | 3715.61 (3318.97-4162.82) | 240.3 (200.99-281.48) | 230.82 (193.06-270.37) | 3902.49 (3290.77-4549.71) | 2011 (1695.77-2344.52) |
| Sweden | 32952.66 (26788.37-39960.39) | 718.06 (583.74-870.76) | 18.9 (16.5-21.42) | 0.41 (0.36-0.47) | 4858.58 (3528.26-6323.92) | 105.87 (76.88-137.8) | 111762.79 (95323.96-132878.75) | 3424.62 (2920.91-4071.65) | 5929.08 (5043.53-6741.6) | 181.68 (154.54-206.58) | 76421.61 (68098.61-84555.26) | 1028.73 (916.7-1138.22) |
| Switzerland | 11802.88 (9941.95-13944.71) | 278.68 (234.74-329.25) | 11.19 (8.98-13.78) | 0.26 (0.21-0.33) | 1931.65 (1451.11-2481.16) | 45.61 (34.26-58.58) | 58439.46 (52337.98-65363.66) | 2088.7 (1870.63-2336.18) | 3437.43 (2765.86-4029.13) | 122.86 (98.86-144.01) | 42243.68 (36154.6-47697.62) | 692.88 (593-782.33) |
| Syrian Arab Republic | 46834.56 (41504.98-52565.01) | 726.1 (643.47-814.94) | 343.82 (237.03-490.42) | 5.33 (3.67-7.6) | 21057.93 (16152.27-27368.16) | 326.47 (250.42-424.3) | 102398.28 (91421-115569.31) | 4749.58 (4240.42-5360.5) | 4586.32 (3575.99-5833.87) | 212.73 (165.87-270.59) | 92639.68 (73188.97-115837.6) | 2473.06 (1953.81-3092.34) |
| Taiwan (Province of China) | 58085.55 (50231.55-66847.71) | 474.01 (409.92-545.51) | 106.13 (74.57-144.79) | 0.87 (0.61-1.18) | 15343.28 (11646.18-19358.81) | 125.21 (95.04-157.98) | 269549.34 (243427.91-297964.83) | 3798.8 (3430.67-4199.26) | 6681.07 (5272.73-8223.02) | 94.16 (74.31-115.89) | 139183.22 (116189.05-165106.47) | 1009.49 (842.71-1197.51) |
| Tajikistan | 13143.41 (11236.42-15384.87) | 292.17 (249.78-341.99) | 36.83 (25.84-56.24) | 0.82 (0.57-1.25) | 3864.28 (2951.14-4992.52) | 85.9 (65.6-110.98) | 11823.12 (10311-13469.13) | 1349.85 (1177.21-1537.77) | 1013.24 (827.51-1260.78) | 115.68 (94.48-143.94) | 19749.63 (16035.84-24889.4) | 1474.16 (1196.96-1857.81) |
| Thailand | 166243.2 (145840.5-190384.78) | 448.98 (393.88-514.18) | 987.72 (678.6-1374.96) | 2.67 (1.83-3.71) | 72336.52 (55512.61-91151.78) | 195.36 (149.92-246.18) | 565225.52 (501978.95-628554.39) | 3118.14 (2769.23-3467.5) | 19245.61 (14462.25-25346.95) | 106.17 (79.78-139.83) | 388896.5 (308080.4-485438.32) | 1128.32 (893.84-1408.41) |
| Timor-Leste | 2419.37 (2122.91-2750.03) | 453.43 (397.87-515.4) | 15.03 (6.49-25.59) | 2.82 (1.22-4.8) | 1084.29 (680.62-1538.51) | 203.22 (127.56-288.34) | 7035 (6204.47-7961.24) | 5164.31 (4554.63-5844.26) | 408.65 (299.29-587.08) | 299.99 (219.71-430.97) | 8025.09 (6095.44-11103.09) | 3146.16 (2389.66-4352.86) |
| Togo | 18719.23 (16418.06-21268.18) | 553.65 (485.59-629.04) | 90.54 (57.78-129.57) | 2.68 (1.71-3.83) | 6494.4 (4844.81-8399.8) | 192.08 (143.29-248.44) | 20299.8 (18068.61-22641.1) | 3475.81 (3093.78-3876.7) | 829.95 (676.14-1022.44) | 142.11 (115.77-175.07) | 17191.06 (14194.12-20973.2) | 1716.58 (1417.32-2094.24) |
| Tokelau | 3.26 (2.87-3.7) | 551.7 (486.53-626.11) | 0.02 (0.02-0.03) | 4.06 (2.68-5.77) | 1.65 (1.23-2.14) | 278.68 (208.54-361.95) | 9.5 (8.53-10.58) | 4071.39 (3655.44-4534.55) | 0.44 (0.36-0.53) | 186.9 (153.24-227.52) | 8.34 (7.04-9.94) | 1877.91 (1584.67-2238.46) |
| Tonga | 210.79 (183.93-242.04) | 491.64 (429.01-564.54) | 0.78 (0.53-1.11) | 1.81 (1.23-2.6) | 72.38 (54.91-92.43) | 168.83 (128.07-215.58) | 505.48 (454.16-567.65) | 3871.04 (3478.05-4347.19) | 21.82 (17.51-26.47) | 167.09 (134.06-202.71) | 403.84 (336.49-473.92) | 1573.83 (1311.37-1846.93) |
| Trinidad and Tobago | 5341.68 (4659.23-6077.61) | 772.3 (673.63-878.7) | 18.37 (12.28-26.36) | 2.66 (1.78-3.81) | 1228.78 (928.14-1576.9) | 177.66 (134.19-227.99) | 12264.01 (11001.79-13759.42) | 3668.66 (3291.08-4116) | 699.59 (540.39-884.68) | 209.28 (161.65-264.64) | 10992.69 (8681.11-13800.38) | 1725.79 (1362.89-2166.58) |
| Tunisia | 34148.34 (29819.52-38563.41) | 577.08 (503.92-651.69) | 257.02 (171.52-363.94) | 4.34 (2.9-6.15) | 15743.76 (11656.13-20722.23) | 266.06 (196.98-350.19) | 104708.64 (92946.87-117041.5) | 4754.53 (4220.46-5314.53) | 6485.83 (4846.97-8304.2) | 294.5 (220.09-377.07) | 111553.64 (86680.67-141457.76) | 2714.13 (2108.97-3441.71) |
| Turkey | 223395.75 (195041.28-256994.71) | 508.74 (444.17-585.26) | 673.3 (490.19-897.66) | 1.53 (1.12-2.04) | 58864.63 (47072.01-72729.04) | 134.05 (107.2-165.63) | 592740.81 (533744.33-661610.82) | 3911.56 (3522.23-4366.04) | 29528.12 (23555.08-35932.63) | 194.86 (155.44-237.12) | 488020.25 (404936.36-579592.09) | 1685.45 (1398.51-2001.71) |
| Turkmenistan | 14818.65 (13069.53-16746.75) | 594.7 (524.51-672.08) | 212.43 (151.64-290.27) | 8.53 (6.09-11.65) | 11629.86 (8964.56-14968.28) | 466.73 (359.77-600.71) | 25444.12 (22499.45-28741.13) | 3766.45 (3330.55-4254.5) | 2356.32 (1887.92-2884.43) | 348.8 (279.47-426.98) | 48428.64 (38581.09-59558.29) | 4235.1 (3373.93-5208.39) |
| Tuvalu | 27.73 (24.52-31.36) | 510.04 (451.01-576.88) | 0.28 (0.19-0.4) | 5.21 (3.42-7.32) | 17.58 (12.92-23.04) | 323.32 (237.62-423.83) | 71.3 (64.18-79.99) | 3999.82 (3600.49-4487.54) | 3.67 (2.94-4.61) | 205.76 (164.86-258.36) | 74.03 (60.59-90.58) | 2279.2 (1865.34-2788.79) |
| Uganda | 69818.11 (59948.26-80554.36) | 466.19 (400.29-537.88) | 153.01 (94.69-243.74) | 1.02 (0.63-1.63) | 15547.33 (11627.82-20252.88) | 103.81 (77.64-135.23) | 79481.95 (71274.94-88479.79) | 3574.5 (3205.41-3979.16) | 3571.1 (2682.32-4410.72) | 160.6 (120.63-198.36) | 67134.11 (53047.98-81620.44) | 1702.29 (1345.11-2069.61) |
| Ukraine | 157510.11 (129194.87-192826.72) | 728.79 (597.78-892.2) | 1880.5 (1452.78-2380.45) | 8.7 (6.72-11.01) | 105938.38 (86199.05-129084.44) | 490.17 (398.84-597.27) | 533264.89 (447072.19-628789.24) | 3958.67 (3318.82-4667.79) | 70220.77 (60442.83-80271.31) | 521.28 (448.69-595.89) | 1147298.18 (1001466.18-1307004.08) | 4219.21 (3682.91-4806.53) |
| United Arab Emirates | 70959.57 (62947.16-79936.15) | 987.79 (876.25-1112.75) | 538.97 (351.9-776.34) | 7.5 (4.9-10.81) | 32860.47 (24357.21-43887.09) | 457.43 (339.06-610.93) | 35758.51 (31772.96-40279.5) | 6592.55 (5857.76-7426.06) | 698.19 (514.84-909.07) | 128.72 (94.92-167.6) | 21077.41 (16185.32-26544.31) | 3006.92 (2309.01-3786.83) |
| United Kingdom | 105362.71 (86023.31-127796.02) | 334.71 (273.28-405.98) | 157.01 (147.63-173.09) | 0.5 (0.47-0.55) | 19601.58 (15388.73-24089.52) | 62.27 (48.89-76.53) | 458379.67 (392860.55-540383.63) | 2268.82 (1944.52-2674.71) | 33489.05 (28827.78-35972.7) | 165.76 (142.69-178.05) | 409790.99 (366072.75-440304.17) | 924.29 (825.69-993.12) |
| United Republic of Tanzania | 116254.37 (100501.12-132877.83) | 525.12 (453.97-600.21) | 337.99 (207.71-603.26) | 1.53 (0.94-2.72) | 29584.04 (21759.84-41569.39) | 133.63 (98.29-187.77) | 148620.99 (132399.11-167081.3) | 3811.28 (3395.28-4284.68) | 8481.45 (6312.18-11123.32) | 217.5 (161.87-285.25) | 155767.95 (121503.35-203902.18) | 2198.51 (1714.9-2877.87) |
| United States of America | 923318.94 (742561.01-1130638.51) | 616.57 (495.86-755.01) | 870.68 (814.82-955.3) | 0.58 (0.54-0.64) | 158040.14 (119671.62-202713.14) | 105.53 (79.91-135.37) | 4838167.88 (4175377.97-5597595.64) | 5017.07 (4329.77-5804.57) | 108045.64 (91547.49-119358.97) | 112.04 (94.93-123.77) | 1880447.59 (1642021.96-2106921.88) | 946.86 (826.81-1060.9) |
| United States Virgin Islands | 273.02 (236.62-311.77) | 613.82 (531.98-700.95) | 0.83 (0.56-1.16) | 1.87 (1.26-2.61) | 55.37 (42.82-70.3) | 124.49 (96.27-158.04) | 937 (829.27-1044.86) | 2837.72 (2511.46-3164.36) | 48.31 (41.8-54.63) | 146.31 (126.6-165.45) | 803.3 (700.75-902.63) | 1201.68 (1048.27-1350.27) |
| Uruguay | 6178.07 (5224.32-7206.23) | 387.32 (327.53-451.78) | 21.39 (17.65-25.51) | 1.34 (1.11-1.6) | 1737.43 (1407.94-2083.3) | 108.93 (88.27-130.61) | 29081.51 (25636.43-33270.22) | 3269.99 (2882.62-3740.98) | 2356.44 (2003.4-2608.78) | 264.96 (225.27-293.34) | 32033.25 (28577.47-34955.21) | 1666.39 (1486.62-1818.39) |
| Uzbekistan | 85076.16 (71749.33-101388.66) | 505.66 (426.45-602.62) | 1100.21 (873.99-1392.53) | 6.54 (5.19-8.28) | 60701.83 (50193.52-74006.9) | 360.79 (298.33-439.87) | 117253.45 (100517.2-136409.27) | 3165.14 (2713.36-3682.23) | 9836.71 (8368.69-11512.07) | 265.53 (225.9-310.76) | 226371.01 (192096.87-263927.28) | 4097.73 (3477.3-4777.56) |
| Vanuatu | 793.64 (703.64-894.63) | 625.51 (554.58-705.11) | 7.88 (5.02-12.01) | 6.21 (3.95-9.47) | 499.36 (361.56-690.32) | 393.57 (284.97-544.08) | 1461.37 (1299.58-1651.72) | 5166.01 (4594.08-5838.9) | 66.9 (50.79-94.87) | 236.49 (179.56-335.38) | 1456.06 (1158.77-1928.43) | 2853.17 (2270.61-3778.78) |
| Venezuela (Bolivarian Republic of) | 67417.55 (58360.79-78301.93) | 491.01 (425.05-570.29) | 96.39 (66.31-134.3) | 0.7 (0.48-0.98) | 9641.06 (7509.18-12075.13) | 70.22 (54.69-87.95) | 129922.38 (116524.27-145106.58) | 2578.01 (2312.15-2879.3) | 6516.52 (4996.81-8196.73) | 129.31 (99.15-162.65) | 98958.37 (79029.34-122995.66) | 1039.31 (830-1291.76) |
| Viet Nam | 251936.41 (221472.6-287762.26) | 483.03 (424.62-551.72) | 2073.09 (1331.82-3049.49) | 3.97 (2.55-5.85) | 131669 (95589.57-174750.76) | 252.45 (183.27-335.04) | 700002.88 (621719.87-796677.99) | 4336.38 (3851.43-4935.26) | 60278.62 (48468.74-71005.75) | 373.41 (300.25-439.87) | 1025201.23 (831484.44-1208503.73) | 3603.53 (2922.62-4247.83) |
| Yemen | 77410.85 (69233.23-86464.08) | 591.91 (529.38-661.13) | 880.76 (578.41-1275.62) | 6.73 (4.42-9.75) | 49612.27 (35552.52-66739.54) | 379.35 (271.85-510.31) | 116820.64 (102928.46-132068.97) | 5536.3 (4877.93-6258.94) | 7986.91 (6110.48-10480.22) | 378.51 (289.58-496.67) | 155978.54 (123769.24-202650.52) | 4145.61 (3289.55-5386.06) |
| Zambia | 35075.3 (30187.16-40134.52) | 472.22 (406.41-540.33) | 128.73 (79.27-201.47) | 1.73 (1.07-2.71) | 10043.55 (7531.14-13413.44) | 135.22 (101.39-180.59) | 34870.92 (31272.26-38959.85) | 3411.69 (3059.61-3811.75) | 2533.8 (1996.37-3296.89) | 247.9 (195.32-322.56) | 46246.03 (36854.91-60082.53) | 2556.32 (2037.21-3321.16) |
| Zimbabwe | 32043.16 (27943.39-36422.53) | 510.58 (445.26-580.37) | 154.67 (102.93-225.42) | 2.46 (1.64-3.59) | 10858.62 (8326.81-14294.48) | 173.02 (132.68-227.77) | 44650.89 (39920.36-50006.15) | 4007.92 (3583.3-4488.62) | 2594.99 (2057.43-3246.97) | 232.93 (184.68-291.45) | 49599.09 (40186.99-61757.83) | 2503.41 (2028.35-3117.1) |

Table S10 The PAD burden in 204 countries and territories by the year 2019.

|  | 20-54 age group | | | | | | 55+ age group | | | | | |
| --- | --- | --- | --- | --- | --- | --- | --- | --- | --- | --- | --- | --- |
|  | Prevalence | | Death | | DALYs | | Prevalence | | Death | | DALYs | |
| location | Number in 2019 | Rate in 2019 | Number in 2019 | Rate in 2019 | Number in 2019 | Rate in 2019 | Number in 2019 | Rate in 2019 | Number in 2019 | Rate in 2019 | Number in 2019 | Rate in 2019 |
| Afghanistan | 36245.32 (28350.18-45404.57) | 241.05 (188.55-301.97) | 1.02 (0.58-1.61) | 0.01 (0-0.01) | 63.23 (39.63-97.56) | 0.42 (0.26-0.65) | 80313.46 (68071.2-93623.63) | 4902.89 (4155.54-5715.44) | 8.91 (5.71-13.63) | 0.54 (0.35-0.83) | 645.16 (363.73-1026.01) | 21.19 (11.95-33.71) |
| Albania | 5207.15 (4053.81-6549.14) | 405.23 (315.47-509.66) | 0.17 (0.1-0.26) | 0.01 (0.01-0.02) | 9.37 (5.97-13.75) | 0.73 (0.46-1.07) | 47689.58 (40656.24-55088.58) | 6196.93 (5282.99-7158.37) | 8.12 (5.72-11.1) | 1.05 (0.74-1.44) | 354.55 (228.42-550.57) | 23.32 (15.03-36.22) |
| Algeria | 62657.44 (48797.13-78051.96) | 292.11 (227.49-363.87) | 2.64 (1.55-4.19) | 0.01 (0.01-0.02) | 137.64 (87.12-208.19) | 0.64 (0.41-0.97) | 291645.87 (246393.54-338981.64) | 5191.29 (4385.8-6033.86) | 72.91 (45.67-111.82) | 1.3 (0.81-1.99) | 2565.96 (1588.13-3863.09) | 24.89 (15.4-37.47) |
| American Samoa | 145.09 (112.86-181.6) | 590.91 (459.62-739.61) | 0.01 (0.01-0.01) | 0.04 (0.02-0.06) | 0.45 (0.29-0.69) | 1.85 (1.19-2.82) | 635.38 (538.92-737.57) | 7669.49 (6505.12-8902.98) | 0.12 (0.1-0.15) | 1.46 (1.17-1.83) | 5.9 (3.86-8.75) | 39.8 (26.02-59.03) |
| Andorra | 387.67 (307.45-477.65) | 877.34 (695.78-1080.97) | 0.04 (0.02-0.07) | 0.1 (0.05-0.16) | 1.87 (0.99-3.1) | 4.24 (2.24-7.01) | 2285.24 (1958.64-2626.39) | 9528.01 (8166.3-10950.41) | 2.8 (1.72-4) | 11.68 (7.17-16.69) | 41.43 (27.36-57.78) | 86.48 (57.12-120.62) |
| Angola | 25123.02 (19519.92-31187.73) | 222.72 (173.05-276.48) | 10.27 (5.56-16.4) | 0.09 (0.05-0.15) | 425.7 (240.9-675.53) | 3.77 (2.14-5.99) | 89356.82 (75388.93-104385.17) | 4957.28 (4182.38-5791.01) | 105.37 (57.49-146.84) | 5.85 (3.19-8.15) | 2563.74 (1542.77-3493.5) | 86.5 (52.06-117.88) |
| Antigua and Barbuda | 135.85 (105.11-170.24) | 288.44 (223.17-361.47) | 0.08 (0.03-0.16) | 0.17 (0.07-0.34) | 3.2 (1.44-6.35) | 6.79 (3.07-13.48) | 811.34 (687.09-939.55) | 4595.42 (3891.71-5321.64) | 1.88 (0.91-3.59) | 10.66 (5.14-20.34) | 32.17 (17.03-57.02) | 98.02 (51.9-173.72) |
| Argentina | 106231.38 (83732.75-131097.03) | 485.05 (382.33-598.59) | 3.9 (1.84-7.33) | 0.02 (0.01-0.03) | 213.16 (119.39-358.7) | 0.97 (0.55-1.64) | 742306.87 (629007.55-862156.03) | 8191.44 (6941.17-9513.99) | 253.59 (121.83-496.67) | 2.8 (1.34-5.48) | 7033.51 (4238.28-11438.64) | 37.61 (22.66-61.16) |
| Armenia | 5811.28 (4522.23-7336.9) | 392.38 (305.34-495.39) | 0.36 (0.15-0.82) | 0.02 (0.01-0.06) | 17.22 (8.4-36.43) | 1.16 (0.57-2.46) | 51723.78 (44163.17-59690.98) | 6770.81 (5781.1-7813.74) | 11.41 (5.2-20.87) | 1.49 (0.68-2.73) | 428.25 (258.88-698.54) | 29.69 (17.95-48.43) |
| Australia | 52271.29 (41272.3-64386.5) | 449.39 (354.83-553.55) | 6.23 (2.96-12.68) | 0.05 (0.03-0.11) | 267.3 (134.89-513.32) | 2.3 (1.16-4.41) | 475756.49 (406399.37-547355.65) | 6880.16 (5877.15-7915.59) | 1252.38 (595.04-2349.08) | 18.11 (8.61-33.97) | 15392.02 (8320.66-27801.22) | 104.39 (56.43-188.56) |
| Austria | 35686.45 (28352.31-43551.49) | 831.16 (660.34-1014.34) | 6.41 (2.79-14.53) | 0.15 (0.07-0.34) | 269.2 (125.83-589.77) | 6.27 (2.93-13.74) | 312509.21 (266542.79-361356.3) | 10853.95 (9257.46-12550.48) | 725.62 (347.24-1375.21) | 25.2 (12.06-47.76) | 9632.79 (5159.67-17416.26) | 153.81 (82.39-278.1) |
| Azerbaijan | 19777.5 (15373.54-24826.2) | 355.92 (276.66-446.77) | 0.23 (0.17-0.32) | 0 (0-0.01) | 21.15 (13.11-33.04) | 0.38 (0.24-0.59) | 86531.01 (73996.46-101427.21) | 5017 (4290.25-5880.67) | 4.38 (3.22-5.66) | 0.25 (0.19-0.33) | 500.29 (269.62-854.2) | 18.22 (9.82-31.11) |
| Bahamas | 538.4 (419.16-673.66) | 270.76 (210.8-338.78) | 0.47 (0.2-0.87) | 0.24 (0.1-0.44) | 18.87 (8.3-34.65) | 9.49 (4.17-17.42) | 2947.95 (2503.26-3402.79) | 4362.61 (3704.52-5035.71) | 8.46 (4.1-16.19) | 12.53 (6.07-23.96) | 139.11 (74.71-248.29) | 113.99 (61.22-203.47) |
| Bahrain | 3324.78 (2631.99-4126.06) | 354.3 (280.48-439.69) | 0.62 (0.37-0.93) | 0.07 (0.04-0.1) | 26.22 (16.59-39.47) | 2.79 (1.77-4.21) | 7624.98 (6419.75-8868.73) | 4155.29 (3498.49-4833.08) | 6.13 (4.57-8.03) | 3.34 (2.49-4.37) | 151.6 (111.27-199.2) | 57.58 (42.26-75.67) |
| Bangladesh | 165445.62 (128655.45-209043.47) | 211.99 (164.85-267.85) | 11.74 (6.22-18.69) | 0.02 (0.01-0.02) | 559.15 (332.07-852.49) | 0.72 (0.43-1.09) | 857258.32 (730950.38-998099.73) | 3923.66 (3345.55-4568.29) | 325.93 (173.06-479.45) | 1.49 (0.79-2.19) | 9735.48 (5884.47-14526.56) | 23.41 (14.15-34.93) |
| Barbados | 455.44 (353.6-570.94) | 321.42 (249.55-402.93) | 0.69 (0.32-1.33) | 0.49 (0.23-0.94) | 27.5 (12.68-52.44) | 19.41 (8.95-37.01) | 4402.28 (3747.53-5125.67) | 5074.2 (4319.52-5908.01) | 33.87 (16.14-62.71) | 39.04 (18.61-72.29) | 479.66 (243.68-854.96) | 277.03 (140.74-493.79) |
| Belarus | 21773.59 (17022.02-27132.59) | 478.68 (374.22-596.49) | 3.55 (1.16-9.02) | 0.08 (0.03-0.2) | 149.16 (55.59-366.76) | 3.28 (1.22-8.06) | 213528.7 (182304.41-246274.67) | 7453.11 (6363.24-8596.09) | 171.22 (65.13-364.37) | 5.98 (2.27-12.72) | 3556.35 (1832.01-6766.27) | 63.32 (32.62-120.47) |
| Belgium | 46286.93 (36908.76-56894.59) | 892.68 (711.81-1097.25) | 2.52 (1.11-5.11) | 0.05 (0.02-0.1) | 121.75 (62.18-228.55) | 2.35 (1.2-4.41) | 440853.58 (375610.51-509773.28) | 11994.4 (10219.32-13869.51) | 326.78 (152.25-621.25) | 8.89 (4.14-16.9) | 5619.73 (3381.87-9225.23) | 69.71 (41.95-114.43) |
| Belize | 424.81 (334.75-530.84) | 215.45 (169.77-269.22) | 0.07 (0.03-0.14) | 0.03 (0.02-0.07) | 2.93 (1.41-5.58) | 1.49 (0.72-2.83) | 2053.08 (1746.58-2382.03) | 4489.34 (3819.15-5208.65) | 1.28 (0.6-2.5) | 2.8 (1.32-5.46) | 30 (18.03-49.24) | 35.94 (21.61-59) |
| Benin | 8220.41 (6384.77-10330.22) | 171.39 (133.12-215.38) | 0.21 (0.13-0.32) | 0 (0-0.01) | 13.59 (8.92-19.64) | 0.28 (0.19-0.41) | 33856.16 (28859.8-39549.35) | 4414.06 (3762.65-5156.32) | 11.58 (6.04-16.89) | 1.51 (0.79-2.2) | 423.35 (258.69-624.83) | 30.47 (18.62-44.98) |
| Bermuda | 99.9 (77.79-125.1) | 335.79 (261.45-420.49) | 0.09 (0.04-0.19) | 0.29 (0.13-0.63) | 3.46 (1.52-7.39) | 11.64 (5.12-24.83) | 1152.76 (985.84-1332.43) | 5151.1 (4405.19-5953.92) | 5.26 (2.47-9.64) | 23.49 (11.03-43.1) | 67.93 (35.98-120.89) | 147.08 (77.89-261.73) |
| Bhutan | 744.71 (577.94-934.91) | 187.82 (145.76-235.78) | 0.05 (0.02-0.09) | 0.01 (0.01-0.02) | 2.41 (1.3-4.05) | 0.61 (0.33-1.02) | 3769.58 (3191.07-4366.84) | 4060.69 (3437.51-4704.08) | 1.79 (1.1-2.76) | 1.93 (1.18-2.97) | 47.85 (30.4-70.67) | 27.31 (17.35-40.34) |
| Bolivia (Plurinational State of) | 10561.83 (8225.18-13313.86) | 191.18 (148.89-241) | 0.53 (0.28-0.9) | 0.01 (0.01-0.02) | 27.04 (15.87-43.12) | 0.49 (0.29-0.78) | 61071.75 (51650.09-70914.79) | 4092.88 (3461.46-4752.54) | 12 (7.73-16.96) | 0.8 (0.52-1.14) | 528.54 (322.86-825.02) | 18.9 (11.55-29.5) |
| Bosnia and Herzegovina | 8679.77 (6797.03-10920.25) | 551.66 (432-694.05) | 0.5 (0.31-0.78) | 0.03 (0.02-0.05) | 24.11 (15.55-35.81) | 1.53 (0.99-2.28) | 80706.64 (68419.58-93656.92) | 7594.01 (6437.87-8812.56) | 24.9 (18.51-32.25) | 2.34 (1.74-3.03) | 767.8 (537.3-1089.44) | 36.58 (25.6-51.9) |
| Botswana | 2950.36 (2295.93-3716.05) | 246.7 (191.98-310.73) | 2.13 (1.11-3.56) | 0.18 (0.09-0.3) | 87.83 (46.72-144.72) | 7.34 (3.91-12.1) | 12533.01 (10633.95-14698.42) | 5592.02 (4744.69-6558.19) | 11.82 (7.34-16.85) | 5.27 (3.27-7.52) | 308.38 (203.89-425.34) | 80.19 (53.02-110.61) |
| Brazil | 271378.39 (211803.7-340107.84) | 242.93 (189.6-304.45) | 125.28 (56.39-242.24) | 0.11 (0.05-0.22) | 5140.91 (2399.1-9799.92) | 4.6 (2.15-8.77) | 1905421.28 (1627267.26-2203813.42) | 4675.41 (3992.89-5407.59) | 2645.13 (1199.01-4914.22) | 6.49 (2.94-12.06) | 48962.32 (26456.11-83548.16) | 62.48 (33.76-106.61) |
| Brunei Darussalam | 1066.23 (832.64-1327.42) | 420.34 (328.26-523.31) | 0.08 (0.06-0.12) | 0.03 (0.02-0.05) | 3.93 (2.85-5.52) | 1.55 (1.12-2.18) | 2908.21 (2470.97-3371.36) | 5582.83 (4743.45-6471.93) | 1.48 (1.18-1.87) | 2.84 (2.26-3.59) | 37 (28.18-49.02) | 44.58 (33.95-59.06) |
| Bulgaria | 15577.41 (12194.63-19381.77) | 487.86 (381.92-607.01) | 2.28 (0.88-5.1) | 0.07 (0.03-0.16) | 98.32 (42.68-209.47) | 3.08 (1.34-6.56) | 177081.39 (149216.53-206139.09) | 7269.57 (6125.66-8462.45) | 87.74 (41.48-171.75) | 3.6 (1.7-7.05) | 2234.46 (1340.59-3792.2) | 42.28 (25.37-71.76) |
| Burkina Faso | 14060.43 (10950.49-17590.71) | 166.3 (129.52-208.06) | 0.43 (0.28-0.62) | 0.01 (0-0.01) | 26.18 (17.63-37.58) | 0.31 (0.21-0.44) | 57361.22 (48845.68-67172.44) | 3911.17 (3330.54-4580.15) | 29.93 (14.06-50.65) | 2.04 (0.96-3.45) | 956.18 (556.77-1474.65) | 36.81 (21.44-56.78) |
| Burundi | 7452.54 (5839.08-9286.2) | 164.61 (128.97-205.11) | 2.45 (1.19-4.29) | 0.05 (0.03-0.09) | 103.7 (53.59-175.85) | 2.29 (1.18-3.88) | 28680.88 (24318.58-33604.72) | 3841.24 (3257-4500.7) | 29.42 (14.54-43.72) | 3.94 (1.95-5.86) | 718.94 (438.25-1014.92) | 57.56 (35.08-81.25) |
| Cabo Verde | 592.41 (462.16-743.62) | 209.41 (163.36-262.85) | 0.01 (0.01-0.02) | 0 (0-0.01) | 0.89 (0.61-1.32) | 0.31 (0.22-0.47) | 3526.57 (3026.27-4083.53) | 4923.86 (4225.33-5701.49) | 2.35 (1.32-3.14) | 3.28 (1.84-4.39) | 54.63 (34.38-76.64) | 40.73 (25.63-57.14) |
| Cambodia | 30340.13 (23870.96-38276.72) | 376.46 (296.19-474.94) | 0.84 (0.52-1.32) | 0.01 (0.01-0.02) | 52.68 (33.02-80.5) | 0.65 (0.41-1) | 129631.66 (110058.05-150622.91) | 6392.27 (5427.07-7427.37) | 12.57 (8.5-18.88) | 0.62 (0.42-0.93) | 1020.37 (566.95-1663.27) | 28.43 (15.79-46.34) |
| Cameroon | 19197.39 (14955.84-24221.77) | 160.75 (125.23-202.82) | 0.71 (0.41-1.12) | 0.01 (0-0.01) | 40.07 (25.55-59.25) | 0.34 (0.21-0.5) | 75985.13 (64265.38-88870.93) | 3983.44 (3369.04-4658.96) | 37.48 (20.25-58.95) | 1.97 (1.06-3.09) | 1165.66 (713.33-1736.25) | 34.93 (21.37-52.02) |
| Canada | 138541.7 (110606.25-169305.04) | 833.91 (665.76-1019.08) | 22.69 (10.36-46.02) | 0.14 (0.06-0.28) | 968.93 (478.19-1874.79) | 5.83 (2.88-11.28) | 1359833.81 (1154650.7-1570754.47) | 11548.08 (9805.61-13339.28) | 1406.43 (684.33-2633.67) | 11.94 (5.81-22.37) | 23099.91 (13431.16-39643.68) | 94.38 (54.88-161.98) |
| Central African Republic | 4632.57 (3611.24-5813.16) | 216.27 (168.59-271.38) | 1.85 (0.92-3.46) | 0.09 (0.04-0.16) | 76.52 (39.37-142.02) | 3.57 (1.84-6.63) | 15016.51 (12686.44-17614.62) | 4428.31 (3741.18-5194.48) | 15.59 (8.19-24.28) | 4.6 (2.42-7.16) | 423.15 (264.58-619.09) | 77.77 (48.63-113.79) |
| Chad | 8523.6 (6691.28-10784.11) | 157.29 (123.48-199) | 0.24 (0.15-0.37) | 0 (0-0.01) | 15.34 (9.79-22.96) | 0.28 (0.18-0.42) | 34185.97 (29230.96-39886.57) | 3843.24 (3286.19-4484.1) | 12.1 (6.09-18.82) | 1.36 (0.68-2.12) | 455.9 (273.07-672) | 28.55 (17.1-42.09) |
| Chile | 48945.27 (38505.23-60411.15) | 535.3 (421.12-660.7) | 4.24 (1.97-8.62) | 0.05 (0.02-0.09) | 194.74 (101.35-366.69) | 2.13 (1.11-4.01) | 323247.2 (273808.92-374516) | 7713.34 (6533.64-8936.72) | 283.64 (134.74-528.64) | 6.77 (3.22-12.61) | 5217.28 (2958.84-8802.29) | 63.1 (35.78-106.45) |
| China | 4781774.19 (3717221.53-6022235.85) | 620.45 (482.32-781.41) | 114.7 (91.06-142.95) | 0.01 (0.01-0.02) | 6755.32 (5036.59-9200.37) | 0.88 (0.65-1.19) | 23707862.47 (20154086.17-27409410.96) | 6740.09 (5729.76-7792.43) | 2094.62 (1721.51-2607.38) | 0.6 (0.49-0.74) | 158973.52 (90160.53-265778.73) | 23.79 (13.49-39.77) |
| Colombia | 58058.39 (45146.07-72582.73) | 245.23 (190.69-306.58) | 7.78 (3.22-17.54) | 0.03 (0.01-0.07) | 339.64 (159.54-735.53) | 1.43 (0.67-3.11) | 458976.75 (390998.76-531154.77) | 5048.61 (4300.87-5842.55) | 299.39 (129.54-620.02) | 3.29 (1.42-6.82) | 5999.89 (3519.6-10271.52) | 33.39 (19.59-57.16) |
| Comoros | 709.42 (548.3-890.08) | 214.46 (165.76-269.08) | 0.25 (0.12-0.41) | 0.07 (0.03-0.12) | 10.3 (4.85-16.9) | 3.11 (1.47-5.11) | 3603.03 (3058.56-4207.76) | 4574.75 (3883.44-5342.58) | 4.85 (2.18-7.24) | 6.16 (2.76-9.19) | 99.58 (53.97-142.71) | 66.55 (36.07-95.37) |
| Congo | 5671.26 (4429.81-7088.15) | 240.7 (188.01-300.83) | 3.71 (1.88-6.13) | 0.16 (0.08-0.26) | 151.68 (77.43-247.35) | 6.44 (3.29-10.5) | 19965.53 (16883.26-23356.65) | 4856.42 (4106.69-5681.28) | 40.66 (24.54-57.17) | 9.89 (5.97-13.91) | 872.76 (586.16-1213.7) | 123.91 (83.22-172.31) |
| Cook Islands | 49.43 (38.75-61.76) | 620.74 (486.65-775.64) | 0 (0-0.01) | 0.05 (0.03-0.09) | 0.21 (0.12-0.33) | 2.59 (1.55-4.16) | 345.58 (295.09-398.21) | 7727.84 (6598.78-8904.69) | 0.09 (0.07-0.12) | 2.05 (1.48-2.7) | 3.38 (2.23-5) | 39.3 (25.91-58.19) |
| Costa Rica | 5857.89 (4573.16-7375.14) | 243.53 (190.12-306.61) | 0.52 (0.22-1.1) | 0.02 (0.01-0.05) | 23.63 (11.63-47.16) | 0.98 (0.48-1.96) | 45059.79 (38282.4-52196.43) | 5036.58 (4279.03-5834.28) | 19.62 (8.67-38.11) | 2.19 (0.97-4.26) | 464.23 (284.12-780.59) | 26.91 (16.47-45.24) |
| Cte d'Ivoire | 19943.85 (15539.58-24871.44) | 178.63 (139.18-222.76) | 0.48 (0.3-0.72) | 0 (0-0.01) | 31.4 (20.3-46.07) | 0.28 (0.18-0.41) | 69506.72 (58858.74-81329.05) | 4134.31 (3500.96-4837.52) | 22 (12.57-34.42) | 1.31 (0.75-2.05) | 842.51 (521.03-1234.81) | 29.47 (18.23-43.2) |
| Croatia | 8840.97 (6867.9-10917.2) | 452.11 (351.21-558.29) | 3.89 (1.44-9.27) | 0.2 (0.07-0.47) | 157.25 (61.23-367.16) | 8.04 (3.13-18.78) | 102957.38 (87454.48-119515.36) | 7000.82 (5946.66-8126.72) | 215.04 (91.03-396.42) | 14.62 (6.19-26.96) | 3467.86 (1731.47-6290.64) | 110.24 (55.04-199.98) |
| Cuba | 21023.69 (16399.95-26295.67) | 372.65 (290.69-466.09) | 18.12 (7.55-38.26) | 0.32 (0.13-0.68) | 719.05 (307.7-1507.43) | 12.75 (5.45-26.72) | 171539.92 (146134.75-199451.27) | 5298.19 (4513.53-6160.26) | 558.58 (254.38-1018.45) | 17.25 (7.86-31.46) | 8381.39 (4269.71-14868.32) | 126.65 (64.52-224.66) |
| Cyprus | 5513.84 (4361.94-6762.4) | 782.48 (619.01-959.67) | 0.14 (0.1-0.2) | 0.02 (0.01-0.03) | 8.45 (6.03-11.59) | 1.2 (0.86-1.64) | 36959.72 (31099.37-43010.28) | 11191.66 (9417.11-13023.82) | 19.44 (11.64-24.72) | 5.89 (3.53-7.49) | 399.08 (282.57-547.98) | 59.11 (41.85-81.17) |
| Czechia | 24427.42 (19184.91-30417.56) | 487.02 (382.5-606.45) | 9.12 (3.58-20.84) | 0.18 (0.07-0.42) | 371.52 (151.95-843.61) | 7.41 (3.03-16.82) | 261346.69 (220980.93-305854.85) | 7575.2 (6405.19-8865.28) | 447.52 (211.12-903.09) | 12.97 (6.12-26.18) | 7664 (4140.53-14377.63) | 101.99 (55.1-191.34) |
| Democratic People's Republic of Korea | 80530.98 (62967.68-101998.07) | 569.67 (445.43-721.52) | 2.49 (1.4-4.02) | 0.02 (0.01-0.03) | 148.16 (93.48-226.05) | 1.05 (0.66-1.6) | 364356.94 (312037.53-423035.59) | 6757.11 (5786.83-7845.33) | 26.3 (18.58-33.73) | 0.49 (0.34-0.63) | 2744.8 (1508.15-4692.45) | 26.46 (14.54-45.23) |
| Democratic Republic of the Congo | 64162.08 (49679.29-80673.06) | 185.25 (143.44-232.92) | 48.52 (16.54-95.51) | 0.14 (0.05-0.28) | 1969.46 (717.79-3807.09) | 5.69 (2.07-10.99) | 250019.21 (210974.12-291085.78) | 4345.15 (3666.58-5058.86) | 540.69 (238.76-941.05) | 9.4 (4.15-16.35) | 11869.95 (5831.48-20083.71) | 121.6 (59.74-205.75) |
| Denmark | 30923.91 (24645.7-37972.26) | 1171.3 (933.5-1438.27) | 2.39 (1.1-4.95) | 0.09 (0.04-0.19) | 108.92 (57.39-210.06) | 4.13 (2.17-7.96) | 278355.59 (234375.09-324720.28) | 14970.61 (12605.24-17464.21) | 342.58 (163.37-640.31) | 18.43 (8.79-34.44) | 5199.08 (3044.56-8851.02) | 127.08 (74.42-216.35) |
| Djibouti | 1205.12 (937.01-1514.95) | 206.65 (160.68-259.78) | 0.56 (0.25-1) | 0.1 (0.04-0.17) | 23.1 (10.59-41.31) | 3.96 (1.82-7.08) | 3820.23 (3219.51-4484.76) | 4022.4 (3389.89-4722.1) | 5.66 (2.61-8.68) | 5.96 (2.75-9.14) | 129.51 (67.48-191.81) | 82.16 (42.81-121.68) |
| Dominica | 91.34 (71.57-114.51) | 278.31 (218.06-348.91) | 0.04 (0.03-0.07) | 0.14 (0.09-0.2) | 1.81 (1.17-2.66) | 5.53 (3.56-8.1) | 758.03 (640.81-879.93) | 4851.95 (4101.63-5632.2) | 1.6 (1.25-2.06) | 10.26 (7.99-13.22) | 26.35 (21.02-33.11) | 84.69 (67.55-106.39) |
| Dominican Republic | 12541.29 (9780.89-15663.59) | 235.73 (183.84-294.42) | 0.86 (0.53-1.33) | 0.02 (0.01-0.02) | 40.86 (26.68-60.15) | 0.77 (0.5-1.13) | 77786.42 (66107.78-90532.52) | 5026.86 (4272.14-5850.57) | 27.58 (18.42-36.76) | 1.78 (1.19-2.38) | 809.85 (544.4-1169.97) | 27.09 (18.21-39.14) |
| Ecuador | 16969.1 (13148.45-21345.35) | 203.47 (157.65-255.94) | 0.92 (0.57-1.4) | 0.01 (0.01-0.02) | 45.99 (29.59-67.43) | 0.55 (0.35-0.81) | 109992.3 (92963.71-128371.81) | 4294.69 (3629.8-5012.32) | 18.1 (13.62-23.73) | 0.71 (0.53-0.93) | 833.23 (505.64-1315.76) | 16.84 (10.22-26.59) |
| Egypt | 125268.8 (97689.7-155961.16) | 270.38 (210.86-336.63) | 3.68 (2.2-5.94) | 0.01 (0-0.01) | 216.44 (137.81-325.63) | 0.47 (0.3-0.7) | 508831.36 (429401.02-598687.8) | 4699.92 (3966.25-5529.89) | 70.79 (47.53-102.49) | 0.65 (0.44-0.95) | 3855.73 (2385.08-6062.52) | 20.81 (12.87-32.72) |
| El Salvador | 6588.25 (5150.97-8185.24) | 220.78 (172.62-274.3) | 0.12 (0.09-0.18) | 0 (0-0.01) | 8.52 (5.66-12.56) | 0.29 (0.19-0.42) | 51933.02 (43896.36-60511.69) | 5297.3 (4477.54-6172.35) | 6.74 (4.16-9.03) | 0.69 (0.42-0.92) | 359.71 (209.7-592.49) | 17.81 (10.38-29.34) |
| Equatorial Guinea | 976.48 (758.83-1219.89) | 163.22 (126.84-203.91) | 0.55 (0.21-1.13) | 0.09 (0.03-0.19) | 22.77 (8.82-45.74) | 3.81 (1.47-7.64) | 3979.97 (3390.99-4618.16) | 5311.85 (4525.77-6163.6) | 8.76 (3.77-14.72) | 11.69 (5.03-19.65) | 171.29 (85.89-280.93) | 128.96 (64.66-211.5) |
| Eritrea | 4761.62 (3698.13-5935.37) | 164.73 (127.94-205.33) | 2.06 (1.04-3.52) | 0.07 (0.04-0.12) | 86.33 (46.02-146.08) | 2.99 (1.59-5.05) | 15492.47 (13154.5-18120.8) | 3699.82 (3141.48-4327.5) | 16.6 (7.31-25.75) | 3.96 (1.75-6.15) | 410.36 (230.64-595) | 59.86 (33.64-86.79) |
| Estonia | 2898.7 (2251.42-3652.71) | 481.45 (373.94-606.69) | 0.09 (0.04-0.18) | 0.01 (0.01-0.03) | 4.71 (2.63-8.57) | 0.78 (0.44-1.42) | 37612.39 (32019.86-43792.72) | 8680.41 (7389.73-10106.74) | 6.96 (2.51-14.43) | 1.61 (0.58-3.33) | 270.05 (154.93-450.9) | 28.32 (16.25-47.29) |
| Eswatini | 1194.72 (930.11-1501.67) | 230.74 (179.63-290.02) | 0.65 (0.33-1.18) | 0.13 (0.06-0.23) | 26.98 (13.83-48.64) | 5.21 (2.67-9.39) | 5779.08 (4863.22-6752.71) | 6201.63 (5218.81-7246.46) | 4.12 (2.68-5.92) | 4.42 (2.87-6.35) | 118.19 (80-161.93) | 70.84 (47.95-97.07) |
| Ethiopia | 57211.6 (44301.76-71860.41) | 134.64 (104.26-169.11) | 21.62 (12.48-33.77) | 0.05 (0.03-0.08) | 903.54 (529.4-1391.89) | 2.13 (1.25-3.28) | 243213.05 (206869.76-283216.8) | 3754.08 (3193.11-4371.56) | 381.88 (205.4-553.71) | 5.89 (3.17-8.55) | 7796.12 (4680.54-10995.37) | 65.48 (39.31-92.36) |
| Fiji | 2422.28 (1893.71-3039.68) | 552.56 (431.99-693.4) | 0.09 (0.06-0.14) | 0.02 (0.01-0.03) | 5.39 (3.48-7.71) | 1.23 (0.79-1.76) | 9878.29 (8433.88-11497.99) | 7542.72 (6439.82-8779.47) | 0.91 (0.62-1.22) | 0.7 (0.47-0.94) | 74.62 (42.56-121.8) | 33.69 (19.22-54.99) |
| Finland | 17558.04 (13929.25-21805.15) | 733.4 (581.83-910.8) | 0.99 (0.35-2.15) | 0.04 (0.01-0.09) | 47.73 (20.16-95.05) | 1.99 (0.84-3.97) | 210131.23 (177763.47-243597.77) | 10671.67 (9027.85-12371.3) | 205.52 (83.27-369.97) | 10.44 (4.23-18.79) | 3171.13 (1720-5315.45) | 71.83 (38.96-120.41) |
| France | 220891.62 (173380.17-273072.01) | 769.5 (603.99-951.27) | 9.28 (3.77-18.75) | 0.03 (0.01-0.07) | 475 (235.57-863.02) | 1.65 (0.82-3.01) | 2291952.49 (1948785.09-2638422.29) | 10626.56 (9035.47-12232.95) | 1240.66 (542.19-2356.11) | 5.75 (2.51-10.92) | 23586.83 (14328.7-38097.01) | 48.76 (29.62-78.76) |
| Gabon | 1952.27 (1494.69-2447.05) | 239.29 (183.21-299.94) | 1.54 (0.75-2.62) | 0.19 (0.09-0.32) | 62.44 (30.53-104.03) | 7.65 (3.74-12.75) | 9009.99 (7687.44-10425.57) | 5176.93 (4417.02-5990.29) | 22.76 (13.04-32.13) | 13.08 (7.49-18.46) | 460.38 (292.85-631.66) | 151.49 (96.37-207.86) |
| Gambia | 1554.21 (1204.72-1966.13) | 170.47 (132.14-215.65) | 0.04 (0.02-0.06) | 0 (0-0.01) | 2.49 (1.6-3.74) | 0.27 (0.18-0.41) | 7224.24 (6134.16-8426) | 4809.96 (4084.17-5610.1) | 2.76 (1.41-4.18) | 1.84 (0.94-2.78) | 94.11 (56.8-142.31) | 33.29 (20.09-50.34) |
| Georgia | 7541.78 (5915.99-9496.63) | 447.33 (350.9-563.28) | 0.4 (0.16-0.92) | 0.02 (0.01-0.05) | 20.12 (9.7-41.33) | 1.19 (0.58-2.45) | 76891.74 (65647.42-89079.06) | 7274.49 (6210.7-8427.49) | 11.33 (4.6-23.73) | 1.07 (0.44-2.24) | 577.1 (323.58-972.9) | 26.78 (15.01-45.14) |
| Germany | 314860.31 (248032.02-389492.87) | 816.01 (642.82-1009.44) | 62.39 (26.7-124.57) | 0.16 (0.07-0.32) | 2597.63 (1185.33-5071.6) | 6.73 (3.07-13.14) | 3316995.05 (2839225.44-3831930.72) | 10831.77 (9271.6-12513.31) | 7687.19 (3763.56-14139.06) | 25.1 (12.29-46.17) | 103828.39 (56106.06-183042.17) | 153.35 (82.86-270.34) |
| Ghana | 26202.03 (20318.32-32793.95) | 182.62 (141.61-228.56) | 0.5 (0.36-0.68) | 0 (0-0) | 35.93 (23.51-53.11) | 0.25 (0.16-0.37) | 110098 (93811.02-128992.21) | 4245.03 (3617.05-4973.53) | 13.45 (9.84-20.93) | 0.52 (0.38-0.81) | 890.63 (530.45-1430.85) | 19.35 (11.53-31.09) |
| Greece | 45124.94 (35704.97-55590.87) | 955.23 (755.82-1176.78) | 0.86 (0.39-1.8) | 0.02 (0.01-0.04) | 55.97 (30.43-95.82) | 1.18 (0.64-2.03) | 447497.37 (377325.28-518761.66) | 12290.92 (10363.58-14248.25) | 117.7 (52.46-210.88) | 3.23 (1.44-5.79) | 3349.71 (1999.69-5429.88) | 39.81 (23.77-64.54) |
| Greenland | 241.88 (192.55-295) | 862.18 (686.34-1051.53) | 0.04 (0.02-0.08) | 0.14 (0.08-0.27) | 1.71 (1.08-3.07) | 6.09 (3.84-10.95) | 1226.2 (1051.51-1422.68) | 9515.28 (8159.71-11039.93) | 1.21 (0.92-1.58) | 9.37 (7.16-12.27) | 25.22 (19.91-33.37) | 114.27 (90.2-151.19) |
| Grenada | 154.72 (119.75-195.46) | 293.22 (226.93-370.41) | 0.12 (0.06-0.24) | 0.23 (0.11-0.45) | 4.86 (2.27-9.28) | 9.21 (4.31-17.59) | 918.83 (773.95-1070.31) | 4708.21 (3965.8-5484.43) | 2.55 (1.19-4.65) | 13.08 (6.11-23.83) | 44.29 (23.91-77.52) | 122.97 (66.38-215.24) |
| Guam | 395.34 (307.71-493) | 503.16 (391.63-627.46) | 0.02 (0.02-0.04) | 0.03 (0.02-0.05) | 1.18 (0.8-1.72) | 1.5 (1.01-2.2) | 2317.38 (1978.15-2667.96) | 6953.03 (5935.22-8004.92) | 0.45 (0.35-0.57) | 1.34 (1.04-1.7) | 20.06 (12.67-30.86) | 31.65 (19.99-48.68) |
| Guatemala | 14244.42 (11142.66-17725.13) | 172.83 (135.2-215.06) | 0.39 (0.23-0.69) | 0 (0-0.01) | 23.42 (14.97-37.74) | 0.28 (0.18-0.46) | 89263.82 (75308.96-104015.56) | 4937.82 (4165.88-5753.84) | 8.43 (3.7-16.17) | 0.47 (0.2-0.89) | 630.43 (346.86-1076.58) | 17.77 (9.78-30.35) |
| Guinea | 7352.98 (5713.55-9254.55) | 155.98 (121.2-196.32) | 0.21 (0.14-0.32) | 0 (0-0.01) | 13.37 (8.7-19.96) | 0.28 (0.18-0.42) | 35480.48 (30115.73-41394.12) | 3985.62 (3382.99-4649.92) | 14.13 (6.93-23.03) | 1.59 (0.78-2.59) | 490.06 (284.05-743.75) | 29.76 (17.25-45.17) |
| Guinea-Bissau | 1209.24 (938.42-1519) | 153.63 (119.22-192.98) | 0.04 (0.02-0.05) | 0 (0-0.01) | 2.27 (1.5-3.34) | 0.29 (0.19-0.42) | 4534.16 (3849.96-5316.59) | 4018.32 (3411.96-4711.74) | 1.67 (0.88-2.76) | 1.48 (0.78-2.45) | 62.73 (37.43-95.66) | 32.15 (19.19-49.03) |
| Guyana | 910.23 (706.08-1148.76) | 239.13 (185.5-301.8) | 0.33 (0.14-0.7) | 0.09 (0.04-0.18) | 13.76 (5.84-28.13) | 3.61 (1.53-7.39) | 4670.82 (3986.5-5488.55) | 4308.79 (3677.51-5063.14) | 4.62 (2.11-9.05) | 4.26 (1.95-8.35) | 102.23 (57.38-179.68) | 53.63 (30.1-94.26) |
| Haiti | 10228.24 (7990.86-12711.66) | 177.57 (138.72-220.68) | 4.15 (2.2-7.33) | 0.07 (0.04-0.13) | 171.88 (93.17-299.01) | 2.98 (1.62-5.19) | 44286.93 (37283.45-52060.34) | 3883.38 (3269.26-4565) | 53.61 (34.31-86.9) | 4.7 (3.01-7.62) | 1202.25 (818.79-1823.06) | 59.75 (40.69-90.6) |
| Honduras | 8736.5 (6701.7-11031.26) | 196.23 (150.53-247.77) | 0.63 (0.33-1.05) | 0.01 (0.01-0.02) | 29.53 (16.99-46.31) | 0.66 (0.38-1.04) | 49229.62 (41871.36-57588.67) | 4926.11 (4189.81-5762.55) | 19.31 (11.32-27.75) | 1.93 (1.13-2.78) | 555.25 (363.34-811.89) | 29.58 (19.36-43.26) |
| Hungary | 23739.87 (18700.88-29482.81) | 515.5 (406.08-640.21) | 24.62 (9.99-56.13) | 0.53 (0.22-1.22) | 987.95 (406.98-2241.13) | 21.45 (8.84-48.67) | 255431.21 (216302.48-297103.82) | 7984.69 (6761.54-9287.36) | 986.07 (374.35-2099.23) | 30.82 (11.7-65.62) | 15835.37 (7128.14-32461.77) | 229.62 (103.36-470.72) |
| Iceland | 1125.67 (887.26-1383.99) | 689.78 (543.69-848.08) | 0.03 (0.01-0.06) | 0.02 (0.01-0.04) | 1.77 (1.03-3.07) | 1.09 (0.63-1.88) | 9359.67 (8004.43-10779.15) | 10045.66 (8591.08-11569.17) | 4.06 (1.88-8.03) | 4.35 (2.02-8.61) | 82.78 (50.69-131.6) | 42.57 (26.07-67.68) |
| India | 1545687.98 (1209920.49-1933518.3) | 224.37 (175.63-280.67) | 92.29 (70.16-122.65) | 0.01 (0.01-0.02) | 4426.88 (3360.98-5955.89) | 0.64 (0.49-0.86) | 8230032.34 (7020807.15-9551536.77) | 4301.93 (3669.85-4992.7) | 2222.32 (1648.62-2853.03) | 1.16 (0.86-1.49) | 81733.92 (55719.12-119701.3) | 22.99 (15.68-33.67) |
| Indonesia | 686317.19 (538598.62-853982.65) | 509.52 (399.86-634) | 25.24 (15.25-37.96) | 0.02 (0.01-0.03) | 1361.2 (853.68-1979.83) | 1.01 (0.63-1.47) | 2638276.67 (2246413.75-3055996.98) | 7176.61 (6110.67-8312.89) | 337.75 (227.69-494.33) | 0.92 (0.62-1.34) | 21725.67 (12876-34575.13) | 34.27 (20.31-54.54) |
| Iran (Islamic Republic of) | 134070.18 (104567.08-167164.74) | 289.54 (225.82-361.01) | 7.63 (6.45-8.7) | 0.02 (0.01-0.02) | 364.75 (299.15-433.18) | 0.79 (0.65-0.94) | 654480.97 (560994.78-755726.05) | 5393.88 (4623.42-6228.29) | 163.79 (116.77-188.66) | 1.35 (0.96-1.55) | 5566.76 (3766.12-8287.92) | 24.9 (16.85-37.08) |
| Iraq | 54886.61 (43040.26-68906.5) | 271.03 (212.53-340.26) | 3.27 (1.97-5.03) | 0.02 (0.01-0.02) | 161.9 (101.29-235.59) | 0.8 (0.5-1.16) | 202675.66 (172098.12-235318.55) | 5510.66 (4679.27-6398.2) | 16.9 (12.61-28.51) | 0.46 (0.34-0.78) | 1324.93 (767.82-2198.1) | 20.29 (11.76-33.67) |
| Ireland | 19942.81 (15967.24-24392.86) | 857.69 (686.71-1049.08) | 2.41 (1.05-5.08) | 0.1 (0.05-0.22) | 104.29 (49.48-209.48) | 4.49 (2.13-9.01) | 138411.59 (117356.4-160159.65) | 11108.61 (9418.77-12854.06) | 243.58 (114.54-476.32) | 19.55 (9.19-38.23) | 3508.39 (1886.93-6453.02) | 135.03 (72.62-248.36) |
| Israel | 28680.41 (22780.72-35063.24) | 700.84 (556.68-856.81) | 3.43 (1.5-6.85) | 0.08 (0.04-0.17) | 148.34 (70.7-282.92) | 3.62 (1.73-6.91) | 208560.97 (177721.91-241857.92) | 10956.91 (9336.76-12706.19) | 329.44 (160.93-595.15) | 17.31 (8.45-31.27) | 4787.51 (2702.03-8201.98) | 118.35 (66.8-202.76) |
| Italy | 255502.8 (203146.6-313017.18) | 931.89 (740.94-1141.67) | 24.94 (10.6-53.38) | 0.09 (0.04-0.19) | 1085.89 (531.33-2214.38) | 3.96 (1.94-8.08) | 2554803.39 (2169222.53-2936062.85) | 11581.93 (9833.94-13310.33) | 3589.67 (1682.92-6866.78) | 16.27 (7.63-31.13) | 51133.14 (28812.83-89871.06) | 101.33 (57.1-178.09) |
| Jamaica | 3758.63 (2928.16-4686.73) | 261.04 (203.36-325.5) | 2.47 (0.93-5.94) | 0.17 (0.06-0.41) | 99.69 (39.29-235.02) | 6.92 (2.73-16.32) | 28500.24 (24406.1-32948.31) | 5619.44 (4812.19-6496.47) | 71.34 (30.43-132.43) | 14.07 (6-26.11) | 1078.37 (546.19-2021.8) | 106.53 (53.96-199.74) |
| Japan | 311501.49 (245926.02-383377.14) | 572.14 (451.7-704.16) | 7.55 (3.92-15.01) | 0.01 (0.01-0.03) | 423.55 (243.69-749.71) | 0.78 (0.45-1.38) | 4553999.89 (3878286.31-5272548.8) | 8800.58 (7494.77-10189.17) | 1036.39 (424.64-1839.42) | 2 (0.82-3.55) | 31034.94 (17998.48-50869.07) | 24.4 (14.15-40) |
| Jordan | 16558.65 (12907.27-20823.22) | 292.67 (228.13-368.05) | 0.28 (0.21-0.38) | 0 (0-0.01) | 19.5 (13.37-28.37) | 0.34 (0.24-0.5) | 55389.07 (46909.01-64816.82) | 5188.19 (4393.88-6071.27) | 3.92 (3.08-5.02) | 0.37 (0.29-0.47) | 328.44 (181.23-553.26) | 17.72 (9.78-29.85) |
| Kazakhstan | 31329.7 (24299.18-39175.05) | 344.76 (267.4-431.09) | 1.65 (0.73-3.49) | 0.02 (0.01-0.04) | 82.37 (41.74-157.49) | 0.91 (0.46-1.73) | 169011.8 (143749.23-196761.76) | 5555.56 (4725.16-6467.73) | 20.79 (9.93-39.09) | 0.68 (0.33-1.28) | 1194.49 (682.64-2001.14) | 22.24 (12.71-37.26) |
| Kenya | 37829.55 (29453.44-47329.65) | 174.6 (135.94-218.44) | 12.62 (8.3-17.6) | 0.06 (0.04-0.08) | 529.59 (357.91-723.83) | 2.44 (1.65-3.34) | 153544.83 (130497.93-178503.19) | 4340.37 (3688.88-5045.89) | 178.17 (90.05-243.52) | 5.04 (2.55-6.88) | 3947.71 (2386.86-5252.84) | 64.27 (38.86-85.52) |
| Kiribati | 277.37 (213.74-348.85) | 513.39 (395.6-645.69) | 0.01 (0.01-0.02) | 0.02 (0.01-0.04) | 0.67 (0.43-1.02) | 1.25 (0.79-1.88) | 941.56 (789.88-1099.16) | 8097.6 (6793.15-9453.02) | 0.07 (0.05-0.14) | 0.6 (0.4-1.22) | 7.45 (4.06-12.26) | 40.42 (22.05-66.57) |
| Kuwait | 8427.92 (6649-10530.85) | 287.31 (226.67-359) | 0.14 (0.1-0.23) | 0 (0-0.01) | 9.28 (6.06-14.21) | 0.32 (0.21-0.48) | 19016.59 (16220.69-21841.29) | 4792.11 (4087.55-5503.92) | 1.95 (0.86-3.87) | 0.49 (0.22-0.97) | 110.31 (60.24-181.78) | 16.49 (9.01-27.18) |
| Kyrgyzstan | 8505.56 (6700.6-10690.52) | 271.54 (213.92-341.3) | 1.52 (0.58-3.16) | 0.05 (0.02-0.1) | 65.22 (27.29-132.37) | 2.08 (0.87-4.23) | 37399.74 (31991.41-43338.91) | 4737.55 (4052.46-5489.89) | 17.46 (8.11-34.59) | 2.21 (1.03-4.38) | 479.21 (276.18-795.75) | 35.39 (20.4-58.77) |
| Lao People's Democratic Republic | 12972.54 (10064.31-16334.53) | 372.91 (289.31-469.56) | 0.46 (0.25-0.76) | 0.01 (0.01-0.02) | 26.55 (16.29-40.07) | 0.76 (0.47-1.15) | 46359.38 (39227.05-53821.28) | 6415.2 (5428.23-7447.78) | 5.54 (3.91-7.47) | 0.77 (0.54-1.03) | 398.7 (233.74-640.21) | 31.72 (18.6-50.94) |
| Latvia | 4324.36 (3359.76-5389.31) | 502.8 (390.65-626.63) | 0.35 (0.12-1.01) | 0.04 (0.01-0.12) | 15.79 (6.58-40.7) | 1.84 (0.77-4.73) | 55693.31 (47212.29-64577.65) | 8398.1 (7119.23-9737.78) | 29.25 (8.01-93.1) | 4.41 (1.21-14.04) | 663.45 (321.06-1583.73) | 45.88 (22.2-109.52) |
| Lebanon | 9133.04 (7126.5-11472.52) | 353.55 (275.87-444.11) | 0.28 (0.17-0.43) | 0.01 (0.01-0.02) | 15.41 (9.89-22.48) | 0.6 (0.38-0.87) | 61522.44 (52160.84-71863.61) | 7102.95 (6022.13-8296.87) | 12.26 (8.05-16.15) | 1.42 (0.93-1.86) | 456.38 (293.2-700.47) | 25.9 (16.64-39.75) |
| Lesotho | 2377.28 (1867.67-2964.43) | 237.69 (186.74-296.4) | 1 (0.5-1.71) | 0.1 (0.05-0.17) | 41.5 (21.38-71.18) | 4.15 (2.14-7.12) | 12421.93 (10511.54-14469.78) | 5892.23 (4986.05-6863.61) | 8.15 (4.99-11.78) | 3.87 (2.36-5.59) | 240.29 (161.51-330.99) | 64.38 (43.27-88.67) |
| Liberia | 4049.36 (3109.61-5120.62) | 194.77 (149.57-246.3) | 0.09 (0.06-0.15) | 0 (0-0.01) | 5.97 (3.78-8.86) | 0.29 (0.18-0.43) | 13769.71 (11711.29-16056.27) | 4401.15 (3743.22-5131.99) | 4.63 (2.43-7.52) | 1.48 (0.78-2.4) | 166.8 (99.37-252.49) | 29.82 (17.76-45.14) |
| Libya | 11882.35 (9249.49-14787) | 305.6 (237.89-380.31) | 0.3 (0.18-0.45) | 0.01 (0-0.01) | 17.73 (11.8-26.24) | 0.46 (0.3-0.67) | 41414.49 (35438.29-48033.78) | 5235.2 (4479.75-6071.94) | 6.44 (4.03-9.05) | 0.81 (0.51-1.14) | 302.94 (180.44-476.18) | 20.77 (12.37-32.64) |
| Lithuania | 6349.71 (4962.2-7962.08) | 497.56 (388.84-623.9) | 1.46 (0.55-3.12) | 0.11 (0.04-0.24) | 59.9 (24.81-124.63) | 4.69 (1.94-9.77) | 77457.89 (65530.75-90361.74) | 8034.85 (6797.62-9373.39) | 86.5 (37.02-158.3) | 8.97 (3.84-16.42) | 1595.14 (839.13-2755.31) | 76.62 (40.31-132.35) |
| Luxembourg | 2783.94 (2215.09-3413.19) | 868.7 (691.19-1065.04) | 0.14 (0.05-0.3) | 0.04 (0.02-0.1) | 6.67 (3.2-13.56) | 2.08 (1-4.23) | 18883.15 (16234.63-21770.88) | 11392.11 (9794.27-13134.26) | 12.64 (5.84-25.22) | 7.62 (3.52-15.22) | 227.46 (130.29-392.84) | 66.12 (37.87-114.19) |
| Madagascar | 19704.56 (15165.01-24757.08) | 178.06 (137.04-223.72) | 12.68 (5.95-21.12) | 0.11 (0.05-0.19) | 525.77 (256.9-867) | 4.75 (2.32-7.83) | 65609.14 (55535.61-77310.79) | 3743.42 (3168.66-4411.07) | 100.41 (48.39-150.49) | 5.73 (2.76-8.59) | 2336.66 (1288.99-3427) | 81.32 (44.86-119.26) |
| Malawi | 12205.8 (9547.94-15297.7) | 171.3 (134-214.69) | 3.84 (1.99-6.47) | 0.05 (0.03-0.09) | 162.97 (88.17-271.47) | 2.29 (1.24-3.81) | 54151.1 (45909.95-63436.81) | 4739.18 (4017.93-5551.84) | 56.25 (26.89-82.87) | 4.92 (2.35-7.25) | 1304.92 (751.76-1811.48) | 62.62 (36.08-86.93) |
| Malaysia | 62709.67 (48694.45-78266.65) | 385.88 (299.64-481.61) | 1.99 (1.29-2.95) | 0.01 (0.01-0.02) | 113.14 (74.83-160.08) | 0.7 (0.46-0.99) | 302248.35 (257526.39-349453.98) | 6529.34 (5563.23-7549.11) | 32.89 (24.52-42.73) | 0.71 (0.53-0.92) | 2189.59 (1290.41-3546.64) | 26.06 (15.36-42.22) |
| Maldives | 973.47 (755.64-1205.66) | 314.16 (243.86-389.1) | 0.03 (0.02-0.05) | 0.01 (0.01-0.02) | 1.75 (1.07-2.65) | 0.56 (0.35-0.86) | 3086.38 (2631.29-3545.09) | 6380.3 (5439.52-7328.59) | 0.91 (0.64-1.54) | 1.89 (1.33-3.19) | 28.52 (19.03-43.26) | 33.31 (22.23-50.53) |
| Mali | 12651.42 (9864.81-15998.65) | 163.32 (127.35-206.54) | 0.31 (0.2-0.47) | 0 (0-0.01) | 20.7 (13.45-30.85) | 0.27 (0.17-0.4) | 55191.94 (46999.51-64053.66) | 3954.87 (3367.83-4589.87) | 20 (10.32-32.57) | 1.43 (0.74-2.33) | 722.76 (445.32-1077.87) | 29 (17.87-43.25) |
| Malta | 1716.48 (1360.01-2138.43) | 850 (673.48-1058.95) | 0.19 (0.08-0.42) | 0.1 (0.04-0.21) | 8.5 (3.99-17.1) | 4.21 (1.98-8.47) | 18958.43 (15953.3-21973.38) | 12328.59 (10374.36-14289.2) | 22.3 (10.25-42.26) | 14.5 (6.67-27.48) | 350.98 (199.34-599.85) | 104.56 (59.38-178.69) |
| Marshall Islands | 112.38 (88.44-140.26) | 413.2 (325.18-515.69) | 0.01 (0.01-0.02) | 0.04 (0.02-0.06) | 0.47 (0.27-0.77) | 1.74 (1-2.83) | 340.17 (287.64-398.1) | 5959.17 (5038.85-6973.92) | 0.06 (0.04-0.09) | 1.04 (0.71-1.5) | 3.36 (2.1-5.13) | 36.59 (22.86-55.91) |
| Mauritania | 2936.37 (2295.58-3690.32) | 182.51 (142.68-229.37) | 0.07 (0.04-0.12) | 0 (0-0.01) | 4.59 (2.81-7.24) | 0.29 (0.17-0.45) | 14874.64 (12680.09-17300.56) | 4335.04 (3695.46-5042.05) | 6.45 (2.98-10.12) | 1.88 (0.87-2.95) | 205.33 (113.51-305.05) | 32.32 (17.87-48.01) |
| Mauritius | 4036.74 (3159.67-5026.92) | 621.47 (486.44-773.91) | 0.11 (0.05-0.25) | 0.02 (0.01-0.04) | 7.06 (3.82-12.73) | 1.09 (0.59-1.96) | 25001.16 (21279.7-29173) | 7838.16 (6671.44-9146.08) | 4.69 (2.16-9.22) | 1.47 (0.68-2.89) | 205.62 (119.78-344.71) | 34.95 (20.36-58.6) |
| Mexico | 162430.9 (126578.13-202603.34) | 261.96 (204.13-326.74) | 26.53 (12.25-56.89) | 0.04 (0.02-0.09) | 1122.62 (539.92-2299.16) | 1.81 (0.87-3.71) | 1068663.63 (910030.18-1239975.81) | 5420.51 (4615.88-6289.45) | 731.99 (334.45-1497.49) | 3.71 (1.7-7.6) | 15287.34 (8972.46-25746.64) | 40.45 (23.74-68.12) |
| Micronesia (Federated States of) | 211.42 (165.08-263.05) | 447.82 (349.66-557.17) | 0.02 (0.01-0.03) | 0.04 (0.02-0.07) | 0.85 (0.45-1.46) | 1.8 (0.95-3.09) | 740.21 (621.68-867.37) | 5990.92 (5031.63-7020.08) | 0.15 (0.1-0.22) | 1.23 (0.85-1.78) | 7.44 (4.73-11.13) | 38.24 (24.31-57.2) |
| Monaco | 157.7 (125.12-195.29) | 1008.87 (800.43-1249.33) | 0.01 (0-0.01) | 0.05 (0.03-0.07) | 0.38 (0.24-0.55) | 2.42 (1.56-3.54) | 1763.75 (1504.04-2028.26) | 11643.78 (9929.24-13389.98) | 0.92 (0.69-1.22) | 6.07 (4.59-8.03) | 18.06 (13.08-25.66) | 53.03 (38.42-75.36) |
| Mongolia | 5664.53 (4407.98-7101.79) | 319.85 (248.9-401) | 0.09 (0.06-0.14) | 0.01 (0-0.01) | 6.89 (4.45-10.56) | 0.39 (0.25-0.6) | 18333.12 (15756.45-21398.87) | 4756.93 (4088.35-5552.4) | 1.1 (0.83-1.67) | 0.29 (0.21-0.43) | 113.76 (61.17-195.17) | 18.1 (9.73-31.06) |
| Montenegro | 1533.67 (1196.09-1926.49) | 517.65 (403.71-650.24) | 0.08 (0.06-0.12) | 0.03 (0.02-0.04) | 4.1 (2.9-5.7) | 1.38 (0.98-1.92) | 13556.1 (11555.47-15730.36) | 7772.66 (6625.56-9019.31) | 2.58 (2.01-3.34) | 1.48 (1.15-1.92) | 104.96 (71.07-159.27) | 30.52 (20.67-46.32) |
| Morocco | 54556.4 (42682.07-68113.24) | 303.76 (237.65-379.25) | 1.62 (0.94-2.52) | 0.01 (0.01-0.01) | 94.82 (60.61-143.72) | 0.53 (0.34-0.8) | 261504.34 (222509.16-304631.02) | 4799.45 (4083.76-5590.96) | 50.43 (33.87-67.94) | 0.93 (0.62-1.25) | 2137.8 (1354.66-3279.27) | 22.34 (14.15-34.26) |
| Mozambique | 20117 (15488.58-25276.15) | 186.62 (143.68-234.47) | 9.51 (4.32-15.71) | 0.09 (0.04-0.15) | 395.04 (189.88-642.7) | 3.66 (1.76-5.96) | 81064.89 (68808.9-95100.11) | 4672.18 (3965.8-5481.1) | 118.33 (59.41-172.3) | 6.82 (3.42-9.93) | 2665.3 (1506.32-3706.65) | 87.14 (49.25-121.18) |
| Myanmar | 140921.12 (109391.13-176021.63) | 525.39 (407.84-656.25) | 4.87 (2.82-7.46) | 0.02 (0.01-0.03) | 281.27 (177.09-415.4) | 1.05 (0.66-1.55) | 599572.79 (509204.23-699277.63) | 7575.55 (6433.75-8835.31) | 71.01 (53.55-95.56) | 0.9 (0.68-1.21) | 4970.32 (2933.03-8072.05) | 35.11 (20.72-57.02) |
| Namibia | 2598.71 (2032.66-3245.89) | 236.54 (185.01-295.44) | 1.71 (0.92-2.85) | 0.16 (0.08-0.26) | 70.16 (38.35-118.07) | 6.39 (3.49-10.75) | 13272.75 (11346.16-15369.18) | 5857.19 (5007-6782.34) | 13.78 (8.73-19.61) | 6.08 (3.85-8.65) | 329.1 (223.75-456.78) | 77.62 (52.77-107.73) |
| Nauru | 18.52 (14.43-23.04) | 381.09 (296.9-474.09) | 0 (0-0) | 0.04 (0.02-0.07) | 0.09 (0.05-0.15) | 1.8 (1.07-3.11) | 36.8 (30.78-43.13) | 5707.12 (4773.71-6688.83) | 0.01 (0-0.01) | 0.99 (0.67-1.45) | 0.34 (0.21-0.52) | 37.69 (23.7-57.42) |
| Nepal | 30719.24 (24091.16-38171.52) | 217.5 (170.57-270.27) | 1.49 (0.84-2.42) | 0.01 (0.01-0.02) | 77.1 (48.41-121.83) | 0.55 (0.34-0.86) | 156403.1 (132488.28-182994.41) | 4144.68 (3510.94-4849.35) | 47.25 (29.02-66.09) | 1.25 (0.77-1.75) | 1663.55 (1056.38-2467.15) | 23.92 (15.19-35.47) |
| Netherlands | 64775.55 (51101.22-79494.62) | 836.8 (660.15-1026.95) | 9.92 (4.6-19.54) | 0.13 (0.06-0.25) | 420 (206.94-802.66) | 5.43 (2.67-10.37) | 589702.81 (504810.18-688377.67) | 10444.5 (8940.93-12192.18) | 1195.51 (573.75-2180.39) | 21.17 (10.16-38.62) | 16588.49 (9247.01-28931.03) | 137 (76.37-238.93) |
| New Zealand | 9627.12 (7551.11-11941.55) | 483.16 (378.97-599.32) | 0.97 (0.48-2) | 0.05 (0.02-0.1) | 41.88 (21.83-80.91) | 2.1 (1.1-4.06) | 87431.41 (74473.71-101011.95) | 6628.99 (5646.55-7658.66) | 195.04 (93.16-370.94) | 14.79 (7.06-28.12) | 2473.6 (1357.24-4460.66) | 89.33 (49.02-161.1) |
| Nicaragua | 6542.82 (5102.27-8208.26) | 208.18 (162.35-261.18) | 0.17 (0.12-0.25) | 0.01 (0-0.01) | 10.31 (7.07-15.19) | 0.33 (0.22-0.48) | 35652.3 (30235.84-41432.57) | 4826.38 (4093.13-5608.87) | 5.48 (4.2-6.77) | 0.74 (0.57-0.92) | 261.88 (161.66-413.57) | 19.28 (11.9-30.45) |
| Niger | 11417.65 (8832.1-14294.22) | 153.42 (118.68-192.08) | 0.29 (0.18-0.45) | 0 (0-0.01) | 19.24 (11.99-28.22) | 0.26 (0.16-0.38) | 47369.51 (40247.75-54959.64) | 3656.19 (3106.5-4242.03) | 14.77 (6.77-26.78) | 1.14 (0.52-2.07) | 598.51 (352.04-940.23) | 27.16 (15.97-42.67) |
| Nigeria | 140883.43 (108879.1-177106.34) | 169.63 (131.09-213.24) | 3.41 (2.37-5.03) | 0 (0-0.01) | 213.35 (147.7-306.81) | 0.26 (0.18-0.37) | 545994.72 (465213.67-632868.98) | 3910.5 (3331.94-4532.71) | 212.91 (106.4-315.74) | 1.52 (0.76-2.26) | 7107.58 (4227.62-10647.78) | 29.22 (17.38-43.78) |
| Niue | 4.66 (3.64-5.89) | 624.84 (487.58-790) | 0 (0-0) | 0.05 (0.03-0.08) | 0.02 (0.01-0.03) | 2.35 (1.39-3.68) | 29.91 (25.57-34.52) | 7736.29 (6612.96-8926.51) | 0.01 (0.01-0.01) | 1.86 (1.41-2.43) | 0.29 (0.2-0.43) | 38.7 (26.34-57.38) |
| North Macedonia | 5032.37 (3932.44-6270.73) | 452.04 (353.24-563.28) | 0.17 (0.11-0.26) | 0.01 (0.01-0.02) | 9.26 (6.14-13.59) | 0.83 (0.55-1.22) | 37464.12 (31909.52-43859.66) | 6550.19 (5579.03-7668.38) | 7.03 (5.19-9.12) | 1.23 (0.91-1.59) | 296.64 (193.12-449.67) | 27.25 (17.74-41.3) |
| Northern Mariana Islands | 153.26 (119.78-192.18) | 737.51 (576.4-924.78) | 0.01 (0.01-0.02) | 0.06 (0.03-0.09) | 0.56 (0.35-0.86) | 2.69 (1.68-4.12) | 553.48 (467.55-646.84) | 5502.96 (4648.56-6431.17) | 0.15 (0.11-0.2) | 1.54 (1.1-1.95) | 5.46 (3.71-8.02) | 35.2 (23.92-51.75) |
| Norway | 20099.76 (15975-24715.38) | 792.07 (629.52-973.95) | 1.27 (0.56-2.81) | 0.05 (0.02-0.11) | 58.24 (28.94-119.41) | 2.3 (1.14-4.71) | 176330.64 (149710.72-203612.56) | 11334.4 (9623.29-13088.06) | 156.5 (73.09-294.3) | 10.06 (4.7-18.92) | 2575.1 (1480.85-4313.3) | 76.51 (44-128.16) |
| Oman | 5525.17 (4323.41-6898.8) | 182.59 (142.87-227.98) | 0.3 (0.16-0.51) | 0.01 (0.01-0.02) | 14.38 (8.59-22.78) | 0.48 (0.28-0.75) | 12445.11 (10521.43-14482.07) | 4521.69 (3822.76-5261.78) | 3.1 (1.69-4.51) | 1.13 (0.61-1.64) | 109.11 (70.8-160.55) | 25.14 (16.31-36.99) |
| Pakistan | 209980.21 (164317.14-262164.85) | 222.4 (174.04-277.67) | 14.68 (8.9-23.11) | 0.02 (0.01-0.02) | 692.74 (458.5-1053.63) | 0.73 (0.49-1.12) | 804422.42 (684530.83-937218.76) | 4524.17 (3849.89-5271.03) | 246.72 (166.63-358.55) | 1.39 (0.94-2.02) | 8824.12 (5909.44-13279.37) | 28.41 (19.03-42.76) |
| Palau | 65.37 (51.12-82.45) | 679.82 (531.58-857.39) | 0 (0-0) | 0.02 (0.01-0.05) | 0.13 (0.06-0.25) | 1.4 (0.58-2.63) | 248.97 (211.19-289.65) | 6470 (5488-7527.1) | 0.01 (0.01-0.01) | 0.24 (0.17-0.37) | 1.47 (0.75-2.56) | 23.31 (11.94-40.6) |
| Palestine | 5124.8 (4028.91-6395.92) | 233.79 (183.8-291.78) | 0.07 (0.06-0.09) | 0 (0-0) | 5.64 (3.92-8.35) | 0.26 (0.18-0.38) | 18225.08 (15502.83-21202.06) | 4676.57 (3978.04-5440.47) | 1.33 (1-1.64) | 0.34 (0.26-0.42) | 111.35 (62.05-184.65) | 16.56 (9.23-27.46) |
| Panama | 4732.49 (3712.66-5921.94) | 240.19 (188.43-300.56) | 1.25 (0.51-2.79) | 0.06 (0.03-0.14) | 52.12 (22.66-114) | 2.65 (1.15-5.79) | 35439.3 (30056.15-41037.25) | 5090.18 (4317-5894.22) | 45.7 (20.41-90.38) | 6.56 (2.93-12.98) | 729.62 (417.56-1301.88) | 52.48 (30.03-93.64) |
| Papua New Guinea | 15640.23 (12252.65-19522.3) | 348.66 (273.14-435.2) | 0.62 (0.32-1.15) | 0.01 (0.01-0.03) | 35.52 (20.93-61.85) | 0.79 (0.47-1.38) | 41849.4 (35212.29-48883.54) | 5616.07 (4725.39-6560.03) | 4.35 (2.94-7.49) | 0.58 (0.39-1.01) | 354.75 (202.91-567.68) | 28.81 (16.48-46.1) |
| Paraguay | 7743.77 (6049.7-9697.4) | 229.51 (179.3-287.41) | 1.04 (0.67-1.53) | 0.03 (0.02-0.05) | 46.17 (30.86-66.26) | 1.37 (0.91-1.96) | 49263.39 (41774.37-57322.24) | 5236.25 (4440.23-6092.83) | 22.88 (14.86-30.44) | 2.43 (1.58-3.24) | 587.17 (406.24-832.03) | 32.94 (22.79-46.67) |
| Peru | 33471.98 (26123.26-41706.66) | 200.79 (156.7-250.18) | 1.1 (0.68-1.68) | 0.01 (0-0.01) | 61.87 (40.24-92.87) | 0.37 (0.24-0.56) | 222139.01 (188571.07-257452.62) | 4170.23 (3540.06-4833.17) | 38.17 (26.11-52.63) | 0.72 (0.49-0.99) | 1641.66 (1001.24-2646.37) | 15.5 (9.45-24.98) |
| Philippines | 211454.07 (165434.81-263430.66) | 401.16 (313.85-499.76) | 11.79 (8.51-15.52) | 0.02 (0.02-0.03) | 586.32 (428.78-764.29) | 1.11 (0.81-1.45) | 891626.35 (760962.12-1032715.07) | 6794.15 (5798.5-7869.24) | 94.87 (76.91-117.84) | 0.72 (0.59-0.9) | 7145.26 (4285.66-11385.98) | 30.88 (18.52-49.2) |
| Poland | 73935.33 (57664.74-92350.26) | 396.12 (308.95-494.78) | 19.52 (6.83-44.46) | 0.1 (0.04-0.24) | 801.11 (295.91-1776.45) | 4.29 (1.59-9.52) | 864124.17 (738782.12-996725.68) | 7164.34 (6125.15-8263.73) | 1122.09 (397.85-2611.67) | 9.3 (3.3-21.65) | 19677.39 (9525.42-39981.06) | 78.82 (38.16-160.15) |
| Portugal | 43496.39 (34416-53379.41) | 889.08 (703.48-1091.1) | 5.8 (2.58-12.13) | 0.12 (0.05-0.25) | 249.64 (121.66-493.25) | 5.1 (2.49-10.08) | 424265.73 (359409.73-490717.59) | 11197.99 (9486.19-12951.91) | 770.49 (358.52-1428.98) | 20.34 (9.46-37.72) | 10540.88 (5701.19-18652.3) | 123.51 (66.8-218.55) |
| Puerto Rico | 4929.75 (3861.05-6188.05) | 309.91 (242.73-389.01) | 2.62 (1.1-5.24) | 0.16 (0.07-0.33) | 105.91 (45.93-209.71) | 6.66 (2.89-13.18) | 67027.7 (56680.4-77663.1) | 5699.05 (4819.27-6603.32) | 157 (70.38-306.68) | 13.35 (5.98-26.08) | 2217.83 (1208.15-4143.82) | 85.89 (46.79-160.48) |
| Qatar | 4057.14 (3146.02-5065.94) | 186.62 (144.71-233.02) | 0.09 (0.06-0.15) | 0 (0-0.01) | 5.51 (3.47-8.47) | 0.25 (0.16-0.39) | 5805.78 (4847.83-6847.97) | 3359.45 (2805.14-3962.5) | 1.24 (0.71-1.74) | 0.72 (0.41-1.01) | 41.65 (25.58-62.34) | 18.32 (11.25-27.42) |
| Republic of Korea | 140341.26 (109682.82-174028.44) | 504.22 (394.07-625.25) | 3.15 (2.29-4.28) | 0.01 (0.01-0.02) | 196.24 (135.93-286.11) | 0.71 (0.49-1.03) | 942212.32 (809982.3-1088932.22) | 5876.8 (5052.05-6791.93) | 201.96 (160.7-245.48) | 1.26 (1-1.53) | 6413.4 (4329.2-9840.41) | 20.83 (14.06-31.95) |
| Republic of Moldova | 8080.18 (6280.18-10109.59) | 430.98 (334.97-539.23) | 0.84 (0.27-3.09) | 0.04 (0.01-0.17) | 37.3 (14.13-124.88) | 1.99 (0.75-6.66) | 72426.31 (62083.54-84632.68) | 6960.75 (5966.72-8133.88) | 32.98 (13.4-72.33) | 3.17 (1.29-6.95) | 878.76 (464.02-1662.72) | 43.65 (23.05-82.58) |
| Romania | 42037.37 (32833.66-52460.85) | 465.1 (363.27-580.42) | 23.09 (8.22-50.23) | 0.26 (0.09-0.56) | 932.66 (349.21-1994.36) | 10.32 (3.86-22.07) | 417422.32 (353697.53-486240.39) | 6797.21 (5759.53-7917.83) | 752.48 (338.76-1472.86) | 12.25 (5.52-23.98) | 13369.63 (6708.97-24975.85) | 101.1 (50.73-188.86) |
| Russian Federation | 347517.21 (271287.31-432013.94) | 497.35 (388.25-618.27) | 224.46 (90.32-502.58) | 0.32 (0.13-0.72) | 8950.52 (3658.97-19912.6) | 12.81 (5.24-28.5) | 3744652.03 (3210575.64-4310668.18) | 8819.01 (7561.21-10152.03) | 8827.83 (4298.06-17225.13) | 20.79 (10.12-40.57) | 156664.77 (81260.05-294296.15) | 188.22 (97.63-353.57) |
| Rwanda | 9817.7 (7645.15-12329.59) | 180.68 (140.7-226.91) | 3.25 (1.52-5.58) | 0.06 (0.03-0.1) | 138.32 (67.45-230.58) | 2.55 (1.24-4.24) | 43925.62 (37169.83-51410.05) | 4374.5 (3701.7-5119.87) | 43.28 (17.7-70.34) | 4.31 (1.76-7.01) | 1017.34 (532.98-1507.24) | 59.16 (30.99-87.65) |
| Saint Kitts and Nevis | 91.53 (71.39-116.01) | 293.71 (229.08-372.27) | 0.1 (0.04-0.21) | 0.32 (0.12-0.68) | 3.96 (1.54-8.47) | 12.7 (4.95-27.19) | 499.25 (421.47-582.15) | 4128.44 (3485.26-4814.02) | 2.25 (1.07-4.32) | 18.64 (8.84-35.73) | 37.19 (19.08-68.46) | 182.13 (93.41-335.26) |
| Saint Lucia | 281.92 (220.61-356.36) | 304.4 (238.2-384.78) | 0.27 (0.12-0.55) | 0.3 (0.13-0.59) | 10.93 (5.08-21.76) | 11.8 (5.48-23.49) | 1844.59 (1562.93-2139.97) | 5021.21 (4254.51-5825.3) | 7.3 (3.52-13.89) | 19.88 (9.57-37.8) | 108.55 (58.02-194.77) | 152.34 (81.42-273.34) |
| Saint Vincent and the Grenadines | 164.05 (128.52-203.26) | 297.27 (232.87-368.3) | 0.08 (0.03-0.16) | 0.14 (0.06-0.28) | 3.23 (1.43-6.19) | 5.85 (2.6-11.22) | 1167.51 (990.54-1357.83) | 4907.32 (4163.48-5707.3) | 1.98 (0.95-3.75) | 8.33 (4-15.76) | 35.32 (19.8-61.47) | 78.07 (43.77-135.87) |
| Samoa | 472.11 (366.19-592.01) | 528.38 (409.84-662.57) | 0.03 (0.02-0.05) | 0.03 (0.02-0.05) | 1.41 (0.88-2.18) | 1.58 (0.98-2.44) | 2006.76 (1713.56-2330.32) | 8167.02 (6973.75-9483.85) | 0.33 (0.25-0.45) | 1.35 (1-1.83) | 17.49 (10.89-27.26) | 39.43 (24.55-61.46) |
| San Marino | 140.96 (110.32-174.04) | 903.94 (707.47-1116.1) | 0 (0-0.01) | 0.03 (0.01-0.05) | 0.24 (0.13-0.39) | 1.51 (0.82-2.5) | 1149.91 (978.3-1326.23) | 11378.7 (9680.56-13123.41) | 0.87 (0.53-1.35) | 8.64 (5.22-13.33) | 13.58 (9.11-19.35) | 60.54 (40.65-86.27) |
| Sao Tome and Principe | 179.58 (139.06-225.56) | 197.15 (152.66-247.63) | 0.01 (0-0.01) | 0.01 (0-0.01) | 0.33 (0.22-0.49) | 0.36 (0.24-0.54) | 735.05 (628.11-859.09) | 4324.91 (3695.72-5054.74) | 0.33 (0.18-0.48) | 1.97 (1.04-2.83) | 10.26 (6.21-15.18) | 34.05 (20.63-50.4) |
| Saudi Arabia | 51168.44 (40162.83-63884.37) | 219.21 (172.06-273.69) | 0.9 (0.63-1.3) | 0 (0-0.01) | 58.37 (40.08-84.28) | 0.25 (0.17-0.36) | 102209.59 (86879.39-118884.4) | 3759.84 (3195.91-4373.24) | 7.08 (5.47-9.15) | 0.26 (0.2-0.34) | 572.18 (319.57-950.75) | 13.49 (7.54-22.42) |
| Senegal | 11144.21 (8630.57-13929.44) | 182.22 (141.12-227.76) | 0.3 (0.18-0.47) | 0 (0-0.01) | 19.28 (12.27-28.63) | 0.32 (0.2-0.47) | 54013.26 (45964.4-62806.53) | 4356.36 (3707.19-5065.56) | 21.41 (10.63-31.87) | 1.73 (0.86-2.57) | 727.26 (429.64-1072.47) | 32.4 (19.14-47.78) |
| Serbia | 18667.9 (14605.78-23278.27) | 464.51 (363.44-579.23) | 3.59 (2.3-5.35) | 0.09 (0.06-0.13) | 151.65 (101.36-219.71) | 3.77 (2.52-5.47) | 201127.67 (170693.53-234566.42) | 7348.46 (6236.51-8570.19) | 134.76 (91.01-174.95) | 4.92 (3.33-6.39) | 2975.14 (2154.1-3918.7) | 52.17 (37.77-68.71) |
| Seychelles | 289.23 (225.68-361.32) | 533.65 (416.4-666.67) | 0.02 (0.01-0.03) | 0.04 (0.02-0.06) | 0.98 (0.57-1.55) | 1.8 (1.05-2.86) | 1324.65 (1135.32-1531.84) | 6907.68 (5920.38-7988.09) | 0.28 (0.17-0.41) | 1.46 (0.91-2.14) | 12.07 (7.76-18.77) | 35.13 (22.57-54.61) |
| Sierra Leone | 6043.46 (4662.44-7574.57) | 175.25 (135.2-219.65) | 0.13 (0.08-0.21) | 0 (0-0.01) | 9.09 (5.74-13.73) | 0.26 (0.17-0.4) | 26294.82 (22417.01-30630.96) | 4639.42 (3955.22-5404.48) | 7.26 (3.7-11.78) | 1.28 (0.65-2.08) | 298.25 (179.19-457.29) | 28.71 (17.25-44.01) |
| Singapore | 12496.07 (9857.64-15491.21) | 385.57 (304.16-477.98) | 0.58 (0.24-1.43) | 0.02 (0.01-0.04) | 28.76 (14.3-62.3) | 0.89 (0.44-1.92) | 71476.9 (60983.09-82204.81) | 5140.11 (4385.47-5911.59) | 30.4 (10.16-68.76) | 2.19 (0.73-4.94) | 681.19 (366.71-1208.96) | 26.01 (14-46.16) |
| Slovakia | 10627.58 (8367.79-13343.59) | 391.25 (308.06-491.24) | 2.6 (1.65-3.9) | 0.1 (0.06-0.14) | 109.25 (71.22-161.55) | 4.02 (2.62-5.95) | 98532.2 (83357.06-114388.45) | 6131.44 (5187.12-7118.14) | 90.14 (52.04-122.08) | 5.61 (3.24-7.6) | 1868.81 (1262.84-2483.76) | 57.74 (39.01-76.73) |
| Slovenia | 4385.95 (3429.24-5487.08) | 458.03 (358.12-573.03) | 0.69 (0.04-1.69) | 0.07 (0-0.18) | 28.99 (3.38-68.7) | 3.03 (0.35-7.17) | 49741.33 (42626.72-57502.17) | 6968.12 (5971.46-8055.32) | 35.06 (4.47-76.73) | 4.91 (0.63-10.75) | 707.67 (245.76-1335) | 46.07 (16-86.91) |
| Solomon Islands | 1088.37 (858.17-1353.41) | 384.88 (303.48-478.61) | 0.07 (0.03-0.13) | 0.02 (0.01-0.05) | 3.44 (1.91-6.18) | 1.22 (0.68-2.18) | 2870.46 (2417.29-3368.63) | 6100.95 (5137.77-7159.78) | 0.35 (0.24-0.54) | 0.74 (0.51-1.14) | 25.23 (14.84-39.37) | 32.37 (19.05-50.52) |
| Somalia | 11715.02 (9169.62-14627.77) | 155.67 (121.85-194.38) | 3.22 (1.45-5.76) | 0.04 (0.02-0.08) | 139.88 (65.59-245.23) | 1.86 (0.87-3.26) | 41770.62 (35103.52-49706.83) | 4103.29 (3448.35-4882.89) | 27.37 (10.74-48.78) | 2.69 (1.06-4.79) | 810.75 (428.71-1251.3) | 46.99 (24.84-72.52) |
| South Africa | 76866.05 (59775.2-96223.83) | 270.26 (210.17-338.33) | 84.86 (65.16-105.96) | 0.3 (0.23-0.37) | 3461.2 (2701.58-4282.7) | 12.17 (9.5-15.06) | 467706.44 (400209.42-538420.31) | 6184.07 (5291.62-7119.06) | 779.99 (616.79-883.6) | 10.31 (8.16-11.68) | 16859.39 (13536.66-20055.76) | 121.56 (97.6-144.6) |
| South Sudan | 7255.88 (5578.85-9047.77) | 214.84 (165.18-267.89) | 2.55 (1.08-4.63) | 0.08 (0.03-0.14) | 106.71 (46.64-188) | 3.16 (1.38-5.57) | 25606.04 (21832.51-29659.04) | 4201.39 (3582.24-4866.4) | 34.6 (15.97-52.96) | 5.68 (2.62-8.69) | 757.9 (406.2-1131.13) | 71.75 (38.45-107.08) |
| Spain | 187249.23 (148854.31-229816.78) | 851.53 (676.93-1045.11) | 16.33 (6.92-35.4) | 0.07 (0.03-0.16) | 727.64 (354.37-1478.77) | 3.31 (1.61-6.72) | 1577956.42 (1353511.07-1820684.27) | 10524.77 (9027.75-12143.74) | 1873.6 (877.7-3481.14) | 12.5 (5.85-23.22) | 27321.12 (15651.29-45732.14) | 81.6 (46.75-136.59) |
| Sri Lanka | 52774.21 (41192.92-65980.97) | 503.96 (393.37-630.08) | 1.07 (0.64-1.75) | 0.01 (0.01-0.02) | 70.73 (44.82-111.46) | 0.68 (0.43-1.06) | 314645.34 (269004.82-365278.94) | 6905.37 (5903.72-8016.61) | 29.53 (20.22-40.48) | 0.65 (0.44-0.89) | 2230.47 (1313.99-3701.5) | 26.14 (15.4-43.38) |
| Sudan | 34251.79 (26744.82-42386.2) | 194.12 (151.58-240.22) | 1.17 (0.63-1.9) | 0.01 (0-0.01) | 65.6 (40.2-101.66) | 0.37 (0.23-0.58) | 122926.69 (104620.36-142292.74) | 4222.88 (3594.01-4888.16) | 23.77 (14.41-35.91) | 0.82 (0.49-1.23) | 1064.82 (656.02-1661.4) | 20 (12.32-31.21) |
| Suriname | 857.73 (667.1-1080.23) | 308.44 (239.89-388.45) | 0.07 (0.05-0.11) | 0.03 (0.02-0.04) | 3.46 (2.32-4.85) | 1.24 (0.83-1.75) | 5294.85 (4499.84-6129.93) | 5085.9 (4322.27-5888.03) | 1.1 (0.87-1.4) | 1.06 (0.84-1.34) | 47.28 (31.01-72.16) | 24.37 (15.98-37.19) |
| Sweden | 35598.54 (28111.06-43786.98) | 775.72 (612.56-954.15) | 1.39 (0.67-2.85) | 0.03 (0.01-0.06) | 70.25 (37.82-127.85) | 1.53 (0.82-2.79) | 382245.98 (324094.11-443701.64) | 11712.74 (9930.85-13595.85) | 294.52 (134.05-540.36) | 9.02 (4.11-16.56) | 4923.51 (2943.14-7950.37) | 66.28 (39.62-107.02) |
| Switzerland | 34617.3 (27226.73-42667.71) | 817.35 (642.85-1007.42) | 2.29 (1.02-4.93) | 0.05 (0.02-0.12) | 106.8 (54.26-208.7) | 2.52 (1.28-4.93) | 307844.29 (262411.73-355253.42) | 11002.75 (9378.93-12697.21) | 307.08 (150-568.74) | 10.98 (5.36-20.33) | 4686.46 (2735.51-7774.86) | 76.87 (44.87-127.52) |
| Syrian Arab Republic | 23582.08 (18449.7-29501.03) | 365.61 (286.04-457.37) | 0.34 (0.24-0.48) | 0.01 (0-0.01) | 26.51 (17.23-41.07) | 0.41 (0.27-0.64) | 98189.64 (83220.97-114465.52) | 4554.37 (3860.07-5309.3) | 5.62 (4.06-7.41) | 0.26 (0.19-0.34) | 575.95 (312.72-977.22) | 15.38 (8.35-26.09) |
| Taiwan (Province of China) | 96116.06 (75685.87-115512.7) | 784.36 (617.64-942.65) | 1.07 (0.6-1.96) | 0.01 (0-0.02) | 90.87 (52.82-149.93) | 0.74 (0.43-1.22) | 558519.8 (490877.03-621401.39) | 7871.3 (6918-8757.5) | 39.6 (17.47-79.34) | 0.56 (0.25-1.12) | 3355.81 (1793.14-5680.12) | 24.34 (13.01-41.2) |
| Tajikistan | 11680.46 (9183.68-14718.81) | 259.65 (204.15-327.19) | 0.18 (0.13-0.24) | 0 (0-0.01) | 14.35 (9.35-21.55) | 0.32 (0.21-0.48) | 37470.54 (31682.89-43735.69) | 4278.02 (3617.25-4993.32) | 2.25 (1.54-2.85) | 0.26 (0.18-0.33) | 232.3 (125.93-392.27) | 17.34 (9.4-29.28) |
| Thailand | 192080.42 (149036.26-240158.02) | 518.76 (402.51-648.6) | 2.17 (1.44-3.25) | 0.01 (0-0.01) | 195.37 (123.59-307.27) | 0.53 (0.33-0.83) | 1111327.87 (949110.63-1281481.71) | 6130.78 (5235.89-7069.46) | 78.39 (54.53-103.02) | 0.43 (0.3-0.57) | 7158.58 (3923.95-12174.71) | 20.77 (11.38-35.32) |
| Timor-Leste | 1887.76 (1489.18-2356.84) | 353.8 (279.1-441.71) | 0.05 (0.02-0.09) | 0.01 (0-0.02) | 3.25 (1.65-5.15) | 0.61 (0.31-0.97) | 9133.61 (7719.02-10680.05) | 6704.88 (5666.45-7840.1) | 0.96 (0.59-1.44) | 0.7 (0.43-1.06) | 76.33 (42.78-127.65) | 29.93 (16.77-50.05) |
| Togo | 6614.04 (5107.08-8381.22) | 195.62 (151.05-247.89) | 0.19 (0.12-0.29) | 0.01 (0-0.01) | 11.78 (7.56-17.59) | 0.35 (0.22-0.52) | 23953.45 (20410.56-28032.08) | 4101.41 (3494.78-4799.77) | 9.31 (4.34-15) | 1.59 (0.74-2.57) | 329.29 (192.24-499.5) | 32.88 (19.2-49.88) |
| Tokelau | 3.05 (2.4-3.81) | 516.8 (406.33-644.34) | 0 (0-0) | 0.03 (0.02-0.05) | 0.01 (0.01-0.01) | 1.53 (0.89-2.5) | 17.21 (14.64-19.95) | 7378.4 (6274.34-8554.52) | 0 (0-0) | 1.42 (1.02-1.92) | 0.15 (0.1-0.24) | 34.56 (21.73-53.32) |
| Tonga | 221.13 (172.16-277.37) | 515.78 (401.54-646.94) | 0.01 (0.01-0.02) | 0.03 (0.02-0.04) | 0.6 (0.37-0.91) | 1.4 (0.87-2.11) | 1098.96 (931.64-1279.06) | 8416.04 (7134.68-9795.31) | 0.19 (0.14-0.26) | 1.48 (1.05-1.97) | 10.01 (6.14-15.54) | 38.99 (23.93-60.57) |
| Trinidad and Tobago | 2082.09 (1606.14-2614.92) | 301.03 (232.21-378.06) | 0.37 (0.1-0.88) | 0.05 (0.01-0.13) | 15.81 (4.82-35.9) | 2.29 (0.7-5.19) | 17716.55 (15022.56-20647.55) | 5299.74 (4493.86-6176.52) | 11.95 (4.91-22.51) | 3.57 (1.47-6.73) | 266.37 (151.9-457.86) | 41.82 (23.85-71.88) |
| Tunisia | 20587.79 (16037.29-25901.13) | 347.92 (271.02-437.71) | 0.62 (0.37-1) | 0.01 (0.01-0.02) | 35.54 (22.17-52.37) | 0.6 (0.37-0.88) | 121747.69 (104382.3-140747.88) | 5528.23 (4739.71-6390.98) | 22.41 (15.6-30.35) | 1.02 (0.71-1.38) | 913.09 (582.89-1410.28) | 22.22 (14.18-34.31) |
| Turkey | 141769.33 (111412.12-177446.54) | 322.85 (253.72-404.1) | 45.87 (30.5-63.72) | 0.1 (0.07-0.15) | 1918.11 (1296.27-2624.2) | 4.37 (2.95-5.98) | 850038.53 (725958.17-983995.46) | 5609.49 (4790.67-6493.49) | 769.52 (566.3-1242.35) | 5.08 (3.74-8.2) | 15960.74 (12057.13-22334.53) | 55.12 (41.64-77.14) |
| Turkmenistan | 7761 (6056.52-9680.84) | 311.46 (243.06-388.51) | 0.72 (0.29-1.6) | 0.03 (0.01-0.06) | 33 (15.04-68.12) | 1.32 (0.6-2.73) | 34473.71 (29556.77-39615.43) | 5103.08 (4375.24-5864.2) | 7.06 (3.08-13.23) | 1.05 (0.46-1.96) | 305.24 (178.3-499.33) | 26.69 (15.59-43.67) |
| Tuvalu | 25.05 (19.58-31.26) | 460.72 (360.19-575.03) | 0 (0-0) | 0.03 (0.02-0.05) | 0.08 (0.05-0.13) | 1.55 (0.92-2.47) | 127.98 (108.42-148.77) | 7179.67 (6082.32-8346.11) | 0.02 (0.01-0.03) | 1.1 (0.79-1.56) | 1.14 (0.68-1.78) | 35.13 (21-54.68) |
| Uganda | 23317.85 (18154.1-29488.88) | 155.7 (121.22-196.9) | 10 (4.05-17.17) | 0.07 (0.03-0.11) | 416.37 (178.88-702.85) | 2.78 (1.19-4.69) | 92546.18 (78208.71-108062.91) | 4162.03 (3517.24-4859.86) | 122.72 (45.35-190.97) | 5.52 (2.04-8.59) | 2696.74 (1274.96-3953.99) | 68.38 (32.33-100.26) |
| Ukraine | 111643.99 (87172.49-141017.28) | 516.57 (403.34-652.48) | 71.87 (29.51-159.77) | 0.33 (0.14-0.74) | 2867.38 (1187.65-6314.63) | 13.27 (5.5-29.22) | 1204697.26 (1030038.26-1391405.37) | 8943.02 (7646.45-10329.04) | 2873.18 (1372.9-5268.95) | 21.33 (10.19-39.11) | 49744.93 (25473.55-90474.53) | 182.94 (93.68-332.72) |
| United Arab Emirates | 20387.44 (15971.38-25437.04) | 283.8 (222.33-354.1) | 1.78 (0.79-3.55) | 0.02 (0.01-0.05) | 81.14 (41.25-153.21) | 1.13 (0.57-2.13) | 17822.56 (14829.51-21233.58) | 3285.82 (2734.01-3914.69) | 3.36 (1.47-6.64) | 0.62 (0.27-1.22) | 157.1 (88.71-255.16) | 22.41 (12.66-36.4) |
| United Kingdom | 211955.65 (168001.32-259756.57) | 673.34 (533.7-825.19) | 16.29 (6.98-29.76) | 0.05 (0.02-0.09) | 736.36 (354.91-1302.98) | 2.34 (1.13-4.14) | 1950295.98 (1659546.38-2250585.92) | 9653.28 (8214.17-11139.61) | 2263.91 (940.87-3942.72) | 11.21 (4.66-19.52) | 33882.78 (18172.15-56065.51) | 76.42 (40.99-126.46) |
| United Republic of Tanzania | 43124.59 (33385.87-54152.3) | 194.79 (150.8-244.61) | 17.9 (7.24-34.8) | 0.08 (0.03-0.16) | 742.94 (327.6-1410.03) | 3.36 (1.48-6.37) | 181981.25 (154957.87-211489.99) | 4666.78 (3973.78-5423.51) | 295.45 (108.39-463.01) | 7.58 (2.78-11.87) | 6088.38 (2804.95-9069.47) | 85.93 (39.59-128.01) |
| United States of America | 1224147.69 (1048171.74-1406714.3) | 817.45 (699.94-939.36) | 260.63 (121.95-504.21) | 0.17 (0.08-0.34) | 10887.08 (5444.64-20406.35) | 7.27 (3.64-13.63) | 11237227.14 (10056487.43-12470980.46) | 11652.74 (10428.34-12932.11) | 14266.42 (6870.92-26658.63) | 14.79 (7.12-27.64) | 225143.92 (127081.85-391184.44) | 113.37 (63.99-196.97) |
| United States Virgin Islands | 146.23 (113.86-181.98) | 328.77 (255.98-409.15) | 0.16 (0.1-0.24) | 0.36 (0.23-0.54) | 6.37 (4.12-9.36) | 14.32 (9.26-21.05) | 1605.2 (1348.03-1870.07) | 4861.36 (4082.52-5663.53) | 5.61 (4.44-7.46) | 16.99 (13.44-22.6) | 93.75 (74.28-123.35) | 140.25 (111.12-184.52) |
| Uruguay | 9530.65 (7477.53-11789.36) | 597.51 (468.79-739.12) | 0.1 (0.07-0.17) | 0.01 (0-0.01) | 9.4 (5.67-15.05) | 0.59 (0.36-0.94) | 85749.65 (72741.38-99158.9) | 9641.88 (8179.2-11149.64) | 7.35 (3.57-14.23) | 0.83 (0.4-1.6) | 502.91 (271.23-865.03) | 26.16 (14.11-45) |
| Uzbekistan | 46874.51 (36881.65-58721.68) | 278.6 (219.21-349.02) | 2.34 (1.1-5.01) | 0.01 (0.01-0.03) | 118.76 (61.93-226.56) | 0.71 (0.37-1.35) | 152379.94 (127758.25-179766.36) | 4113.34 (3448.7-4852.61) | 17.62 (8.21-34.53) | 0.48 (0.22-0.93) | 1108.23 (621.97-1864.42) | 20.06 (11.26-33.75) |
| Vanuatu | 498.2 (390.57-622.94) | 392.66 (307.83-490.97) | 0.03 (0.01-0.05) | 0.02 (0.01-0.04) | 1.5 (0.87-2.52) | 1.18 (0.69-1.98) | 1987.32 (1682.12-2323.3) | 7025.27 (5946.36-8212.96) | 0.24 (0.17-0.38) | 0.86 (0.61-1.35) | 17.99 (10.57-28.67) | 35.25 (20.71-56.18) |
| Venezuela (Bolivarian Republic of) | 37228.56 (29238.71-46499.89) | 271.14 (212.95-338.67) | 4.12 (1.73-8.29) | 0.03 (0.01-0.06) | 182.99 (85.88-342.89) | 1.33 (0.63-2.5) | 253697.62 (213327.86-294109.63) | 5034.04 (4233-5835.92) | 119.41 (53.31-242.24) | 2.37 (1.06-4.81) | 2878.04 (1726.41-4690.25) | 30.23 (18.13-49.26) |
| Viet Nam | 247523.91 (192552.55-310627.43) | 474.57 (369.18-595.56) | 12.16 (6.71-20.14) | 0.02 (0.01-0.04) | 622.82 (389.68-968.99) | 1.19 (0.75-1.86) | 1077640.48 (915081.82-1247691.3) | 6675.77 (5668.75-7729.2) | 181.15 (131.47-237.03) | 1.12 (0.81-1.47) | 8886.06 (5404.39-13925.18) | 31.23 (19-48.95) |
| Yemen | 25557.4 (19957.6-31981.89) | 195.42 (152.6-244.54) | 0.75 (0.43-1.27) | 0.01 (0-0.01) | 44.21 (27.14-66.83) | 0.34 (0.21-0.51) | 96300.18 (81307.23-111961.9) | 4563.8 (3853.26-5306.03) | 12.38 (8.06-19.67) | 0.59 (0.38-0.93) | 759.87 (440.49-1202.6) | 20.2 (11.71-31.96) |
| Zambia | 12226.89 (9463.25-15388.06) | 164.61 (127.4-207.17) | 6.5 (3.56-11.34) | 0.09 (0.05-0.15) | 269.42 (152.98-457.49) | 3.63 (2.06-6.16) | 44271 (37471.28-51683.98) | 4331.38 (3666.11-5056.65) | 78.37 (47.66-120.79) | 7.67 (4.66-11.82) | 1655.28 (1078.85-2452.91) | 91.5 (59.64-135.59) |
| Zimbabwe | 14407.1 (11213-18029.43) | 229.57 (178.67-287.29) | 0.39 (0.25-0.59) | 0.01 (0-0.01) | 24.44 (16.11-35.9) | 0.39 (0.26-0.57) | 63942.39 (54116.51-74908.65) | 5739.55 (4857.57-6723.9) | 4.39 (2.7-5.95) | 0.39 (0.24-0.53) | 468.75 (261.81-780.97) | 23.66 (13.21-39.42) |

Table S11 The temporal trends of ASCVDs burden in people aged 20-54 in 204 countries and territories.

|  | IHD | | | IS | | | PAD | | |
| --- | --- | --- | --- | --- | --- | --- | --- | --- | --- |
| Location | EAPC of Prevalence | EAPC of death | EAPC of DALY | EAPC of Prevalence | EAPC of death | EAPC of DALY | EAPC of Prevalence | EAPC of death | EAPC of DALY |
| Afghanistan | 0.3 (-0.25-0.84) | -0.41 (-0.51--0.31) | -1.28 (-1.55--1.01) | 1.11 (0.82-1.39) | 2.21 (1.75-2.66) | 1.94 (1.6-2.28) | 0.29 (-0.52-1.1) | 2.28 (1.31-3.25) | 0.62 (0.48-0.77) |
| Albania | 0.97 (0.77-1.17) | -0.66 (-0.79--0.54) | 1.19 (0.75-1.63) | 0.99 (0.87-1.11) | 0.75 (0.34-1.17) | 0.11 (0.02-0.21) | 1.95 (1.68-2.22) | 4.49 (3.78-5.21) | 2.04 (1.67-2.41) |
| Algeria | 1 (0.91-1.1) | 0.38 (0.14-0.63) | -2.13 (-2.31--1.94) | 0.66 (0.59-0.72) | 0.85 (0.63-1.08) | 1.56 (1.37-1.75) | 1.17 (1.05-1.3) | 3.55 (3.23-3.87) | 0.74 (0.62-0.86) |
| American Samoa | 1.58 (1.44-1.72) | -0.13 (-0.28-0.02) | 1.32 (1.2-1.44) | 0.85 (0.77-0.92) | 0.5 (0.26-0.74) | 2.12 (1.87-2.36) | 2.44 (2.23-2.66) | 4.93 (2.76-7.15) | 3.22 (3.04-3.4) |
| Andorra | 1.03 (0.86-1.21) | -0.99 (-1.14--0.83) | -0.79 (-0.96--0.61) | 0.57 (0.44-0.69) | -1.64 (-1.89--1.39) | -0.52 (-0.76--0.28) | 1.07 (0.88-1.25) | 3.17 (2.7-3.64) | 1.29 (1.15-1.43) |
| Angola | -0.03 (-0.06-0.01) | 0.3 (0.18-0.42) | -1.15 (-1.25--1.06) | -0.02 (-0.07-0.02) | -0.62 (-0.72--0.53) | 0.34 (0.2-0.48) | 0.47 (0.39-0.55) | 1.03 (0.85-1.21) | 2.68 (2.47-2.88) |
| Antigua and Barbuda | 1.14 (1.08-1.2) | -1.39 (-1.62--1.15) | -1.74 (-1.92--1.56) | 0.6 (0.57-0.62) | -0.55 (-0.78--0.32) | -0.37 (-0.72--0.03) | 1.78 (1.68-1.88) | 2.76 (2.52-3) | 3.55 (3.17-3.94) |
| Argentina | -0.32 (-0.34--0.3) | 1.68 (1.25-2.11) | -3.04 (-3.23--2.86) | -0.44 (-0.51--0.37) | -4.36 (-4.65--4.06) | -2.96 (-3.28--2.63) | -0.93 (-1--0.87) | 0.56 (0.46-0.67) | -0.23 (-0.39--0.07) |
| Armenia | 0.83 (0.52-1.14) | 1.04 (0.87-1.22) | -0.48 (-0.84--0.12) | -0.27 (-0.42--0.11) | -1.48 (-2.06--0.9) | 0.24 (0.15-0.33) | 1.34 (0.87-1.82) | 2.13 (1.72-2.54) | 2.22 (2-2.44) |
| Australia | 0.18 (-0.02-0.38) | -1.28 (-1.55--1.01) | -2.7 (-2.8--2.59) | 0.07 (0-0.14) | -3.43 (-3.61--3.26) | 0.58 (0.36-0.79) | -0.75 (-0.81--0.69) | 1.25 (0.99-1.5) | 1.65 (1.3-1.99) |
| Austria | 0.54 (0.46-0.62) | -3.7 (-3.92--3.48) | -3.32 (-3.6--3.04) | 0.06 (-0.03-0.15) | -6.47 (-6.74--6.21) | 0.32 (0.13-0.5) | 0.04 (-0.08-0.17) | 1.42 (1.31-1.54) | 0.5 (0.17-0.82) |
| Azerbaijan | 1.28 (0.98-1.59) | -0.03 (-0.17-0.11) | -0.77 (-1.02--0.52) | 0.79 (0.61-0.96) | 0.99 (0.45-1.53) | -1.58 (-1.71--1.46) | 2.15 (1.68-2.62) | 2.65 (2.18-3.12) | 0.44 (0.21-0.67) |
| Bahamas | 1.37 (1.29-1.46) | -0.37 (-0.49--0.25) | -0.03 (-0.22-0.16) | 0.56 (0.48-0.64) | 0.39 (0.11-0.66) | 0.74 (0.09-1.41) | 1.72 (1.62-1.82) | 2.89 (2.68-3.09) | 2.6 (1.63-3.59) |
| Bahrain | 1.42 (1.22-1.62) | -0.09 (-0.21-0.04) | -4.29 (-4.9--3.67) | 0.61 (0.47-0.75) | -1.18 (-1.5--0.87) | -0.24 (-0.66-0.18) | 2.31 (2.12-2.5) | 2.4 (1.85-2.95) | 2.08 (1.69-2.47) |
| Bangladesh | 1.05 (1-1.11) | 0.64 (0.38-0.9) | 0.57 (0.31-0.82) | 0.77 (0.59-0.95) | 0.24 (0.05-0.42) | -0.49 (-0.72--0.27) | 1.13 (1.03-1.23) | 4.08 (3.79-4.37) | 0.75 (0.62-0.87) |
| Barbados | 1.42 (1.24-1.59) | -0.67 (-1.02--0.32) | -1.24 (-1.42--1.06) | 0.52 (0.46-0.59) | -0.53 (-0.67--0.39) | 0.14 (-0.01-0.29) | 1.83 (1.62-2.05) | 2.33 (2.15-2.51) | 1.64 (1.17-2.1) |
| Belarus | 0.72 (0.49-0.94) | -0.43 (-0.53--0.33) | -0.49 (-1.23-0.26) | 0.47 (0.25-0.68) | -1.33 (-2.06--0.58) | 2.53 (2-3.05) | 1.03 (0.78-1.28) | 3.14 (2.73-3.56) | 3.9 (3.53-4.27) |
| Belgium | 0.64 (0.48-0.8) | -0.86 (-1.14--0.59) | -3.4 (-3.64--3.16) | -0.08 (-0.15--0.01) | -3.92 (-4.08--3.76) | -0.15 (-0.34-0.04) | -0.19 (-0.34--0.03) | 1.02 (0.72-1.32) | 0.29 (0.09-0.48) |
| Belize | 1.11 (1.03-1.18) | -0.47 (-0.64--0.31) | -1.1 (-1.5--0.7) | 0.65 (0.61-0.68) | 0.33 (-0.01-0.68) | 2.21 (1.93-2.5) | 1.19 (1.1-1.27) | 2.62 (2.39-2.84) | 2.92 (2.69-3.15) |
| Benin | 0.4 (0.37-0.44) | 1.11 (0.98-1.25) | -0.37 (-0.49--0.25) | -0.1 (-0.2--0.01) | -1.13 (-1.3--0.97) | -1.87 (-2.12--1.62) | 0.27 (0.19-0.35) | 0.01 (-0.07-0.08) | 2.43 (2.24-2.61) |
| Bermuda | 0.95 (0.84-1.05) | 0.18 (0.07-0.3) | -2.73 (-3.06--2.41) | 0.31 (0.3-0.33) | -2.02 (-2.34--1.7) | 0.38 (0.25-0.5) | 1.58 (1.43-1.72) | 1.73 (1.62-1.84) | 1.88 (1.6-2.16) |
| Bhutan | 0.13 (0.04-0.22) | -0.15 (-0.3--0.01) | -0.81 (-0.95--0.67) | 0.01 (-0.1-0.11) | -1.26 (-1.43--1.09) | 0.5 (0.34-0.66) | 0.11 (-0.01-0.24) | 2.44 (2.27-2.6) | 2.37 (1.85-2.89) |
| Bolivia (Plurinational State of) | 0.41 (0.39-0.44) | 0.64 (0.43-0.86) | -1.76 (-1.91--1.6) | -0.25 (-0.37--0.14) | -2.1 (-2.4--1.79) | -0.35 (-0.41--0.29) | 0.17 (0.15-0.2) | 1.12 (1.03-1.2) | 0.8 (0.65-0.95) |
| Bosnia and Herzegovina | 0.65 (0.58-0.73) | 0.24 (-0.21-0.7) | -1.85 (-2.09--1.6) | 0.9 (0.79-1.02) | -1.3 (-1.53--1.08) | 1.32 (1.16-1.48) | 1.52 (1.46-1.58) | 2.84 (2.46-3.21) | 1.62 (1.45-1.79) |
| Botswana | 0.7 (0.61-0.78) | 0.71 (0.58-0.84) | -0.22 (-0.89-0.45) | 0.02 (-0.05-0.1) | -1.01 (-1.51--0.5) | 0.66 (0.49-0.83) | 0.43 (0.37-0.5) | 0.7 (0.33-1.07) | 1.63 (1.56-1.7) |
| Brazil | 0.69 (0.65-0.72) | -0.64 (-0.88--0.41) | -1.46 (-1.53--1.39) | -0.87 (-1.12--0.61) | -3.78 (-4.03--3.53) | 0.49 (0.28-0.69) | 0.31 (0.25-0.36) | 1.16 (1.12-1.2) | 1.36 (0.88-1.83) |
| Brunei Darussalam | 0.77 (0.69-0.85) | -1.67 (-1.92--1.42) | -0.73 (-1.13--0.33) | 0.16 (0.07-0.25) | -2.16 (-2.58--1.75) | -0.38 (-0.49--0.28) | 0.39 (0.27-0.51) | 1.9 (1.78-2.02) | 2.71 (2.47-2.95) |
| Bulgaria | 0.19 (0.08-0.3) | 0.77 (0.62-0.91) | -1.57 (-2.04--1.09) | 0.44 (0.37-0.5) | 0.4 (0.18-0.63) | -0.52 (-1.1-0.07) | 0.16 (0.08-0.25) | 0.37 (0.13-0.62) | 1.71 (1.1-2.33) |
| Burkina Faso | -0.1 (-0.19-0) | -0.61 (-0.81--0.42) | -0.13 (-0.28-0.02) | -0.22 (-0.31--0.13) | -1.08 (-1.31--0.85) | -2.13 (-2.35--1.9) | -0.26 (-0.33--0.18) | 0.13 (-0.15-0.41) | 0.1 (-0.03-0.22) |
| Burundi | 0.15 (-0.03-0.33) | 0.2 (0.08-0.33) | -1.39 (-1.62--1.15) | -0.63 (-0.74--0.52) | -2.47 (-2.71--2.23) | -0.64 (-0.85--0.43) | -0.02 (-0.22-0.18) | -1.11 (-1.28--0.94) | -0.45 (-0.67--0.23) |
| Cabo Verde | 1.68 (1.53-1.84) | -3.11 (-3.57--2.64) | 0.64 (0.51-0.78) | 0.44 (0.38-0.5) | 0.15 (0-0.29) | 0.26 (0.01-0.51) | 1.74 (1.42-2.05) | 1.8 (1.56-2.05) | 2.61 (2.22-3) |
| Cambodia | 0.45 (0.37-0.52) | -1.1 (-1.36--0.84) | -0.83 (-0.93--0.73) | 0.22 (0.12-0.32) | -0.77 (-0.89--0.64) | 1.56 (1.29-1.84) | 0.79 (0.66-0.92) | 2.17 (2.1-2.24) | 1.3 (1.1-1.51) |
| Cameroon | 0.4 (0.34-0.46) | 1.07 (0.75-1.4) | 0.65 (0.31-1) | 0.15 (0.07-0.22) | -0.27 (-0.57-0.03) | 0.49 (0.33-0.65) | -0.02 (-0.07-0.03) | 0.51 (0.35-0.67) | 1.94 (1.71-2.17) |
| Canada | -0.04 (-0.16-0.08) | -0.81 (-0.95--0.67) | -2.15 (-2.28--2.02) | 0.02 (-0.25-0.29) | -2.97 (-3.09--2.84) | -2.08 (-2.37--1.79) | -0.6 (-0.74--0.45) | 1.58 (1.29-1.87) | 0.66 (0.47-0.84) |
| Central African Republic | 0.48 (0.4-0.56) | 0.09 (-0.27-0.45) | 0.3 (0.18-0.42) | 0.19 (0.1-0.28) | 0.28 (0.1-0.46) | 0.22 (-0.04-0.47) | 0.5 (0.38-0.62) | 0.42 (0.26-0.58) | 0.32 (-0.2-0.83) |
| Chad | 0.43 (0.33-0.52) | 0.91 (0.74-1.08) | 0.38 (0.14-0.63) | 0.09 (0.04-0.13) | -0.64 (-0.76--0.52) | -1.29 (-1.65--0.93) | 0 (-0.04-0.03) | 0.34 (0.27-0.4) | 0.42 (0.16-0.67) |
| Chile | 0.73 (0.67-0.8) | -0.83 (-0.93--0.73) | 0.19 (-0.02-0.41) | 0.41 (0.28-0.55) | -1.72 (-1.9--1.54) | -1.07 (-1.21--0.94) | 0.43 (0.32-0.54) | 2.9 (2.59-3.21) | -1.93 (-2.21--1.66) |
| China | 1.59 (1.44-1.74) | -1.15 (-1.25--1.06) | 0.63 (0.56-0.7) | 0.73 (0.54-0.93) | 0.36 (0.13-0.59) | 0.69 (0.55-0.83) | 2.34 (2.16-2.52) | 1.64 (1.56-1.73) | 1.73 (1.59-1.86) |
| Colombia | 0.88 (0.81-0.96) | 2.29 (1.93-2.65) | -2 (-2.16--1.83) | -0.35 (-0.43--0.26) | -4.34 (-4.75--3.93) | -3.16 (-3.45--2.88) | 0.71 (0.65-0.78) | 1.5 (1.36-1.63) | 0.4 (0.21-0.58) |
| Comoros | 0.58 (0.5-0.66) | 0.57 (0.31-0.82) | 0.09 (-0.27-0.45) | -0.65 (-0.76--0.53) | -1.01 (-1.46--0.55) | -1.67 (-1.96--1.38) | 0.67 (0.51-0.83) | 0.38 (-0.04-0.81) | 4.21 (3.79-4.64) |
| Congo | 0.76 (0.55-0.97) | 0.71 (0.58-0.85) | -1.33 (-1.57--1.09) | -0.26 (-0.38--0.15) | -0.99 (-1.22--0.76) | -2.29 (-2.44--2.14) | 0.64 (0.41-0.86) | 0.97 (0.56-1.39) | 1.29 (1.13-1.45) |
| Cook Islands | 1.32 (1.16-1.47) | 0.65 (0.31-1) | 0.4 (0.03-0.77) | 0.97 (0.87-1.06) | 0.02 (-0.34-0.39) | -0.36 (-0.42--0.31) | 2.12 (1.91-2.33) | 3.16 (2.66-3.67) | -0.33 (-0.57--0.1) |
| Costa Rica | 0.99 (0.86-1.12) | -0.58 (-0.76--0.41) | -0.83 (-1.07--0.59) | 0.34 (0.31-0.38) | -1.59 (-2.02--1.16) | 0.02 (-0.13-0.18) | 1.08 (0.91-1.25) | 2.46 (2.21-2.71) | 2.45 (2.36-2.54) |
| Croatia | 0.04 (-0.13-0.21) | -1.66 (-1.78--1.55) | -2.46 (-2.66--2.25) | -0.35 (-0.46--0.25) | -3.18 (-3.48--2.88) | -1.33 (-1.62--1.03) | 0.06 (-0.1-0.23) | 0.8 (0.51-1.09) | 2.98 (2.73-3.23) |
| Cte d'Ivoire | 1.31 (1.21-1.4) | -1.8 (-1.92--1.68) | -1.48 (-1.78--1.17) | 0.66 (0.54-0.78) | 0.61 (0.24-0.98) | -0.95 (-1.07--0.83) | 1.79 (1.58-2) | 2.9 (2.6-3.2) | 0.48 (0.33-0.64) |
| Cuba | 0.37 (0.23-0.51) | 0.82 (0.58-1.06) | -2.7 (-2.92--2.48) | 0.2 (0.08-0.33) | -3.83 (-4.15--3.5) | 1.08 (0.92-1.24) | -0.66 (-0.78--0.55) | 1.1 (0.93-1.28) | -0.48 (-0.71--0.24) |
| Cyprus | -0.42 (-0.58--0.25) | -1.12 (-1.43--0.81) | -4.71 (-4.96--4.46) | -0.9 (-1.07--0.73) | -5.93 (-6.24--5.62) | 0.23 (0-0.46) | -0.07 (-0.28-0.14) | -0.04 (-0.36-0.28) | 0.92 (0.61-1.23) |
| Czechia | 0.59 (0.53-0.65) | 3.23 (2.71-3.76) | -0.64 (-0.88--0.41) | -0.2 (-0.25--0.14) | -1.39 (-1.61--1.17) | -0.49 (-0.69--0.29) | 0.44 (0.34-0.53) | 0.13 (-0.15-0.41) | 1.37 (1.12-1.63) |
| Democratic People's Republic of Korea | 1.01 (0.85-1.16) | 2.27 (1.75-2.78) | 0.95 (0.84-1.06) | 0.62 (0.5-0.74) | 0.63 (0.46-0.8) | 0.86 (0.73-1) | 1.06 (0.78-1.34) | 1.38 (1.1-1.67) | 2.36 (1.89-2.83) |
| Democratic Republic of the Congo | 0.12 (0.07-0.16) | 1.41 (1.15-1.66) | -0.43 (-0.53--0.33) | -0.23 (-0.3--0.15) | -0.7 (-0.76--0.64) | -0.67 (-1.12--0.21) | 0.04 (-0.02-0.1) | -0.15 (-0.46-0.16) | 3.93 (3.71-4.15) |
| Denmark | -0.39 (-0.49--0.29) | -1.33 (-1.55--1.12) | -4.9 (-5.08--4.73) | -0.67 (-0.78--0.56) | -4.38 (-4.75--4.02) | -0.7 (-1.04--0.37) | -0.95 (-1.05--0.86) | -0.34 (-0.66--0.02) | 2.72 (2.54-2.89) |
| Djibouti | 0.82 (0.74-0.9) | -1.9 (-1.97--1.84) | 0.91 (0.74-1.08) | 0.31 (0.23-0.4) | 0.54 (0.4-0.69) | -1.76 (-1.99--1.52) | 0.74 (0.62-0.87) | 0.91 (0.69-1.12) | 2.58 (2.32-2.84) |
| Dominica | 1.34 (1.23-1.46) | -0.04 (-0.43-0.36) | -1.21 (-1.32--1.1) | 0.62 (0.57-0.67) | 0.23 (0.15-0.3) | -2.61 (-2.94--2.28) | 1.51 (1.4-1.63) | 3 (2.84-3.15) | -0.76 (-0.99--0.52) |
| Dominican Republic | 1.22 (1.09-1.34) | -2.16 (-2.32--2.01) | 2.04 (1.79-2.29) | 0.83 (0.79-0.87) | 1.31 (1.07-1.56) | -1.39 (-2.15--0.64) | 1.05 (0.95-1.15) | 3.47 (3.26-3.69) | 1.46 (1.08-1.84) |
| Ecuador | 0.87 (0.82-0.91) | 0.64 (0.51-0.78) | -0.63 (-0.85--0.4) | 0.04 (-0.02-0.1) | -1.81 (-2.07--1.55) | -1.62 (-1.88--1.36) | 0.75 (0.67-0.82) | 3.88 (3.48-4.28) | 1.43 (1.23-1.62) |
| Egypt | 0.4 (0.38-0.43) | -3.14 (-3.57--2.72) | -0.64 (-0.73--0.56) | 1.29 (1.17-1.4) | 2.87 (2.5-3.25) | 1.21 (0.56-1.88) | 0.57 (0.56-0.59) | 2.66 (2.51-2.82) | 3.45 (2.97-3.92) |
| El Salvador | 1.04 (0.96-1.13) | -0.48 (-0.56--0.4) | -1.51 (-1.96--1.06) | 0.41 (0.33-0.5) | -2.68 (-3.36--1.99) | -2.54 (-2.62--2.47) | 0.76 (0.69-0.84) | 0.31 (0.14-0.48) | -1.42 (-1.61--1.23) |
| Equatorial Guinea | -1.14 (-1.24--1.03) | -0.12 (-0.41-0.18) | -4.97 (-5.5--4.43) | -0.85 (-0.94--0.76) | -4.06 (-4.45--3.66) | -2.4 (-2.77--2.02) | -1.18 (-1.26--1.1) | 0.38 (0.17-0.58) | -0.43 (-1.14-0.28) |
| Eritrea | 0.15 (0.09-0.2) | 1.29 (1.08-1.49) | 0.18 (0.07-0.3) | -0.25 (-0.32--0.18) | -0.57 (-0.69--0.46) | -0.4 (-0.6--0.2) | 0.15 (0.07-0.23) | 0.31 (0.19-0.43) | 1.83 (1.68-1.97) |
| Estonia | 0.19 (0.12-0.26) | -0.5 (-0.57--0.42) | -5.93 (-6.54--5.32) | -0.95 (-1.09--0.81) | -8.54 (-9.58--7.48) | -3.33 (-3.62--3.05) | 0.23 (0.16-0.29) | 1.73 (1.48-1.99) | 1 (0.89-1.11) |
| Eswatini | 0.18 (0.08-0.27) | -0.26 (-0.51--0.01) | 1.34 (0.67-2.02) | 0.06 (-0.02-0.14) | 0.43 (-0.11-0.97) | -2.28 (-2.52--2.05) | -0.04 (-0.09-0) | 1.28 (1.09-1.47) | 0.97 (0.3-1.64) |
| Ethiopia | 0.02 (-0.02-0.07) | 0.95 (0.84-1.06) | -3.7 (-3.92--3.48) | -0.14 (-0.27--0.01) | -3.55 (-3.81--3.29) | 2.88 (2.44-3.32) | -0.49 (-0.55--0.42) | -1.07 (-1.35--0.79) | 3.19 (2.49-3.91) |
| Fiji | 0.88 (0.83-0.94) | -4.3 (-4.74--3.87) | -0.39 (-0.5--0.27) | 0.43 (0.37-0.49) | -0.16 (-0.34-0.03) | -1.39 (-1.66--1.12) | 1.29 (1.1-1.48) | 3.33 (2.93-3.73) | 0.78 (0.63-0.93) |
| Finland | -0.87 (-0.95--0.8) | -1.76 (-1.91--1.6) | -4.42 (-4.7--4.13) | -0.14 (-0.22--0.06) | -5.36 (-5.61--5.1) | -0.79 (-0.93--0.64) | -1.2 (-1.32--1.07) | 0.4 (0.05-0.75) | 4.22 (3.76-4.68) |
| France | 0.39 (0.3-0.47) | -0.01 (-0.16-0.13) | -1.97 (-2.31--1.64) | 0.48 (0.37-0.59) | -1.98 (-2.37--1.59) | 0.78 (0.7-0.87) | -0.35 (-0.46--0.24) | 0.81 (0.39-1.24) | 1.6 (1.44-1.75) |
| Gabon | 0.6 (0.5-0.69) | -1.33 (-1.57--1.09) | -0.74 (-0.92--0.55) | 0.16 (0.11-0.2) | -0.29 (-0.56--0.01) | -0.65 (-0.83--0.47) | 0.9 (0.74-1.06) | 1.17 (0.97-1.36) | 2.09 (1.94-2.24) |
| Gambia | 0.51 (0.46-0.57) | -1.51 (-1.96--1.06) | 0.64 (0.43-0.86) | 0.12 (0.05-0.18) | -0.25 (-0.48--0.02) | -0.21 (-0.68-0.26) | 0.36 (0.31-0.42) | 0.6 (0.35-0.84) | 0.65 (0.5-0.8) |
| Georgia | 1.02 (0.8-1.24) | 1.34 (0.67-2.02) | -2.73 (-3.2--2.26) | 0.53 (0.36-0.69) | 1.61 (1.04-2.17) | 1.56 (1.51-1.62) | 1.42 (1.14-1.71) | 3.21 (2.74-3.68) | 2.28 (2.13-2.44) |
| Germany | 0.08 (-0.05-0.2) | 0.78 (0.68-0.89) | -3.01 (-3.22--2.79) | 0.07 (-0.07-0.22) | -2.98 (-3.41--2.56) | 0.47 (0.33-0.61) | -0.79 (-0.92--0.66) | 1.11 (0.87-1.35) | 1.51 (1.43-1.6) |
| Ghana | 0.32 (0.22-0.41) | -1.82 (-2.06--1.57) | -0.26 (-0.51--0.01) | 0.21 (0.14-0.28) | -0.22 (-0.37--0.07) | -1.49 (-1.89--1.08) | 0.29 (0.22-0.36) | 0.61 (0.47-0.74) | 1.36 (1.1-1.63) |
| Greece | 0.61 (0.42-0.8) | 0.5 (0.33-0.67) | 0.26 (0.11-0.4) | 0.28 (0.15-0.41) | -2.58 (-2.76--2.39) | -1.88 (-1.98--1.77) | -0.39 (-0.63--0.14) | 1.22 (0.9-1.54) | -0.03 (-0.3-0.25) |
| Greenland | 0.67 (0.52-0.82) | 2.04 (1.79-2.29) | -1.73 (-1.88--1.57) | 0.1 (-0.14-0.33) | -2.98 (-3.18--2.77) | 0.26 (0.1-0.42) | 0.6 (0.36-0.84) | 1.64 (1.16-2.11) | 7.49 (4.88-10.18) |
| Grenada | 1.43 (1.38-1.48) | -1.44 (-1.98--0.9) | -1.54 (-1.82--1.26) | 0.46 (0.39-0.53) | -0.89 (-1.24--0.54) | -0.8 (-1.05--0.54) | 1.76 (1.67-1.86) | 3.09 (3-3.18) | 0.43 (0.11-0.75) |
| Guam | 2.01 (1.75-2.27) | -1.1 (-1.5--0.7) | 2.66 (2.24-3.09) | 1.74 (1.55-1.94) | 2.17 (1.8-2.55) | -0.37 (-0.47--0.27) | 2.64 (2.36-2.92) | 7.52 (4.6-10.53) | 1.83 (1.47-2.19) |
| Guatemala | 0.42 (0.37-0.46) | -0.8 (-1.14--0.46) | -3.14 (-3.57--2.72) | -0.39 (-0.51--0.26) | -3.08 (-3.41--2.75) | -0.4 (-0.54--0.26) | -0.27 (-0.31--0.22) | 0.13 (0.02-0.23) | -0.82 (-1.11--0.53) |
| Guinea | 0.35 (0.27-0.43) | -0.38 (-0.55--0.21) | 1.04 (0.87-1.22) | 0.27 (0.2-0.34) | 0.01 (-0.09-0.11) | -1.01 (-1.18--0.85) | -0.21 (-0.29--0.13) | -0.15 (-0.24--0.06) | -0.07 (-0.18-0.04) |
| Guinea-Bissau | 0.17 (0.12-0.22) | -0.33 (-0.84-0.19) | -0.09 (-0.21-0.04) | -0.07 (-0.12--0.02) | -0.46 (-0.53--0.38) | -1.28 (-1.49--1.06) | -0.27 (-0.3--0.23) | 0.08 (0.04-0.13) | 1 (0.76-1.23) |
| Guyana | 1.18 (0.96-1.39) | 0.36 (0.13-0.59) | -0.3 (-0.5--0.11) | 0.35 (0.22-0.47) | -0.8 (-0.96--0.63) | -0.94 (-1.15--0.74) | 1.66 (1.4-1.93) | 3.7 (3.46-3.95) | 1.19 (1.09-1.3) |
| Haiti | -0.08 (-0.13--0.03) | -0.3 (-0.5--0.11) | -1.1 (-1.36--0.84) | -0.1 (-0.22-0.02) | -0.7 (-0.97--0.44) | -1.15 (-1.47--0.82) | -0.26 (-0.32--0.2) | 0.4 (0.17-0.63) | 0.89 (0.67-1.11) |
| Honduras | 0.44 (0.41-0.47) | 0.23 (-0.16-0.62) | -1.66 (-1.78--1.55) | -0.02 (-0.1-0.06) | -0.94 (-1.12--0.77) | -0.74 (-0.98--0.49) | 0.03 (0-0.05) | -0.11 (-0.24-0.02) | 1.41 (1.32-1.51) |
| Hungary | -0.71 (-0.86--0.56) | -0.52 (-1.08-0.05) | -3.79 (-4.04--3.54) | -1.32 (-1.4--1.25) | -4.6 (-4.94--4.25) | 0.19 (-0.18-0.55) | -0.16 (-0.33-0.01) | 0.01 (-0.18-0.21) | 0.78 (0.54-1.03) |
| Iceland | 0.32 (0.16-0.49) | 5.06 (4.12-6.02) | -2.82 (-2.99--2.64) | -0.16 (-0.25--0.07) | -5.32 (-5.67--4.98) | -0.82 (-1.07--0.58) | -0.57 (-0.77--0.37) | 0.97 (0.71-1.23) | 0.71 (0.45-0.97) |
| India | 0.5 (0.35-0.65) | 0.52 (0.32-0.71) | -0.01 (-0.16-0.13) | 0.5 (0.36-0.64) | 0.06 (-0.13-0.26) | -0.59 (-0.66--0.52) | 0.38 (0.34-0.42) | 2.51 (2.35-2.67) | 1.55 (1.45-1.64) |
| Indonesia | 1.28 (1.2-1.37) | -0.31 (-0.48--0.13) | 0.86 (0.74-0.98) | 0.91 (0.82-1) | 1.47 (1.33-1.61) | 1.36 (1.07-1.64) | 1.66 (1.54-1.77) | 2.63 (2.58-2.67) | 1.88 (1.61-2.15) |
| Iran (Islamic Republic of) | 0.94 (0.84-1.05) | 2.05 (1.53-2.57) | -2.29 (-2.48--2.09) | 0.04 (-0.1-0.18) | -1.9 (-2.04--1.75) | 2.04 (1.74-2.34) | 1.25 (1.07-1.44) | 5.27 (4.29-6.26) | 2.58 (2.38-2.78) |
| Iraq | 0.35 (0.24-0.46) | -2 (-2.16--1.83) | -1.89 (-2.17--1.61) | -0.04 (-0.12-0.05) | -0.74 (-0.86--0.63) | 0.21 (-0.04-0.45) | 0.14 (-0.03-0.31) | 0.47 (0.27-0.68) | 0.67 (0.46-0.87) |
| Ireland | -0.23 (-0.36--0.1) | -0.22 (-0.89-0.45) | -4.13 (-4.35--3.91) | -0.13 (-0.26-0) | -4.87 (-5.2--4.54) | 1.79 (1.51-2.08) | -0.92 (-1.1--0.73) | 0.39 (0.18-0.6) | 1.44 (1.2-1.67) |
| Israel | -0.03 (-0.17-0.11) | -0.75 (-1.23--0.26) | -5.07 (-5.35--4.8) | -0.2 (-0.3--0.1) | -4.39 (-4.61--4.16) | -1.12 (-1.34--0.9) | -0.75 (-0.93--0.57) | 0.89 (0.57-1.22) | 0.39 (0.18-0.59) |
| Italy | 0.31 (0.13-0.48) | 0.6 (0.54-0.67) | -2.25 (-2.4--2.09) | 0.19 (0.05-0.33) | -4.77 (-5.29--4.25) | 0.28 (0.24-0.33) | -0.72 (-1.03--0.42) | 0.15 (-0.06-0.36) | 2.71 (2.13-3.3) |
| Jamaica | 1.45 (1.32-1.58) | -0.53 (-0.76--0.29) | 0.9 (0.43-1.37) | 0.91 (0.86-0.96) | -0.32 (-0.94-0.29) | -1.1 (-1.38--0.83) | 1.68 (1.53-1.84) | 3.04 (2.75-3.32) | 1.2 (1.08-1.33) |
| Japan | -0.32 (-0.5--0.14) | -1.46 (-1.53--1.39) | -0.93 (-1.05--0.81) | 0.76 (0.64-0.87) | -3.73 (-4.04--3.43) | -2.03 (-2.34--1.72) | -1.68 (-2.02--1.33) | -0.02 (-0.25-0.2) | 3.15 (2.9-3.4) |
| Jordan | 0.76 (0.51-1.02) | -0.63 (-0.85--0.4) | -2.29 (-2.58--2) | -0.19 (-0.31--0.07) | -3.08 (-3.45--2.72) | -1.73 (-1.9--1.56) | 1.08 (0.72-1.44) | 1.28 (0.91-1.66) | 0.12 (-0.01-0.25) |
| Kazakhstan | 0.06 (-0.14-0.26) | 1.09 (0.92-1.27) | -3.3 (-4.45--2.15) | -0.41 (-0.68--0.14) | -1.57 (-2.48--0.65) | 1.02 (0.83-1.22) | 0.78 (0.54-1.02) | 1.93 (1.73-2.13) | 2.53 (2.11-2.95) |
| Kenya | 0.46 (0.4-0.52) | -2.13 (-2.51--1.75) | 2.27 (1.75-2.78) | 0.16 (0.09-0.23) | 0.81 (0.52-1.11) | -2.28 (-2.43--2.12) | 0.46 (0.38-0.54) | 1.68 (1.6-1.75) | 0.49 (0.03-0.96) |
| Kiribati | 0.64 (0.5-0.79) | 1.08 (0.84-1.33) | -0.48 (-0.56--0.4) | -0.04 (-0.12-0.04) | -0.24 (-0.43--0.05) | -1.39 (-1.57--1.2) | 1.75 (1.54-1.95) | 0.27 (-0.26-0.81) | -0.32 (-0.39--0.24) |
| Kuwait | 1.18 (1.08-1.29) | -2.13 (-2.31--1.94) | -0.69 (-1.07--0.3) | 0.77 (0.65-0.88) | 0.06 (-0.79-0.91) | -2.62 (-2.94--2.3) | 1.83 (1.67-1.99) | 1.84 (1.65-2.02) | -1.01 (-1.2--0.81) |
| Kyrgyzstan | 0.6 (0.37-0.82) | -0.74 (-0.92--0.55) | -1.44 (-1.98--0.9) | -0.44 (-0.56--0.33) | -1.24 (-1.66--0.83) | 0.23 (-0.02-0.48) | 1.47 (1.12-1.82) | 1.99 (1.61-2.38) | 2.19 (1.97-2.42) |
| Lao People's Democratic Republic | -0.06 (-0.19-0.07) | -0.64 (-0.73--0.56) | -0.58 (-0.76--0.41) | -0.41 (-0.49--0.34) | -0.27 (-0.42--0.12) | -0.42 (-0.57--0.27) | 0.26 (0.16-0.36) | 2 (1.9-2.11) | 0.42 (0.26-0.59) |
| Latvia | 0.49 (0.43-0.56) | 0.86 (0.74-0.98) | -3.92 (-4.52--3.32) | 0.06 (-0.02-0.14) | -2.68 (-3.23--2.12) | -0.03 (-0.13-0.07) | 0.92 (0.77-1.07) | 3.49 (3.16-3.82) | 0.75 (0.7-0.8) |
| Lebanon | 0.25 (0.15-0.36) | -0.39 (-0.5--0.27) | -1.61 (-2.05--1.16) | 0.51 (0.37-0.65) | 1.01 (0.62-1.4) | -3.37 (-3.57--3.16) | 0.14 (0.03-0.25) | 2.09 (1.87-2.31) | 0.26 (0.16-0.35) |
| Lesotho | 0.34 (0.29-0.39) | -1.48 (-1.78--1.17) | 3.23 (2.71-3.76) | 0.54 (0.49-0.59) | 2.74 (2.25-3.23) | -0.97 (-1.39--0.55) | 0.14 (0.04-0.24) | 1.64 (1.54-1.75) | 2.76 (2.44-3.07) |
| Liberia | 0.17 (-0.1-0.44) | -1.54 (-1.82--1.26) | -0.67 (-1.02--0.32) | -0.18 (-0.4-0.03) | -1.47 (-1.82--1.12) | 2.8 (2.53-3.07) | -0.3 (-0.66-0.05) | -0.07 (-0.61-0.47) | 2.49 (1.89-3.09) |
| Libya | 1.18 (0.82-1.54) | -2.29 (-2.48--2.09) | 0.28 (-0.12-0.68) | 1.88 (1.68-2.08) | 3.27 (2.93-3.61) | -0.92 (-1.19--0.64) | 1.29 (0.81-1.78) | 3.96 (3.34-4.58) | -0.45 (-0.93-0.04) |
| Lithuania | 0.7 (0.57-0.82) | -1.49 (-1.85--1.12) | -1.83 (-2.4--1.25) | 0.17 (0.06-0.28) | -1.61 (-2.23--0.99) | 1.75 (1.49-2.02) | 0.93 (0.77-1.08) | 2.92 (2.59-3.26) | 1.58 (1.48-1.69) |
| Luxembourg | 0.45 (0.35-0.56) | -0.5 (-1.14-0.15) | -4.32 (-4.44--4.21) | -0.47 (-0.57--0.36) | -6.22 (-6.39--6.05) | 0.78 (0.53-1.03) | -0.2 (-0.28--0.11) | 0.46 (0.33-0.59) | 1.06 (0.89-1.24) |
| Madagascar | 0.76 (0.68-0.83) | -1.89 (-2.17--1.61) | -0.15 (-0.3--0.01) | 0.07 (0.03-0.12) | 0 (-0.13-0.13) | -1.87 (-2.27--1.47) | 0.58 (0.5-0.65) | 0.79 (0.74-0.84) | 2.91 (2.6-3.22) |
| Malawi | -0.13 (-0.26--0.01) | -0.38 (-0.46--0.31) | -0.86 (-1.14--0.59) | -0.02 (-0.11-0.07) | -0.84 (-1.1--0.58) | -0.3 (-0.4--0.21) | -0.46 (-0.64--0.29) | 0.5 (0.33-0.66) | 1.14 (1.07-1.2) |
| Malaysia | 0.68 (0.59-0.77) | -1.79 (-2.33--1.26) | 0.98 (0.81-1.15) | 0.17 (0.08-0.25) | 0.42 (0-0.85) | -1.24 (-1.58--0.91) | 0.63 (0.44-0.83) | 3.25 (2.91-3.58) | 1.91 (1.53-2.3) |
| Maldives | 0.06 (-0.11-0.23) | -0.83 (-1.07--0.59) | -4.3 (-4.74--3.87) | -0.72 (-0.9--0.54) | -4.58 (-5.04--4.12) | 0.28 (-0.24-0.81) | 0.09 (-0.1-0.28) | -1 (-1.17--0.82) | 1.69 (1.46-1.92) |
| Mali | 0 (-0.08-0.07) | 1.19 (0.75-1.63) | -0.99 (-1.14--0.83) | -0.5 (-0.59--0.42) | -1.97 (-2.21--1.74) | -0.23 (-0.39--0.06) | -0.21 (-0.25--0.18) | -0.17 (-0.26--0.09) | 0.64 (0.22-1.07) |
| Malta | -0.27 (-0.39--0.14) | -0.77 (-1.02--0.52) | -2.51 (-2.69--2.34) | -0.54 (-0.67--0.41) | -4.21 (-4.41--4.02) | 0.42 (0.32-0.52) | -1.15 (-1.37--0.94) | 0.73 (0.22-1.23) | 1.59 (1.52-1.66) |
| Marshall Islands | 0.97 (0.87-1.07) | 0.9 (0.43-1.37) | 1.29 (1.08-1.49) | 0.68 (0.57-0.79) | 1.3 (1.05-1.54) | -1.37 (-2.14--0.58) | 1.64 (1.43-1.85) | 2.68 (2.23-3.13) | 1.52 (1.26-1.78) |
| Mauritania | 0.43 (0.36-0.49) | -4.97 (-5.5--4.43) | -1.8 (-1.92--1.68) | -0.5 (-0.57--0.43) | -2.46 (-2.68--2.23) | -1.89 (-2.12--1.66) | 0.58 (0.5-0.66) | -0.03 (-0.17-0.12) | 0.69 (0.27-1.11) |
| Mauritius | 1.02 (0.83-1.21) | 0.63 (0.56-0.7) | -2.83 (-3.17--2.48) | -0.26 (-0.4--0.11) | -3.24 (-3.7--2.77) | -0.99 (-1.16--0.83) | 2.47 (2.17-2.77) | 3.3 (3.03-3.56) | -0.72 (-0.97--0.47) |
| Mexico | 0.72 (0.63-0.8) | -0.55 (-0.94--0.16) | 1.08 (0.84-1.33) | -0.04 (-0.18-0.1) | -1.78 (-2.14--1.41) | 0.02 (-0.33-0.37) | 0.76 (0.7-0.81) | 1.3 (1.16-1.43) | 2.99 (2.72-3.25) |
| Micronesia (Federated States of) | 0.84 (0.73-0.96) | -1.08 (-1.46--0.7) | 0.78 (0.68-0.89) | 0.15 (0.11-0.18) | 0.53 (0.46-0.6) | -1.96 (-2.29--1.63) | 1.9 (1.66-2.13) | 2.91 (2.27-3.56) | 0.01 (-0.22-0.24) |
| Monaco | 0.07 (-0.04-0.17) | -0.48 (-0.84--0.12) | -1.55 (-1.8--1.3) | 0.17 (0.08-0.26) | -2.13 (-2.51--1.76) | -1.89 (-2.06--1.72) | -0.58 (-0.74--0.41) | 0.75 (0.57-0.94) | 0.66 (0.36-0.97) |
| Mongolia | 1.48 (1.29-1.67) | -1.9 (-2.2--1.59) | -0.8 (-1.14--0.46) | 0.94 (0.87-1.02) | 2.4 (1.98-2.81) | -1.93 (-2.14--1.73) | 1.87 (1.57-2.17) | 1.52 (1.43-1.62) | 0.2 (0.01-0.4) |
| Montenegro | 0.36 (0.18-0.54) | -0.04 (-0.63-0.55) | -0.89 (-1.34--0.44) | 0.43 (0.36-0.49) | 0.13 (-0.42-0.69) | -0.62 (-0.72--0.52) | 1.01 (0.8-1.21) | 0.67 (0.46-0.87) | 0.31 (0.13-0.48) |
| Morocco | 0.85 (0.75-0.95) | -2.67 (-2.79--2.55) | -0.5 (-0.57--0.42) | 1.29 (1.2-1.37) | 2.51 (2.14-2.89) | -1.44 (-1.57--1.3) | 1.16 (1.11-1.21) | 3.83 (3.56-4.11) | 4.16 (3.33-4.99) |
| Mozambique | -0.1 (-0.24-0.04) | -2.73 (-3.2--2.26) | 1.68 (1.25-2.11) | 0.36 (0.22-0.5) | 1.98 (1.57-2.4) | 1.12 (1.02-1.22) | -0.31 (-0.49--0.13) | 2 (1.72-2.27) | 2.2 (2.12-2.29) |
| Myanmar | 0.61 (0.49-0.74) | -2.83 (-3.17--2.48) | -2.16 (-2.32--2.01) | 0.57 (0.45-0.68) | -0.75 (-0.85--0.66) | 0.22 (0.09-0.35) | 1.09 (1-1.17) | 2.02 (1.89-2.14) | 1.95 (1.81-2.09) |
| Namibia | 0.17 (0.09-0.26) | -3.04 (-3.23--2.86) | -0.33 (-0.84-0.19) | -0.19 (-0.26--0.12) | -1.4 (-1.83--0.98) | -2.05 (-2.26--1.85) | 0.23 (0.14-0.32) | 0.71 (0.45-0.98) | 0.55 (0.33-0.77) |
| Nauru | 0.15 (0.09-0.2) | -1.61 (-2.05--1.16) | 0.23 (-0.16-0.62) | 0 (-0.13-0.13) | 0.22 (-0.21-0.65) | -3.6 (-3.83--3.38) | 0.65 (0.51-0.8) | 0.8 (0.53-1.07) | -0.03 (-0.22-0.15) |
| Nepal | -0.06 (-0.11--0.02) | 0.28 (-0.12-0.68) | -0.61 (-0.81--0.42) | 0.04 (-0.05-0.14) | -1.22 (-1.59--0.85) | -1 (-1.15--0.85) | 0.25 (0.2-0.3) | 2.12 (1.99-2.26) | -0.21 (-0.32--0.11) |
| Netherlands | 0.32 (0.16-0.48) | -0.47 (-0.63--0.32) | -4.33 (-4.71--3.95) | 0.18 (0.04-0.32) | -2.44 (-2.98--1.89) | -0.58 (-0.81--0.36) | -0.31 (-0.39--0.22) | 1.07 (0.83-1.3) | 0.36 (0.14-0.58) |
| New Zealand | 0.14 (0.1-0.17) | 1.32 (1.2-1.44) | -2.99 (-3.1--2.88) | 0.66 (0.51-0.82) | -3.15 (-3.34--2.96) | -0.68 (-0.82--0.54) | -0.37 (-0.48--0.25) | 1.41 (1.3-1.51) | 3.58 (3.33-3.82) |
| Nicaragua | 0.83 (0.79-0.87) | -1.85 (-2.09--1.6) | -0.04 (-0.43-0.36) | 0.01 (-0.07-0.08) | -2.23 (-2.54--1.92) | -0.28 (-0.33--0.22) | 0.65 (0.6-0.69) | 1.44 (1.1-1.79) | -0.15 (-0.2--0.11) |
| Niger | 0.17 (0.08-0.25) | -3.3 (-4.45--2.15) | -0.66 (-0.79--0.54) | -0.26 (-0.35--0.17) | -1.48 (-1.7--1.26) | 0.16 (0.08-0.25) | 0.27 (0.14-0.4) | -0.17 (-0.27--0.06) | -0.26 (-0.35--0.17) |
| Nigeria | 0.29 (0.25-0.33) | -0.77 (-0.92--0.63) | -1.33 (-1.55--1.12) | 0.31 (0.2-0.42) | -1.01 (-1.22--0.8) | -2.03 (-2.27--1.79) | 0.37 (0.23-0.5) | -1.18 (-1.5--0.85) | -0.24 (-0.32--0.17) |
| Niue | 0.78 (0.71-0.85) | -1.21 (-1.32--1.1) | -0.47 (-0.63--0.32) | 0.36 (0.3-0.43) | -0.61 (-0.74--0.48) | 1.85 (1.58-2.11) | 1.19 (1.09-1.29) | 2.05 (1.64-2.46) | 6.58 (4.04-9.19) |
| North Macedonia | 0.35 (0.21-0.48) | -2.29 (-2.58--2) | -1.76 (-2.12--1.39) | 0.03 (-0.09-0.15) | -1.07 (-1.44--0.71) | -0.75 (-1.03--0.47) | 0.81 (0.64-0.99) | 0.31 (-0.07-0.69) | 3 (2.91-3.09) |
| Northern Mariana Islands | 3.22 (2.81-3.62) | 0.65 (0-1.31) | 3.06 (2.54-3.58) | 1.34 (1.18-1.51) | 0.01 (-0.25-0.28) | -1.47 (-1.64--1.29) | 4.9 (4.31-5.49) | 7.81 (5.05-10.64) | 1.55 (1.12-1.99) |
| Norway | -0.06 (-0.12--0.01) | 0.98 (0.81-1.15) | -4.78 (-4.9--4.66) | 0.14 (0.07-0.21) | -5.62 (-5.91--5.33) | -0.85 (-0.98--0.71) | -0.54 (-0.64--0.43) | 0.11 (-0.19-0.41) | 0.35 (0.01-0.69) |
| Oman | 0.27 (0.19-0.35) | 1.53 (1.45-1.61) | -3.88 (-4.52--3.23) | -0.02 (-0.1-0.05) | -2.4 (-3.01--1.8) | -0.08 (-0.22-0.05) | -0.21 (-0.35--0.08) | 2 (1.68-2.32) | 0.38 (0.31-0.45) |
| Pakistan | 0.42 (0.36-0.49) | -1.24 (-1.42--1.06) | 1.07 (0.75-1.4) | 0.3 (0.24-0.35) | 0.48 (0.25-0.72) | -1.77 (-2.1--1.45) | 0 (-0.06-0.05) | 1.98 (1.87-2.09) | 0.91 (0.67-1.14) |
| Palau | 2 (1.89-2.11) | -1.74 (-1.92--1.56) | 1.53 (1.45-1.61) | 1.37 (1.33-1.42) | 1.9 (1.82-1.99) | 1 (0.63-1.37) | 2.99 (2.85-3.12) | 1.96 (1.8-2.12) | 2.74 (2.24-3.24) |
| Palestine | 0.89 (0.83-0.96) | -2.95 (-3.24--2.67) | -1.82 (-2.06--1.57) | 0.23 (0.08-0.38) | -0.28 (-0.93-0.37) | -0.11 (-0.26-0.03) | 0.93 (0.79-1.07) | 0.72 (0.53-0.91) | 0.44 (0.29-0.6) |
| Panama | 1.16 (1.09-1.23) | -1.76 (-2.12--1.39) | -0.55 (-0.94--0.16) | 0.36 (0.28-0.44) | -1.5 (-1.79--1.21) | -0.13 (-0.3-0.04) | 0.94 (0.87-1.01) | 2.17 (2.01-2.34) | 1.12 (0.93-1.32) |
| Papua New Guinea | 0.55 (0.51-0.58) | -0.49 (-1.23-0.26) | 1.11 (0.98-1.25) | 0.45 (0.4-0.5) | 1.09 (0.95-1.23) | -0.69 (-0.86--0.52) | 0.86 (0.79-0.92) | 1.98 (1.75-2.22) | 0.5 (0.15-0.85) |
| Paraguay | 0.43 (0.26-0.6) | -3.07 (-3.44--2.7) | -0.53 (-0.76--0.29) | 0.14 (0.1-0.18) | -1.31 (-1.53--1.08) | -2.94 (-3.06--2.82) | 0.33 (0.22-0.45) | 4.95 (4.41-5.5) | 0.07 (-0.23-0.37) |
| Peru | 0.74 (0.65-0.82) | -3.74 (-4.08--3.39) | -2.13 (-2.51--1.75) | 0.16 (0.08-0.24) | -2.34 (-2.74--1.93) | -0.01 (-0.14-0.12) | 0.99 (0.93-1.05) | 0.92 (0.71-1.12) | 2.67 (2.4-2.94) |
| Philippines | 0.75 (0.57-0.94) | -4.29 (-4.9--3.67) | 5.06 (4.12-6.02) | 0.49 (0.43-0.55) | 5.57 (4.61-6.53) | -1.55 (-1.75--1.36) | 1.06 (0.86-1.26) | 3.94 (3.06-4.83) | -1.08 (-1.35--0.81) |
| Poland | -0.3 (-0.61-0.01) | -1.88 (-2.1--1.65) | -4.9 (-5.1--4.7) | -0.53 (-0.64--0.42) | -3.17 (-3.6--2.73) | 0.25 (-0.09-0.59) | -0.14 (-0.52-0.25) | 1.1 (0.4-1.8) | 1.25 (1.07-1.44) |
| Portugal | 1.12 (0.9-1.34) | 0.19 (-0.02-0.41) | -2.95 (-3.24--2.67) | -0.57 (-0.69--0.45) | -5.85 (-6.2--5.51) | -5.6 (-6.27--4.92) | -0.07 (-0.28-0.15) | 1.2 (1.11-1.29) | 0.99 (0.81-1.17) |
| Puerto Rico | 0.74 (0.66-0.83) | -2.04 (-2.44--1.64) | -2.13 (-2.3--1.96) | 1.05 (0.77-1.34) | -3.02 (-3.38--2.67) | -0.41 (-0.49--0.33) | 0.84 (0.74-0.93) | 1.9 (1.75-2.06) | 0.29 (0.17-0.4) |
| Qatar | -0.41 (-0.82-0) | -1.73 (-1.88--1.57) | -4.44 (-5.07--3.81) | -0.72 (-1.01--0.42) | -4.41 (-5--3.83) | -2.53 (-2.78--2.29) | -0.65 (-1.26--0.04) | -0.22 (-0.99-0.56) | 0.33 (0.14-0.52) |
| Republic of Korea | 1.09 (0.97-1.22) | -1.57 (-2.04--1.09) | -4.44 (-5.07--3.81) | -0.17 (-0.29--0.06) | -5.15 (-5.34--4.96) | -1.56 (-2.02--1.1) | 0.68 (0.51-0.86) | -2.23 (-2.54--1.91) | 0.31 (0.16-0.45) |
| Republic of Moldova | 0.8 (0.64-0.96) | 0.4 (0.03-0.77) | -0.04 (-0.63-0.55) | 0.63 (0.48-0.77) | 1.66 (0.72-2.61) | 2.4 (2.12-2.69) | 1.2 (0.95-1.45) | 3.87 (3.38-4.37) | 1.66 (1.57-1.76) |
| Romania | 0.25 (0.11-0.39) | -2.41 (-2.84--1.98) | -2.04 (-2.44--1.64) | -0.13 (-0.23--0.03) | -2.34 (-2.74--1.93) | -1.29 (-1.48--1.11) | 0.53 (0.37-0.69) | 1.47 (1.27-1.68) | 2.88 (2.58-3.19) |
| Russian Federation | 0.5 (0.31-0.69) | -2.06 (-2.19--1.93) | -1.75 (-2.73--0.77) | -0.13 (-0.33-0.07) | -1.76 (-2.69--0.83) | 1.05 (0.87-1.23) | 0.9 (0.59-1.22) | 1.51 (1.11-1.91) | 2.86 (2.68-3.03) |
| Rwanda | 0.13 (0.07-0.19) | 3.06 (2.54-3.58) | -3.11 (-3.57--2.64) | -0.89 (-1.02--0.75) | -4.28 (-4.79--3.76) | 0.23 (0.19-0.26) | 0.36 (0.28-0.44) | -0.77 (-1.02--0.52) | 2.9 (2.74-3.06) |
| Saint Kitts and Nevis | 2.01 (1.82-2.2) | -3.88 (-4.52--3.23) | -3.07 (-3.44--2.7) | 0.34 (0.26-0.42) | -2.04 (-2.32--1.75) | 0.42 (0.32-0.52) | 2.71 (2.42-3) | 2.69 (2.42-2.96) | 0.87 (0.65-1.08) |
| Saint Lucia | 1.56 (1.51-1.62) | -3.79 (-4.04--3.54) | -1.49 (-1.85--1.12) | 0.33 (0.21-0.44) | -0.98 (-1.43--0.53) | -2.51 (-2.68--2.34) | 1.95 (1.86-2.05) | 2.75 (2.57-2.94) | -0.49 (-0.78--0.2) |
| Saint Vincent and the Grenadines | 1.79 (1.67-1.9) | -0.89 (-1.34--0.44) | -0.31 (-0.48--0.13) | 1.11 (1.05-1.17) | -0.88 (-1.5--0.26) | -0.52 (-0.56--0.47) | 2.39 (2.23-2.54) | 4.03 (3.81-4.26) | -0.17 (-0.46-0.13) |
| Samoa | 1.04 (0.91-1.17) | -2.46 (-2.66--2.25) | 1.09 (0.92-1.27) | 0.59 (0.52-0.67) | 1.12 (0.94-1.3) | 0.58 (0.46-0.71) | 1.89 (1.7-2.08) | 2.53 (1.97-3.09) | 1.13 (0.84-1.43) |
| San Marino | 0.22 (0.12-0.31) | 0.26 (0.11-0.4) | -0.2 (-0.52-0.11) | 0.37 (0.27-0.46) | -1.65 (-2.04--1.25) | -0.99 (-1.15--0.82) | -0.02 (-0.28-0.23) | 2.35 (2.14-2.55) | 0.17 (-0.04-0.38) |
| Sao Tome and Principe | 0.9 (0.74-1.07) | -0.03 (-0.22-0.16) | 0.82 (0.58-1.06) | 0.33 (0.28-0.37) | 0.17 (-0.14-0.48) | -3.59 (-3.83--3.35) | 0.65 (0.4-0.89) | 1.43 (1.15-1.71) | -0.07 (-0.37-0.24) |
| Saudi Arabia | 0.89 (0.77-1.02) | -0.27 (-0.48--0.06) | 0.67 (0.39-0.94) | 1.11 (0.91-1.3) | 1.05 (0.87-1.24) | -1.29 (-1.44--1.14) | 0.53 (0.39-0.68) | -0.6 (-0.93--0.28) | 0.36 (0.22-0.5) |
| Senegal | 0.29 (0.24-0.34) | -2.25 (-2.4--2.09) | -0.47 (-0.64--0.31) | -0.26 (-0.33--0.2) | -1.37 (-1.54--1.21) | 0.46 (0.18-0.73) | 0.2 (0.17-0.24) | 0.74 (0.49-0.99) | 2.84 (2.55-3.12) |
| Serbia | 0.32 (0.15-0.49) | -2.51 (-2.69--2.34) | -2.41 (-2.84--1.98) | -0.19 (-0.32--0.05) | -1.75 (-2.16--1.33) | -1.72 (-1.86--1.59) | 0.47 (0.26-0.68) | 3.35 (3.06-3.64) | 0.74 (0.47-1.02) |
| Seychelles | 1.35 (1.27-1.42) | -4.9 (-5.1--4.7) | -0.77 (-0.92--0.63) | 0.72 (0.66-0.78) | -0.2 (-0.46-0.06) | -0.61 (-0.82--0.4) | 2.41 (2.25-2.58) | 2.56 (2.42-2.7) | 2.23 (2-2.46) |
| Sierra Leone | -0.22 (-0.31--0.13) | -5.07 (-5.35--4.8) | -0.03 (-0.17-0.11) | -0.28 (-0.34--0.21) | -0.55 (-0.62--0.48) | 0.41 (0.19-0.63) | -0.63 (-0.77--0.49) | 0.19 (-0.09-0.47) | 2.98 (2.5-3.47) |
| Singapore | 0.22 (0.16-0.28) | -1.75 (-2.73--0.77) | -2.26 (-2.43--2.08) | -0.49 (-0.58--0.4) | -6.23 (-6.48--5.98) | -0.75 (-0.92--0.58) | -0.49 (-0.64--0.35) | 1.9 (1.76-2.05) | 0.97 (0.57-1.36) |
| Slovakia | -0.44 (-0.6--0.28) | 0.67 (0.39-0.94) | -3.36 (-3.66--3.05) | -0.45 (-0.5--0.41) | -2.34 (-2.82--1.85) | -0.85 (-1.12--0.58) | -0.12 (-0.35-0.12) | 4.69 (4.21-5.16) | 0.38 (-0.04-0.79) |
| Slovenia | -0.12 (-0.22--0.02) | -3.36 (-3.66--3.05) | -3.82 (-4.15--3.48) | -0.62 (-0.78--0.46) | -5.96 (-6.3--5.63) | -2.61 (-2.94--2.29) | 0.59 (0.48-0.71) | 0.45 (0.25-0.64) | 1.37 (1.24-1.49) |
| Solomon Islands | 0.48 (0.35-0.61) | -2.73 (-3.06--2.41) | 0.71 (0.58-0.84) | 0.2 (0.08-0.32) | 0.8 (0.62-0.98) | 0.38 (0.2-0.57) | 0.85 (0.66-1.04) | 2.02 (1.82-2.23) | 1.61 (1.47-1.75) |
| Somalia | -0.67 (-0.78--0.57) | 2.66 (2.24-3.09) | -0.41 (-0.51--0.31) | -0.58 (-0.69--0.47) | -1.54 (-1.69--1.4) | -0.69 (-0.84--0.54) | -1.25 (-1.39--1.11) | -1.99 (-2.25--1.72) | 2.41 (2.16-2.66) |
| South Africa | 0.14 (0.01-0.27) | -2.13 (-2.3--1.96) | -1.79 (-2.33--1.26) | -0.39 (-0.49--0.29) | -1.85 (-2.39--1.3) | -0.35 (-0.44--0.27) | 0.11 (-0.01-0.23) | 0.52 (0.26-0.78) | 0.14 (0.09-0.2) |
| South Sudan | 1.12 (0.93-1.31) | -3.92 (-4.52--3.32) | 0.64 (0.38-0.9) | 0.53 (0.39-0.68) | -0.18 (-0.63-0.28) | 0.26 (0.12-0.4) | 1.4 (1.06-1.75) | 0.29 (-0.24-0.81) | 0.41 (0.26-0.56) |
| Spain | 0.65 (0.46-0.84) | -0.73 (-1.13--0.33) | -2.06 (-2.19--1.93) | -0.06 (-0.18-0.05) | -4.62 (-5.01--4.22) | -0.73 (-0.92--0.53) | -0.17 (-0.42-0.08) | 0.86 (0.65-1.07) | 1.37 (1.1-1.64) |
| Sri Lanka | 0.76 (0.68-0.84) | -4.71 (-4.96--4.46) | -1.9 (-2.2--1.59) | 0.58 (0.49-0.67) | 0.55 (0.3-0.81) | -0.06 (-0.28-0.16) | 1.43 (1.22-1.64) | 2.51 (2.28-2.75) | 0.2 (0.07-0.33) |
| Sudan | 0.37 (0.32-0.43) | -4.44 (-5.07--3.81) | -1.9 (-1.97--1.84) | 1.14 (1.06-1.23) | 1.64 (1.32-1.96) | -0.44 (-0.55--0.34) | 0.36 (0.24-0.47) | 2.18 (1.92-2.43) | 1.46 (1.38-1.53) |
| Suriname | 1.62 (1.54-1.7) | -1.97 (-2.31--1.64) | -0.75 (-1.23--0.26) | 0.79 (0.71-0.86) | 0.28 (-0.06-0.62) | 0.07 (-0.02-0.15) | 1.62 (1.43-1.82) | 2.88 (2.45-3.3) | 1.62 (1.23-2) |
| Sweden | 0.21 (0.13-0.29) | -5.93 (-6.54--5.32) | -3.7 (-3.84--3.55) | 0.99 (0.77-1.2) | -4.41 (-4.64--4.18) | -1.67 (-1.85--1.5) | -1.14 (-1.24--1.05) | -0.25 (-0.47--0.03) | -1.11 (-1.28--0.94) |
| Switzerland | 0.31 (0.27-0.35) | -2.7 (-2.8--2.59) | -3.74 (-3.85--3.62) | -0.21 (-0.31--0.11) | -4.88 (-5.13--4.64) | -0.64 (-0.8--0.49) | -0.32 (-0.36--0.28) | 0.23 (0.1-0.37) | -0.17 (-0.39-0.06) |
| Syrian Arab Republic | 2.1 (1.65-2.54) | -2.99 (-3.1--2.88) | -0.52 (-1.08-0.05) | 0.62 (0.31-0.92) | -0.68 (-1.43-0.07) | 0.38 (0.22-0.53) | 2.07 (1.49-2.66) | 1.77 (1.26-2.29) | 0.34 (0.12-0.57) |
| Taiwan (Province of China) | 1.42 (1.15-1.7) | -3.82 (-4.15--3.48) | 0.95 (0.73-1.17) | 1 (0.87-1.12) | -2.31 (-2.54--2.08) | -0.9 (-1.11--0.69) | 2.61 (2.41-2.81) | 2.68 (2.49-2.86) | 1.67 (1.56-1.78) |
| Tajikistan | 0.88 (0.67-1.09) | -2.7 (-2.92--2.48) | -0.12 (-0.41-0.18) | 0.67 (0.51-0.83) | 0.43 (0.07-0.79) | -3.01 (-3.3--2.73) | 1.27 (0.91-1.62) | 1.55 (1.34-1.76) | 1.09 (1.05-1.14) |
| Thailand | 1.54 (1.47-1.6) | -1.83 (-2.4--1.25) | -1.08 (-1.46--0.7) | 0.74 (0.68-0.8) | 0.7 (0.47-0.92) | -0.62 (-0.95--0.29) | 1.74 (1.66-1.82) | 1.79 (1.68-1.9) | 0.7 (0.33-1.07) |
| Timor-Leste | 0.27 (0.2-0.35) | -3.17 (-3.34--2.99) | 1.41 (1.15-1.66) | 0.49 (0.41-0.57) | 2.01 (1.77-2.26) | -0.79 (-0.97--0.61) | 0.53 (0.35-0.71) | 2.77 (2.61-2.93) | 2.49 (2.18-2.81) |
| Togo | 0.95 (0.9-0.99) | -3.32 (-3.6--3.04) | 0.77 (0.62-0.91) | 0.09 (0.03-0.15) | -0.5 (-0.58--0.43) | -1.59 (-1.84--1.35) | 0.81 (0.74-0.89) | 0.9 (0.72-1.08) | 0.75 (0.69-0.82) |
| Tokelau | 0.92 (0.74-1.1) | -3.4 (-3.64--3.16) | 0.52 (0.32-0.71) | 0.5 (0.42-0.59) | 0.56 (0.34-0.79) | -0.65 (-0.78--0.53) | 1.33 (1.07-1.59) | 2.72 (2.18-3.26) | 1.73 (1.59-1.86) |
| Tonga | 0.65 (0.56-0.74) | -0.69 (-1.07--0.3) | 0.6 (0.54-0.67) | 0.26 (0.14-0.38) | 0.52 (0.36-0.69) | -1.04 (-1.23--0.84) | 1.03 (0.89-1.17) | 2.25 (1.85-2.64) | 1.66 (1.56-1.77) |
| Trinidad and Tobago | 0.82 (0.71-0.94) | -4.42 (-4.7--4.13) | -1.88 (-2.1--1.65) | -0.01 (-0.12-0.09) | -2.47 (-2.77--2.16) | -0.67 (-0.79--0.56) | 1.01 (0.85-1.17) | 2.59 (2.4-2.78) | 0.01 (-0.07-0.09) |
| Tunisia | 1.42 (1.34-1.5) | -1.18 (-1.38--0.98) | -0.38 (-0.46--0.31) | 1.46 (1.37-1.54) | 2.68 (2.29-3.08) | 0.37 (0.15-0.59) | 1.63 (1.53-1.73) | 3.9 (3.66-4.15) | 2.45 (2.24-2.65) |
| Turkey | 0.04 (-0.1-0.19) | -2.26 (-2.43--2.08) | -3.74 (-4.08--3.39) | 0.19 (0.14-0.23) | -0.23 (-0.6-0.15) | -2 (-2.12--1.87) | 0.6 (0.49-0.72) | 0.37 (0.16-0.58) | 0.7 (0.45-0.96) |
| Turkmenistan | 1.53 (1.36-1.7) | -4.13 (-4.35--3.91) | -0.5 (-1.14-0.15) | 1.68 (1.54-1.81) | 2.89 (2.23-3.56) | -0.84 (-1.4--0.27) | 2.01 (1.75-2.28) | 4.38 (4-4.76) | 2.89 (2.48-3.3) |
| Tuvalu | 0.45 (0.24-0.65) | 0.95 (0.73-1.17) | 0.5 (0.33-0.67) | -0.01 (-0.11-0.09) | 0.23 (0.04-0.41) | -0.32 (-0.41--0.22) | 0.71 (0.42-1.01) | 1.86 (1.35-2.38) | 2.25 (2.08-2.43) |
| Uganda | 0.13 (0.06-0.2) | -2.82 (-2.99--2.64) | 0.24 (-0.21-0.7) | -0.16 (-0.23--0.09) | -0.99 (-1.41--0.57) | 0.3 (0.14-0.46) | 0.2 (0.04-0.37) | 0.8 (0.67-0.93) | 3.28 (3.02-3.54) |
| Ukraine | 0.31 (0.15-0.47) | -0.93 (-1.05--0.81) | 0.65 (0-1.31) | 0.33 (0.18-0.47) | -0.52 (-1.06-0.02) | -0.34 (-0.56--0.11) | 0.31 (0.14-0.48) | 2.12 (1.7-2.53) | 2.38 (1.85-2.91) |
| United Arab Emirates | 0.71 (0.01-1.42) | -3.7 (-3.84--3.55) | -0.82 (-1.69-0.05) | 0.77 (0.34-1.21) | 0.63 (-0.18-1.45) | 0.29 (0.09-0.49) | 0.57 (-0.31-1.45) | 3.02 (2.04-4.01) | 2.82 (2.62-3.01) |
| United Kingdom | -0.46 (-0.59--0.34) | -2.15 (-2.28--2.02) | -3.17 (-3.34--2.99) | -0.07 (-0.17-0.02) | -3.44 (-3.63--3.24) | 0.75 (0.39-1.12) | -1.36 (-1.57--1.14) | 0.82 (0.54-1.09) | 2.25 (1.6-2.9) |
| United Republic of Tanzania | 0.89 (0.79-1) | -4.44 (-5.07--3.81) | 0.2 (0.08-0.33) | 0.47 (0.34-0.6) | 0.1 (-0.17-0.37) | -2.79 (-3.02--2.56) | 0.66 (0.5-0.82) | 0.53 (0.2-0.86) | 1.27 (1.17-1.38) |
| United States of America | -0.3 (-0.4--0.19) | -0.82 (-1.69-0.05) | -1.18 (-1.38--0.98) | 1.66 (1.31-2.01) | -1.72 (-1.84--1.59) | -0.97 (-1.06--0.88) | 0.32 (0.15-0.49) | 1.78 (1.42-2.14) | 1.02 (0.79-1.25) |
| United States Virgin Islands | 0.56 (0.45-0.67) | -4.33 (-4.71--3.95) | -0.27 (-0.48--0.06) | 0.53 (0.37-0.69) | 0.29 (0.1-0.49) | -1.23 (-1.63--0.83) | 0.62 (0.48-0.76) | 2.32 (2.09-2.55) | 1.9 (1.46-2.34) |
| Uruguay | -0.15 (-0.17--0.12) | -0.2 (-0.52-0.11) | -2.67 (-2.79--2.55) | -0.56 (-0.62--0.51) | -4.21 (-4.62--3.8) | -2.4 (-2.61--2.19) | -0.72 (-0.79--0.65) | 0.85 (0.63-1.08) | -0.02 (-0.1-0.06) |
| Uzbekistan | 1.45 (1.25-1.65) | -4.9 (-5.08--4.73) | 2.05 (1.53-2.57) | 0.41 (0.32-0.51) | -0.47 (-0.89--0.04) | -0.22 (-0.36--0.08) | 2.18 (1.87-2.5) | 4.16 (3.81-4.52) | 2.7 (2.46-2.93) |
| Vanuatu | 0.29 (0.24-0.33) | -0.79 (-0.96--0.61) | 0.71 (0.58-0.85) | -0.02 (-0.05-0.02) | 0.54 (0.34-0.74) | -0.36 (-0.42--0.3) | 0.96 (0.85-1.07) | 3.27 (3.02-3.52) | 0.98 (0.81-1.15) |
| Venezuela (Bolivarian Republic of) | 0.73 (0.63-0.83) | -4.32 (-4.44--4.21) | -0.38 (-0.55--0.21) | 0.16 (0.03-0.3) | -1.2 (-1.55--0.84) | -0.21 (-0.36--0.07) | 0.61 (0.49-0.73) | 1.44 (1.29-1.58) | 2.87 (2.47-3.28) |
| Viet Nam | 1.63 (1.48-1.79) | -3.01 (-3.22--2.79) | 0.36 (0.13-0.59) | 1.2 (1.07-1.34) | 2.87 (2.47-3.26) | 0.56 (0.43-0.69) | 2.57 (2.35-2.79) | 3.62 (3.45-3.79) | 4.54 (2.66-6.45) |
| Yemen | -0.11 (-0.25-0.04) | -1.55 (-1.8--1.3) | -1.67 (-1.92--1.42) | 0.67 (0.62-0.72) | 1.72 (1.47-1.97) | 0.69 (0.54-0.85) | -0.2 (-0.33--0.06) | 1.64 (1.49-1.79) | 2.66 (2.37-2.95) |
| Zambia | 0.17 (0-0.34) | -4.78 (-4.9--4.66) | -1.12 (-1.43--0.81) | -0.11 (-0.2--0.01) | 0.34 (0.18-0.49) | 0.79 (0.53-1.06) | -0.14 (-0.32-0.03) | 2.16 (1.78-2.54) | 3.52 (2.98-4.06) |
| Zimbabwe | 0.44 (0.37-0.52) | -3.74 (-3.85--3.62) | 2.29 (1.93-2.65) | 0.59 (0.5-0.68) | 2.78 (2.24-3.33) | 2.08 (1.66-2.5) | 0.33 (0.25-0.4) | 0.73 (0.47-0.99) | 0.77 (-0.09-1.64) |

ASCVDs: atherosclerotic cardiovascular disease; IHD: ischemic heart disease; IS: ischemic stroke; PAD: peripheral artery disease; EAPCs: Estimated annual percentage changes; DALYs: disability-adjusted life years.

Table S12 The temporal trends of ASCVDs burden in people older than 55 in 204 countries and territories.

|  | IHD | | | IS | | | PAD | | |
| --- | --- | --- | --- | --- | --- | --- | --- | --- | --- |
| Location | EAPC of Prevalence | EAPC of death | EAPC of DALY | EAPC of Prevalence | EAPC of death | EAPC of DALY | EAPC of Prevalence | EAPC of death | EAPC of DALY |
| Afghanistan | 0.11 (0.06-0.16) | -0.73 (-0.85--0.6) | -1.38 (-1.55--1.21) | 0.9 (0.79-1.01) | 1.53 (1.28-1.78) | 1.04 (0.78-1.3) | 0.76 (0.69-0.83) | 3.08 (2.73-3.43) | 0.61 (0.54-0.67) |
| Albania | 0.11 (0.06-0.16) | 0.51 (0.28-0.75) | -0.32 (-0.5--0.13) | 0.3 (0.14-0.46) | 0.63 (0.26-1) | -0.06 (-0.35-0.23) | 0.3 (0.26-0.34) | 3.4 (3.03-3.76) | 0.44 (0.34-0.53) |
| Algeria | -0.12 (-0.16--0.07) | -1.42 (-1.53--1.31) | -2.01 (-2.14--1.88) | -0.04 (-0.09-0) | -0.25 (-0.41--0.09) | -0.64 (-0.72--0.56) | 0.02 (0-0.04) | 2.84 (2.65-3.04) | 0.46 (0.39-0.53) |
| American Samoa | 0.29 (0.2-0.38) | 0.34 (0.24-0.43) | -0.13 (-0.19--0.08) | -0.02 (-0.1-0.06) | -0.23 (-0.38--0.09) | -0.61 (-0.73--0.48) | 0.48 (0.32-0.64) | 3.14 (1.44-4.86) | 1.11 (0.3-1.92) |
| Andorra | -0.66 (-0.8--0.53) | -0.14 (-0.29-0.01) | -1.55 (-1.72--1.38) | -0.92 (-1.03--0.81) | 0.29 (0.12-0.46) | -1.11 (-1.23--0.99) | -0.8 (-0.88--0.73) | 4.66 (4.01-5.31) | 1.54 (1.34-1.74) |
| Angola | -0.14 (-0.17--0.1) | -0.68 (-0.8--0.56) | -0.9 (-1--0.8) | -0.3 (-0.32--0.27) | 0.02 (-0.08-0.12) | -0.22 (-0.29--0.16) | 0.27 (0.24-0.29) | 1.5 (1.42-1.57) | 0.94 (0.9-0.99) |
| Antigua and Barbuda | -0.74 (-0.78--0.7) | -2.71 (-2.9--2.53) | -1.86 (-2.04--1.68) | -0.98 (-1.07--0.89) | -2.88 (-3.13--2.62) | -1.93 (-2.16--1.7) | -1.02 (-1.06--0.97) | -0.23 (-0.45-0) | 0.47 (0.28-0.67) |
| Argentina | -0.65 (-0.69--0.62) | -1.9 (-2.04--1.75) | -2.38 (-2.57--2.19) | -1.04 (-1.13--0.95) | -1.85 (-2.02--1.68) | -2.29 (-2.49--2.1) | -0.7 (-0.75--0.64) | 1.84 (1.64-2.04) | -0.12 (-0.2--0.04) |
| Armenia | 0.42 (0.19-0.66) | -0.78 (-0.95--0.61) | -1.71 (-1.88--1.54) | -1.18 (-1.41--0.95) | -1.56 (-2.1--1.02) | -2.53 (-2.87--2.19) | 0.48 (0.27-0.7) | 1.26 (0.87-1.65) | -0.07 (-0.2-0.05) |
| Australia | -0.41 (-0.45--0.38) | -3.87 (-4.09--3.64) | -4.71 (-4.95--4.48) | -1.53 (-1.68--1.39) | -2.84 (-3.04--2.64) | -3.43 (-3.6--3.25) | -1.5 (-1.66--1.33) | 1.35 (1.16-1.55) | 0.33 (0.25-0.41) |
| Austria | -0.51 (-0.55--0.46) | -2.15 (-2.31--2) | -3.03 (-3.21--2.86) | -1.05 (-1.16--0.94) | -4.69 (-5.02--4.35) | -4.56 (-4.83--4.29) | -1.03 (-1.15--0.91) | 1.4 (1.29-1.51) | 0.52 (0.43-0.61) |
| Azerbaijan | -0.2 (-0.51-0.11) | -0.13 (-0.55-0.28) | -0.31 (-0.56--0.05) | 0.46 (0.24-0.68) | 1.96 (1.15-2.78) | 1.58 (1.12-2.04) | -0.18 (-0.42-0.05) | 0.65 (0.34-0.96) | -0.37 (-0.44--0.29) |
| Bahamas | -0.11 (-0.15--0.07) | -1.91 (-2.12--1.7) | -1.68 (-1.9--1.47) | -0.62 (-0.71--0.54) | -1.47 (-1.62--1.32) | -1.3 (-1.45--1.15) | -0.4 (-0.41--0.39) | 0.84 (0.65-1.03) | 0.69 (0.57-0.8) |
| Bahrain | -0.84 (-0.97--0.7) | -5.78 (-6.3--5.25) | -5.22 (-5.56--4.87) | -1.83 (-1.95--1.7) | -3.73 (-4.3--3.16) | -2.98 (-3.28--2.67) | -0.76 (-0.93--0.58) | -0.63 (-1.37-0.12) | -0.28 (-0.71-0.15) |
| Bangladesh | 0.32 (0.29-0.35) | 0.57 (0.29-0.86) | 0.52 (0.18-0.86) | 0.46 (0.37-0.56) | 0.31 (-0.06-0.67) | 0.14 (-0.22-0.51) | -0.08 (-0.16-0) | 3.4 (3.15-3.64) | 0.73 (0.63-0.82) |
| Barbados | -0.47 (-0.52--0.42) | -3.22 (-3.51--2.93) | -2.36 (-2.62--2.09) | -0.86 (-0.97--0.74) | -2.88 (-3.15--2.6) | -2.18 (-2.46--1.89) | -0.83 (-0.89--0.76) | -0.18 (-0.37-0.01) | 0.43 (0.37-0.5) |
| Belarus | 0.33 (0.06-0.6) | 0.43 (-0.01-0.86) | 0.03 (-0.37-0.42) | -0.47 (-0.7--0.24) | -0.63 (-1.08--0.19) | -1.28 (-1.65--0.91) | 0.25 (0.13-0.37) | 2.42 (2.28-2.56) | 1.12 (1.08-1.16) |
| Belgium | -0.35 (-0.44--0.27) | -2.74 (-2.87--2.6) | -3.47 (-3.59--3.34) | -1.29 (-1.31--1.26) | -2.52 (-2.69--2.35) | -3.02 (-3.2--2.84) | -0.89 (-0.97--0.81) | 1.46 (1.23-1.69) | -0.11 (-0.2--0.02) |
| Belize | -0.1 (-0.19-0) | -2.78 (-3.27--2.28) | -2.21 (-2.66--1.75) | -0.48 (-0.6--0.36) | -1.77 (-2.16--1.39) | -1.26 (-1.58--0.94) | -0.55 (-0.65--0.45) | 0.83 (0.51-1.15) | 0.49 (0.34-0.63) |
| Benin | 0 (-0.03-0.03) | -0.61 (-0.66--0.55) | -0.34 (-0.41--0.27) | -0.45 (-0.49--0.41) | -1.05 (-1.14--0.97) | -0.89 (-0.99--0.79) | -0.1 (-0.11--0.08) | 1.09 (0.96-1.22) | 0.39 (0.36-0.42) |
| Bermuda | -0.05 (-0.07--0.02) | -3.48 (-3.88--3.07) | -4.26 (-4.66--3.86) | -0.93 (-1.04--0.82) | -2.1 (-2.29--1.91) | -2.63 (-2.82--2.44) | 0.05 (0.01-0.09) | 0.82 (0.68-0.95) | -0.06 (-0.17-0.06) |
| Bhutan | 0.55 (0.47-0.63) | 1.07 (0.97-1.18) | 0.01 (-0.04-0.05) | -0.09 (-0.12--0.06) | 0.72 (0.69-0.76) | -0.22 (-0.25--0.19) | 0.31 (0.27-0.35) | 4.53 (4.31-4.75) | 1.12 (1.08-1.16) |
| Bolivia (Plurinational State of) | 0.19 (0.18-0.2) | -0.89 (-1.12--0.66) | -1.24 (-1.48--0.99) | -0.68 (-0.81--0.55) | -0.75 (-0.99--0.52) | -1.09 (-1.35--0.84) | -0.19 (-0.22--0.16) | 1.8 (1.72-1.88) | -0.17 (-0.24--0.1) |
| Bosnia and Herzegovina | 0.44 (0.28-0.6) | -0.79 (-0.97--0.61) | -2.08 (-2.3--1.86) | 0.83 (0.65-1.01) | 1.01 (0.72-1.31) | -0.37 (-0.55--0.2) | 1.06 (0.98-1.14) | 3.49 (3.18-3.8) | 0.99 (0.93-1.06) |
| Botswana | 0.05 (-0.02-0.12) | -0.18 (-0.51-0.16) | -0.1 (-0.42-0.22) | -0.25 (-0.3--0.21) | -0.63 (-0.94--0.32) | -0.61 (-0.86--0.37) | -0.11 (-0.21--0.02) | 0.77 (0.45-1.09) | 0.48 (0.24-0.71) |
| Brazil | 0.18 (0.14-0.21) | -1.97 (-2.03--1.91) | -2.29 (-2.37--2.21) | -1.57 (-1.67--1.47) | -2.22 (-2.28--2.15) | -2.81 (-2.93--2.69) | -0.75 (-0.84--0.65) | 1.2 (1.16-1.25) | 0.07 (0.02-0.12) |
| Brunei Darussalam | -0.87 (-0.96--0.77) | -2.35 (-2.52--2.18) | -2.03 (-2.19--1.87) | -2.25 (-2.42--2.07) | -3.67 (-3.9--3.45) | -3.12 (-3.29--2.94) | -1.82 (-1.92--1.71) | -0.18 (-0.33--0.02) | -0.69 (-0.8--0.59) |
| Bulgaria | -0.11 (-0.25-0.03) | -1.37 (-1.8--0.94) | -2.24 (-2.62--1.86) | 0.32 (0.27-0.36) | 1.18 (1.01-1.34) | 0.08 (-0.05-0.22) | 0.37 (0.33-0.41) | 1.29 (1.11-1.48) | 0.08 (0-0.17) |
| Burkina Faso | 0.02 (-0.15-0.19) | 1.02 (0.76-1.29) | 0.96 (0.77-1.16) | -0.22 (-0.25--0.19) | 0.52 (0.29-0.75) | 0.22 (0.12-0.31) | 0.04 (-0.01-0.1) | 3.05 (2.83-3.27) | 1.43 (1.33-1.53) |
| Burundi | -0.28 (-0.35--0.2) | -1.23 (-1.34--1.12) | -0.74 (-0.81--0.67) | -1.51 (-1.6--1.43) | -1.53 (-1.65--1.42) | -1.25 (-1.32--1.17) | -0.56 (-0.61--0.5) | -0.94 (-1.06--0.83) | -0.51 (-0.65--0.37) |
| Cabo Verde | 0.17 (-0.09-0.43) | -0.3 (-0.5--0.09) | -0.35 (-0.72-0.02) | 0.08 (-0.08-0.24) | 1.32 (0.88-1.76) | 0.94 (0.64-1.24) | 0.03 (-0.22-0.27) | 2.23 (1.77-2.69) | 0.84 (0.69-0.99) |
| Cambodia | -0.16 (-0.25--0.08) | -0.15 (-0.2--0.1) | -0.6 (-0.67--0.52) | -0.06 (-0.1--0.02) | 0.23 (0.14-0.32) | -0.17 (-0.23--0.11) | 0.02 (-0.04-0.09) | 2.03 (2-2.07) | -0.14 (-0.16--0.12) |
| Cameroon | 0.55 (0.42-0.68) | 0.62 (0.39-0.85) | 0.61 (0.37-0.85) | 0.28 (0.15-0.41) | 0.35 (0.17-0.53) | 0.19 (0.01-0.37) | 0.33 (0.25-0.41) | 1.78 (1.68-1.88) | 0.79 (0.74-0.84) |
| Canada | -1.31 (-1.43--1.19) | -3.47 (-3.73--3.21) | -3.83 (-4.07--3.6) | -1.53 (-1.83--1.24) | -2.92 (-3.22--2.62) | -2.94 (-3.19--2.69) | -1.74 (-1.89--1.59) | 0.71 (0.6-0.83) | -0.26 (-0.32--0.2) |
| Central African Republic | -0.09 (-0.15--0.03) | -0.18 (-0.21--0.14) | -0.17 (-0.25--0.09) | -0.15 (-0.17--0.13) | 0.13 (0.07-0.19) | 0.03 (-0.02-0.08) | 0.02 (-0.08-0.12) | 0.04 (-0.06-0.15) | 0.05 (0-0.1) |
| Chad | 0.33 (0.28-0.38) | -0.13 (-0.19--0.07) | 0.31 (0.24-0.39) | -0.2 (-0.23--0.17) | -0.57 (-0.66--0.48) | -0.24 (-0.31--0.17) | -0.15 (-0.16--0.13) | 0.87 (0.8-0.94) | 0.48 (0.45-0.51) |
| Chile | -0.39 (-0.47--0.3) | -2.94 (-3.09--2.78) | -3.05 (-3.21--2.88) | -1.14 (-1.27--1.01) | -1.47 (-1.57--1.37) | -2.15 (-2.27--2.02) | -0.81 (-0.87--0.74) | 2.36 (1.99-2.74) | 0.68 (0.53-0.83) |
| China | 0.42 (0.34-0.5) | 1.75 (1.49-2.01) | 0.84 (0.57-1.1) | 1.46 (1.39-1.54) | 0.5 (0.28-0.72) | 0.09 (-0.04-0.21) | 0.32 (0.2-0.43) | 1.48 (1.42-1.54) | -0.35 (-0.43--0.26) |
| Colombia | -0.06 (-0.08--0.05) | -1.72 (-1.87--1.58) | -2.64 (-2.79--2.49) | -1.85 (-1.99--1.71) | -2.68 (-2.86--2.51) | -3.51 (-3.71--3.31) | -0.29 (-0.36--0.23) | 1.44 (1.22-1.66) | -0.12 (-0.28-0.04) |
| Comoros | -0.14 (-0.18--0.1) | -0.09 (-0.16--0.01) | -0.37 (-0.46--0.28) | -0.84 (-0.89--0.79) | -0.11 (-0.18--0.03) | -0.54 (-0.63--0.45) | 0.07 (0.05-0.1) | 0.78 (0.66-0.89) | 0.22 (0.12-0.32) |
| Congo | 0 (-0.05-0.04) | -0.82 (-0.91--0.73) | -1.09 (-1.2--0.98) | -0.6 (-0.65--0.55) | -0.38 (-0.49--0.26) | -0.7 (-0.78--0.62) | -0.23 (-0.3--0.16) | 0.89 (0.79-0.99) | 0.45 (0.3-0.61) |
| Cook Islands | 0.4 (0.33-0.47) | -0.68 (-0.88--0.47) | -1.39 (-1.59--1.19) | 0.31 (0.26-0.37) | -0.67 (-0.89--0.45) | -1.03 (-1.2--0.86) | 0.77 (0.67-0.87) | 2.06 (1.78-2.35) | 0.7 (0.54-0.86) |
| Costa Rica | -0.11 (-0.16--0.05) | -2.4 (-2.67--2.14) | -2.37 (-2.61--2.12) | -0.76 (-0.89--0.64) | -2.13 (-2.55--1.71) | -2.22 (-2.61--1.83) | -0.52 (-0.57--0.46) | 1.26 (1.12-1.4) | 0.03 (-0.03-0.1) |
| Croatia | -0.05 (-0.17-0.07) | -1 (-1.1--0.9) | -2.23 (-2.31--2.15) | -0.5 (-0.65--0.35) | -1.16 (-1.36--0.96) | -2.21 (-2.39--2.04) | -0.21 (-0.31--0.11) | 1.58 (1.43-1.73) | 0.25 (0.16-0.34) |
| Cte d'Ivoire | -0.34 (-0.41--0.27) | -2.11 (-2.4--1.83) | -2.35 (-2.6--2.09) | -0.39 (-0.53--0.26) | -0.13 (-0.25--0.02) | -0.51 (-0.61--0.41) | -0.28 (-0.35--0.2) | 1.53 (1.38-1.68) | 0.86 (0.72-1) |
| Cuba | -0.19 (-0.34--0.04) | -3.16 (-3.4--2.92) | -3.5 (-3.78--3.23) | -1.44 (-1.58--1.3) | -3.23 (-3.42--3.03) | -3.65 (-3.87--3.43) | -0.89 (-0.96--0.83) | 1.59 (1.44-1.73) | -0.27 (-0.33--0.22) |
| Cyprus | -0.7 (-0.87--0.53) | -2.36 (-2.58--2.14) | -3.18 (-3.29--3.07) | -1.91 (-2.03--1.79) | -4.47 (-4.79--4.16) | -4.69 (-4.92--4.47) | -0.07 (-0.2-0.06) | 0.59 (0.48-0.69) | 0.08 (0-0.16) |
| Czechia | 0.34 (0.24-0.45) | -0.29 (-0.48--0.1) | -0.72 (-0.87--0.56) | -0.42 (-0.53--0.32) | -0.23 (-0.4--0.06) | -0.8 (-0.93--0.66) | 0.29 (0.23-0.36) | 1.21 (1.15-1.27) | 0.34 (0.31-0.37) |
| Democratic People's Republic of Korea | 0.92 (0.81-1.04) | 1.76 (1.55-1.97) | 0.6 (0.42-0.78) | 0.76 (0.64-0.88) | 1.12 (0.95-1.29) | 0.1 (-0.03-0.24) | 0.71 (0.61-0.81) | 1.41 (1.14-1.68) | 0.32 (0.23-0.41) |
| Democratic Republic of the Congo | -0.24 (-0.34--0.14) | -0.26 (-0.47--0.05) | -0.59 (-0.63--0.54) | -0.58 (-0.66--0.5) | 0.2 (-0.08-0.48) | -0.33 (-0.42--0.24) | -0.07 (-0.2-0.06) | -0.44 (-0.62--0.26) | -0.61 (-0.88--0.33) |
| Denmark | -1.34 (-1.51--1.18) | -5.28 (-5.62--4.94) | -5.6 (-5.88--5.31) | -2.17 (-2.25--2.09) | -3.06 (-3.27--2.85) | -3.26 (-3.45--3.07) | -1.57 (-1.68--1.45) | -0.03 (-0.27-0.2) | -0.74 (-0.89--0.6) |
| Djibouti | 0.43 (0.4-0.46) | 0.76 (0.7-0.82) | 0.57 (0.48-0.67) | -0.25 (-0.31--0.19) | 0.6 (0.55-0.64) | 0.24 (0.19-0.28) | 0.2 (0.19-0.21) | 1.33 (1.15-1.51) | 0.79 (0.67-0.91) |
| Dominica | -0.19 (-0.26--0.12) | -2.25 (-2.37--2.13) | -1.76 (-1.96--1.56) | -0.44 (-0.48--0.4) | -0.48 (-0.71--0.24) | -0.16 (-0.27--0.06) | -0.7 (-0.82--0.58) | 0.99 (0.76-1.22) | 0.98 (0.91-1.05) |
| Dominican Republic | 0.34 (0.26-0.42) | 2.05 (1.83-2.27) | 1.73 (1.49-1.97) | 0.65 (0.59-0.72) | 1.97 (1.7-2.23) | 1.45 (1.3-1.6) | 0.01 (-0.08-0.1) | 2.99 (2.8-3.17) | 0.7 (0.6-0.8) |
| Ecuador | 0.22 (0.2-0.25) | -0.62 (-0.87--0.36) | -0.83 (-1.09--0.56) | -0.44 (-0.46--0.42) | -0.84 (-1.06--0.62) | -1.24 (-1.49--0.99) | -0.09 (-0.13--0.06) | 2.74 (2.37-3.11) | -0.02 (-0.14-0.09) |
| Egypt | 0.06 (0.03-0.09) | -0.45 (-0.55--0.35) | -0.16 (-0.27--0.05) | 1.2 (1.11-1.29) | 0.99 (0.72-1.25) | 1.42 (1.19-1.65) | 0.14 (0.13-0.15) | 2.63 (2.37-2.89) | 0.49 (0.44-0.55) |
| El Salvador | 0.64 (0.57-0.72) | -0.02 (-0.14-0.1) | -0.79 (-0.94--0.64) | -0.14 (-0.23--0.06) | -1.15 (-1.46--0.84) | -1.68 (-2.02--1.34) | 0.25 (0.2-0.29) | 1.76 (1.61-1.91) | -0.18 (-0.2--0.15) |
| Equatorial Guinea | 0.16 (0.09-0.24) | -1.65 (-1.88--1.41) | -2.51 (-2.83--2.19) | 0.01 (-0.06-0.07) | -0.26 (-0.36--0.16) | -0.93 (-1.07--0.78) | 0.5 (0.42-0.58) | 3.78 (3.56-3.99) | 2.28 (2.17-2.4) |
| Eritrea | 0.16 (0.11-0.2) | 0.73 (0.65-0.81) | 0.19 (0.13-0.25) | -0.31 (-0.33--0.28) | 0.98 (0.92-1.03) | 0.24 (0.2-0.28) | 0.2 (0.16-0.23) | 1.29 (1.08-1.5) | 0.43 (0.26-0.6) |
| Estonia | 0.69 (0.64-0.74) | -2.83 (-3.12--2.55) | -4.04 (-4.38--3.7) | -2.35 (-2.55--2.14) | -5.66 (-6.24--5.07) | -6.28 (-6.83--5.73) | 0.45 (0.38-0.53) | 4.68 (4.01-5.34) | 0.53 (0.35-0.7) |
| Eswatini | 0.12 (0.09-0.14) | 0.27 (0.06-0.47) | 0.4 (0.05-0.74) | 0.01 (-0.02-0.04) | -0.15 (-0.25--0.05) | 0 (-0.18-0.19) | 0.12 (0.08-0.17) | 1.41 (1.27-1.55) | 0.95 (0.8-1.09) |
| Ethiopia | 0.56 (0.5-0.63) | -0.74 (-0.82--0.65) | -1.79 (-1.91--1.68) | -0.08 (-0.19-0.02) | 0.81 (0.74-0.87) | -0.33 (-0.43--0.22) | 0.39 (0.37-0.41) | 2.19 (2.02-2.36) | 0.84 (0.69-0.99) |
| Fiji | 0.05 (0-0.11) | -0.79 (-0.91--0.67) | -0.81 (-0.91--0.71) | -0.07 (-0.14-0) | -1.08 (-1.36--0.8) | -0.88 (-1.08--0.67) | 0.24 (0.14-0.34) | 1.69 (1.47-1.91) | 0.34 (0.23-0.44) |
| Finland | -1.2 (-1.34--1.06) | -2.2 (-2.37--2.03) | -3.22 (-3.35--3.09) | -1.2 (-1.29--1.1) | -2.46 (-2.72--2.19) | -3.1 (-3.27--2.93) | -1.31 (-1.57--1.04) | 1.93 (1.7-2.15) | 0.16 (0.01-0.32) |
| France | -0.75 (-0.82--0.68) | -2.24 (-2.42--2.07) | -2.98 (-3.1--2.86) | -0.47 (-0.55--0.39) | -2.71 (-2.99--2.44) | -2.87 (-3.06--2.68) | -1.2 (-1.31--1.09) | 0.78 (0.65-0.91) | -0.75 (-0.8--0.7) |
| Gabon | 0.03 (-0.1-0.17) | -0.43 (-0.66--0.2) | -0.51 (-0.6--0.42) | -0.47 (-0.61--0.32) | -0.13 (-0.45-0.19) | -0.3 (-0.46--0.13) | -0.01 (-0.14-0.12) | 1.25 (1.04-1.46) | 0.99 (0.92-1.06) |
| Gambia | 0.59 (0.5-0.69) | 1.07 (0.93-1.21) | 0.54 (0.4-0.68) | 0.08 (0.04-0.12) | 0.96 (0.87-1.05) | 0.21 (0.11-0.32) | 0.42 (0.35-0.5) | 2.35 (2.19-2.51) | 0.75 (0.67-0.84) |
| Georgia | 0.32 (0.13-0.5) | -1.63 (-2.06--1.2) | -2.74 (-3.03--2.44) | 0.12 (0.02-0.22) | 0.99 (0.62-1.37) | -0.04 (-0.34-0.27) | 0.96 (0.85-1.07) | 3.77 (3.41-4.13) | 1.03 (0.87-1.18) |
| Germany | -0.84 (-0.92--0.75) | -2.74 (-3.02--2.46) | -3.68 (-3.99--3.36) | -0.72 (-0.88--0.56) | -3.19 (-3.6--2.78) | -3.49 (-3.88--3.1) | -1.42 (-1.59--1.26) | 1.58 (1.43-1.72) | 0.26 (0.12-0.4) |
| Ghana | 0.37 (0.28-0.47) | 0.62 (0.52-0.72) | 0.31 (0.23-0.39) | 0.42 (0.38-0.46) | 0.8 (0.67-0.93) | 0.46 (0.37-0.55) | 0.26 (0.24-0.28) | -1.01 (-1.53--0.49) | -0.57 (-0.76--0.38) |
| Greece | -0.02 (-0.08-0.04) | -0.55 (-0.68--0.42) | -1.88 (-2--1.76) | -0.96 (-1.05--0.87) | -1.87 (-2.18--1.56) | -2.91 (-3.17--2.65) | -0.58 (-0.68--0.49) | 2.23 (1.93-2.52) | -0.59 (-0.76--0.42) |
| Greenland | -0.51 (-0.57--0.46) | -2.85 (-3.08--2.62) | -3.14 (-3.34--2.94) | -1.2 (-1.39--1.01) | -3.28 (-3.6--2.96) | -3.2 (-3.45--2.95) | -1.38 (-1.56--1.21) | -1.28 (-1.58--0.98) | -1.73 (-1.98--1.48) |
| Grenada | -0.46 (-0.65--0.28) | -3.31 (-3.77--2.85) | -1.85 (-2.18--1.52) | -0.8 (-1.02--0.58) | -3.68 (-4.09--3.26) | -2.41 (-2.7--2.11) | -0.91 (-1.06--0.76) | -0.47 (-0.67--0.28) | 0.62 (0.54-0.71) |
| Guam | 1.02 (0.9-1.14) | 0.52 (0.22-0.83) | -0.37 (-0.68--0.05) | 0.51 (0.45-0.57) | -1.07 (-1.45--0.69) | -1.34 (-1.59--1.08) | 1.05 (0.95-1.14) | 4.54 (2.7-6.4) | 1.61 (0.87-2.35) |
| Guatemala | 1.1 (1.02-1.18) | -0.79 (-1.35--0.21) | -1.93 (-2.47--1.38) | -0.32 (-0.44--0.19) | -0.13 (-0.41-0.15) | -1.28 (-1.57--0.99) | 0.72 (0.64-0.79) | 2.26 (2.02-2.49) | 0.07 (0.02-0.13) |
| Guinea | 0.58 (0.51-0.64) | 0.97 (0.81-1.14) | 0.98 (0.84-1.12) | 0.19 (0.17-0.21) | 0.72 (0.54-0.91) | 0.45 (0.35-0.55) | 0.17 (0.1-0.24) | 1.47 (1.31-1.63) | 0.57 (0.53-0.61) |
| Guinea-Bissau | 0.13 (0.09-0.17) | 0.01 (-0.08-0.1) | 0.13 (0.03-0.23) | -0.21 (-0.22--0.19) | -0.04 (-0.11-0.02) | -0.08 (-0.13--0.02) | -0.03 (-0.04--0.01) | 1.36 (1.26-1.46) | 0.56 (0.51-0.61) |
| Guyana | -0.26 (-0.35--0.17) | -1.3 (-1.44--1.16) | -1.03 (-1.16--0.91) | -0.63 (-0.69--0.56) | -1.68 (-1.86--1.49) | -1.65 (-1.87--1.44) | -0.31 (-0.42--0.2) | 1.1 (1-1.2) | 0.78 (0.72-0.83) |
| Haiti | -0.11 (-0.19--0.03) | -0.62 (-0.7--0.54) | -0.82 (-0.99--0.65) | -0.01 (-0.06-0.04) | -0.08 (-0.15--0.01) | -0.31 (-0.43--0.2) | -0.22 (-0.29--0.16) | 0.44 (0.34-0.53) | 0.08 (-0.03-0.19) |
| Honduras | 0.26 (0.2-0.32) | 0.89 (0.67-1.12) | 0.91 (0.7-1.12) | -0.45 (-0.51--0.38) | 1.21 (0.95-1.48) | 1.22 (1.01-1.43) | -0.29 (-0.32--0.26) | 0.61 (0.36-0.87) | 0.06 (-0.06-0.17) |
| Hungary | -0.72 (-0.79--0.64) | -1.03 (-1.13--0.92) | -1.86 (-1.95--1.76) | -1.51 (-1.63--1.38) | -2.65 (-2.86--2.43) | -3.11 (-3.28--2.95) | -0.14 (-0.2--0.08) | 0.66 (0.55-0.78) | 0.08 (-0.01-0.16) |
| Iceland | -0.71 (-0.8--0.62) | -3.25 (-3.38--3.12) | -3.77 (-3.9--3.65) | -1.53 (-1.59--1.48) | -3.45 (-3.73--3.16) | -3.56 (-3.75--3.36) | -1.7 (-1.84--1.57) | 0.54 (0.37-0.71) | -0.99 (-1.03--0.95) |
| India | 0.68 (0.64-0.73) | 0.16 (0.04-0.28) | -0.67 (-0.81--0.52) | 0.23 (0.13-0.32) | -0.3 (-0.46--0.14) | -0.99 (-1.12--0.86) | 0.41 (0.35-0.48) | 2.99 (2.91-3.07) | 0.6 (0.57-0.63) |
| Indonesia | 0.03 (-0.07-0.14) | 0.79 (0.62-0.95) | 0.59 (0.51-0.68) | 0.62 (0.57-0.68) | 1.54 (1.37-1.71) | 1.24 (1.15-1.33) | 0.44 (0.33-0.55) | 2.11 (1.98-2.24) | 0.55 (0.48-0.62) |
| Iran (Islamic Republic of) | 0.26 (0.07-0.44) | -1.43 (-1.76--1.11) | -2.7 (-2.96--2.44) | -0.37 (-0.45--0.28) | -0.75 (-1.13--0.37) | -1.84 (-2.04--1.64) | 0.68 (0.62-0.74) | 6.23 (5.36-7.11) | 1.56 (1.2-1.92) |
| Iraq | -0.41 (-0.45--0.38) | -1.37 (-1.48--1.26) | -1.08 (-1.2--0.96) | -0.4 (-0.45--0.34) | -0.62 (-0.69--0.54) | -0.29 (-0.35--0.23) | -0.73 (-0.82--0.63) | 0.16 (0.03-0.29) | -0.76 (-0.82--0.7) |
| Ireland | -1.34 (-1.46--1.22) | -4.32 (-4.61--4.03) | -4.9 (-5.16--4.63) | -2.31 (-2.56--2.06) | -3.84 (-4.16--3.52) | -4.37 (-4.68--4.06) | -1.64 (-1.75--1.54) | 0.32 (0.18-0.46) | -0.48 (-0.57--0.39) |
| Israel | -1.03 (-1.17--0.89) | -4.73 (-5.05--4.4) | -5.37 (-5.69--5.04) | -1.46 (-1.71--1.22) | -3.11 (-3.4--2.81) | -3.41 (-3.59--3.24) | -1.17 (-1.29--1.05) | 1.31 (1.11-1.51) | 0.22 (0.15-0.29) |
| Italy | -0.27 (-0.34--0.19) | -1.6 (-1.72--1.48) | -2.9 (-3.06--2.74) | -0.96 (-1.14--0.78) | -2.27 (-2.5--2.05) | -3.35 (-3.63--3.08) | -1.37 (-1.51--1.22) | 1.1 (1-1.2) | -0.55 (-0.66--0.43) |
| Jamaica | 0.21 (0.13-0.28) | -0.81 (-1.08--0.53) | -0.57 (-0.91--0.23) | -0.19 (-0.3--0.07) | -0.93 (-1.16--0.69) | -1.02 (-1.3--0.73) | 0.11 (0.04-0.18) | 1.17 (0.86-1.48) | 1 (0.79-1.22) |
| Japan | 0.15 (0.01-0.3) | -1.24 (-1.51--0.98) | -2.85 (-3.04--2.66) | -0.05 (-0.19-0.08) | -1.92 (-2.26--1.59) | -2.91 (-3.12--2.69) | -0.94 (-1.16--0.71) | 2.55 (2.39-2.71) | -1.09 (-1.29--0.89) |
| Jordan | 0.13 (0.06-0.2) | -2.7 (-2.96--2.44) | -3.16 (-3.48--2.83) | -0.98 (-1.19--0.78) | -2.56 (-2.83--2.3) | -2.77 (-3.09--2.45) | 0.23 (0.16-0.3) | 0.86 (0.68-1.03) | -0.35 (-0.37--0.33) |
| Kazakhstan | -0.53 (-0.78--0.27) | -1.59 (-2.33--0.83) | -1.84 (-2.56--1.1) | -0.88 (-1.1--0.66) | -0.98 (-1.28--0.68) | -1.11 (-1.43--0.78) | -0.19 (-0.28--0.1) | 1.16 (1.03-1.29) | -0.2 (-0.27--0.13) |
| Kenya | -0.2 (-0.32--0.08) | 0.72 (0.49-0.95) | 1.08 (0.83-1.34) | -0.53 (-0.57--0.49) | 0.07 (-0.06-0.21) | 0.18 (0.07-0.29) | -0.15 (-0.21--0.1) | 1.29 (1.25-1.32) | 1.06 (1.02-1.1) |
| Kiribati | -0.11 (-0.2--0.02) | -0.45 (-0.49--0.41) | -0.16 (-0.28--0.04) | -0.35 (-0.4--0.31) | -0.64 (-0.79--0.48) | -0.39 (-0.46--0.32) | 0.79 (0.68-0.9) | -0.32 (-0.79-0.16) | 0.6 (0.48-0.72) |
| Kuwait | 0.14 (-0.16-0.44) | -1.68 (-2.15--1.2) | -2.02 (-2.3--1.74) | -0.25 (-0.65-0.14) | 1.42 (0.33-2.52) | 0.6 (-0.17-1.38) | 0.18 (-0.07-0.43) | 2.69 (2.13-3.25) | 0.13 (0-0.27) |
| Kyrgyzstan | -0.45 (-0.71--0.19) | 0.98 (0.22-1.75) | 0.98 (0.53-1.43) | -1.54 (-1.74--1.35) | -2.47 (-3.23--1.71) | -1.94 (-2.37--1.5) | -0.36 (-0.55--0.16) | 0.78 (0.21-1.36) | 0.31 (0.21-0.42) |
| Lao People's Democratic Republic | -0.22 (-0.25--0.19) | -0.37 (-0.5--0.23) | -0.68 (-0.73--0.63) | -0.38 (-0.43--0.34) | 0.06 (-0.18-0.3) | -0.25 (-0.37--0.13) | -0.08 (-0.14--0.02) | 1.7 (1.53-1.87) | -0.09 (-0.13--0.05) |
| Latvia | 0.53 (0.46-0.59) | -1.07 (-1.28--0.87) | -2.15 (-2.42--1.88) | -0.69 (-0.78--0.61) | -1.17 (-1.35--0.99) | -2.09 (-2.3--1.87) | 0.98 (0.87-1.09) | 5.96 (5.12-6.8) | 2.39 (1.99-2.78) |
| Lebanon | 0.79 (0.71-0.86) | 0.05 (-0.16-0.27) | -1.15 (-1.39--0.91) | 0.91 (0.78-1.04) | 1.08 (0.81-1.35) | 0.21 (0-0.42) | 0.87 (0.79-0.94) | 4.11 (3.83-4.39) | 0.64 (0.51-0.77) |
| Lesotho | 0.19 (0.06-0.33) | 1.4 (1.08-1.72) | 1.83 (1.56-2.1) | 0.42 (0.37-0.48) | 0.99 (0.64-1.34) | 1.24 (1-1.47) | 0.12 (-0.03-0.26) | 2.14 (1.93-2.35) | 1.49 (1.39-1.59) |
| Liberia | 0.08 (0.02-0.14) | -0.51 (-0.65--0.37) | -0.4 (-0.55--0.25) | -0.55 (-0.63--0.47) | -0.55 (-0.72--0.38) | -0.64 (-0.72--0.55) | -0.06 (-0.11-0) | 1.56 (1.28-1.85) | 0.51 (0.36-0.66) |
| Libya | 0.34 (0.23-0.45) | -0.17 (-0.32--0.03) | -0.47 (-0.66--0.28) | 0.96 (0.85-1.08) | 0.68 (0.48-0.88) | 0.62 (0.47-0.77) | 0.24 (0.17-0.31) | 3.32 (2.97-3.68) | 0.49 (0.45-0.54) |
| Lithuania | 0.34 (0.26-0.43) | -0.73 (-0.92--0.54) | -1.7 (-1.9--1.5) | -0.6 (-1.06--0.13) | 0.15 (-0.09-0.38) | -1.02 (-1.28--0.76) | 0.35 (0.28-0.42) | 2.95 (2.69-3.21) | 1.31 (1.15-1.48) |
| Luxembourg | -0.34 (-0.41--0.27) | -3.32 (-3.42--3.21) | -4.04 (-4.15--3.93) | -2.47 (-2.55--2.38) | -4.39 (-4.53--4.26) | -4.82 (-4.98--4.66) | -1.02 (-1.1--0.95) | 1.27 (1.15-1.38) | -0.28 (-0.34--0.23) |
| Madagascar | -0.13 (-0.2--0.06) | -0.44 (-0.64--0.25) | 0.2 (0.05-0.35) | -0.28 (-0.36--0.2) | -0.19 (-0.47-0.09) | 0.26 (0.07-0.46) | -0.3 (-0.36--0.24) | -0.16 (-0.34-0.02) | 0.33 (0.21-0.45) |
| Malawi | -0.01 (-0.14-0.12) | -0.22 (-0.46-0.01) | -0.5 (-0.7--0.31) | -0.19 (-0.29--0.1) | 0.28 (0.04-0.52) | -0.08 (-0.25-0.1) | -0.03 (-0.09-0.02) | 1.57 (1.46-1.68) | 0.81 (0.76-0.85) |
| Malaysia | 0.08 (0.03-0.13) | -1.47 (-1.8--1.15) | -1.24 (-1.53--0.95) | -0.51 (-0.66--0.35) | -1.47 (-1.76--1.17) | -1.18 (-1.39--0.97) | -0.18 (-0.21--0.15) | 1.78 (1.39-2.17) | -0.13 (-0.18--0.07) |
| Maldives | 0.18 (0.04-0.33) | -2.37 (-2.51--2.24) | -4.03 (-4.3--3.76) | -1 (-1.11--0.9) | -0.42 (-0.51--0.33) | -2.02 (-2.22--1.83) | 0.67 (0.45-0.9) | 3.5 (3.33-3.68) | 0.2 (0.16-0.25) |
| Mali | 0.35 (0.32-0.38) | 0 (-0.04-0.03) | -0.26 (-0.36--0.15) | -0.5 (-0.58--0.43) | -0.1 (-0.17--0.04) | -0.56 (-0.65--0.46) | 0.24 (0.2-0.28) | 1.65 (1.52-1.77) | 0.57 (0.54-0.6) |
| Malta | -0.4 (-0.65--0.15) | -2.47 (-2.66--2.29) | -3.21 (-3.31--3.11) | -1.71 (-1.91--1.5) | -2.85 (-3.05--2.65) | -3.55 (-3.7--3.39) | -1 (-1.17--0.82) | 1.07 (0.87-1.26) | -0.17 (-0.28--0.06) |
| Marshall Islands | -0.55 (-0.65--0.45) | -0.57 (-0.65--0.49) | 0.6 (0.39-0.81) | -0.65 (-0.77--0.53) | -1.76 (-1.92--1.6) | -0.49 (-0.55--0.44) | -0.4 (-0.51--0.29) | 0.19 (-0.17-0.55) | 0.37 (0.2-0.54) |
| Mauritania | 0.18 (0.09-0.27) | -1.16 (-1.25--1.07) | -1.32 (-1.47--1.16) | -1.1 (-1.19--1.02) | -1.29 (-1.37--1.22) | -1.53 (-1.68--1.39) | 0.05 (-0.01-0.11) | 1 (0.88-1.13) | 0.29 (0.24-0.35) |
| Mauritius | -0.49 (-0.65--0.33) | -4.19 (-4.69--3.69) | -4.58 (-5.03--4.14) | -2.45 (-2.68--2.22) | -4.16 (-4.75--3.57) | -4.33 (-4.81--3.85) | 0.22 (0.09-0.35) | 2.03 (1.72-2.34) | 0.51 (0.41-0.61) |
| Mexico | -0.17 (-0.21--0.13) | -0.1 (-0.29-0.09) | -0.55 (-0.72--0.37) | -1.19 (-1.34--1.03) | -1.79 (-1.94--1.63) | -2.03 (-2.18--1.88) | -0.51 (-0.55--0.48) | 0.45 (0.36-0.54) | -0.33 (-0.4--0.26) |
| Micronesia (Federated States of) | -0.78 (-0.88--0.68) | -0.25 (-0.32--0.17) | 0.6 (0.46-0.74) | -0.9 (-1.01--0.8) | -1.01 (-1.24--0.78) | -0.36 (-0.41--0.31) | -0.22 (-0.4--0.05) | 1.23 (0.61-1.85) | 0.59 (0.39-0.8) |
| Monaco | -0.85 (-0.94--0.76) | -2.09 (-2.28--1.91) | -2.44 (-2.66--2.23) | -0.65 (-0.72--0.58) | -2.83 (-3.15--2.5) | -2.69 (-2.98--2.4) | -1.46 (-1.58--1.34) | 0.62 (0.38-0.87) | -0.6 (-0.64--0.55) |
| Mongolia | -0.39 (-0.49--0.29) | -2.67 (-3.13--2.22) | -2.14 (-2.59--1.7) | -0.36 (-0.47--0.25) | 0.73 (0.11-1.35) | 0.95 (0.48-1.43) | -0.43 (-0.49--0.38) | -0.62 (-0.81--0.42) | -0.68 (-0.74--0.62) |
| Montenegro | -0.04 (-0.16-0.07) | 0.52 (0.36-0.69) | 0.08 (-0.08-0.24) | 0.61 (0.5-0.72) | 2.17 (1.79-2.54) | 1.6 (1.31-1.89) | 0.3 (0.19-0.4) | 1.16 (1.07-1.25) | 0.13 (0.11-0.16) |
| Morocco | -0.27 (-0.42--0.12) | -0.49 (-0.59--0.39) | -0.68 (-0.84--0.51) | 0.32 (0.23-0.41) | 0.85 (0.63-1.07) | 0.76 (0.65-0.86) | -0.15 (-0.28--0.03) | 3.62 (3.39-3.85) | 0.37 (0.33-0.4) |
| Mozambique | 0.2 (0.15-0.24) | 1.32 (1.15-1.5) | 1.36 (1.13-1.6) | 0.81 (0.75-0.87) | 1.4 (1.26-1.53) | 1.4 (1.25-1.55) | 0.28 (0.24-0.32) | 2.24 (2.11-2.36) | 1.7 (1.63-1.77) |
| Myanmar | -0.25 (-0.29--0.2) | -1.09 (-1.24--0.95) | -1.61 (-1.66--1.55) | 0.04 (-0.02-0.1) | 0.23 (0.07-0.39) | -0.33 (-0.38--0.28) | -0.21 (-0.25--0.16) | 1.73 (1.6-1.87) | -0.3 (-0.33--0.28) |
| Namibia | -0.28 (-0.39--0.18) | -0.25 (-0.5-0) | -0.49 (-0.69--0.28) | -0.93 (-1.01--0.85) | -0.25 (-0.56-0.06) | -0.67 (-0.88--0.45) | -0.25 (-0.34--0.17) | 1.12 (0.93-1.31) | 0.38 (0.25-0.5) |
| Nauru | -0.69 (-0.78--0.6) | -0.54 (-0.9--0.19) | 0.42 (0.02-0.82) | -0.53 (-0.68--0.38) | -1.57 (-1.91--1.24) | -0.39 (-0.64--0.14) | -0.1 (-0.16--0.05) | -0.57 (-1.02--0.12) | 0.19 (-0.04-0.41) |
| Nepal | 0.04 (-0.03-0.12) | 0.83 (0.74-0.92) | 0.22 (0.1-0.34) | -0.18 (-0.27--0.09) | 0.12 (-0.06-0.3) | -0.39 (-0.6--0.18) | 0.11 (0.03-0.19) | 3.41 (3.22-3.6) | 0.61 (0.55-0.68) |
| Netherlands | -0.82 (-0.92--0.72) | -4.28 (-4.61--3.95) | -4.84 (-5.14--4.54) | -1.55 (-1.71--1.38) | -2.91 (-3.27--2.54) | -3.08 (-3.39--2.78) | -1.54 (-1.7--1.38) | 0.56 (0.48-0.64) | -0.11 (-0.17--0.06) |
| New Zealand | -0.93 (-1.01--0.84) | -3.18 (-3.36--2.99) | -4.04 (-4.24--3.83) | -1.4 (-1.46--1.34) | -2.47 (-2.65--2.29) | -3.01 (-3.15--2.87) | -1.76 (-1.98--1.54) | 1.17 (1.08-1.26) | 0.14 (0.09-0.19) |
| Nicaragua | 0.08 (-0.01-0.16) | 0.43 (0.05-0.82) | 0.29 (0.03-0.55) | -1.03 (-1.11--0.95) | -1.43 (-1.91--0.95) | -1.44 (-1.78--1.1) | -0.39 (-0.41--0.37) | 1.54 (1.14-1.94) | -0.34 (-0.4--0.28) |
| Niger | 0.1 (0.06-0.15) | -0.38 (-0.52--0.25) | -0.42 (-0.55--0.28) | -0.52 (-0.63--0.42) | -0.59 (-0.69--0.5) | -0.76 (-0.87--0.65) | 0.16 (0.11-0.22) | 0.96 (0.83-1.09) | 0.35 (0.26-0.44) |
| Nigeria | 0.3 (0.18-0.42) | -0.92 (-1.06--0.78) | -0.93 (-1.07--0.78) | -0.31 (-0.36--0.26) | -1.05 (-1.22--0.87) | -0.98 (-1.13--0.83) | 0.11 (0.08-0.15) | 0.83 (0.72-0.95) | 0.4 (0.3-0.5) |
| Niue | -0.04 (-0.11-0.04) | -0.5 (-0.58--0.42) | -0.22 (-0.31--0.13) | -0.21 (-0.29--0.14) | -1.26 (-1.4--1.12) | -0.79 (-0.83--0.76) | 0.23 (0.13-0.33) | 0.96 (0.78-1.14) | 0.51 (0.4-0.62) |
| North Macedonia | -0.17 (-0.24--0.1) | -0.91 (-1.14--0.68) | -1.51 (-1.72--1.3) | -0.26 (-0.45--0.08) | -0.05 (-0.24-0.15) | -0.51 (-0.7--0.31) | 0.18 (0.08-0.27) | -0.03 (-0.21-0.14) | -0.37 (-0.42--0.33) |
| Northern Mariana Islands | -0.2 (-0.38--0.02) | 0.88 (0.77-1) | 1.42 (1.2-1.64) | -1.11 (-1.31--0.9) | -2.63 (-3.07--2.19) | -1.85 (-2.14--1.56) | -0.04 (-0.23-0.15) | 2.92 (1.39-4.46) | 1.78 (0.81-2.75) |
| Norway | -1.16 (-1.29--1.03) | -4.28 (-4.5--4.06) | -4.63 (-4.77--4.5) | -0.16 (-0.31--0.01) | -3.99 (-4.22--3.77) | -3.55 (-3.65--3.44) | -1.58 (-1.65--1.51) | -0.17 (-0.42-0.08) | -0.71 (-0.81--0.61) |
| Oman | 0.05 (-0.14-0.24) | -2.17 (-2.53--1.81) | -2.14 (-2.41--1.87) | -0.57 (-0.75--0.38) | -0.8 (-1.2--0.39) | -0.78 (-1.07--0.48) | 0.02 (-0.13-0.17) | 2.56 (2.12-2.99) | 0.95 (0.79-1.11) |
| Pakistan | -0.01 (-0.02-0.01) | 0.28 (0.07-0.49) | 0.82 (0.53-1.11) | 0.03 (0.01-0.05) | -0.74 (-0.86--0.62) | -0.12 (-0.28-0.04) | -0.23 (-0.28--0.19) | 1.14 (1.07-1.21) | 0.45 (0.38-0.53) |
| Palau | -0.55 (-0.65--0.45) | -0.96 (-1.06--0.85) | -0.07 (-0.12--0.01) | -0.43 (-0.53--0.33) | -1.18 (-1.33--1.03) | -0.29 (-0.35--0.24) | -0.18 (-0.28--0.08) | -0.19 (-0.49-0.12) | 0 (-0.07-0.06) |
| Palestine | -0.08 (-0.13--0.02) | -1.83 (-1.96--1.71) | -1.71 (-1.85--1.58) | -0.21 (-0.37--0.06) | -0.61 (-1.15--0.06) | -0.42 (-0.95-0.11) | -0.15 (-0.19--0.11) | 0.66 (0.54-0.79) | -0.17 (-0.2--0.14) |
| Panama | 0.3 (0.27-0.32) | -1.84 (-2.06--1.63) | -2.02 (-2.25--1.78) | -0.61 (-0.71--0.51) | -1.59 (-1.78--1.4) | -1.94 (-2.09--1.8) | -0.17 (-0.19--0.15) | 0.88 (0.64-1.11) | 0.34 (0.21-0.47) |
| Papua New Guinea | 0.04 (-0.09-0.16) | 0.9 (0.68-1.12) | 1.08 (0.93-1.23) | -0.12 (-0.19--0.05) | 0.33 (0.04-0.62) | 0.29 (0.2-0.39) | 0.17 (0.04-0.31) | 1.61 (1.2-2.02) | 0.47 (0.34-0.59) |
| Paraguay | 0.02 (-0.05-0.09) | -0.55 (-0.79--0.32) | -0.41 (-0.68--0.14) | -0.54 (-0.6--0.48) | -1.17 (-1.33--1.01) | -1.18 (-1.33--1.04) | -0.27 (-0.33--0.21) | 4.52 (4.06-4.98) | 1.52 (1.35-1.7) |
| Peru | 0.21 (0.16-0.26) | -2.03 (-2.36--1.69) | -2.71 (-3.08--2.35) | -0.52 (-0.58--0.46) | -1.74 (-2.05--1.44) | -2.29 (-2.61--1.98) | 0.28 (0.24-0.31) | 0.67 (0.38-0.95) | -0.33 (-0.44--0.22) |
| Philippines | -0.14 (-0.32-0.04) | 2.16 (1.62-2.7) | 2.55 (1.97-3.13) | 0.04 (0-0.07) | 1.4 (0.85-1.95) | 1.53 (1.11-1.96) | 0.09 (-0.1-0.28) | 1.88 (1.46-2.31) | 0.39 (0.18-0.61) |
| Poland | -1.16 (-1.33--0.98) | -2.82 (-3.03--2.61) | -3.5 (-3.74--3.27) | -0.9 (-0.98--0.81) | -1.78 (-2.09--1.47) | -2.24 (-2.45--2.03) | -0.5 (-0.63--0.36) | 2.28 (2-2.56) | 0.78 (0.64-0.93) |
| Portugal | -0.07 (-0.22-0.08) | -2.66 (-2.86--2.45) | -3.82 (-4.05--3.6) | -2.65 (-2.79--2.51) | -3.75 (-4.03--3.47) | -4.81 (-5.09--4.53) | -0.63 (-0.72--0.53) | 2.02 (1.93-2.1) | 0.3 (0.24-0.36) |
| Puerto Rico | 0.2 (0.17-0.23) | -3.01 (-3.24--2.78) | -3.56 (-3.74--3.38) | 0.35 (0.07-0.63) | -2.17 (-2.43--1.9) | -2.39 (-2.61--2.18) | 0.21 (0.18-0.23) | 1.88 (1.79-1.98) | 0.84 (0.75-0.92) |
| Qatar | -0.72 (-0.83--0.61) | -4.97 (-5.47--4.48) | -4.38 (-4.84--3.92) | -2 (-2.39--1.6) | -4.55 (-5--4.1) | -3.27 (-3.67--2.88) | -1.26 (-1.37--1.15) | -1.36 (-1.96--0.75) | -1.62 (-1.92--1.32) |
| Republic of Korea | -0.38 (-0.47--0.28) | -3.57 (-4.01--3.13) | -4.87 (-5.31--4.43) | -2.49 (-2.66--2.31) | -4.18 (-4.45--3.9) | -5.01 (-5.27--4.75) | -1.1 (-1.18--1.01) | -1.54 (-2.04--1.04) | -2.49 (-2.71--2.26) |
| Republic of Moldova | 0.26 (0.13-0.38) | -1.05 (-1.46--0.65) | -1.63 (-1.97--1.28) | 0.05 (-0.08-0.18) | 0.27 (-0.19-0.73) | -0.08 (-0.46-0.31) | 0.65 (0.58-0.73) | 3.57 (3.14-4) | 1.53 (1.23-1.83) |
| Romania | -0.13 (-0.25--0.02) | -1.11 (-1.31--0.9) | -2.16 (-2.38--1.94) | -0.74 (-0.79--0.68) | -0.5 (-0.74--0.26) | -1.63 (-1.88--1.39) | 0.46 (0.39-0.54) | 1.84 (1.73-1.94) | 0.59 (0.51-0.67) |
| Russian Federation | 0.2 (0.01-0.38) | -0.73 (-1.23--0.23) | -1.24 (-1.7--0.77) | -1.07 (-1.18--0.97) | -1.76 (-2.34--1.17) | -2.27 (-2.79--1.76) | 0.27 (0.17-0.37) | 1.39 (1.29-1.49) | 0.77 (0.7-0.84) |
| Rwanda | -0.41 (-0.5--0.33) | -1.91 (-2.17--1.65) | -1.97 (-2.23--1.7) | -1.75 (-1.9--1.6) | -1.83 (-2.1--1.57) | -1.96 (-2.2--1.73) | -0.27 (-0.35--0.18) | -0.22 (-0.56-0.13) | -0.35 (-0.61--0.1) |
| Saint Kitts and Nevis | -0.98 (-1.17--0.79) | -4.09 (-4.45--3.72) | -2.93 (-3.18--2.67) | -1.96 (-2.1--1.81) | -3.58 (-3.96--3.2) | -2.64 (-2.78--2.5) | -1.36 (-1.61--1.11) | -0.03 (-0.59-0.54) | 0.69 (0.47-0.92) |
| Saint Lucia | -0.11 (-0.16--0.05) | -2.95 (-3.39--2.52) | -2.89 (-3.34--2.44) | -1.22 (-1.39--1.06) | -2.22 (-2.61--1.84) | -2.46 (-2.88--2.04) | -0.33 (-0.37--0.28) | 0.86 (0.72-1) | 0.37 (0.21-0.53) |
| Saint Vincent and the Grenadines | -0.09 (-0.16--0.02) | -1.55 (-1.7--1.39) | -1.44 (-1.73--1.15) | -0.01 (-0.07-0.06) | -1.61 (-1.92--1.3) | -1.58 (-2--1.16) | -0.34 (-0.45--0.24) | 1.2 (1.03-1.37) | 0.94 (0.78-1.09) |
| Samoa | -0.01 (-0.14-0.11) | 0.14 (0.03-0.25) | -0.1 (-0.16--0.04) | -0.2 (-0.34--0.07) | -0.08 (-0.22-0.06) | -0.37 (-0.39--0.35) | 0.47 (0.29-0.66) | 1.12 (0.8-1.44) | 0.47 (0.33-0.62) |
| San Marino | -0.53 (-0.58--0.49) | -0.65 (-0.71--0.59) | -1.61 (-1.78--1.43) | -0.59 (-0.61--0.56) | -0.11 (-0.22--0.01) | -0.95 (-1.02--0.88) | -1.01 (-1.07--0.95) | 2.97 (2.57-3.38) | 0.39 (0.28-0.5) |
| Sao Tome and Principe | 0.23 (0.02-0.45) | 1 (0.62-1.38) | 0.87 (0.72-1.02) | 0.09 (-0.06-0.25) | 0.52 (0.15-0.88) | 0.31 (0.18-0.44) | 0.06 (-0.12-0.25) | 2.37 (2.13-2.6) | 0.91 (0.85-0.96) |
| Saudi Arabia | -0.04 (-0.13-0.04) | -1.14 (-1.4--0.87) | -0.18 (-0.44-0.09) | 0.05 (-0.13-0.22) | -1.95 (-2.14--1.76) | -0.94 (-1.12--0.76) | -0.49 (-0.56--0.43) | -0.15 (-0.34-0.04) | -0.6 (-0.64--0.56) |
| Senegal | 0 (-0.09-0.09) | -0.22 (-0.32--0.11) | -0.33 (-0.45--0.2) | -0.65 (-0.68--0.62) | -0.27 (-0.34--0.19) | -0.55 (-0.62--0.48) | -0.07 (-0.12--0.03) | 1.28 (1.15-1.42) | 0.38 (0.31-0.46) |
| Serbia | 0.31 (0.19-0.43) | -0.55 (-0.77--0.33) | -1.63 (-1.88--1.37) | -0.2 (-0.38--0.03) | -0.05 (-0.39-0.29) | -1.12 (-1.45--0.79) | 0.69 (0.61-0.77) | 5.75 (5.02-6.48) | 2.4 (1.98-2.83) |
| Seychelles | -0.46 (-0.62--0.31) | -1.73 (-1.99--1.48) | -1.34 (-1.48--1.2) | -0.8 (-0.91--0.69) | -1.63 (-1.86--1.39) | -1.17 (-1.35--0.99) | -0.21 (-0.4--0.01) | 0.16 (-0.15-0.46) | 0 (-0.11-0.11) |
| Sierra Leone | -0.12 (-0.17--0.08) | -0.33 (-0.48--0.18) | 0 (-0.14-0.14) | -0.3 (-0.32--0.28) | -0.51 (-0.63--0.38) | -0.31 (-0.38--0.25) | -0.31 (-0.33--0.3) | 1.02 (0.95-1.09) | 0.23 (0.18-0.28) |
| Singapore | -0.7 (-0.75--0.64) | -3.99 (-4.24--3.75) | -4.43 (-4.58--4.28) | -3.13 (-3.27--2.99) | -5.35 (-5.58--5.13) | -5.29 (-5.44--5.13) | -1.68 (-1.8--1.57) | 1.63 (1.43-1.83) | -0.6 (-0.78--0.42) |
| Slovakia | -1.15 (-1.25--1.04) | -2.06 (-2.32--1.81) | -2.57 (-2.77--2.36) | -1.1 (-1.29--0.91) | -1.6 (-1.83--1.37) | -1.69 (-1.88--1.5) | -0.83 (-0.96--0.7) | 6.16 (5.71-6.61) | 2.98 (2.64-3.32) |
| Slovenia | -0.41 (-0.48--0.34) | -2.65 (-2.88--2.41) | -3.5 (-3.72--3.27) | -1.89 (-2.03--1.75) | -3.04 (-3.41--2.67) | -3.92 (-4.27--3.57) | -0.08 (-0.16-0) | -0.88 (-1.21--0.55) | -1.16 (-1.35--0.97) |
| Solomon Islands | 0.11 (0.03-0.18) | 0.23 (0.18-0.29) | -0.14 (-0.25--0.04) | 0.08 (0.02-0.15) | 1.05 (0.98-1.13) | 0.38 (0.31-0.44) | 0.56 (0.47-0.64) | 1.55 (1.2-1.91) | 0.48 (0.36-0.6) |
| Somalia | 0.13 (0.07-0.2) | 0.45 (0.3-0.61) | 0.45 (0.32-0.58) | -0.43 (-0.54--0.31) | 0.24 (0-0.48) | 0.12 (0.02-0.23) | 0.23 (0.14-0.31) | -0.14 (-0.42-0.14) | -0.07 (-0.17-0.04) |
| South Africa | -0.51 (-0.64--0.39) | -0.05 (-0.56-0.47) | 0.1 (-0.44-0.65) | -0.22 (-0.34--0.1) | 0.44 (-0.14-1.02) | 0.5 (-0.05-1.04) | -0.89 (-0.96--0.81) | 1.36 (1.15-1.58) | 1.14 (0.94-1.33) |
| South Sudan | 0.04 (-0.06-0.14) | -0.39 (-0.47--0.31) | -0.11 (-0.2--0.03) | -0.55 (-0.58--0.53) | -0.58 (-0.66--0.51) | -0.46 (-0.51--0.41) | 0.05 (0-0.1) | -0.01 (-0.09-0.07) | 0.08 (0.03-0.12) |
| Spain | -0.52 (-0.59--0.45) | -2.13 (-2.32--1.95) | -3.15 (-3.31--2.99) | -1.72 (-1.79--1.65) | -3.1 (-3.33--2.86) | -3.87 (-4.11--3.63) | -1.02 (-1.12--0.91) | 1.38 (1.03-1.72) | -0.28 (-0.44--0.13) |
| Sri Lanka | -0.3 (-0.36--0.24) | -1.25 (-1.43--1.08) | -1.53 (-1.73--1.34) | -0.53 (-0.61--0.45) | -1.11 (-1.45--0.78) | -1.17 (-1.42--0.92) | 0.28 (0.19-0.38) | 1.75 (1.53-1.98) | 0.13 (0.01-0.26) |
| Sudan | -0.04 (-0.1-0.02) | -1.12 (-1.2--1.05) | -1.26 (-1.33--1.19) | 0.68 (0.62-0.75) | 0.34 (0.08-0.6) | 0.4 (0.25-0.55) | 0.19 (0.13-0.24) | 3.07 (2.81-3.34) | 0.63 (0.56-0.7) |
| Suriname | 0.4 (0.3-0.5) | -1.92 (-2.33--1.51) | -1.97 (-2.38--1.57) | -0.04 (-0.14-0.05) | -0.59 (-1.04--0.14) | -0.7 (-1.12--0.28) | 0.22 (0.17-0.27) | 1.01 (0.73-1.29) | 0.25 (0.17-0.33) |
| Sweden | -0.65 (-0.74--0.57) | -3.15 (-3.26--3.03) | -3.58 (-3.67--3.48) | -1.09 (-1.19--0.99) | -2.28 (-2.48--2.08) | -2.61 (-2.79--2.43) | -1.34 (-1.49--1.2) | 0.47 (0.38-0.56) | -0.46 (-0.5--0.42) |
| Switzerland | -0.57 (-0.68--0.46) | -2.89 (-3.09--2.69) | -3.64 (-3.79--3.48) | -1.65 (-1.81--1.49) | -3.2 (-3.4--3.01) | -3.44 (-3.65--3.23) | -1.15 (-1.27--1.03) | 0.39 (0.21-0.58) | -0.49 (-0.58--0.4) |
| Syrian Arab Republic | 0.06 (-0.01-0.13) | -0.93 (-1.05--0.8) | -1.03 (-1.23--0.84) | -0.4 (-0.47--0.33) | -1.67 (-1.92--1.42) | -1.51 (-1.79--1.23) | -0.22 (-0.24--0.2) | 0.05 (-0.12-0.22) | -0.65 (-0.72--0.57) |
| Taiwan (Province of China) | 0.15 (-0.08-0.39) | -1.51 (-1.6--1.43) | -2.25 (-2.43--2.07) | -1.12 (-1.36--0.88) | -3.17 (-3.44--2.91) | -3.4 (-3.63--3.17) | 0.27 (0.09-0.45) | 2.2 (2.02-2.39) | -0.13 (-0.2--0.06) |
| Tajikistan | -0.61 (-0.88--0.33) | 0.77 (0.2-1.34) | 1.63 (1.39-1.87) | 0.18 (0.08-0.29) | 0.74 (-0.24-1.73) | 1.8 (1.22-2.37) | -1.21 (-1.4--1.02) | -2.14 (-2.74--1.54) | -0.94 (-1.01--0.87) |
| Thailand | 0.23 (0.18-0.28) | -1.64 (-1.82--1.47) | -2.18 (-2.33--2.03) | -0.68 (-0.77--0.58) | -1.26 (-1.42--1.1) | -1.83 (-1.99--1.67) | -0.17 (-0.26--0.09) | 1.13 (0.92-1.33) | -0.73 (-0.81--0.65) |
| Timor-Leste | 0.53 (0.49-0.58) | 1.91 (1.71-2.11) | 1.11 (0.97-1.25) | 1.13 (1.07-1.19) | 2.23 (2.01-2.46) | 1.5 (1.38-1.63) | 0.63 (0.6-0.67) | 3.12 (2.95-3.29) | 0.4 (0.36-0.44) |
| Togo | 0.03 (0-0.06) | -0.29 (-0.33--0.24) | -0.05 (-0.13-0.03) | -0.44 (-0.48--0.4) | -0.73 (-0.77--0.68) | -0.59 (-0.64--0.53) | -0.01 (-0.03-0.02) | 0.95 (0.83-1.07) | 0.44 (0.39-0.49) |
| Tokelau | -0.19 (-0.34--0.03) | -0.28 (-0.63-0.07) | 0.02 (-0.1-0.14) | -0.39 (-0.55--0.24) | -0.78 (-1.26--0.31) | -0.47 (-0.67--0.27) | 0.01 (-0.2-0.23) | 1.93 (1.33-2.53) | 0.49 (0.3-0.68) |
| Tonga | 0.38 (0.3-0.46) | 0.64 (0.44-0.83) | -0.21 (-0.36--0.06) | 0.17 (0.12-0.21) | 1.28 (1.09-1.46) | 0.26 (0.13-0.39) | 0.76 (0.64-0.89) | 2.26 (1.77-2.74) | 0.68 (0.5-0.86) |
| Trinidad and Tobago | -0.3 (-0.35--0.25) | -2.96 (-3.29--2.62) | -2.83 (-3.13--2.53) | -1.08 (-1.17--0.98) | -2.82 (-3.11--2.54) | -2.78 (-3.04--2.52) | -0.54 (-0.58--0.49) | 0.63 (0.46-0.8) | 0.11 (0.04-0.17) |
| Tunisia | 0.13 (-0.01-0.27) | -0.48 (-0.72--0.23) | -1.02 (-1.11--0.92) | 0.63 (0.43-0.82) | 0.63 (0.22-1.04) | 0.3 (0.08-0.53) | 0.2 (0.1-0.3) | 3.58 (3.2-3.95) | 0.52 (0.48-0.55) |
| Turkey | -0.8 (-0.93--0.66) | -1.99 (-2.22--1.76) | -3.15 (-3.46--2.83) | 0.18 (0.11-0.25) | 1.5 (1-2) | 0.68 (0.28-1.09) | -0.23 (-0.36--0.11) | 0.33 (0.09-0.58) | -0.75 (-0.87--0.63) |
| Turkmenistan | -0.01 (-0.19-0.18) | -1.26 (-1.79--0.73) | -1.33 (-1.78--0.88) | 0.37 (0.28-0.45) | 0.69 (0.19-1.2) | 0.75 (0.36-1.15) | -0.05 (-0.17-0.07) | 2.2 (1.99-2.41) | 0.44 (0.37-0.51) |
| Tuvalu | 0.13 (0.01-0.26) | 0.03 (-0.08-0.15) | -0.22 (-0.29--0.15) | 0.05 (-0.08-0.19) | 0.14 (-0.12-0.4) | -0.21 (-0.29--0.13) | 0.36 (0.21-0.51) | 1.36 (0.92-1.81) | 0.38 (0.24-0.51) |
| Uganda | -0.05 (-0.11-0.02) | -0.06 (-0.3-0.18) | -0.16 (-0.44-0.11) | -0.58 (-0.72--0.45) | -0.16 (-0.38-0.07) | -0.4 (-0.63--0.18) | 0.14 (0.1-0.19) | 1.32 (1.27-1.37) | 0.83 (0.81-0.85) |
| Ukraine | 0.45 (0.24-0.66) | 0.89 (0.51-1.27) | 0.41 (0.01-0.82) | -0.88 (-0.93--0.82) | -1.67 (-1.88--1.46) | -2.03 (-2.26--1.8) | 0.11 (0.07-0.16) | 1.5 (1.35-1.66) | 0.76 (0.63-0.88) |
| United Arab Emirates | -0.5 (-0.61--0.39) | -3.31 (-3.78--2.85) | -1.98 (-2.32--1.64) | -0.74 (-0.85--0.64) | -3.52 (-4.18--2.86) | -1.75 (-2.19--1.3) | -0.92 (-1.02--0.83) | 0.25 (-0.18-0.69) | 0.2 (0.07-0.33) |
| United Kingdom | -1.21 (-1.4--1.01) | -4.02 (-4.28--3.75) | -4.61 (-4.88--4.34) | -1.4 (-1.47--1.34) | -3.34 (-3.54--3.13) | -3.85 (-4.03--3.66) | -1.94 (-2.19--1.68) | 1.43 (1.01-1.85) | -0.23 (-0.4--0.06) |
| United Republic of Tanzania | 0.61 (0.58-0.65) | 0.65 (0.57-0.72) | 0.28 (0.25-0.32) | 0.53 (0.5-0.56) | 1.74 (1.52-1.96) | 1.12 (0.98-1.27) | 0.53 (0.48-0.58) | 1.33 (1.18-1.47) | 0.74 (0.59-0.9) |
| United States of America | -2.37 (-2.57--2.16) | -2.93 (-3.16--2.71) | -2.82 (-2.99--2.65) | -0.37 (-0.47--0.28) | -2.56 (-2.86--2.26) | -1.98 (-2.14--1.83) | -1.05 (-1.23--0.87) | 0.83 (0.58-1.08) | 0.5 (0.41-0.59) |
| United States Virgin Islands | 0.34 (0.23-0.44) | 0.02 (-0.24-0.28) | -0.22 (-0.44-0) | 0.42 (0.32-0.51) | 0.15 (-0.15-0.44) | -0.08 (-0.3-0.13) | 0.22 (0.08-0.36) | 2.13 (1.96-2.3) | 1.52 (1.32-1.72) |
| Uruguay | -0.42 (-0.51--0.34) | -2.29 (-2.41--2.17) | -2.88 (-3.04--2.72) | -1.04 (-1.16--0.92) | -1.48 (-1.72--1.24) | -2.23 (-2.39--2.08) | -0.8 (-0.83--0.77) | 1.79 (1.68-1.91) | -0.93 (-1--0.87) |
| Uzbekistan | -0.14 (-0.45-0.17) | 0.53 (-0.26-1.33) | 1.91 (1.26-2.57) | -0.49 (-0.68--0.3) | -1.36 (-2.26--0.45) | -0.05 (-0.69-0.6) | -0.37 (-0.53--0.21) | 1.08 (0.9-1.26) | 0.48 (0.42-0.54) |
| Vanuatu | -0.05 (-0.1--0.01) | 0.3 (0.17-0.44) | 0.24 (0.11-0.36) | -0.09 (-0.15--0.04) | -0.17 (-0.37-0.03) | -0.19 (-0.32--0.05) | 0.47 (0.44-0.5) | 2.5 (2.36-2.64) | 0.85 (0.78-0.91) |
| Venezuela (Bolivarian Republic of) | -0.43 (-0.49--0.37) | -1.28 (-1.45--1.12) | -1.32 (-1.5--1.14) | -1.04 (-1.19--0.89) | -1.04 (-1.2--0.88) | -1.25 (-1.44--1.07) | -0.98 (-1.07--0.89) | 0.32 (0.08-0.56) | -0.58 (-0.68--0.47) |
| Viet Nam | -0.05 (-0.23-0.12) | -0.41 (-0.51--0.32) | -0.46 (-0.62--0.3) | 0.32 (0.15-0.49) | 0.82 (0.34-1.31) | 0.76 (0.51-1.01) | 0.26 (0.06-0.47) | 1.46 (1.21-1.72) | 0.25 (0.18-0.31) |
| Yemen | 0.13 (0.1-0.16) | -0.71 (-0.8--0.62) | -1.09 (-1.21--0.96) | 0.69 (0.61-0.78) | 0.96 (0.79-1.14) | 0.78 (0.64-0.92) | 0.22 (0.19-0.24) | 3.07 (2.81-3.33) | 0.4 (0.36-0.44) |
| Zambia | -0.03 (-0.1-0.05) | -0.77 (-1.11--0.43) | -1.08 (-1.39--0.76) | 0.03 (-0.01-0.07) | 1.7 (1.63-1.78) | 1.26 (1.18-1.35) | 0.18 (0.11-0.24) | 2.67 (2.44-2.9) | 1.87 (1.66-2.08) |
| Zimbabwe | -0.03 (-0.23-0.17) | 0.98 (0.73-1.24) | 1.24 (1.05-1.43) | 0.49 (0.33-0.65) | 0.86 (0.48-1.23) | 1.01 (0.76-1.27) | 0.24 (0.1-0.38) | 0.11 (-0.26-0.49) | 0.39 (0.22-0.57) |

ASCVDs: atherosclerotic cardiovascular disease; IHD: ischemic heart disease; IS: ischemic stroke; PAD: peripheral artery disease; EAPCs: Estimated annual percentage changes; DALYs: disability-adjusted life years.

Table S13 Ischemic heart disease burden in people under 40 across SDI regions during 1990-2019.

|  |  | Prevalence | | | | | Deaths | | | | | DALY | | | | |
| --- | --- | --- | --- | --- | --- | --- | --- | --- | --- | --- | --- | --- | --- | --- | --- | --- |
|  |  | 1990 | | 2019 | | 1990-2019 | 1990 | | 2019 | | 1990-2019 | 1990 | | 2019 | | 1990-2019 |
| gender | location | Number | Rate | Number | Rate | EAPC | Number | Rate | Number | Rate | EAPC | Number | Rate | Number | Rate | EAPC |
| Female | Global | 1564938.92 (1277514.18-1907200.39) | 80.78 (65.95-98.45) | 2387020.73 (1967077.74-2884606.29) | 98.91 (81.51-119.53) | 0.66 (0.59 - 0.74) | 55039.72 (49051.01-62104.73) | 2.84 (2.53-3.21) | 60738.09 (53005.27-68583.38) | 2.52 (2.2-2.84) | -0.69 (-0.87 - -0.51) | 3239256.2 (2884277.41-3656694.49) | 167.21 (148.89-188.76) | 3543552.08 (3104077.9-4005638.91) | 146.84 (128.63-165.98) | -0.72 (-0.9 - -0.54) |
|  | SDI region |  |  |  |  |  |  |  |  |  |  |  |  |  |  |  |
|  | High SDI | 269905.55 (226654.57-319318.23) | 111.2 (93.38-131.56) | 319753.39 (272229.61-375709.95) | 133.59 (113.73-156.96) | 0.62 (0.51 - 0.73) | 3063.51 (2869.66-3265.43) | 1.26 (1.18-1.35) | 2189.11 (1940.49-2507.19) | 0.91 (0.81-1.05) | -1.14 (-1.25 - -1.02) | 179648.79 (167648.91-191918.81) | 74.02 (69.07-79.07) | 126632.96 (112367.58-144680.91) | 52.9 (46.94-60.44) | -1.14 (-1.25 - -1.02) |
|  | High-middle SDI | 416878.46 (337029.96-512035.98) | 108.26 (87.53-132.97) | 528349.01 (427932.3-647873.75) | 144.05 (116.67-176.63) | 0.93 (0.85 - 1.01) | 9226 (8094.47-10598.19) | 2.4 (2.1-2.75) | 6693.27 (5832.82-7650.39) | 1.82 (1.59-2.09) | -1.54 (-1.88 - -1.2) | 538552.74 (472090.23-618436.09) | 139.86 (122.6-160.61) | 392572.42 (343476.62-446942.78) | 107.03 (93.64-121.85) | -1.5 (-1.83 - -1.16) |
|  | Middle SDI | 524560.72 (420628.91-651954.94) | 80.54 (64.58-100.1) | 821806.15 (668255.51-1008267.61) | 112.94 (91.84-138.57) | 1.11 (0.95 - 1.27) | 21465.48 (18940.15-24257.96) | 3.3 (2.91-3.72) | 21033.34 (18144.53-23990.82) | 2.89 (2.49-3.3) | -0.67 (-0.85 - -0.48) | 1268624.08 (1119849.73-1432188.58) | 194.79 (171.95-219.9) | 1232612.99 (1064473-1405010.32) | 169.4 (146.29-193.09) | -0.69 (-0.88 - -0.51) |
|  | Low-middle SDI | 256890.06 (210332.15-313092.71) | 58.3 (47.74-71.06) | 485853.88 (399223.73-591621.15) | 78.38 (64.4-95.44) | 1.02 (0.96 - 1.08) | 16465.01 (13667.69-20046.66) | 3.74 (3.1-4.55) | 22108.29 (18033.69-26601.22) | 3.57 (2.91-4.29) | -0.37 (-0.5 - -0.23) | 971073.59 (804854.53-1182202.67) | 220.39 (182.67-268.31) | 1284932.32 (1051532.92-1545342.97) | 207.28 (169.63-249.29) | -0.43 (-0.56 - -0.29) |
|  | Low SDI | 95644.7 (79399.93-115168.46) | 44.19 (36.68-53.21) | 229558.51 (190596.24-276792.4) | 50.11 (41.6-60.42) | 0.48 (0.46 - 0.51) | 4789.24 (3727.89-6254.65) | 2.21 (1.72-2.89) | 8671.67 (6936.89-10695.56) | 1.89 (1.51-2.33) | -0.74 (-0.96 - -0.52) | 279584.2 (217175.49-365643.73) | 129.17 (100.34-168.94) | 504352.94 (404314.37-621532.48) | 110.09 (88.25-135.67) | -0.77 (-0.99 - -0.55) |
| Male |  |  |  |  |  |  |  |  |  |  |  |  |  |  |  |  |
|  | Global | 2047033.19 (1670647.45-2498997.97) | 101.83 (83.11-124.31) | 3089297.04 (2543137.53-3747974.61) | 122.87 (101.15-149.06) | 0.59 (0.52 - 0.65) | 110450.31 (102228.69-118995.55) | 5.49 (5.09-5.92) | 151478.55 (136811.4-170274.05) | 6.02 (5.44-6.77) | 0.22 (0.1 - 0.33) | 6304735.85 (5828165.31-6798117.44) | 313.63 (289.92-338.17) | 8610174.31 (7771102.54-9685489.1) | 342.44 (309.07-385.21) | 0.21 (0.1 - 0.33) |
|  | SDI region |  |  |  |  |  |  |  |  |  |  |  |  |  |  |  |
|  | High SDI | 337166.53 (282468.51-405310.68) | 133.25 (111.64-160.19) | 379341.69 (322979.64-446322.94) | 148.64 (126.56-174.89) | 0.26 (0.14 - 0.38) | 10863.39 (10276.14-11357.45) | 4.29 (4.06-4.49) | 7158.7 (6474.26-8205.45) | 2.81 (2.54-3.22) | -1.45 (-1.6 - -1.29) | 610674.46 (574925.24-639728.31) | 241.35 (227.22-252.83) | 402238.13 (364182.04-458995.67) | 157.61 (142.7-179.85) | -1.41 (-1.57 - -1.25) |
|  | High-middle SDI | 517537.15 (415831.44-641894.58) | 128.74 (103.44-159.68) | 634608.67 (508827.4-783625.89) | 160.6 (128.77-198.31) | 0.67 (0.59 - 0.76) | 29489.61 (26478.45-31971.83) | 7.34 (6.59-7.95) | 25959.34 (23313.22-29293.62) | 6.57 (5.9-7.41) | -0.94 (-1.14 - -0.74) | 1661246.33 (1489738.45-1803934.56) | 413.25 (370.59-448.75) | 1464813.99 (1315354.09-1653153.32) | 370.7 (332.88-418.37) | -0.89 (-1.08 - -0.7) |
|  | Middle SDI | 707035.55 (565621.56-879318.94) | 104.49 (83.59-129.95) | 1071091.23 (870897.4-1309912.01) | 140.74 (114.43-172.12) | 1 (0.85 - 1.16) | 37327.9 (33918.85-40997.24) | 5.52 (5.01-6.06) | 54143.44 (48282.11-60935.64) | 7.11 (6.34-8.01) | 0.97 (0.84 - 1.09) | 2158194.75 (1960589.55-2369747.99) | 318.94 (289.74-350.21) | 3090588.19 (2753729.88-3478779.38) | 406.09 (361.83-457.1) | 0.93 (0.82 - 1.05) |
|  | Low-middle SDI | 344120.22 (283741.24-417873.4) | 75.12 (61.94-91.22) | 653676.4 (539788.87-794177.38) | 102.31 (84.48-124.29) | 1 (0.94 - 1.05) | 25326.16 (21964.5-29311.26) | 5.53 (4.79-6.4) | 46325.27 (39608.65-54674.71) | 7.25 (6.2-8.56) | 0.93 (0.7 - 1.15) | 1448541.77 (1255289.68-1678573.25) | 316.19 (274.01-366.41) | 2624767.82 (2246359.36-3097757.65) | 410.8 (351.57-484.82) | 0.88 (0.66 - 1.1) |
|  | Low SDI | 139872.63 (115976.49-168668.98) | 63.76 (52.86-76.88) | 348312.23 (289065.16-419946.11) | 75.32 (62.51-90.81) | 0.59 (0.56 - 0.61) | 7384.56 (6092.35-8945.13) | 3.37 (2.78-4.08) | 17798.86 (14646.38-21434.2) | 3.85 (3.17-4.63) | 0.49 (0.27 - 0.71) | 422739.18 (347925.35-512977.22) | 192.69 (158.59-233.82) | 1022497.05 (841606.65-1231912.44) | 221.1 (181.99-266.38) | 0.48 (0.27 - 0.7) |

EAPCs: Estimated annual percentage changes; SDI: socio-demographic index; DALYs: disability-adjusted life years.

Table S14 Ischemic stroke burden in people under 40 across SDI regions during 1990-2019.

|  |  | Prevalence | | | | | Deaths | | | | | DALY | | | | |
| --- | --- | --- | --- | --- | --- | --- | --- | --- | --- | --- | --- | --- | --- | --- | --- | --- |
|  |  | 1990 | | 2019 | | 1990-2019 | 1990 | | 2019 | | 1990-2019 | 1990 | | 2019 | | 1990-2019 |
| gender | location | Number | Rate | Number | Rate | EAPC | Number | Rate | Number | Rate | EAPC | Number | Rate | Number | Rate | EAPC |
| Female | Global | 4077536.43 (3116586.63-5201738.47) | 210.49 (160.88-268.52) | 5734519.33 (4443751.71-7237224.55) | 237.62 (184.14-299.89) | 0.44 (0.34 - 0.53) | 8923.88 (7102.73-11477.48) | 0.46 (0.37-0.59) | 7858.84 (6605.16-9581.37) | 0.33 (0.27-0.4) | -1.32 (-1.42 - -1.23) | 1246949.26 (939587.39-1634195.1) | 64.37 (48.5-84.36) | 1367191.31 (1043416.15-1757704.26) | 56.65 (43.24-72.83) | -0.49 (-0.56 - -0.42) |
|  | SDI region |  |  |  |  |  |  |  |  |  |  |  |  |  |  |  |
|  | High SDI | 496580.47 (380655.92-629857.64) | 204.59 (156.83-259.51) | 580142.94 (447633.6-733025.31) | 242.37 (187.01-306.24) | 0.8 (0.66 - 0.94) | 671.35 (604.76-751.93) | 0.28 (0.25-0.31) | 398.33 (310.95-520.86) | 0.17 (0.13-0.22) | -1.89 (-2.24 - -1.55) | 116136.11 (89238.9-148577.68) | 47.85 (36.77-61.21) | 109692.1 (78861-146210.17) | 45.83 (32.95-61.08) | -0.01 (-0.16 - 0.13) |
|  | High-middle SDI | 876889.13 (665468.7-1123484.44) | 227.73 (172.82-291.77) | 886039.78 (678307.13-1127088.09) | 241.56 (184.93-307.28) | 0.16 (0.07 - 0.26) | 1748.05 (1476.77-2080.99) | 0.45 (0.38-0.54) | 968.92 (827.61-1129.66) | 0.26 (0.23-0.31) | -2.37 (-2.64 - -2.09) | 264111.02 (201218.63-338687.49) | 68.59 (52.26-87.96) | 212623.38 (156858.88-279992.52) | 57.97 (42.77-76.34) | -0.82 (-0.93 - -0.71) |
|  | Middle SDI | 1425891.45 (1079579.25-1831200.99) | 218.94 (165.76-281.17) | 1765164.44 (1355532.64-2239984.89) | 242.59 (186.29-307.85) | 0.32 (0.22 - 0.42) | 3857.1 (2869.79-5246.73) | 0.59 (0.44-0.81) | 2854.96 (2304.25-3553.34) | 0.39 (0.32-0.49) | -1.45 (-1.55 - -1.36) | 502755.78 (365014.6-678559.5) | 77.19 (56.05-104.19) | 461862.35 (349847.16-595811.75) | 63.47 (48.08-81.88) | -0.7 (-0.78 - -0.62) |
|  | Low-middle SDI | 792679.59 (604186.28-1015601.18) | 179.9 (137.12-230.5) | 1367371.35 (1054481.97-1735121.15) | 220.58 (170.11-279.91) | 0.73 (0.62 - 0.84) | 1733.54 (1242.18-2581.96) | 0.39 (0.28-0.59) | 2046.53 (1570.01-2715.84) | 0.33 (0.25-0.44) | -0.65 (-0.76 - -0.54) | 233794.6 (172039.23-318525.21) | 53.06 (39.05-72.29) | 324338.94 (246100.01-418698.1) | 52.32 (39.7-67.54) | -0.06 (-0.12 - 0.01) |
|  | Low SDI | 483265.85 (384280.24-599313.82) | 223.28 (177.55-276.9) | 1132298.06 (899398.88-1403545.17) | 247.16 (196.32-306.36) | 0.39 (0.31 - 0.47) | 907.2 (631.07-1365.67) | 0.42 (0.29-0.63) | 1581.28 (1164.41-2379.82) | 0.35 (0.25-0.52) | -0.67 (-0.76 - -0.57) | 129397.34 (94271.3-175508.46) | 59.78 (43.56-81.09) | 257622.33 (194647.39-343663.19) | 56.23 (42.49-75.01) | -0.2 (-0.24 - -0.16) |
| Male | Global | 2938235.89 (2219325.39-3781594.61) | 146.16 (110.4-188.11) | 4149602.28 (3169145.18-5288006.67) | 165.04 (126.04-210.32) | 0.49 (0.39 - 0.58) | 10097.58 (7578.05-13839.18) | 0.5 (0.38-0.69) | 10515.21 (8583.18-12917.61) | 0.42 (0.34-0.51) | -0.58 (-0.67 - -0.49) | 1071239.29 (789364.33-1434767.04) | 53.29 (39.27-71.37) | 1178049.98 (934279.92-1467042.15) | 46.85 (37.16-58.35) | -0.37 (-0.45 - -0.29) |
|  | SDI region |  |  |  |  |  |  |  |  |  |  |  |  |  |  |  |
|  | High SDI | 362887.79 (271920.08-467334.55) | 143.42 (107.47-184.7) | 452313.04 (343032.69-577549.93) | 177.23 (134.41-226.31) | 1.12 (0.88 - 1.36) | 811.68 (716.75-940.87) | 0.32 (0.28-0.37) | 509.21 (396.46-672.34) | 0.2 (0.16-0.26) | -1.73 (-2.11 - -1.35) | 96949.61 (78692.48-120134.37) | 38.32 (31.1-47.48) | 86762.32 (64712.35-113776.21) | 34 (25.36-44.58) | -0.2 (-0.39 - 0) |
|  | High-middle SDI | 605167.7 (454117.98-782265.99) | 150.54 (112.97-194.6) | 626859.81 (473947.14-805984.23) | 158.64 (119.94-203.97) | 0.17 (0.07 - 0.26) | 2375.36 (1978.29-2836.6) | 0.59 (0.49-0.71) | 1973.22 (1641.23-2308.97) | 0.5 (0.42-0.58) | -0.97 (-1.17 - -0.76) | 233767.49 (189355.13-287305.43) | 58.15 (47.1-71.47) | 204428.04 (164304.32-251529.55) | 51.73 (41.58-63.65) | -0.62 (-0.73 - -0.51) |
|  | Middle SDI | 1020634.82 (768532.03-1315442.04) | 150.83 (113.58-194.4) | 1316269.64 (1001480.84-1683546.19) | 172.95 (131.59-221.21) | 0.5 (0.41 - 0.6) | 4453.91 (3066.22-6592.73) | 0.66 (0.45-0.97) | 4348.64 (3306.52-5658.37) | 0.57 (0.43-0.74) | -0.21 (-0.36 - -0.07) | 454226.44 (317488.72-639903.39) | 67.13 (46.92-94.57) | 443044.6 (342106.97-565630.72) | 58.21 (44.95-74.32) | -0.28 (-0.42 - -0.14) |
|  | Low-middle SDI | 646074.08 (486954.03-833697.16) | 141.03 (106.29-181.98) | 1048724.1 (800023.15-1338814.96) | 164.13 (125.21-209.53) | 0.58 (0.46 - 0.7) | 1665.91 (1007.36-2728.07) | 0.36 (0.22-0.6) | 2251.37 (1621.42-3105.82) | 0.35 (0.25-0.49) | -0.02 (-0.15 - 0.11) | 193907.47 (128945.31-285658.04) | 42.33 (28.15-62.35) | 266960.76 (199395.25-347739.63) | 41.78 (31.21-54.42) | 0.03 (-0.04 - 0.1) |
|  | Low SDI | 301637.08 (232160.48-382670.2) | 137.49 (105.82-174.43) | 702656.48 (542791.96-884411.52) | 151.94 (117.37-191.24) | 0.37 (0.28 - 0.47) | 783.85 (405.37-1388.59) | 0.36 (0.18-0.63) | 1424.12 (941.1-2173.16) | 0.31 (0.2-0.47) | -0.4 (-0.46 - -0.34) | 91710.79 (55183.07-143861.62) | 41.8 (25.15-65.57) | 175987.85 (126673.99-242484.84) | 38.05 (27.39-52.43) | -0.24 (-0.29 - -0.19) |

EAPCs: Estimated annual percentage changes; SDI: socio-demographic index; DALYs: disability-adjusted life years.

Table S15 Ischemic heart disease burden under 55 for male across SDI regions during 1990-2019.

|  | Prevalence | | | | | Deaths | | | | | DALY | | | | |
| --- | --- | --- | --- | --- | --- | --- | --- | --- | --- | --- | --- | --- | --- | --- | --- |
|  | 1990 | | 2019 | | 1990-2019 | 1990 | | 2019 | | 1990-2019 | 1990 | | 2019 | | 1990-2019 |
| location | Number | Rate | Number | Rate | EAPC | Number | Rate | Number | Rate | EAPC | Number | Rate | Number | Rate | EAPC |
| Global | 10405658.15 (8752086.86-12426574.57) | 436.73 (367.33-521.55) | 19093970.04 (16133154.58-22754489.66) | 593.16 (501.18-706.87) | 1.13  (1.08 -1.18) | 506187.17 (477775.9-535872.56) | 21.24 (20.05-22.49) | 733476.68 (662737.12-811644.09) | 22.79 (20.59-25.21) | 0.3  (0.18 -0.42) | 22597341.67 (21278648.4-23953987.78) | 948.42 (893.07-1005.36) | 32659942.74 (29575510.45-36187227.21) | 1014.59 (918.77-1124.17) | 0.27  (0.15 - 0.39) |
| SDI region |  |  |  |  |  |  |  |  |  |  |  |  |  |  |  |
| High SDI | 2029069.21 (1729810.18-2383420.76) | 617.33 (526.28-725.14) | 2492052.55 (2174647.37-2854654.94) | 690.56 (602.61-791.04) | 0.28  (0.21 -0.34) | 77676.95 (75732.12-79885.47) | 23.63 (23.04-24.3) | 56580.64 (52699.69-61719.61) | 15.68 (14.6-17.1) | -1.47  (-1.56 --1.39) | 3350556.16 (3253547.39-3453661.93) | 1019.38 (989.86-1050.75) | 2435143.16 (2265815.49-2664969.64) | 674.79 (627.87-738.48) | -1.48  (-1.56 - -1.4) |
| High-middle SDI | 2695594.61 (2249856.2-3246472.87) | 548.48 (457.78-660.56) | 4255686.27 (3545784.52-5134568.78) | 769.82 (641.41-928.81) | 1.28  (1.16 - 1.41) | 146385.1 (135658.83-154744.07) | 29.79 (27.6-31.49) | 145073.72 (130533.17-160499.62) | 26.24 (23.61-29.03) | -0.76  (-1.15 - -0.37) | 6448856.1 (5966059.1-6836587.1) | 1312.16 (1213.92-1391.05) | 6398657.61 (5768466.09-7086353.42) | 1157.47 (1043.48-1281.87) | -0.8  (-1.17 - -0.43) |
| Middle SDI | 3220502.54 (2666540.46-3927392.75) | 408.28 (338.05-497.9) | 6826439.62 (5707304.37-8228526.54) | 682.23 (570.39-822.36) | 1.94  (1.86 -2.01) | 138990.43 (127890.27-150783.38) | 17.62 (16.21-19.12) | 254840.37 (226748.8-285297.78) | 25.47 (22.66-28.51) | 1.61  (1.48 - 1.74) | 6385753.66 (5872243.71-6933779.16) | 809.56 (744.46-879.03) | 11404279.43 (10170696.32-12766872.3) | 1139.74 (1016.46-1275.92) | 1.48  (1.36 -1.61) |
| Low-middle SDI | 1775447.56 (1498443.72-2112052.75) | 337.56 (284.89-401.55) | 3825018.21 (3224920.48-4547368.66) | 491.96 (414.78-584.87) | 1.38  (1.29 -1.48) | 105580.53 (93631.89-119220.87) | 20.07 (17.8-22.67) | 201028.28 (172489.1-234158.35) | 25.86 (22.19-30.12) | 1.15  (1.02 -1.29) | 4753857.91 (4206048.88-5382953.46) | 903.83 (799.68-1023.44) | 8994539.49 (7729740.43-10482621.38) | 1156.86 (994.18-1348.25) | 1.1  (0.97 -1.22) |
| Low SDI | 678845.35 (573322.58-808733.53) | 275.45 (232.63-328.15) | 1681818.68 (1420183.74-2000010.94) | 320.13 (270.33-380.7) | 0.51  (0.4- 0.61) | 37270.59 (31651.51-43463.82) | 15.12 (12.84-17.64) | 75463.2 (62931.43-89658.3) | 14.36 (11.98-17.07) | -0.19  (-0.23 - -0.16) | 1645713.41 (1393545.59-1925205.36) | 667.77 (565.45-781.18) | 3405661.75 (2840390.4-4045431) | 648.27 (540.67-770.05) | -0.12  (-0.16 - -0.07) |

EAPCs: Estimated annual percentage changes; SDI: socio-demographic index; DALYs: disability-adjusted life years.

Table S16 Ischemic stroke burden under 55 for male across SDI regions during 1990-2019.

|  | Prevalence | | | | | Deaths | | | | | DALY | | | | |
| --- | --- | --- | --- | --- | --- | --- | --- | --- | --- | --- | --- | --- | --- | --- | --- |
|  | 1990 | | 2019 | | 1990-2019 | 1990 | | 2019 | | 1990-2019 | 1990 | | 2019 | | 1990-2019 |
| location | Number | Rate | Number | Rate | EAPC | Number | Rate | Number | Rate | EAPC | Number | Rate | Number | Rate | EAPC |
| Global | 5593580.53 (4363563.05-7060183.53) | 234.76 (183.14-296.32) | 9402607.6 (7416374.63-11772882.33) | 292.09 (230.39-365.73) | 0.89  (0.82 - 0.97) | 46707.8 (39891.43-56927.36) | 1.96 (1.67-2.39) | 66232.7 (56223.7-77134.93) | 2.06 (1.75-2.4) | 0.25  (0.1 - 0.4) | 2888180.23 (2375865.42-3524593.85) | 121.22 (99.72-147.93) | 4119221.17 (3456104.19-4843264.92) | 127.96 (107.36-150.46) | 0.28  (0.19 - 0.37) |
| SDI region |  |  |  |  |  |  |  |  |  |  |  |  |  |  |  |
| High SDI | 879661.1 (687788.23-1106690.33) | 267.63 (209.25-336.7) | 1223788.37 (965396.15-1536135.01) | 339.12 (267.52-425.67) | 1.29  (1.06 - 1.52) | 4652.12 (4252.8-5150.79) | 1.42 (1.29-1.57) | 3491.95 (2907.3-4229.89) | 0.97 (0.81-1.17) | -1.54  (-1.69 - -1.4) | 316894.82 (272232.94-368882.92) | 96.41 (82.82-112.23) | 306102.39 (243966.39-376535.85) | 84.82 (67.6-104.34) | -0.34  (-0.44 - -0.25) |
| High-middle SDI | 1340352.41 (1038887.1-1706249.65) | 272.72 (211.38-347.17) | 1790946.78 (1400815.59-2269010.31) | 323.97 (253.4-410.45) | 0.68  (0.61 -0.75) | 16427.93 (14564.7-18505.93) | 3.34 (2.96-3.77) | 16982.39 (14298.48-19681.78) | 3.07 (2.59-3.56) | -0.49  (-0.91 --0.07) | 898497.41 (782296.19-1027864.16) | 182.82 (159.17-209.14) | 977152.79 (825225.32-1142455.82) | 176.76 (149.28-206.66) | -0.29  (-0.61 - 0.03) |
| Middle SDI | 1820418.76 (1411862.93-2303463.82) | 230.78 (178.99-292.02) | 3241865.46 (2547173.77-4074265.78) | 323.99 (254.56-407.18) | 1.27  (1.18 -1.35) | 16215.4 (12855.04-21390.41) | 2.06 (1.63-2.71) | 28068.86 (22772.11-33880.09) | 2.81 (2.28-3.39) | 1.45  (1.32 - 1.58) | 1036015.5 (807786.22-1346819.13) | 131.34 (102.41-170.74) | 1661641.72 (1358702.27-1994312.19) | 166.06 (135.79-199.31) | 1.12  (1.01 - 1.22) |
| Low-middle SDI | 1067853.9 (829363.52-1349554.98) | 203.03 (157.68-256.58) | 2006280.99 (1578248.8-2509776.04) | 258.04 (202.99-322.8) | 0.93  (0.8 - 1.05) | 6967.69 (4987.08-10031.11) | 1.32 (0.95-1.91) | 12641.08 (9775.86-16419.01) | 1.63 (1.26-2.11) | 0.97  (0.87 -1.07) | 457219.55 (334881.21-631585.96) | 86.93 (63.67-120.08) | 800973.31 (630352.47-1005996.82) | 103.02 (81.07-129.39) | 0.79  (0.7-0.88) |
| Low SDI | 481884.86 (382170.62-598018.32) | 195.53 (155.07-242.65) | 1133532.16 (902991.73-1396360.71) | 215.77 (171.88-265.8) | 0.37  (0.26 - 0.48) | 2423.34 (1562.46-3876.27) | 0.98 (0.63-1.57) | 5009.34 (3489.01-7559.43) | 0.95 (0.66-1.44) | -0.08  (-0.16 - 0) | 178125.74 (121445.55-265356.87) | 72.28 (49.28-107.67) | 370879.36 (275598.97-513748.6) | 70.6 (52.46-97.79) | -0.04  (-0.12 -0.04) |

EAPCs: Estimated annual percentage changes; SDI: socio-demographic index; DALYs: disability-adjusted life years.

Table S17 Peripheral artery disease burden under 55 for male across SDI regions during 1990-2019.

|  | Prevalence | | | | | Deaths | | | | | DALY | | | | |
| --- | --- | --- | --- | --- | --- | --- | --- | --- | --- | --- | --- | --- | --- | --- | --- |
|  | 1990 | | 2019 | | 1990-2019 | 1990 | | 2019 | | 1990-2019 | 1990 | | 2019 | | 1990-2019 |
| location | Number | Rate | Number | Rate | EAPC | Number | Rate | Number | Rate | EAPC | Number | Rate | Number | Rate | EAPC |
| Global | 3201107.04 (2449509.26-4080079.2) | 134.35 (102.81-171.24) | 5388258.34 (4149658.82-6837852.83) | 167.39 (128.91-212.42) | 1.15  (1.02 - 1.28) | 682.26 (332.7-1343.05) | 0.03 (0.01-0.06) | 1217.55 (718.95-2242.58) | 0.04 (0.02-0.07) | 1.21  (1.06 - 1.35) | 28798.06 (14809.11-55417.14) | 1.21 (0.62-2.33) | 51029.98 (31291.39-91683.68) | 1.59 (0.97-2.85) | 1.15  (1.02 - 1.28) |
| SDI region |  |  |  |  |  |  |  |  |  |  |  |  |  |  |  |
| High SDI | 1110482.26 (852760.81-1413585.53) | 337.86 (259.45-430.07) | 1151293.38 (911120.98-1423883.81) | 319.03 (252.48-394.57) | 1.01  (0.82 - 1.21) | 180.61 (63.59-425.35) | 0.05 (0.02-0.13) | 261.59 (90.69-612.53) | 0.07 (0.03-0.17) | 1.13  (0.93 - 1.33) | 7758.07 (3098.45-17445.1) | 2.36 (0.94-5.31) | 10876.52 (4181.67-24607.7) | 3.01 (1.16-6.82) | 1.01  (0.82 - 1.21) |
| High-middle SDI | 753033.52 (577939.01-960051.65) | 153.22 (117.59-195.34) | 1223638.95 (935246.82-1555890.97) | 221.35 (169.18-281.45) | 1.23  (0.99 - 1.47) | 326.87 (119.85-709.41) | 0.07 (0.02-0.14) | 467.71 (203.22-1019.55) | 0.08 (0.04-0.18) | 1.26  (1 - 1.52) | 13201.15 (5098.77-28251.03) | 2.69 (1.04-5.75) | 19037.74 (8487.95-40928.97) | 3.44 (1.54-7.4) | 1.23  (0.99 - 1.47) |
| Middle SDI | 766520.71 (587043.48-981009.2) | 97.18 (74.42-124.37) | 1772453.45 (1359617.44-2256667.55) | 177.14 (135.88-225.53) | 2.49  (2.39 - 2.59) | 90.6 (66.81-129.58) | 0.01 (0.01-0.02) | 238.58 (184.31-325.77) | 0.02 (0.02-0.03) | 2.62  (2.51 - 2.73) | 4108.18 (3032.05-5775.03) | 0.52 (0.38-0.73) | 10431.33 (8038.01-14069.75) | 1.04 (0.8-1.41) | 2.49  (2.39 - 2.59) |
| Low-middle SDI | 417419.89 (318269.91-534480.43) | 79.36 (60.51-101.62) | 875549.47 (666221.56-1122453.42) | 112.61 (85.69-144.37) | 2.86  (2.71 - 3.02) | 34.63 (25.15-51.88) | 0.01 (0-0.01) | 118.47 (89.16-155.82) | 0.02 (0.01-0.02) | 3.14  (2.98 - 3.3) | 1644.76 (1185.05-2380.35) | 0.31 (0.23-0.45) | 5213.21 (3959.68-6798.58) | 0.67 (0.51-0.87) | 2.86  (2.71 -3.02) |
| Low SDI | 151988.05 (115462.85-195252.95) | 61.67 (46.85-79.23) | 362175.62 (274422.06-466198.87) | 68.94 (52.24-88.74) | 0.58  (0.35 - 0.82) | 49.1 (21.91-80.44) | 0.02 (0.01-0.03) | 130.16 (64.69-205.67) | 0.02 (0.01-0.04) | 0.63  (0.38 - 0.88) | 2066.86 (987.07-3350.58) | 0.84 (0.4-1.36) | 5428.7 (2831.58-8479.34) | 1.03 (0.54-1.61) | 0.58  (0.35 - 0.82) |

EAPCs: Estimated annual percentage changes; SDI: socio-demographic index; DALYs: disability-adjusted life years.

Table S18 Ischemic heart disease burden under 65 for female across SDI regions during 1990-2019.

|  | Prevalence | | | | | Deaths | | | | | DALY | | | | |
| --- | --- | --- | --- | --- | --- | --- | --- | --- | --- | --- | --- | --- | --- | --- | --- |
|  | 1990 | | 2019 | | 1990-2019 | 1990 | | 2019 | | 1990-2019 | 1990 | | 2019 | | 1990-2019 |
| location | Number | Rate | Number | Rate | EAPC | Number | Rate | Number | Rate | EAPC | Number | Rate | Number | Rate | EAPC |
| Global | 14450996.72 (12103056.87-17420436.03) | 584.93 (489.89-705.12) | 28452532.59 (23912036.37-34182752.26) | 822.03 (690.85-987.58) | 1.27  (1.16 - 1.37) | 497492.75 (461976.75-537165.05) | 20.14 (18.7-21.74) | 704082.09 (629132.78-776732.06) | 20.34 (18.18-22.44) | -0.02  (-0.14 - 0.1) | 18318228.94 (16918871.96-19852336.36) | 741.46 (684.82-803.56) | 25821620.78 (23076263.26-28499322.63) | 746.02 (666.7-823.38) | -0.05  (-0.14 - 0.05) |
| SDI region |  |  |  |  |  |  |  |  |  |  |  |  |  |  |  |
| High SDI | 2423316.49 (2066133.72-2856030.68) | 677.8 (577.89-798.83) | 3095488.59 (2692104.58-3581264.51) | 761.75 (662.48-881.29) | 0.44  (0.38 - 0.5) | 68464.88 (66583.55-70283.26) | 19.15 (18.62-19.66) | 47467.5 (44597.33-50744.1) | 11.68 (10.97-12.49) | -1.71  (-1.9 - -1.53) | 2334573.13 (2264047.98-2409088.02) | 652.98 (633.25-673.82) | 1678842.66 (1573858.38-1800814.84) | 413.13 (387.3-443.15) | -1.58  (-1.73 - -1.42) |
| High-middle SDI | 4305584.94 (3565249.68-5276350.94) | 821.43 (680.19-1006.64) | 7297595.33 (6033581.85-8941017.94) | 1195.6 (988.51-1464.84) | 1.43  (1.29 - 1.57) | 129248.75 (122112.26-137044.79) | 24.66 (23.3-26.15) | 126482.71 (113977.24-139932.67) | 20.72 (18.67-22.93) | -0.95  (-1.12 - -0.77) | 4534157.75 (4252036.97-4840012.35) | 865.04 (811.21-923.39) | 4498520.07 (4058042.8-4975755.08) | 737.01 (664.85-815.2) | -0.91  (-1.1 --0.72) |
| Middle SDI | 4324137.53 (3588408.8-5265714.74) | 538.87 (447.19-656.21) | 10340423.14 (8639424.58-12521134.9) | 956.86 (799.45-1158.65) | 2.13  (2.05 - 2.22) | 147507.94 (134268.94-162385.5) | 18.38 (16.73-20.24) | 245966.12 (216176.05-275447.62) | 22.76 (20-25.49) | 0.86  (0.73 - 0.99) | 5641809.17 (5114698.26-6220740.01) | 703.08 (637.39-775.23) | 9031609.88 (7956953.74-10094850.22) | 835.75 (736.3-934.13) | 0.69  (0.6 -0.79) |
| Low-middle SDI | 2501076.88 (2096893.79-3015108.29) | 470.42 (394.39-567.1) | 5662783.47 (4754064.12-6815123.03) | 691.56 (580.58-832.28) | 1.33  (1.19 - 1.47) | 110817.99 (94978.4-128847.02) | 20.84 (17.86-24.23) | 204625.85 (171267.41-238730.15) | 24.99 (20.92-29.15) | 0.65  (0.39 - 0.9) | 4250406.33 (3636168.3-4962756.06) | 799.44 (683.91-933.42) | 7619895.7 (6372641.54-8917000.24) | 930.56 (778.25-1088.97) | 0.53  (0.31 -0.75) |
| Low SDI | 888565.97 (753855.02-1049454.37) | 350.64 (297.48-414.13) | 2039026.35 (1730807.69-2416038.72) | 375.52 (318.76-444.96) | 0.21  (0.1 - 0.32) | 41152.81 (33130.17-50561.56) | 16.24 (13.07-19.95) | 79061.1 (66391.98-93317.54) | 14.56 (12.23-17.19) | -0.51  (-0.57 --0.44) | 1546261.96 (1245705.34-1907755.6) | 610.18 (491.58-752.83) | 2975192.14 (2492820.69-3517263.7) | 547.94 (459.1-647.77) | -0.51  (-0.57 --0.45) |

EAPCs: Estimated annual percentage changes; SDI: socio-demographic index; DALYs: disability-adjusted life years.

Table S19 Ischemic stroke burden under 65 for female across SDI regions during 1990-2019.

|  | Prevalence | | | | | Deaths | | | | | DALY | | | | |
| --- | --- | --- | --- | --- | --- | --- | --- | --- | --- | --- | --- | --- | --- | --- | --- |
|  | 1990 | | 2019 | | 1990-2019 | 1990 | | 2019 | | 1990-2019 | 1990 | | 2019 | | 1990-2019 |
| location | Number | Rate | Number | Rate | EAPC | Number | Rate | Number | Rate | EAPC | Number | Rate | Number | Rate | EAPC |
| Global | 11525192.4 (9307550.6-14132735.34) | 466.5 (376.74-572.05) | 19926407.93 (16273481.53-24204685.92) | 575.7 (470.16-699.3) | 0.82  (0.7 - 0.94) | 107495.06 (96628.83-121615.58) | 4.35 (3.91-4.92) | 131521.74 (116714.4-149496.35) | 3.8 (3.37-4.32) | -0.64  (-0.78 --0.5) | 5595623.68 (4784949.06-6540595.53) | 226.49 (193.68-264.74) | 7688264.63 (6486399.74-9004932.45) | 222.12 (187.4-260.16) | -0.14  (-0.25 to -0.03) |
| SDI region |  |  |  |  |  |  |  |  |  |  |  |  |  |  |  |
| High SDI | 1967765.15 (1605425-2392663.23) | 550.38 (449.03-669.22) | 2701486.42 (2204736.72-3272751.85) | 664.79 (542.55-805.37) | 0.88  (0.79 - 0.98) | 9686.46 (9077.34-10400.4) | 2.71 (2.54-2.91) | 5545.17 (4916.35-6296.21) | 1.36 (1.21-1.55) | -2.68  (-2.97 - -2.39) | 617219.45 (520912.71-723176.56) | 172.63 (145.7-202.27) | 589959.82 (457292.55-733897.7) | 145.18 (112.53-180.6) | -0.57  (-0.72 to -0.42) |
| High-middle SDI | 3289437.47 (2656696.6-4033315.71) | 627.57 (506.85-769.49) | 4416222.15 (3587321.84-5403197.15) | 723.53 (587.73-885.23) | 0.58  (0.42 - 0.75) | 43133.78 (39738.46-46964.5) | 8.23 (7.58-8.96) | 32796.91 (28875.91-37518.24) | 5.37 (4.73-6.15) | -1.98  (-2.18 - -1.78) | 1984488.14 (1754795.99-2237458.09) | 378.61 (334.78-426.87) | 1856806.62 (1557058.97-2194152.96) | 304.21 (255.1-359.48) | -1.08  (-1.22 to -0.94) |
| Middle SDI | 3583111.33 (2855621.96-4441274.92) | 446.53 (355.87-553.47) | 7027818.91 (5721834.02-8581470.95) | 650.32 (529.47-794.09) | 1.39  (1.26 - 1.52) | 33253.83 (28601.07-39458.57) | 4.14 (3.56-4.92) | 50831.38 (43221.78-60478.21) | 4.7 (4-5.6) | 0.58  (0.44 - 0.72) | 1821164.79 (1510897.55-2206503.1) | 226.95 (188.29-274.97) | 2923595.97 (2430908.23-3492526.83) | 270.54 (224.95-323.18) | 0.72  (0.6 to 0.85) |
| Low-middle SDI | 1753836.47 (1404149.35-2167827.09) | 329.87 (264.1-407.74) | 3650572.84 (2962851.41-4459953.01) | 445.82 (361.83-544.66) | 1.06  (0.93 - 1.2) | 15304.89 (12418.16-19614.07) | 2.88 (2.34-3.69) | 29752.39 (24727.76-36034.01) | 3.63 (3.02-4.4) | 0.77  (0.61 - 0.93) | 812218.1 (658429.16-1022367.55) | 152.77 (123.84-192.29) | 1558514.09 (1293389.11-1867445.12) | 190.33 (157.95-228.06) | 0.75  (0.62 to 0.88) |
| Low SDI | 924865.03 (761592.43-1113838.65) | 364.97 (300.54-439.54) | 2118233.46 (1740290.27-2555489.8) | 390.11 (320.51-470.64) | 0.23  (0.13 - 0.33) | 6069.72 (4454.11-8299.68) | 2.4 (1.76-3.28) | 12510.4 (10045.45-16725.98) | 2.3 (1.85-3.08) | -0.21  (-0.31 - -0.1) | 357963.09 (275867.29-466070.98) | 141.26 (108.86-183.92) | 754640.61 (606617.34-960050.07) | 138.98 (111.72-176.81) | -0.09  (-0.18 to -0.01) |

EAPCs: Estimated annual percentage changes; SDI: socio-demographic index; DALYs: disability-adjusted life years.

Table S20 Peripheral artery disease burden under 65 for female across SDI regions during 1990-2019.

|  | Prevalence | | | | | Deaths | | | | | DALY | | | | |
| --- | --- | --- | --- | --- | --- | --- | --- | --- | --- | --- | --- | --- | --- | --- | --- |
|  | 1990 | | 2019 | | 1990-2019 | 1990 | | 2019 | | 1990-2019 | 1990 | | 2019 | | 1990-2019 |
| location | Number | Rate | Number | Rate | EAPC | Number | Rate | Number | Rate | EAPC | Number | Rate | Number | Rate | EAPC |
| Global | 15252094.41 (11961298.73-18812530.22) | 617.36 (484.15-761.47) | 25764256.22 (20226856.7-31659719.03) | 744.36 (584.38-914.69) | 0.8  (0.68 - 0.92) | 1068.56 (510.58-1992.66) | 0.04 (0.02-0.08) | 2211.12 (1150.54-4111.17) | 0.06 (0.03-0.12) | 1.4  (1.32- 1.48) | 70270.87 (40119.93-114314.39) | 2.84 (1.62-4.63) | 124941.55 (74060.51-201119.67) | 3.61 (2.14-5.81) | 0.9  (0.78 - 1.02) |
| SDI region |  |  |  |  |  |  |  |  |  |  |  |  |  |  |  |
| High SDI | 5144024.22 (4054226.25-6328762.64) | 1438.77 (1133.96-1770.14) | 5291841.59 (4316846.91-6346836.37) | 1302.23 (1062.3-1561.85) | -0.2  (-0.34 - -0.06) | 381.76 (139.27-827.63) | 0.11 (0.04-0.23) | 812.35 (290.12-1802.72) | 0.2 (0.07-0.44) | 2.38  (2.28 - 2.49) | 22928.16 (11862.56-40367.65) | 6.41 (3.32-11.29) | 36330.25 (17869.16-68154.46) | 8.94 (4.4-16.77) | 1.41  (1.32 - 1.49) |
| High-middle SDI | 4518730.01 (3546497.65-5584185.46) | 862.09 (676.61-1065.36) | 7314784.31 (5703746.91-9048219.63) | 1198.41 (934.47-1482.41) | 1.36  (1.2 - 1.53) | 406.26 (161.72-790.28) | 0.08 (0.03-0.15) | 604.02 (249.58-1246.71) | 0.1 (0.04-0.2) | 0.73  (0.55 - 0.91) | 23673.09 (12628.11-38908.57) | 4.52 (2.41-7.42) | 34039.42 (18191.73-58334.84) | 5.58 (2.98-9.56) | 0.72  (0.52 - 0.93) |
| Middle SDI | 3497337.44 (2705481.43-4358130.99) | 435.84 (337.16-543.11) | 8629155.39 (6675156.83-10731635.05) | 798.5 (617.69-993.06) | 2.21  (2.16 - 2.26) | 129.64 (85.34-196.39) | 0.02 (0.01-0.02) | 365.91 (233.33-573.58) | 0.03 (0.02-0.05) | 2.65  (2.55 - 2.76) | 13202.83 (7406.42-22013.31) | 1.65 (0.92-2.74) | 30242.76 (17902.87-49806) | 2.8 (1.66-4.61) | 1.89  (1.76 - 2.02) |
| Low-middle SDI | 1577362.49 (1219302.59-1970064.39) | 296.68 (229.33-370.54) | 3410645.64 (2639582.59-4256208.8) | 416.52 (322.35-519.78) | 1.1  (1.02 - 1.18) | 75.18 (39.94-146.08) | 0.01 (0.01-0.03) | 232.48 (125.93-329.95) | 0.03 (0.02-0.04) | 2.42  (2.17 - 2.68) | 6634.99 (3717.51-10965.7) | 1.25 (0.7-2.06) | 15390.16 (9302.13-23668.45) | 1.88 (1.14-2.89) | 1.31  (1.15 - 1.47) |
| Low SDI | 508153.5 (391886.7-637491.54) | 200.53 (154.64-251.56) | 1106232.43 (853242.23-1388442.69) | 203.73 (157.14-255.71) | -0.01  (-0.11 - 0.09) | 74.94 (20.68-174.86) | 0.03 (0.01-0.07) | 194.44 (53.18-363.41) | 0.04 (0.01-0.07) | 0.59  (0.35 - 0.84) | 3790.23 (1710.71-6998.87) | 1.5 (0.68-2.76) | 8849.96 (3884.52-14646.7) | 1.63 (0.72-2.7) | 0.2  (0.01 - 0.39) |

EAPCs: Estimated annual percentage changes; SDI: socio-demographic index; DALYs: disability-adjusted life years.

Table S21 Ischemic heart disease burden older than 65 for female across SDI regions during 1990-2019.

|  | Prevalence | | | | | Deaths | | | | | DALY | | | | |
| --- | --- | --- | --- | --- | --- | --- | --- | --- | --- | --- | --- | --- | --- | --- | --- |
|  | 1990 | | 2019 | | 1990-2019 | 1990 | | 2019 | | 1990-2019 | 1990 | | 2019 | | 1990-2019 |
| location | Number | Rate | Number | Rate | EAPC | Number | Rate | Number | Rate | EAPC | Number | Rate | Number | Rate | EAPC |
| Global | 26825316.37 (22824380.5-31440937.28) | 14457.55 (12301.24-16945.15) | 55116136.4 (47087723.28-64586782.66) | 13945.41 (11914.07-16341.66) | -0.1  (-0.12 - -0.09) | 2175938.02 (1977532.19-2307643.28) | 1172.73 (1065.79-1243.71) | 3465457.7 (2971778.24-3791480.31) | 876.83 (751.92-959.32) | -1.04  (-1.07 - -1.01) | 30523266.57 (28211725.69-32250441.99) | 16450.57 (15204.76-17381.43) | 45524862.33 (40105047.36-49421698.04) | 11518.64 (10147.32-12504.61) | -1.28  (-1.33 - -1.24) |
| SDI region |  |  |  |  |  |  |  |  |  |  |  |  |  |  |  |
| High SDI | 7239337.52 (6142415.48-8411897.26) | 12137.62 (10298.5-14103.56) | 8889012.57 (7694919.11-10223682.14) | 8774.33 (7595.64-10091.78) | -1.3  (-1.37 - -1.23) | 757282.17 (673450.5-799745.14) | 1269.68 (1129.12-1340.87) | 631558 (511821.74-703721.65) | 623.41 (505.22-694.64) | -2.8  (-2.93 - -2.67) | 9611134.97 (8774267.6-10050898.61) | 16114.23 (14711.12-16851.55) | 6720729.32 (5710126.62-7377062.36) | 6634.02 (5636.46-7281.89) | -3.45  (-3.6 - -3.29) |
| High-middle SDI | 9490836.31 (8044781.82-11186835.84) | 17507.94 (14840.37-20636.58) | 17216602.15 (14569748.64-20333411.45) | 16315.24 (13806.96-19268.87) | -0.16  (-0.2 - -0.12) | 820045.42 (757784.84-859416.9) | 1512.75 (1397.9-1585.38) | 1204360.21 (1038997.19-1324584.42) | 1141.31 (984.6-1255.24) | -0.99  (-1.09 - -0.89) | 11407593.42 (10678582.56-11916343.73) | 21043.82 (19699-21982.32) | 14986778.46 (13211896.22-16392058.39) | 14202.16 (12520.2-15533.87) | -1.44  (-1.6 - -1.28) |
| Middle SDI | 5671361.4 (4788457.6-6732309.34) | 13670.86 (11542.62-16228.29) | 16321431.99 (13825863.86-19305768.23) | 14835.68 (12567.29-17548.35) | 0.36  (0.33 - 0.39) | 357761.44 (321366.29-392802.17) | 862.39 (774.66-946.85) | 983429.23 (843020.06-1095552.48) | 893.91 (766.28-995.82) | 0.41  (0.28 - 0.54) | 5515962.29 (5021906.98-6027667.28) | 13296.27 (12105.35-14529.74) | 13829862.85 (12070961.62-15351953.38) | 12570.92 (10972.13-13954.46) | 0.03  (-0.09 - 0.15) |
[truncated: 34,226 more chars]
